# Dysregulation of Exosome Cargo by Mutant Tau Expressed in Human-induced Pluripotent Stem Cell (iPSC) Neurons Revealed by Proteomics Analyses*[Fn FN2]

**DOI:** 10.1074/mcp.RA120.002079

**Published:** 2020-04-15

**Authors:** Sonia Podvin, Alexander Jones, Qing Liu, Brent Aulston, Linnea Ransom, Janneca Ames, Gloria Shen, Christopher B. Lietz, Zhenze Jiang, Anthony J. O'Donoghue, Charisse Winston, Tsuneya Ikezu, Robert A. Rissman, Shauna Yuan, Vivian Hook

**Affiliations:** ‡ Skaggs School of Pharmacy and Pharmaceutical Sciences, University of California, San Diego, La Jolla, California; § Biomedical Sciences Graduate Program, University of California, San Diego, La Jolla, California; ¶ Department of Neurosciences, School of Medicine, University of California San Diego, La Jolla, California; ‖ Department of Pharmacology and Experimental Therapeutics, Department of Neurology, Alzheimer's Disease Research Center, Boston University, School of Medicine, Boston, Massachusetts; ** VA San Diego Healthcare System, La Jolla, California

**Keywords:** Exosomes, protein identification, neurodegenerative diseases, signaling molecules, alzheimer's disease, neurobiology, networks, biogenesis, dysregulation, human, iPSC neurons, mutant tau

## Abstract

Accumulation and propagation of hyperphosphorylated Tau (p-Tau) is a common neuropathological hallmark associated with neurodegeneration of Alzheimer's disease (AD), frontotemporal dementia and parkinsonism linked to chromosome 17 (FTDP-17), and related tauopathies. Extracellular vesicles, specifically exosomes, have recently been demonstrated to participate in mediating Tau propagation in brain. Exosomes produced by human induced pluripotent stem cell (iPSC)-derived neurons expressing mutant Tau (mTau), containing the P301L and V337M Tau mutations of FTDP-17, possess the ability to propagate p-Tau pathology after injection into mouse brain. To gain an understanding of the mTau exosome cargo involved in Tau pathogenesis, these pathogenic exosomes were analyzed by proteomics and bioinformatics. The data showed that mTau expression dysregulates the exosome proteome to result in 1) proteins uniquely present only in mTau, and not control exosomes, 2) the absence of proteins in mTau exosomes, uniquely present in control exosomes, and 3) shared proteins which were significantly upregulated or downregulated in mTau compared with control exosomes. Notably, mTau exosomes (not control exosomes) contain ANP32A (also known as I1PP2A), an endogenous inhibitor of the PP2A phosphatase which regulates the phosphorylation state of p-Tau. Several of the mTau exosome-specific proteins have been shown to participate in AD mechanisms involving lysosomes, inflammation, secretases, and related processes. Furthermore, the mTau exosomes lacked a substantial portion of proteins present in control exosomes involved in pathways of localization, vesicle transport, and protein binding functions. The shared proteins present in both mTau and control exosomes represented exosome functions of vesicle-mediated transport, exocytosis, and secretion processes. These data illustrate mTau as a dynamic regulator of the biogenesis of exosomes to result in acquisition, deletion, and up- or downregulation of protein cargo to result in pathogenic mTau exosomes capable of *in vivo* propagation of p-Tau neuropathology in mouse brain.

## Abstract (precis)

Accumulation and propagation of Tau is a neuropathological hallmark of Alzheimer's disease, frontotemporal dementia, and related tauopathies. Extracellular vesicles, especially exosomes, participate in Tau propagation in brain, including those produced by human neurons expressing mutant Tau (mTau). The goal of this study was to define the cargo of these mTau exosomes by proteomics analyses. Findings showed that mTau dysregulates the exosome by acquisition, loss, and up- or downregulation of proteins, resulting in pathogenic exosomes capable of propagating Tau neuropathology.

## Graphical Abstract

**Figure:**
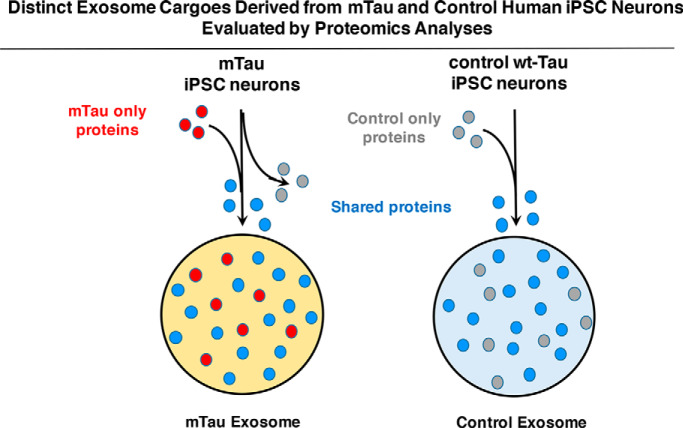

**Highlights**

- Mutant Tau dysregulates exosome cargo generated by human iPSC neurons.
- Expression of P301L and V337M mutations of Tau in human iPSC neurons result in recruitment of distinct proteins to exosomes, and absence of control proteins to exosomes.
- Tau mutations of FTDP-17 result in up- and downregulation of exosome proteins.
- Altered proteome cargo of mTau exosomes may be involved in Tau pathogenesis in vivo in brain.

Neuropathological Tau accumulation occurs in neurodegenerative disorders of Alzheimer's disease (AD)^1^, frontotemporal dementia and parkinsonism linked to chromosome 17 (FTDP-17), progressive supranuclear palsy, chronic traumatic encephalopathy (CTE), and related diseases, commonly known as tauopathies (1–4). Tauopathies are characterized by aggregation of hyperphosphorylated Tau protein into neurofibrillary tangles (NFT) in neurons (2, 3, 5–7). Hyperphosphorylated Tau loses the ability to interact with microtubules, resulting in microtubule destabilization, which has detrimental effects on synaptic functions. In AD, accumulation of NFTs and amyloid plaques occurs in the brain, and NFTs correlate with clinical expression of dementia (8–10). Human Tau oligomers produce impairment of long-term potentiation (LTP) and memory (11). Tau displays transcellular propagation in cortical and hippocampal brain regions, leading to neuronal loss (12–15).

Recent studies suggest that exosomes participate in Tau propagation (16–19). Exosomes are secreted from neurons and many cell types, representing extracellular vesicles (50–150 nm diameter) of endosomal origin (20–23) which function in the removal of cellular components and transcellular shuttling of exosome cargo consisting of proteins, RNAs, lipids, and metabolites (24). Tau is present in exosomes from cerebrospinal fluid (CSF) of AD patients (25) and CTE risk cases (26). Studies of Tau in neuronally-derived exosomes isolated from plasma of AD patients indicate that levels of phosphorylated Tau (p-Tau) predict conversion of MCI (mild cognitive impairment) to AD dementia (17). Notably, injection of these AD plasma exosomes into mouse brain resulted in seeding of Tau aggregation and AD-like neuropathology. In another study, pharmacologic inhibition of exosome synthesis resulted in substantial reduction of Tau propagation in mouse brain, involving microglial mechanisms (16). These findings demonstrate transcellular spreading of Tau by exosomes in brain.

We have developed human induced pluripotent stem cell (iPSC) neurons as a model of human exosome-mediated Tau aggregation and propagation (18, 19). The repeat domain of Tau with the LM mutations P301L and V337M (Tau-RD-LM) of frontotemporal dementia and parkinsonism linked to chromosome 17 (FTDP-17) (27–29) was expressed in human iPSC neurons and resulted in prominent accumulation of intracellular NFTs (18). Moreover, secreted exosomes containing mTau were capable of inducing Tau aggregates and neurotoxicity in normal recipient human iPSC neurons (18). Significantly, injection into mouse brain of these mTau exosomes resulted in the propagation of human Tau in brain regions *in vivo* (18, 19).

These findings beg the question of what is the composition of the protein cargo of mutant Tau (mTau) exosomes generated by human iPSC neurons? Although mTau expression in these neurons results in the insertion of mTau into exosomes, it was not known whether mTau expression modifies the proteome cargo of these exosomes. For this reason, this study conducted global proteomics analyses of mTau exosomes generated by mTau-expressing human iPSC neurons, with comparison to control exosomes from iPSC neurons expressing wild-type Tau (wt-Tau). Proteomics and bioinformatics data analyses included STRING and gene ontology (GO) network and pathway analyses. Data showed that mTau expression dysregulates mTau exosome cargo proteins to result in (1) proteins present only in mTau exosomes, and not controls, (2) the absence of proteins in mTau exosomes which were present only in controls, and (3) shared proteins which were upregulated or downregulated in mTau exosomes. These findings demonstrate that mTau expression in human iPSC neurons dysregulates the protein cargo of mTau exosomes which participates in Tau propagation and neurotoxicity.

## EXPERIMENTAL PROCEDURES

### 

#### 

##### Experimental Design and Statistical Rationale

**Fig. 1.**
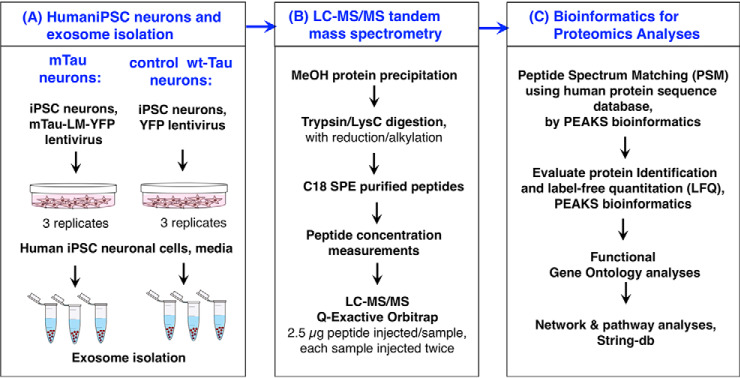
**Workflow of proteomics studies of exosomes produced by mutant Tau and control wild-type Tau human iPSC neurons.** *A*, Mutant Tau (mTau) neurons and control wild-type Tau (wt-Tau) neurons, modeled by human iPSC neurons, were cultured and exosomes isolated. The mTau human induced pluripotent stem cell (iPSC) neurons expressed mTau with the P301L and V337M mutations, generated by lentivrus expression with YFP as a marker. The mTau construct for lentiviral expression is shown in supplemental Fig. S1*A*. The control iPSC neurons express endogenous human wt-Tau, and also expressed YFP as a control for the lentivirus procedure in both groups of cells. Levels of mTau and wt-Tau in the two groups of cells were of similar order of magnitude (supplemental Information S6). Exosomes generated by the neurons were released into in cell culture media (3 biological replicates) which was collected for isolation of exosomes, followed by proteomics studies. *B*, Nano-LC-MS/MS of exosome tryptic digests. Proteins of exosome preparations were collected by MeOH precipitation and subjected to trypsin/LysC digestion, and peptide SPE (solid phase extraction) for nano-LC-MS/MS tandem mass spectrometry. *C*, Bioinformatics of proteomics data. MS/MS mass spectrometry data was subjected to peptide analyses (peptide spectrum matching, PSM) and protein identification, as well as quantification, using PEAKS (v. 8.5) software. Proteomics data was analyzed for functional categories by GO, and for candidate protein networks by STRING-db.

This study was designed to assess proteomics of exosomes isolated from iPSC neurons having prominent accumulation of NFTs resulting from expression of mutant Tau with the P301L and V337M mutations (mTau), compared with that of normal iPSC neurons with no NFTs because these cells express only wild-type Tau (wt-Tau). The overall study design is shown in in Fig. 1.

Human iPSC neurons were prepared from a human tissue biopsy from a non-demented control (NDC). These iPSC neurons were transduced with mTau-YFP lentivirus or control YFP lentivirus, conducted as we have described in serum-free media (18, 19, 30). The lentiviral construct expressed mTau with the P301L and V337M mutations present in the repeat domain of Tau. Control neurons express wt-Tau (endogenous). Both the mTau neurons and control wt-Tau neurons express YFP to monitor the lentivirus transduction. The sample size for each mTau and control group consisted of three biological replicates of neuronal cultures (*n* = 3); this sample size allowed evaluation of statistical comparison (by student's *t*-test, assuming a normal distribution, *p* < 0.05) of the two groups of mTau and control for quantifiable protein identifications. Exosomes were isolated from the media of each neuronal cell replicate culture of the mTau and control groups using ExoQuick-TC (System Biosciences, Palo Alto, CA) (Fig. 1*A*).

Exosome proteins were subjected to trypsin/LysC digestion and SPE (solid phase extraction) of peptides, followed by nano-LC-MS/MS tandem mass spectrometry on a Dionex UltiMate 3000 nano LC and Orbitrap Q-Exactive mass spectrometer (Thermo Fisher Scientific, Carlsbad, CA) (Fig. 1*B*). Each sample was injected twice (two technical replicates) onto the nano-LC-MS/MS system for complete analyses; the order of sample injections was randomized. Raw MS1 and MS2 data were subjected to bioinformatics analyses for peptide and protein identification with label-free quantification by PEAKS Studio 8.5 (Bioinformatics Solutions Inc., Waterloo ON, Canada). PEAKS searched the human protein sequence database (UniprotKB/SwissProt 2018_2 with 71,783 entries) for peptide spectrum matches and protein identification with label-free quantitation (LFQ) (Fig. 1*C*). Proteomics data was subjected to GO and STRING-db functional protein network analyses.

The criteria for inclusion of an identified protein in a biological replicate sample required that the protein was identified in at least one of the two technical replicates (two technical replicates per biological sample). The criteria for inclusion of an identified protein in either the mTau or control groups required that the protein was identified in at least 2 out of the 3 biological replicates per group.

The criteria for inclusion of a quantifiable protein in a biological replicate sample required that the protein be quantifiable (and identified) in at least one of the two technical replicates. The criteria for inclusion of a quantifiable protein in either the mTau and control groups required that the protein be quantifiable (and identified) in at least 2 out of the 3 biological replicates). Quantifiable proteomics data of the mTau and control groups were compared by two-tailed student's *t*-test. The experimental procedures for these phases of the experimental design are described below.

##### Human Induced Pluripotent Stem Cell (iPSC) Neuronal Cultures: mTau Neurons and Control wt-Tau Neurons

Human iPSC neuronal cell cultures derived from control patient biopsies (non-demented) were prepared as described in our previously published protocol (19, 30). Briefly, neural stem cells (NSC) were seeded at a density of 1.5 × 10^5^ cells/cm^2^ on Matrigel-coated (70 μg/ml, BD Bioscience, San Jose, CA) cell culture treated dishes. NSCs were grown to ∼80% confluence, at which time neuronal differentiation was initiated by withdrawal of fibroblast growth factor (bFGF, Biopioneer, San Diego, CA) from the culture media (DMEM-F-12, 1% N-2, 2% B-27, Pen-Strep, 20 ng/ml bFGF).

Four-week differentiated neuronal cultures were transduced by human mTau lentivirus for the 'mTau neurons', and with YFP lentivirus for the 'control wt-Tau neurons', conducted as we have described previously (18, 19, 30). The mTau contains the repeat domain (RD consisting of R1, R2, R3, and R4) with the P301L and V337M mutations (LM). These P301L and V337M mutations have been identified in inherited FTDP-17 dementia (27–29). Expression of YFP was conducted as the control wt-Tau condition of human iPSC neurons expressing endogenous wt-Tau. After 24 h incubation with the mTau and control YFP lentiviral vectors, the virus was removed by washing cells with PBS (phosphate-buffered saline) three times and replenished with fresh media. Three days post-transduction, fresh neuronal media was added to each well and cells were incubated for an additional 3 days, and conditioned media was collected and centrifuged at 1000 rpm for 5 min at 4 °C to clear cell debris. Subsequently, conditioned media was collected from cells every 3–4 days, for several weeks, and used for exosome isolation.

The iPSC neurons were subjected to Gallyas silver staining to assess accumulation of aggregated Tau as neurofibrillary tangles (NFTs), conducted as we have described previously (18). Briefly, cells were fixed with 4% PFA, washed with 5% periodic acid followed by washing in de-ionized water (DDW). Samples were then placed in silver iodide solution for 1 min and washed in 0.5% acetic acid. Samples were then developed in 5% sodium bicarbonate, 0.2% ammonium nitrate, 0.2% silver nitrate, 1% tungstosilicic acid and 9.25% PFA, and examined by light microscopy for the presence of brown NFT densities. Development was terminated by washing in 0.5% acetic acid and washed in distilled water. Samples were then placed in 0.1% gold choride for 5 min, washed in DDW, incubated for 10 min in 1% sodium thiosulfate solution, washed in DDW, and sealed with ProLong-Gold antifade onto a glass slide.

The mTau neurons and control wt-Tau neurons were also collected for LC-MS/MS analyses of mTau and wt-Tau. Cells were prepared as lysates and subjected to the LC-MS/MS analyses used for the exosome proteomics, described in detail below, consisting of MeOH precipitation of proteins, reduction and alkylation, trypsin/LysC digestion, C18 SPE, peptide assay, LC-MS/MS tandem mass spectrometry, and PEAKs search for Tau tryptic peptides from mTau and wt-Tau neurons.

##### Exosome Isolation and NTA (Nanoparticle Tracking Analysis) Analyses

Exosomes were isolated from cell culture media of mTau neurons and control wt-Tau neurons. The media was incubated with ExoQuickTM-TC (EXOTCxxA-1, System Biosciences, Inc.) with rotation overnight at 4 °C, according to the manufacturer's protocol. Media samples were then centrifuged at 1500 × *g* for 30 min at 4 °C. The resultant pellet was resuspended in PBS (phosphate-buffered saline) with protease and phosphatase inhibitor cocktail mixture EDTA-free and stored at −80 °C. Protein concentrations of isolated exosome preparations were determined using a BCA protein assay kit (Pierce Biotechnology, Waltham, MA). Exosomes (10 μg) were subjected to size distribution evaluation by nanoparticle tracking analysis (NTA). Particles were analyzed with a NanoSight LM10 instrument, conducted as described by Mitsuhashi *et al.*, 2013 (31).

##### Trypsin/LysC Digestion of Protein Samples and Preparation for LC-MS/MS

The sample size for each mTau and control wt-Tau conditions consisted of three biological replicates. Exosomes were isolated from each neuronal cell replicate culture as described above. Proteins of isolated exosome samples (100 μg each) were subjected to precipitation in 90% ice-cold methanol with incubation on ice for 15 min, followed by centrifugation for 30 min at 14,000 × *g* (4 °C). The supernatant was discarded, and the resultant pellet of protein was dried in a speedvac. The pellet was then resuspended in 8 m urea, 50 mm Tris-HCl, pH 8 (urea buffer), and sonicated. For reduction, DTT (100 mm DTT stock) was added to a final concentration of 5 mm DTT, incubated at 55 °C for 45 min, and cooled at room temperature (RT) for 5 min. For alkylation, iodoacetamide (200 mm IAA stock in 50 mm Tris-HCl, pH 8) was added to a final concentration of 15 mm IAA and incubated in the dark at RT for 30 min, followed by quenching by addition of DTT (same amount of DTT added as in reduction step). Samples were diluted with 50 mm Tris-HCl, pH 8, to reduce urea to 1.0 m or less. Trypsin/LysC (Promega, Madison, WI), with specificity for cleavage at Arg and Lys residues, was added to each sample at a ratio of 50:1 protein/trypsin concentration (w/w) and incubated at RT for 18–24 h, followed by addition of TFA (trifluoroacetic acid) to 0.5% for quenching. Samples were stored at −70 °C.

Samples were then subjected to peptide purification and desalting by C18 stage tip SPE using Empore C18 wafers (from 3M, Two Harbors, MN), using a protocol described by Rappsilber *et al.*, 2007 (32). The C18 resin was washed with ACN (acetonitrile) and equilibrated with 0.1% TFA; samples were loaded, washed with 0.1% TFA, and eluted with 50% ACN/0.1% TFA. Samples were then dried by speed vac, resuspended in water and sonicated, and peptide concentrations were measured by a total peptide assay kit (Thermo Fisher). Samples were dried using a vacuum centrifuge and stored at −70 °C.

##### LC-MS/MS Tandem Mass Spectrometry

LC-MS/MS was performed on a Dionex UltiMate 3000 nano LC and Orbitrap Q-Exactive mass spectrometer (Thermo Fisher Scientific). Peptide samples (dried by speedvac vacuum centrifuge) were resuspended in 2% ACN, 0.1% TFA to a concentration of 0.6 μg/μl. Each sample was injected twice, at 2.5 μg per injection, onto a nano LC column (75 μm ID, 360 μm OD, 25 cm length) packed with BEH C18 (1.7 μm diameter) solid-phase material and heated to 65 °C with a custom column heater (33) for LC. Samples were injected in a randomized order. LC was conducted with a flow rate of 0.3 μl/min using a 120 min linear gradient of 5% to 25% solvent B, followed by 5 min linear gradient of 25% to 95% B (solvent B = 100% ACN in 0.1% formic acid) with solvent A (0.1% formic acid in water). MS and tandem MS (MS/MS) spectra were recorded as a positive ion data-dependent analysis. MS1 was acquired in profile mode with a 3e6 AGC target, 100 ms maximum injection time, 70,000 resolution (at *m*/*z* 200), and a 310–1250 *m*/*z* window. MS2 was acquired in centroid mode with 1e5 AGC target, 50 ms maximum injection time, 2e3 minimum precursor intensity, 35 s per 10 ppm dynamic exclusion, 17,500 resolution (at *m*/*z* 200), a first mass of *m*/*z* 150, and normalized HCD collision energy set to 28. A full technical MS report is provided in the supplemental information (supplemental Information S1). Raw LC-MS/MS files can be accessed at www.proteomexchange.org under the dataset identifier number PDX016101, or through www.massive.ucsd.edu under the dataset identifier number MSV000084521.

##### Protein Identification

For protein identifications, MS and MS/MS data files were subjected to analyses by PEAKs (v. 8.5) bioinformatics software (34) for peptide identification followed by label-free quantitation (LFQ) analyses (next section). The raw data files were searched against the UniprotKB/SwissProt human protein sequence database (release 2018_02), with addition of the human mTau-RD-LM-YFP protein sequence. The number of entries in this database searched was 71,783. Peptide identifications included searching of a decoy spectrum library of all human proteins, generated by PEAKs from the UniprotKB/SwissProt human protein database. PEAKs parameters were trypsin enzyme (cleavages at Arg and Lys residues, and two missed/nonspecific cleavages permitted). PTMs (post-translational modification were considered for carbomidomethylation on Cys, oxidation of Met, N-terminal acetylation, pyro-Glu, and phosphorylation), with PTM A score confidence of localization ≥13 (*p* < 0.02). Precursor mass error tolerance was 25 ppm, mass tolerance for fragment ion was 0.01 Da, exclusion of keratin and trypsin (known contaminants), and threshold peptide score of −log_10_*p* ≥ 32. The threshold score meets the requirement of < 1% FDR (false discovery rate), which is equivalent to −log_10_*p* > 20. FDR was assessed by searching a decoy human protein sequence data base generated by PEAKs from the UniprotKB/SwissProt human protein database. The technical report of the PEAKs analyses is provided in supplemental Information S2. Protein identifications, based on peptides identified, were determined by PEAKS v. 8.5 using the human UniprotKB/SwissProt database (release 2018_2). The threshold score for protein identification was −log_10_*p* ≥ 55, equivalent to 1% FDR. The supplemental information provides peptide sequences assigned and protein identifications (supplemental Information S3) with Uniprot accession numbers, number of distinct peptides for each protein, % coverage of each protein identified; unique peptides assigned to multiple proteins are indicated in supplemental Information S3. The Master Table (supplemental Information S4) summarizes identified and quantified proteins in mTau exosomes and control exosomes, with analyses of proteins present in only mTau or only control exosomes, and shared proteins with quantifiable protein intensities. Single peptides (which pass the criteria for peptide identification) utilized for protein identification are listed with each of their annotated spectra in supplemental Information S5.

##### Protein Quantification

Label-free quantification (LFQ) of identified proteins was evaluated by PEAKS (v. 8.5) (shown in supplemental Information S4). Extracted ion chromatographs of MS2 peaks were converted to area under the curve (AUC), and the peak areas of MS2 of each peptide spectrum were summed as the relative abundance of each protein. Spectra were filtered for quality parameters prior to LFQ, consisting of peptide quality of >0.3, abundance of 1 × 10^4^. Replicate samples are assessed for retention time (RT) alignment and isotope pattern. The protein area is the sum of the peptide group area. For quantitation, modifications were excluded. Normalization of systematic instrument variations (*i.e.* mass spectrometer instrument) was conducted by LOESS-G using Normalizyer web application (35).

Analytical (technical) replicate reliability was assessed by −log_10_P and quality assessed as 1/log(σ) where σ is the variance between technical runs; −log_10_*p* > 20 is equivalent to 1% FDR. Imputation of quantitative values for “0” of technical replicates was imputed by including a value representative of the bottom 5% of all values. Multiple isoforms in a protein group were manually inspected; all LFQ values were identical for protein Identifications within a protein group. Biological replicate values for protein quantifications of each mTau and control groups were averaged and S.D. (standard deviation) computed. Student's *t* test was used to determine significance (*p* < 0.05) of mTau-RD-LM and control YFP groups.

The PEAKS bioinformatics analyses have been organized in an abbreviated format as the Master Table (supplemental Information S4), which illustrates proteins present in “only mTau,” present in “only YFP control” exosomes, and “shared” proteins present in both groups. The Master Table contains protein identification, quantifiable proteins, and details of protein properties.

##### GO and STRING-db Network Analyses

Protein groups of those present in only mTau, only in YFP control, and shared in both groups were evaluated for GO (gene ontology) pathways and protein interacting networks using STRING-db (https://string-db.org/), an open resource for GO and protein network analyses.

GO enrichment analyses were performed to determine which exosome protein groups significantly assign to a GO term. GO enrichment was determined to be significant with FDR < 1% using Benjamini-Hochberg procedures (36, 37). FDR is assessed by hypergeometric testing, a probability distribution that describes the statistical significance of having hits within the gene sets from this study compared with total genes in the GO pathway.

Protein interaction networks among groups of proteins were assessed for prediction by STRING (version 11.0) (38) (www.string-db.org) which used a database of known protein interactions (databases of DIP, BioGRID, HPRD, IntAct, MINT, and PDB for network analyses). Significant network protein-protein enrichment (PPI) was assessed by a probability *p* value that indicates whether the interacting proteins have more interactions among their group than what would be expected from a randomly selected group of proteins of the same size at the preselected confidence score of 0.7 (39). A network has significantly enriched protein-protein interactions if the number of edges exceeds the expected number of edges of a random group of proteins of the same size.

##### Up- and Downregulated Proteins in mTau and Control Exosomes

For proteins present in both the mTau and control groups, 'shared' proteins, quantifiable protein intensities were used to calculate log_2_ mTau/control ratios and displayed in heat maps, with evaluation of significance by student's *t*-test (*p* < 0.05). Heat maps were generated using Perseus (40) and the heat map script in R studio (https://www.rstudio.com/products/rstudio/) (41).

## RESULTS

### 

#### 

##### Accumulation of Tau Neurofibrillary Tangles (NFTs) in iPSC Neurons Expressing Mutant Tau (mTau) and Absence of NFTs in Neurons Expressing Only Wild-Type Tau (wt-Tau)

Aggregated phospho-Tau in neurofibrillary tangles (NFTs) are the hallmark of Tau toxicity in fronto-termporal dementia (FTD) and related tauopathy-based neurodegenerative diseases. Therefore, we wanted to assess the question of how does the presence of NFTs in human neurons, compared with lack of NFTs, affect exosome cargo which is known to propagate NFTs in brain? We expressed the mutant Tau with P301L and V337M mutations (Tau-RD-LM-YFP) of FTD in human iPSC neurons, and expressed YFP alone and wild-type Tau-RD-YFP as neurons containing only wt-Tau. Highly abundant intracellular NFTs resulted from expression of mTau, but no NFTs were present in neurons containing only wt-Tau, consisting of neurons expressing either YFP with endogenous wt-Tau or neurons expressing wt-Tau-RD-YFP (supplemental Fig. S1). Neurons expressing mTau or YFP had similar levels of mTau and wt-Tau, respectively (supplemental Fig. S2, and supplemental Information S6). These findings support comparison of neurons having accumulation of NFTs with neurons having no NFTs in these studies of exosome cargoes generated by neurons with Tau pathology.

##### Secreted Exosomes from iPSC Neurons Expressing mTau and Control

**Fig. 2.**
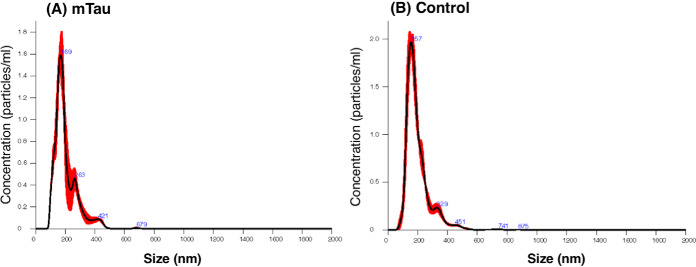
**Nanoparticle tracking analysis (NTA) of mTau and control exosomes.** Mutant Tau (panel *A*) and control (panel *B*) exosomes were subjected to NTA for analyses of particle diameter distribution. Data shows the averaged concentration (particles/ml) per vesicle size (nm); red error bars indicate ± standard error of the mean. The mTau exosomes had a main peak of particles with diameter of 150 nm (∼100–225 nm), and the control exosomes had a main peak of particles with diameter of 150 nm (100–270 nm).

Exosomes were isolated from the mTau and control wt-Tau iPSC neurons (Fig. 1). The distribution of exosome particle sizes was assessed by nanoparticle tracking analysis (NTA) (Fig. 2). The mTau exosomes had a main peak of particles with diameter of 150 nm (∼100–225 nm), and the control exosomes had a main peak of particles with diameter of 150 nm (100–270 nm). These vesicle diameters are consistent with the reported ranges of exosome diameters of 50–150 nm as demonstrated by electron microscopy (42–44). We have shown previously that this preparation of exosomes contains CD63 (18), an exosome marker derived from endosomes (20–23). In addition, the proteomics data of this study indicates the presence of the exosome marker CD81 in the mTau and control exosomes (supplemental Information S4), another exosome marker (20–23).

##### Protein Count of Proteomics Data Acquired from mTau and Control Exosomes

**Fig. 3.**
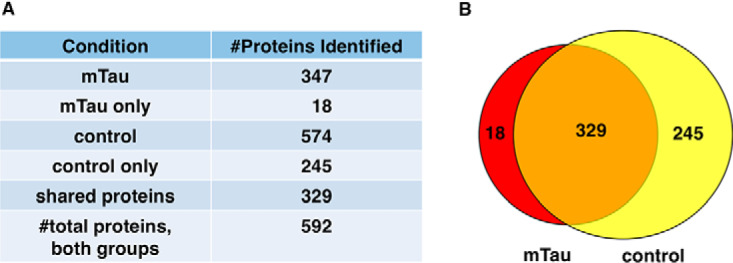
**Protein identification counts in mTau and control exosomes.** *A*, Protein counts. The numbers of proteins identified in mTau and control exosomes are illustrated (panel *A*), including proteins found to be present in only mTau exosomes or only control exosomes, combined with shared proteins present in both mTau and control exosomes. *B*, Venn diagram of unique and shared proteins of mTau and control exosomes. Proteins present in only mTau exosomes, or in only control exosomes are illustrated with shared proteins present in both types of exosomes.

Proteomics identified 592 unique proteins from both groups of exosomes generated by mTau iPSC neurons and by control wt-Tau iPSC neurons (Fig. 3). For the mTau and control exosomes, 347 and 574 proteins were identified, respectively. Eighteen proteins were present in only the mTau exosomes and not in the control exosome, whereas 245 proteins were present in only the control exosomes and not in the mTau exosomes. The mTau and control exosomes shared 329 proteins present in both groups of exosomes.

##### Proteins Present in Only mTau Exosomes

The mTau exosomes contained 18 unique proteins (Table I) which were not present in the control exosomes. The mTau exosomes included the lentiviral expressed mTau protein (shown in supplemental Fig. S3). Tau was not identified in the control YFP exosomes.

**Table I:** Proteins present in only mutant Tau exosomes

Gene name	Protein description	Functions in AD, brain disorders, neurobiology
MAPT*	mutant Tau-RD-LM	· tauopathies and neurodegeneration (1–17)
ACTR3	actin-related protein 3	· ATP binding for actin polymerization (55)
ANP32A	acidic leucine-rich nuclear phosphoprotein 32 family member A	· inhibitor of PP2A phosphatase to dephosphorylate p-Tau (45, 56, 57)
		· mediates memory loss and synaptic dysfunction in Tau mice (46)
		· up-regulated in AD (45)
AP2A1	AP-2 complex subunit α-1	· interacting partner of ADAM10, α-secretase, for membrane trafficking (47)
ATP6V1A	V-type proton ATPase	· lysosomal acidification (49)
	catalytic subunit A	· autophagy (50)
		· AD and neurodegenerative diseases (50)
		· epilepsy (52)
CHID1	chitinase domain-containing protein 1	· inflammation (58)
FTL	ferritin light chain	· interacts with PEN-2, subunit of γ-secretase (59)
		· expression of FTIL increases PEN-2 and γ-secretase activity (59)
GFRA1	GDNF family receptor α-1	· deficiency in Alzheimer's neurons results in cell death (60)
GLO1	glyoxylase	· up-regulated in mutant P301L Tau mice (61)
		· metabolizes methylglyoxal involved in advanced glycation end products (AGEs) which trigger inflammatory signals (62)
HDGF	hepatoma-derived growth factor	· hepatoma-derived growth factor (63)
IFITM3	interferon-induced transmembrane protein 3	· high expression in response to Aβ in APP mice and cultured microglia (64, 65)
PCCB	propionyl-CoA carboxylase beta chain mitochondrial	· associated SNPs for reduced risk of AD and psychosis (66)
SERINC1	serine incorporator 1	· risk factor in frontotemporal dementia (67)
SERPININB6	serpin B6	· elevated expression in female AD brain, but not male AD (68)
SLC1A4	neutral amino acid transporter	· regulator of brain d-serine and neurodevelopment (69)
STX12	syntaxin-12	· participates in syntaxin regulation of protein transport between endosomes and trans-Golgi (70)
TROVE2	60 kDa SS-A/Ro ribonucleoprotein	· involved in autoimmune disease of Sjogren's syndrome (71)
XRCC5	x-ray repair cross-complementing protein 5	· participates in DNA repair (72)

Proteins identified only in the mutant Tau-RD-LM exosomes are listed by gene name and description of protein function. All proteins were identified with FDR less than 1% (see supplemental Information S4, Master Workbook, tab 'Tau only'. *MAPT was identified as the mutant Tau-RD-LM as indicated by the tryptic peptide HVLGGGSVQIVYKPVDLSK containing the P301L mutation.

Several proteins present in only the mTau exosomes have been shown to participate in AD and neurodegeneration with respect to synaptic dysfunction, memory loss, and neuropathology. ANP32A, acidic nuclear phosphoprotein 32 family member A, regulates Tau phosphorylation through inhibition of protein phosphatase-2A (PP2A) which dephosphorylates p-Tau (45). ANP32A is upregulated in AD brain (45) and downregulation of ANP32A in Tau transgenic mice resulted in rescue of memory deficits, amelioration of synaptic dysfunction, and attenuation of AD-like Tau hyperphosphorylation (46). AP2A1, subunit of the adapter protein 2, participates as an interacting partner with ADAM10 for removal of ADAM10 from the plasma membrane (47), suggesting that changes in ADAM10 localization may affect its α-secretase function to limit amyloid-beta (Aβ) formation (48); moreover, AP2/ADAM10 association is increased in AD patients (47). ATP6V1A, V-type proton ATPase catalytic subunit A, participates in lysosomal pH acidification (49); altered v-ATPase has been implicated in numerous neurodegenerative diseases (50, 51). Mutation of the ATP6V1A gene causes encephalopathy with epilepsy (52). IFITM3, interferon induced transmembrane protein 3, is upregulated by Aβ in transgenic AD mouse models and in cultured microglial cells, which may represent an inflammatory role of IFITM3 in AD (53, 54).

**Fig. 4.**
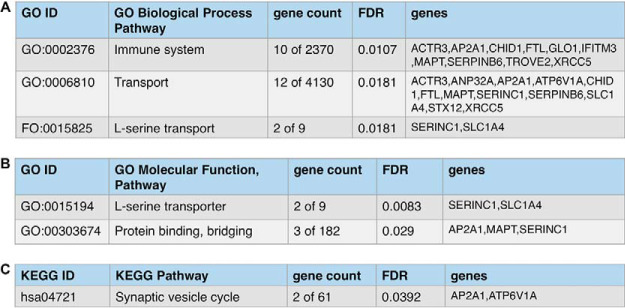
**GO analyses of proteins present only in mutant Tau exosomes.** Gene ontology (GO) analyses of proteins identified in only mTau exosomes indicates involvement in (*A*) biological process pathways, (*B*) molecular function pathways, and (*C*) KEGG pathway. GO enrichment was determined to be significant with FDR estimated at <1% using Benjamini-Hochberg procedures (36, 37).

Gene ontology (GO) and KEGG pathway analyses of proteins present in only the mTau exosomes indicated the biological processes of transport, immune, protein binding, and synaptic vesicle (Fig. 4). Transport, protein binding, and synaptic vesicle systems are certainly involved in synaptic dysfunction of AD (73–75), FTD (76, 77), and Tau neurodegeneration (78–82). Immune systems participate in pathological neuroinflammation observed in AD (83–85), FTD (86, 87), and tauopathies in neurodegenerative diseases (88–90).

Overall, proteins present in only the mTau exosomes have been shown to participate in AD and related neurodegeneration.

##### Proteins Present in Only Control Exosomes, and Absent in mTau Exosomes

**Fig. 5.**
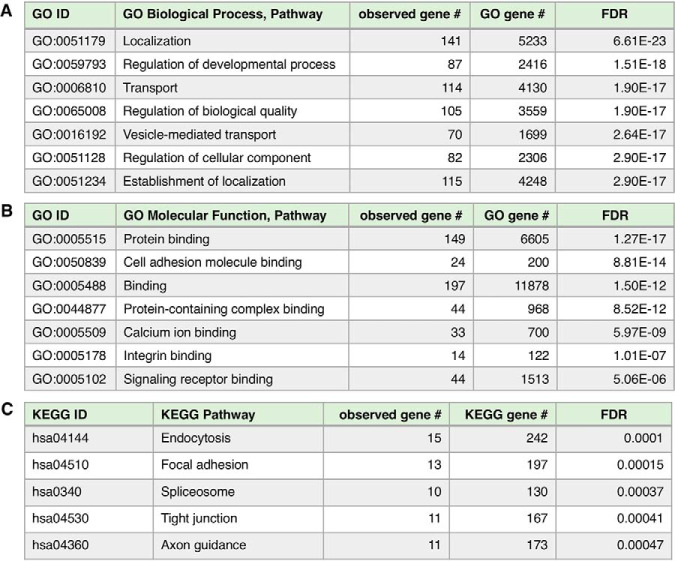
**GO analyses of proteins present in only control exosomes.** Gene ontology (GO) evaluation of proteins identified in only control exosomes illustrates involvement in (*A*) biological processing pathways, (*B*) molecular function pathways, and (*C*) KEGG pathways. GO enrichment was determined to be significant with FDR estimated at <1% using Benjamini-Hochberg procedures (36, 37).

A substantial portion of proteins in the control exosomes (about one-half, 245 out of 574) were absent in mTau exosomes. GO analyses revealed significant enrichment in biological pathways of localization and transport including vesicle-mediated transport and enrichment in molecular functions of protein binding functions in cell adhesion, complexes, calcium, integrin, and signaling (Fig. 5). The enrichment of proteins involved in these pathways in only the control exosomes, and not in the mTau exosomes, suggests that mTau expression alters transport and binding functions of exosomes by dysregulation to prevent packaging of these functional protein systems into the mTau exosomes.

**Fig. 6.**
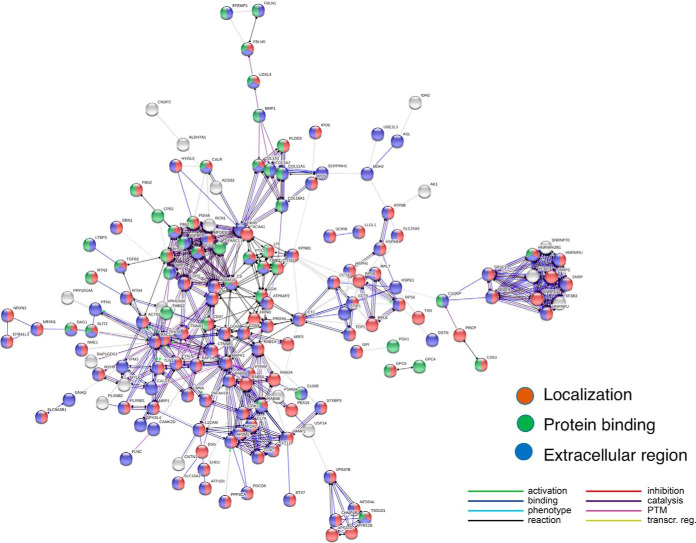
**Protein network analyses of control only proteins.** STRING-db protein interaction network analyses indicated that 234 proteins (out of the 245 proteins identified only in control exosomes) were enriched with respect to known protein-protein interactions, based on String evaluation of protein interactions databases of DIP, BioGRID, HPRD, IntAct, MINT, and PDB (38). Interaction utilized scores set to high confidence (0.7 on a scale of 0–1) that a predicted links exists among proteins (39).

Protein network analyses of proteins present in only control exosomes were assessed by STRING, an open resource for evaluation of protein-protein interaction networks (38), using the STRING database of known and predicted protein-protein interactions combined with confidence scores (https://string-db.org/cgi/about.pl). The control only proteins represent protein interaction networks of localization and protein binding in the extracellular environment (Fig. 6). The analysis shows that 234 out of the 245 control only proteins were in significant networks. The types of protein interactions predicted are illustrated including activation, inhibition, and binding (Fig. 6).

**Fig. 7.**
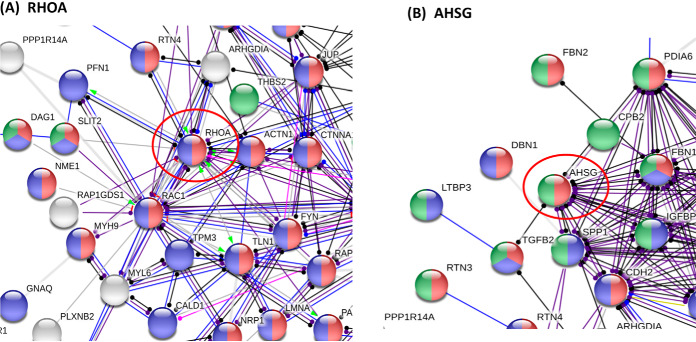
**RHOA and ASHG hub networks of control only proteins.** RHOA (transforming protein RhoA, panel (*A*) and ASHG (alpha-2-HS-glycoprotein, panel (*B*) represent hubs of control only proteins with the highest number of predicted protein interactions numbering 21 and 14, respectively (Table II).

The top hub proteins with the greatest number of interactions displayed 10–20 interactions for each hub (Table II). The interactors for each hub protein are provided in supplemental Information S7. Many of these hub proteins have been reported to participate in AD. RHOA and AHSG hub proteins have the highest number of interactors (Table II). RhoA protein is a GTPase which controls cytoskeleton dynamics and synaptic plasticity; RhoA displays altered localization and expression levels in synapses, neurites, and synapses in brains from human AD and an APP mouse model (Tg 2576), and co-localizes with Tau in inclusions of AD brains (91). Further, RhoA participates in oligomeric Aβ-triggered synaptic loss (92). STRING analyses predicts RhoA as a hub interacting with 21 proteins present in only control exosomes (Fig. 7*A*). Another hub protein, AHSG (α_2_-Heremans-Schmid glycoprotein, also known as fetuin-A) (Fig. 7*B*) is a glycosylated protein related to inflammation (93), which has been found to be decreased in CSF of AD patients (94). Notably, fetuin-A in plasma is associated with the severity of cognitive impairment in mild-to-moderate AD (95). The AHSG hub is predicted by STRING to interact with 14 proteins present in only control exosomes (Table II and supplemental Information S7).

**Table II:** Proteins present in only control exosomes: top hubs of networks

Gene name	Protein description	# Interacting proteins
RHOA	Transforming protein RhoA	21
AHSG	Alpha-2-HS-glycoprotein	14
JUP	Junction plakoglobin	13
RAB5A	Ras-related protein Rab-5A	13
RAC1	Ras-related C3 botulinum toxin substrate 1	13
GDI2	Rab GDP dissociation inhibitor beta	12
SPARCL1	SPARC-like protein 1	12
PDIA6	Protein disulfide-isomerase A6	11
CCT4	T-complex protein 1 subunit delta	10
P4HB	Protein disulfide-isomerase	10
RAP1A	Ras-related protein Rap-1A (Fragment)	10
SNRNP70	U1 small nuclear ribonucleoprotein 70 kDa	10
SPP1	Osteopontin	10

Proteins identified in only the control (YFP) exosomes were analyzed for significant enrichment (conducted as explained in experimental procedures) of protein-protein interactions by String-db. The top hub proteins with the highest number of potential protein interactors are shown here.

Also, the hub protein RAB5A is involved in endocytosis and endosomal recycling compartment (96, 97) and is associated with trafficking of ApoE4, a genetic risk factor for AD (98). RAC1 is a GTPase whose expression is reduced in human AD brain and is increased in plasma of AD patients (99, 100). Inactivation of the RAC1 protein impairs long-term plasticity in mouse hippocampus (101). Evidence suggests that RAC1 may participate in Tau phosphorylation (99). The hub proteins of JUP (102) and GD12 (103) bind to presenilin-1, a component of γ-secretase. SPARC1 is a protein involved in synaptic maintenance and is associated with age-related changes in brain structure (104).

The absence of a substantial portion of control proteins in mTau exosomes (based on the sensitivity of this LC-MS/MS procedure) indicates dysregulation of mTau exosomal cargo with respect to functions of synaptic plasticity, inflammation, subcellular trafficking, and related processes.

##### Proteins Shared by mTau and Control Exosomes; Up- and Downregulation by mTau

**Fig. 8.**
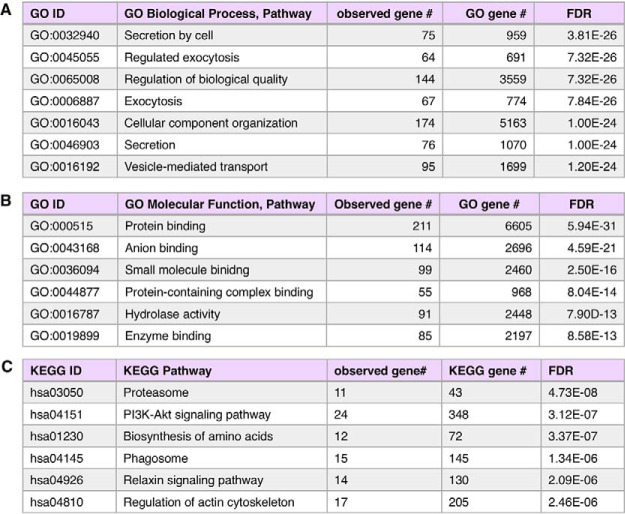
**GO analyses of proteins shared in mTau and control exosomes.** Gene ontology (GO) evaluation of proteins shared by both mTau and control exosomes demonstrates predicted involvement in (*A*) biological processing pathways, (*B*) molecular function pathways, and (*C*) KEGG pathways. GO enrichment was determined to be significant with FDR estimated at <1% (36, 37).

Proteomics results showed that 329 proteins were identified in both mTau and control exosomes (Fig. 3). GO analyses indicated that these shared proteins represent biological pathways of secretion and exocytosis which involve cellular component organization and vesicle-mediated transport (Fig. 8) which are fundamental for exosome functions (20–23). These pathways involve molecular functions for protein binding including complexes, enzymes, and small molecule and anion binding (Fig. 8). These biological and molecular features involve KEGG pathways of the proteasome, signaling pathways for PI3K-Akt and relaxin, actin regulation, phagosome, and amino acid biosynthesis (Fig. 8).

**Fig. 9.**
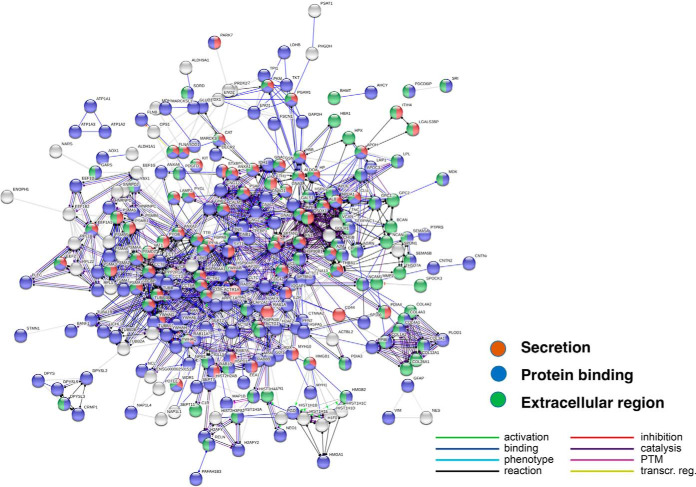
**Protein network analyses of proteins shared in mTau and control exosoms.** STRING-db protein interaction network analyses indicated that 311 proteins (out of the 329 proteins shared between mTau and control exosomes) were enriched for known protein-protein interactions, based on STRING-db evaluation of interaction databases of DIP, BioGRID, HPRD, IntAct, MINT, and PDB (36) and interaction scores set to high confidence (39).

Protein network analyses by STRING predicted the numerous protein-protein interactions of the shared proteins, as illustrated in Fig. 9. The protein hubs of the network represent centers of protein interaction pathways. The top hubs of the shared protein network are listed in Table III. Roles of these hub proteins represent shared exosome functions utilized by mTau and control exosomes, including exocytotic and secretory functions of exosomes.

**Table III:** Shared proteins of mTau and control exosomes: top hubs of networks

Gene name	Protein description	# Interacting proteins
ACTR2	Actin-related protein 2	20
CDC42	Cell division control protein 42 homolog	19
DYNC1H1	Cytoplasmic dynein 1 heavy chain 1	18
GNB1	Guanine nucleotide-binding protein	18
	G(I)/G(S)/G(T) subunit beta-1	
HSPA8	Heat shock cognate 71 kDa protein	18
TF	Serotransferrin (Fragment)	18
PSMA7	Proteasome subunit alpha type-7	17
QSOX1	Sulfhydryl oxidase 1	17
TUBB4B	Tubulin beta-4B chain	17
ALDOA	Fructose-bisphosphate aldolase A	16
RAB5C	Ras-related protein Rab-5C	16

Proteins present in both mTau (mutant Tau-RD-LM-YFP) and control (YFP) exosomes, shared proteins, were analyzed for significant enrichment of protein-protein interactions by String-db. The top hub proteins with the highest number of potential protein interactors are shown in this Table.

**Fig. 10.**
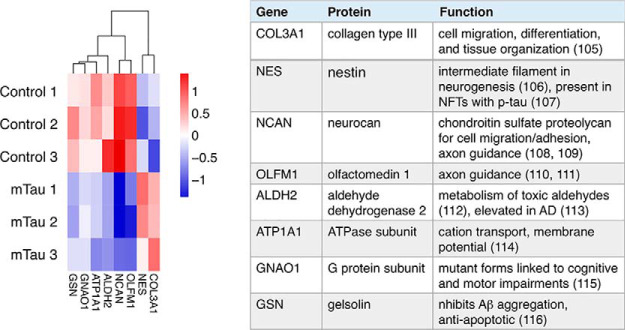
**Heat map of upregulated and downregulated proteins in mTau compared with control exosomes.** Heat map of up- and downregulated proteins shared by mTau and control exosomes. Quantifiable proteins shared between mTau and control exosomes were assessed by computing log_2_(mTau/control) ratios of protein expression. These ratio values were evaluated in heat maps, using Perseus and R-script software (40, 41).

The shared protein components were subjected to label-free quantitation (LFQ) to assess upregulated or downregulated proteins in mTau compared with control exosomes. Heat maps displaying the ratios of log_2_(mTau/control) indicate proteins that were significantly upregulated and downregulated in mTau compared with control exosomes (Fig. 10). COL3A1, collagen type III protein, is upregulated; COL3A1 interacts with cells for regulation of their migration, differentiation, and tissue organization (105). Upregulated NES (nestin), an intermediate filament protein, participates in neurogenesis (106); nestin is present in neurofibrillary tangles (NFTs) with p-Tau in Alzheimer's brains (107).

The mTau exosomes displayed downregulation of NCAN, OLFM1, ALDH2, ATP1A1, GNAO1, and GSN. NCAN is a chondroitin sulfate proteoglycan which participates in cell adhesion, migration, and axonal guidance (108, 109). OLFM1, olfactomedin 1, participates in axon guidance and growth (110, 111). ALDH2, aldehyde dehydrogenase is a mitochondrial enzyme crucial for metabolism of toxic aldehydes in the brain, such as catecholaminergic metabolites (112) and is elevated in Alzheimer's brain putamen (113). ATP1A1 is a Na^+^/K^+^-ATPase for cation transport important for membrane potential and electrical excitability of neurons (114). The GNAO1 protein represents the alpha subunit of the G_o_ heterotrimeric G-protein signaling transducing complex, and mutant forms of GNAO1 are linked to motor and cognitive impairment of epilepsy (115). The GSN gelsolin protein inhibits Aβ aggregation and has antioxidant and anti-apoptotic activities (116).

## DISCUSSION

**Fig. 11.**
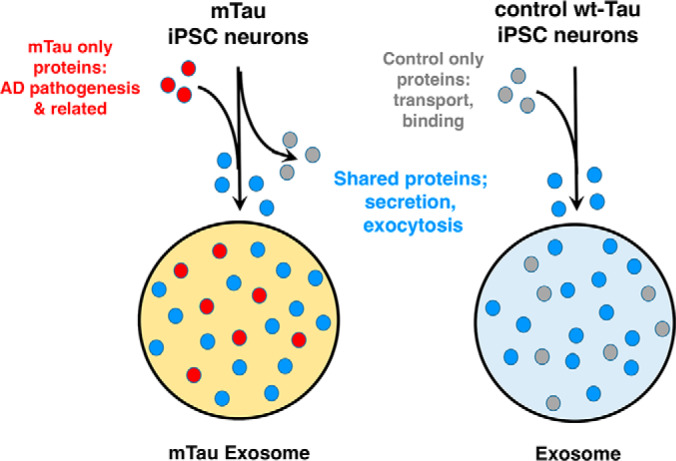
**Mutant Tau dysregulation of exosome cargo: recruitment of factors participating in Alzheimer's disease.** Mutant Tau dysregulates exosome cargo related to AD pathogenesis. Expression of mTau results in packaging of proteins present in only mTau exosomes (red circles; many of these mTau only proteins have been shown to participate in AD pathogenesis (Table I). Expression of mTau also results in the absence of a group of proteins (gray circles, present only in control exosomes) which participate in transport and binding functions (Fig. 5). Both mTau and control exosomes share proteins (blue circles) participating in fundamental exosome functions which include protein binding, transport, vesicle exocytosis, secretion, signaling, and related processes.

This study examined the protein cargo of mTau exosomes derived from mTau iPSC neurons containing prominent NFTs compared with control wt-Tau iPSC neurons having no NFTs. Results can advance understanding of the protein cargo of these mTau exosomes which have been shown to propagate Tau pathology in mouse brain (18, 19). Findings of this study show that mTau dysregulates the exosome proteome to result in (1) proteins present only in mTau, and not control exosomes, (2) the absence of proteins in mTau proteins which are present in only control exosomes, and (3) shared proteins which are upregulated or downregulated in mTau compared with control exosomes (Fig. 11). These data demonstrate that mTau expression dysregulates the proteome cargo of exosomes to result in acquisition, deletion, and up- or downregulation of proteins. The altered exosomal cargo may possibility be associated with the ability of the mTau exosomes to propagate p-Tau neuropathology in mouse brain (18, 19).

The altered exosome cargo resulting from expression of the P301L and V337M Tau mutations suggests that exosome dysregulation may occur in FTDP-17 (frontotemporal dementia and parkinsonism linked to chromosome 17). These mutations represent autosomal dominant inherited forms of FTDP (27, 117–119). FTDP-17 patients display cognitive deficits and Tau aggregation brain pathology that are like the dementia and neuropathology of AD and related neurodegeneration (1–7). Expression of these mutations in transgenic mice result in accumulation of Tau aggregates in brain and cognitive dysfunction (120–123). The Tau mutations of P301L and V337M provide insight into not only FTDP Tau mechanisms but can enhance understanding of Tau functions in neurodegeneration of AD and related dementias. Therefore, analysis of the exosome protein cargo of this study utilizes information from AD studies to infer functional roles of these proteins present in mTau exosomes.

The human iPSC mTau neurons of this study were designed to represent a model of the autosomal dominant nature of the P301L and V337M mutations of FTDP (27, 117–119). The mTau iPSC neurons express the Tau mutations, confirmed by mass spectrometry analyses, combined with the endogenous wt-Tau, which represents a heterozygous model of the P301L and V337M mutations. The control iPSC neurons express the endogenous human wt-Tau.

The unique proteins present in only the mTau exosomes (not control exosomes) have been reported to be involved in Tau phosphorylation and neurodegeneration of AD. Notably, the mTau exosomes contain ANP32A, an endogenous inhibitor of protein phosphatase-2A (PP2A) which dephosphorylates p-Tau (124); ANP32A is also known as I1PP2A (46). PP2A is the major phosphatase for catalyzing dephosphorylation of p-Tau (124) for neuroprotection that reduces formation of NFTs. ANP32A is elevated in human AD brains (45). In human Tau transgenic mice, ANP32 is elevated, and knockdown of ANP32 rescues memory deficits and restores synaptic neurotransmission in electrophysiological studies (46). These findings indicate that mTau-RD-LM expression recruited ANP32A into the mTau exosomes. The presence of ANP32A in the mTau exosomes predicts enhancement of p-Tau, as shown by injection of these exosomes into wild-type mouse brain causing propagation of p-Tau pathology and neuronal degeneration (18, 19).

In addition to ANP32A, proteins which have been reported to participate in Alzheimer's disease and related neurodegeneration were present in only the mTau exosomes, and not in control exosomes (Table I). These “pro-AD” proteins consisted of ATP6V1A (V-type proton ATPase catalytic subunit A) involved in lysosome-autophagy in neurodegeneration (49–52), CHID1 (chitinase domain-containing protein 1) (58) and GLO1 (glyoxylase) (61) in inflammation, GFRA1 (GGNF family receptor alpha-1) in cell death (60), AP2A1 (AP-2 complex subunit apha-1) which interacts with α-secretase (47, 48), FTL (ferritin light chain) interactor of the PEN-2 subunit of γ-secretase (50), IFITM3 (interferon-induced transmembrane protein 3) (64, 65) and SERPININB6 (serpin B6) (68) which are elevated in brains of AD mice or AD patients, PCCB (propionyl-CoA carboxylase beta chain) associated with SNPs of reduced AD risk (66), and SERINC1 (serine incorporator 1), which is a potential risk factor in FTD (67). Among the 18 proteins unique to mTau exosomes, the majority (15 proteins) possess roles in AD and related neurodegeneration based on evidence in the literature.

Notably, a substantial portion of proteins (43%, 245 proteins) in control exosomes were absent in the mTau exosomes. This data indicates that mTau expression resulted in redirecting the routing of nearly half of the normal exosomal proteins in a manner to prevent packaging into the mTau exosomes. The absent proteins represent pathways of localization, transport, vesicle-mediated transport, and protein binding functions in cell adhesion, complexes, calcium, integrin, and signaling (Fig. 5). Thus, components of these pathways have been expelled from the mTau exosomes. Furthermore, the absent proteins represent the lack of hub proteins (based on network analyses) with roles in synaptic functions (RHOA and SPARC1) (91, 92, 104), inflammation (AHSG) (93–95), endocytosis associated with trafficking of ApoE4 (RAB5A) (96–98), presenilin binding (JUP and GD12) (102, 103), and GTPase functions (RAC1) (99–101).

Although there are major differences in the proteome of the mTau and control exosomes, it is important to highlight the finding that they share proteins (329 proteins) common to both exosome types. These shared proteins represent basic exosome functions consisting of vesicle-mediated transport, exocytosis, and secretion processes, which involve molecular protein binding complexes and enzymes. Among these shared proteins, mTau exosomes displayed significant upregulation of COL3A1 and NES, and downregulation of NCAN, OLFM1, ALDH2, ATP1A1, GNAO1, and GSN. These findings show that mTau modulates exosomal processes through up- and downregulation of components.

The dysregulation of the exosome proteome by mTau suggests that mTau expressed in human iPSC neurons may have cellular consequences. Indeed, neurotoxicity occurs upon mTau expression in these model human neurons, resulting in detrimental synaptic density reduction, axon retraction, and enlargement of lysosomes (18). It will be of interest in future studies to define the cellular proteome changes predicted to result during mTau expression, to gain insight into cellular neurotoxic consequences of mTau expression along with biogenesis of pathogenic mTau exosomes.

In plasma of AD patients, neuron-derived exosomes (NDE) contain elevated p-Tau compared with control exosomes, and injection of these AD NDEs into mouse brain resulted in p-Tau aggregation (17). It will be of interest to examine the cargo of the plasma NDEs from AD patients for modulators of p-Tau.

In addition to neurons, microglia cells participate in Tau propagation in brain via secretion of exosomes (125). Depletion of microglia suppressed Tau propagation and, further, inhibition of exosome synthesis reduced Tau spreading in mice. These findings provide evidence that microglia-derived exosomes participate in Tau neuropathology. Moreover, comparison of pathogenic exosome cargoes in neurons and microglia will be of interest to understand cellular proteomes of exosome-mediated Tau pathogenesis.

Human brain proteomics studies have identified proteins associated with cognitive stability in aged patients (70–90 years old) (126), and several of these proteins are present in the mTau exosomes. The unique mTau exosome protein AP2A1 was found to be significantly associated with cognitive stability. Among proteins absent in mTau exosomes, the P4HB protein hub is positively associated, and SPP1 is negatively associated with cognitive stability. For proteins shared in mTau and control exosomes, the hub proteins CDC42 and ALDOA are positively associated with cognitive stability. The presence of mTau exosomal proteins which are associated with cognitive stability in human brain implicates roles for several mTau exosomal proteins in cognitive function.

Tau proteins, mutant and wild-type, in human brain are heterogeneous in molecular forms with respect to different repeat domains and proteolytic fragments (1, 127–132). The finding in this study that the P301L and V337M Tau mutations result in dysregulation of exosome protein cargo leads the question of what are the effects of other Tau isoforms on exosome cargoes and pathogenicity? For example, Tau exists in several forms containing 3 or 4 repeat domains, generated by alternative splicing (1, 127–129). It will be of interest in future studies to compare the P301L and V337M mutations of Tau isoforms with variant repeat domains, as well as wild-type Tau with variant repeat domains, for effects on exosome cargoes. Furthermore, investigation of Tau proteolytic fragments (130–132), with and without mutations, will be important to assess resultant exosome cargoes in propagation of Tau pathology. Knowledge of the roles of heterogeneous isoforms of Tau in exosome-mediated pathogenicity will be important for understanding mechanisms of neurodegeneration and dementia.

Numerous gene mutations are linked to tauopathies including FTD (133–136), AD (137–139), and related neurodegenerative diseases (127, 140–144). Familial FTD is caused by mutations of MAPT (Tau), *C90RF72*, and PGN (progranulin) genes (133). Familial AD (FAD) is linked to mutations of PS1, PS2, and APP (137–139), whereas sporadic AD has no known linked gene mutations. Future comparisons of exosome proteome cargoes in different genetic and sporadic neurodegenerative disorders will be important to provide insight into cargo mechanisms of extracellular vesicle-mediated Tau pathogenesis.

## DATA AVAILABILITY

LC-MS/MS data files can be accessed at www.proteomexchange.org under the dataset identifier PDX016101, or through www.massive.ucsd.edu under MSV000084521. Data analysis information is provided in supplemental Information S1–S5.

## Supplementary Material

Supplemental Figures 1-3

Legends for Supplemental Materials 1-7

Supplement 1. LCMS report

Supplement 2. PEAKS report

Supplement 3. Peptides and Proteins

Supplement 4. Master Table

Supplement 5. Single peptide ID Spectra

Supplement 6 Tau peptides neurons

Supplement 7. Hub proteins

## Footnotes

^1^ The abbreviations used are:

AD: Alzheimer's disease
ADAM10: ADAM metallopeptidase domain 10
AHSG: alpha 2 HS glycoprotein
ALDH2: aldehyde dehydrogenase
ALDOA: fructose-bis phosphate aldolase A
ANP32A: acidic nuclear phosphoprotein 32 family member A
AP2A1: subunit of the adapter protein 2
APP: amyloid precursor protein
ASHG: alpha-2-HS-glycoprotein
ATP1A1: Na^+^/K^+^-ATPase
ATP6V1A: V-type proton ATPase catalytic subunit A
bFGF: basic fibroblast growth factor
CDC42: cell devision control protein 42 homolog
CD63: CD63 antigen
CD81: tetraspanin
CHID1: chitinase doomain-containin protein 1
COL3A1: collagen alpha-1(III) chain
CSF: cerebrospinal fluid
CTE: chronic traumatic encephalopathy
DMEM: Dulbecco's modified eagle medium
FDR: false discovery rate
FTDP-17: frontotemporal dementia and parkinsonism linked to chromosome 17
FTL: ferritin light chain
GD12: rab GDP dissociation inhibitor beta
GFRA1: GGNF family receptor alpha-1
GLO1: glyoxylase
GNAO1: alpha subunit of the Go heterotrimeric G-protein signaling transducing complex
GO: gene ontology
GSN: gelsolin
IFITM3: interferon induced transmembrane protein 3
iPSC: induced pluripotent stem cell
JUP: junction plakoglobin
KEGG: Kyoto Encyclopedia of Genes and Genomes
MAPT: Tau
MCI: mild cognitive impairment
MS: mass spectrometry
mTau: mutant Tau-RD-LM
nano-LC-MS/MS: nano-liquid chromatography tandem mass spectrometry
NCAN: chondroiti sulfate proteolycan
NDE: neuron-derived exosomes
NES: nestin
NFT: neurofibrillary tangles
NSC: neural stem cells
NTA: nanoparticle tracking analysis
OLFM1: olfactomedin 1
p-Tau: hyperphosphorylated Tau
P4HB: prolyl 4-hydroxylase subunit beta
PBS: phosphate-buffered saline
PCCB: propionyl-CoA carboxylase beta chain
PGN: progranulin
PP2A: protein phosphatase
PS1: presenilin 1
PS1: presenilin 2
PTM: post-translational modification
RAB5A: ras-related protein Rab-5A
RAC1: ras-related C3 botulinum toxin substrate 1
RD: repeat domain
RHOA: ras homolog family member A
SERINC1: serine incorporator 1
SERPININB6: serpin B6
SNP: single nucleotide polymorphism
SPARC1: secreted protein acidic and cysteine rich
SPE: solid phase extraction
SPP1: osteopontin
YFP: yellow fluorescent protein.
