# Supplementary material for: Dysregulation of Exosome Cargo by Mutant Tau Expressed in Human-induced Pluripotent Stem Cell (iPSC) Neurons Revealed by Proteomics Analyses
Source: Mol Cell Proteomics. 2020 Apr 15;19(6):1017–34. doi: 10.1074/mcp.RA120.002079 (PMC7261814; doi:10.1074/mcp.RA120.002079)
Supplement: Supplemental Figures 1-3 [file 160003_0_supp_506381_q8dys3.pdf]

Supplemental Figures

Supplemental Figure 1. Accumulation of Tau neurofibrillary tangles (NFTs) in human iPSC neurons expressing mutant Tau, and absence NFTs in neurons expressing wild-type Tau. The hallmark of mutant Tau is its aggregation as neurofibrillary tangles (NFTs) in neurons intracellularly. Aggregated Tau in accumulated NFTs was assessed by silver staining of the human iPSC neurons expressing (a) mutant Tau, by expression of mutant Tau-RD-LM-YFP, (b) wild-type (WT) Tau expressed endogenously in the human iPSC neurons, also expressing YFP as control, and (c) WT Tau, by expression of WT Tau-RD-YFP. Abundant NFTs are illustrated in neurons expressing mutant Tau (mTau) (panel a), but neurons with WT Tau have no NFTs (panels b and c).

Supplemental Figure 2. Tau tryptic peptide fragments in human 'mTau iPSC neurons' and in 'control wt-Tau neurons'.
<u>(a) Mutant Tau with P301L and V337M mutations in the repeat domain of Tau.</u> Mutant Tau contains the P301L and V337M mutations located within the repeat domain of Tau. The wild-type Tau (wt-Tau) contains the repeat domain of Tau.
<u>(b) Identification of mTau and wt-Tau tryptic peptides in 'mTau iPSC neurons' and in 'control wt-Tau neurons,' respectively.</u> The mTau and wt-Tau proteins in the human iPSC neurons were subjected to proteomics analyses by trypsin digestion and LC-MS/MS as described in the methods. The tryptic peptides were mapped to the human Tau primary sequence (MAPT P10636). The mTau iPSC neurons expressed the P301L and V337M mutations, as well as other sequence domains of Tau. The control neurons expressed wt-Tau tryptic fragments. Quantification (shown in supplement 6) showed similar abundances (similar order of magnitude) of mutant and the wild-type tryptic peptides of Tau in mTau neurons and in wt-Tau neurons, respectively.

Supplemental Figure 3. Identification of mTau in exosomes by LC-MS/MS tandem mass spectrometry. Exosomes from the mTau iPSC neurons contain the mTau, shown by the mutant tryptic peptide HVL*GGGSVQIVYKPVDLSK (* indicates the P301L mutation). It is noted that exosomes from wt-Tau iPSC neurons had no detectable Tau in the proteomics data.

Supplemental Figure 1

(a) Mutant Tau,
mTau-RD-LM-YFP

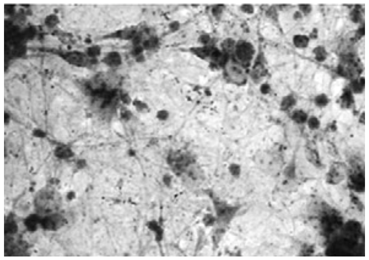

(b) WT Tau,
endogenous,
with YFP

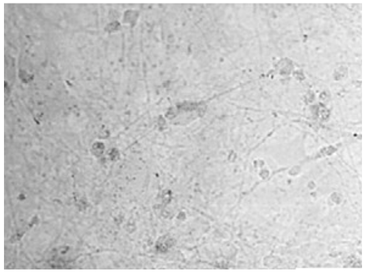

(c) WT Tau,
WT Tau-RD-YFP

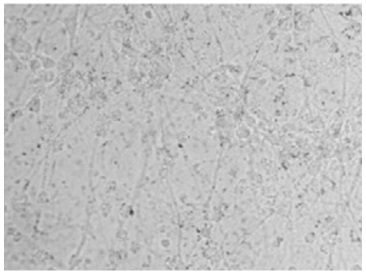

Supplemental Figure 2

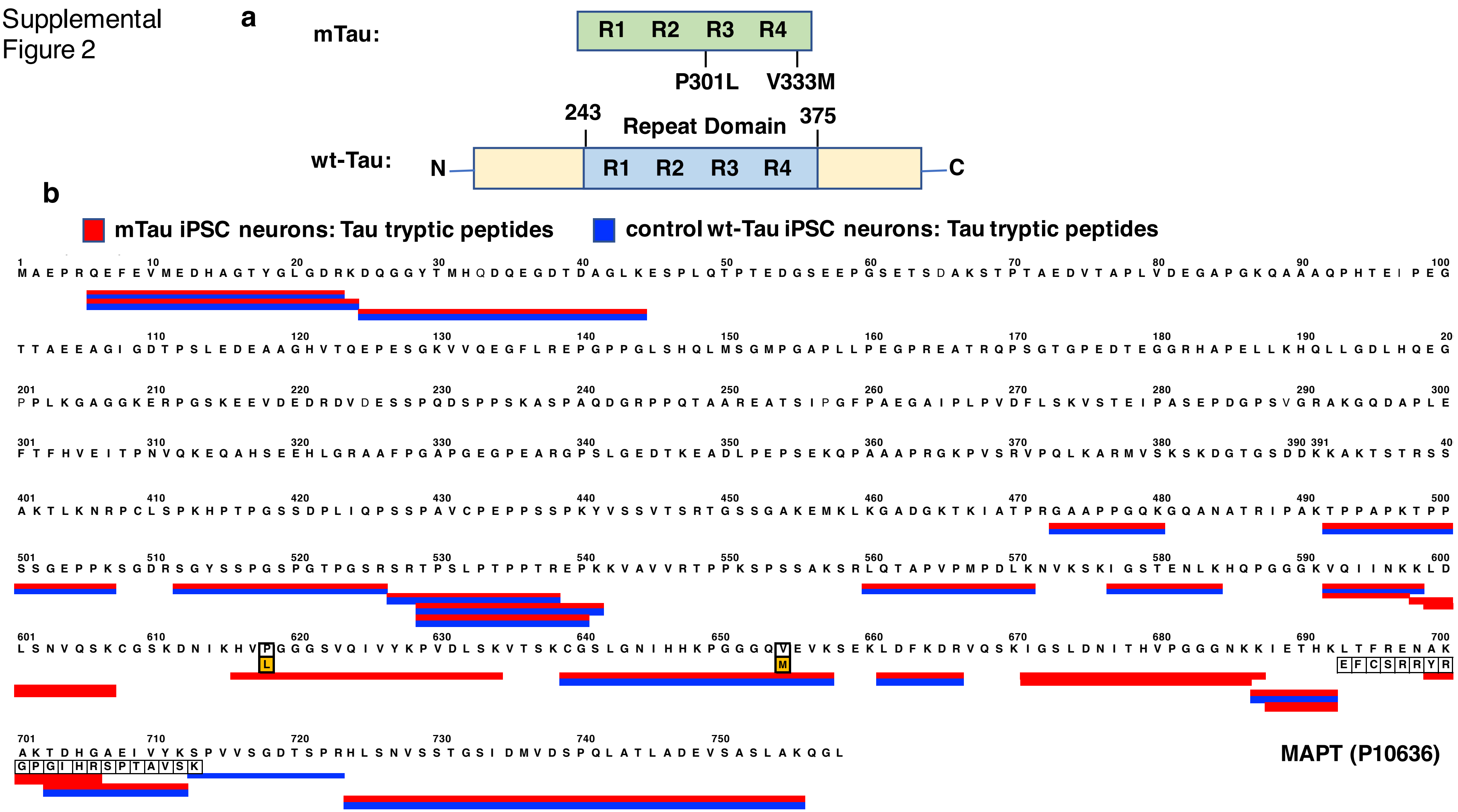

Supplemental Figure 3

mTau exosomes: Tau tryptic peptides **mutation**

MAPT RDLM mutation (P10636MUT)

560 570 580 590 600 610 620 630 640 650 660
MLQTAPVPMPDLKNVKSKIGSTENLKHQPGGGKVQIINKKLDLSNVQSKCGSKDNIKHVLGGGSVQIVYKPVDLSKVTSKCGSLGNIHHKPGGGQMEVKSEK

661 670 680 690 700 710
LDFKDRVQSKIGSLDNITHVPGGGNKKIETHKEFCSRRYRGPGIHRSPTAVSK
