# Supplementary material for: Dysregulation of Exosome Cargo by Mutant Tau Expressed in Human-induced Pluripotent Stem Cell (iPSC) Neurons Revealed by Proteomics Analyses
Source: Mol Cell Proteomics. 2020 Apr 15;19(6):1017–34. doi: 10.1074/mcp.RA120.002079 (PMC7261814; doi:10.1074/mcp.RA120.002079)
Supplement: Legends for Supplemental Materials 1-7 [file 160003_0_supp_506382_q8dys3.pdf]

Legends for Supplemental Materials

Supplement 1. LC-MS/MS report. The LC-MS/MS report of samples analyzed by the Q-Exactive mass spectrometer LC system.

Supplement 2. PEAKS report. PEAKS report for the peptide and protein analyses of LC-MS/MS data.

Supplement 3. Peptide sequences and protein identifications. PEAKS results for peptides assigned and protein identifications with Uniprot accession numbers, number of distinct peptides for each protein, % coverage, and related.

Supplement 4. Master Table of proteins in mTau exosomes and control exosomes. The master table summarizes the complete proteomics data sets utilized in this study.

Supplement 5. SIngle peptide identifications of proteins. A Table of single peptide identifications is provided (with each peptide numbered), followed by the annotated spectra for each peptide (numbered by peptide).

Supplement 6. LC-MS/MS analyses of Tau in mTau neurons and control wt-Tau neurons. Cell lysates of mTau iPSC neurons and control wt-Tau iPSC neurons were subjected to trypsin digestion and LC-MS/MS tandem mass spectrometry for proteomics analyses of Tau (MAPT). The workbook of supplement 6 shows the presence of the P301L and V337M Tau mutations in the mTau neurons. Tau tryptic peptides in the mTau and wt-Tau neurons.

Supplement 7. Hub proteins and interactors. Hub protein interactors present in networks of only control exosomes, and hubs present in networks of shared proteins.
