# Supplementary material for: Dysregulation of Exosome Cargo by Mutant Tau Expressed in Human-induced Pluripotent Stem Cell (iPSC) Neurons Revealed by Proteomics Analyses
Source: Mol Cell Proteomics. 2020 Apr 15;19(6):1017–34. doi: 10.1074/mcp.RA120.002079 (PMC7261814; doi:10.1074/mcp.RA120.002079)
Supplement: Supplement 1. LCMS report [file 160003_0_supp_506361_q8dy2b.pdf]

Supplement 1. LC-MS-MS Report

Samples were resuspended in 2% ACN, 0.1% TFA to a total peptide concentration of 625 ng/uL
Each sample was injected twice, 2.5 ug total peptide per injection

Column was 25 cm long, heated to 60 °C

Solvent A - water, 0.1% formic acid
Solvent B - acetonitrile, 0.1% formic acid

Nano-LC gradient:

A	B	C	D
Time (min)	Flow (nL/min)	% solvent A	% Solvent B
0	350	99	1
0.1	300	95	5
120	300	75	25
125	300	5	95
135	300	5	95
135.1	300	99	1
145	350	99	1

<u>Q-Exactive MS Report:</u>
Thermo Scientific SII for Xcalibur Method - Next Pages

```
Thermo Scientific SII for Xcalibur Method

---- Overview ----
Name: New Instrument Method
Comment:
Run time: 145.000 [min]
Instrument: Hook_nano on 2hyl8d2
Description:
---- Script ----
initial      Instrument Setup
             PumpModule.LoadingPump.%A.Equate: "%A"
             PumpModule.LoadingPump.%B.Equate: "%B"
             PumpModule.LoadingPump.%C.Equate: "%C"
             PumpModule.LoadingPump.Pressure.LowerLimit: 0 [bar]
             PumpModule.LoadingPump.Pressure.UpperLimit: 600 [bar]
             PumpModule.LoadingPump.MaximumFlowRampUp: 101 [µl/min²]
             PumpModule.LoadingPump.MaximumFlowRampDown: 10 [µl/min²]
             PumpModule.NC_Pump.%A.Equate: "%A"
             PumpModule.NC_Pump.%B.Equate: "%B"
             PumpModule.NC_Pump.Pressure.LowerLimit: 0 [psi]
             PumpModule.NC_Pump.Pressure.UpperLimit: 9800 [psi]
             PumpModule.NC_Pump.MaximumFlowRampUp: 0.300 [µl/min²]
             PumpModule.NC_Pump.MaximumFlowRampDown: 0.300 [µl/min²]
             ColumnOven.TempCtrl: Off
             Sampler.LowDispersionMode: Off
             Sampler.WashSpeed: 4.000 [µl/s]
             Sampler.WashVolume: 50.000 [µl]
             Sampler.PunctureDepth: 7.000 [mm]
             Sampler.SampleHeight: 3.000 [mm]
             Sampler.WasteSpeed: 4.000 [µl/s]
             Sampler.DispenseDelay: 2.000 [s]
             Sampler.DispSpeed: 2.000 [µl/s]
             Sampler.DrawSpeed: 0.500 [µl/s]
             Sampler.DrawDelay: 5.000 [s]
             Sampler.RinseBetweenReinjections: No
             Sampler.InjectMode: UserProg
             Sampler.LoopWashFactor: 2.000
             Sampler.PumpDevice: "NC_Pump"
             Sampler.ReagentAVial: R1
             Sampler.ReagentBVial: R2
             Sampler.ReagentCVial: R3
             Sampler.ReagentDVial: R4
             Sampler.ReagentEVial: RA1
             Sampler.ReagentFVial: RA1
             Sampler.ReagentGVial: RA1
             Sampler.ReagentHVial: RA1
             Sampler.TempCtrl: On
             Sampler.Temperature.Nominal: 7.0 [°C]
             Sampler.ReadyTempDelta: None
             Sampler.Temperature.LowerLimit: 4.0 [°C]
```

Thermo Scientific SII for Xcalibur Method

```
            Sampler.Temperature.UpperLimit: 45.0 [°C]
            Sampler.UdpSyringeValve Waste
            Sampler.UdpMixNeedleWash 100 [µl]
            Sampler.UdpSyringeValve Needle
            Sampler.UdpDraw ReagentAVial,
                            (2*Sampler.NeedleVolume)+Sampler.LoopVolume,
                            GlobalSpeed,
                            4 [mm]
            Sampler.UdpMixWait 4 [s]
            Sampler.UdpDispense Drain,
                                0.000,
                                2 [µl/s],
                                5 [mm]
            Sampler.UdpInjectValve Load
            Sampler.UdpDraw SampleVial,
                            Sampler.Volume,
                            0.2 [µl/s],
                            3.000 [mm]
            Sampler.UdpMixWait 4 [s]
            Sampler.UdpDispense Drain,
                                0.000,
                                2 [µl/s],
                                5 [mm]
            Sampler.UdpDraw ReagentAVial,
                            (Sampler.LoopVolume-Sampler.Volume)*0.5+Sampler.NeedleVolume,
                            GlobalSpeed,
                            2 [mm]
            Sampler.UdpMixWait 4 [s]
            Sampler.UdpInjectValve Inject
            Sampler.UdpMixWait Sampler.LoopVolume/PumpModule.NC_Pump.Flow.Nominal*1.1*60
            Sampler.UdpInjectValve Load
            Sampler.UdpInjectMarker
            Sampler.UdpSyringeValve Waste
            Sampler.UdpMixNeedleWash 100.000 [µl]
0.000 [min] Inject Preparation
            Wait PumpModule.LoadingPump.Ready And PumpModule.NC_Pump.Ready And ColumnOven.Ready
0.000 [min] Inject
            Sampler.Inject
0.000 [min] Start Run
            PumpModule.NC_Pump.NC_Pump_Pressure.AcqOn
0.000 [min] Run
            PumpModule.LoadingPump.Flow.Nominal: 0.000 [µl/min]
            PumpModule.LoadingPump.%B.Value: 0.0 [%]
            PumpModule.LoadingPump.%C.Value: 0.0 [%]
            PumpModule.LoadingPump.Curve: 5
            PumpModule.NC_Pump.Flow.Nominal: 0.350 [µl/min]
            PumpModule.NC_Pump.%B.Value: 1.0 [%]
            PumpModule.NC_Pump.Curve: 5
```

```
Thermo Scientific SII for Xcalibur Method

0.100 [min]
            PumpModule.NC_Pump.Flow.Nominal: 0.300 [µl/min]
            PumpModule.NC_Pump.%B.Value: 5.0 [%]
            PumpModule.NC_Pump.Curve: 5
20.000 [min]
            PumpModule.LoadingPump.Flow.Nominal: 0.000 [µl/min]
            PumpModule.LoadingPump.%B.Value: 0.0 [%]
            PumpModule.LoadingPump.%C.Value: 0.0 [%]
            PumpModule.LoadingPump.Curve: 5
120.000 [min]
            PumpModule.NC_Pump.Flow.Nominal: 0.300 [µl/min]
            PumpModule.NC_Pump.%B.Value: 25.0 [%]
            PumpModule.NC_Pump.Curve: 5
125.000 [min]
            PumpModule.NC_Pump.Flow.Nominal: 0.300 [µl/min]
            PumpModule.NC_Pump.%B.Value: 95.0 [%]
            PumpModule.NC_Pump.Curve: 5
135.000 [min]
            PumpModule.NC_Pump.Flow.Nominal: 0.300 [µl/min]
            PumpModule.NC_Pump.%B.Value: 95.0 [%]
            PumpModule.NC_Pump.Curve: 5
135.100 [min]
            PumpModule.NC_Pump.Flow.Nominal: 0.300 [µl/min]
            PumpModule.NC_Pump.%B.Value: 1.0 [%]
            PumpModule.NC_Pump.Curve: 5
145.000 [min]
            PumpModule.NC_Pump.Flow.Nominal: 0.350 [µl/min]
            PumpModule.NC_Pump.%B.Value: 1.0 [%]
            PumpModule.NC_Pump.Curve: 5
145.000 [min] Stop Run
            PumpModule.NC_Pump.NC_Pump_Pressure.AcqOff
```

Method of Q Exactive

Overall method settings

**Global Settings**		
Use lock masses	off	
Lock mass injection	–	
Chrom. peak width (FWHM)	15	s
**Time**		
Method duration	145.00	min
**Customized Tolerances (+/-)**		
Lock Masses	–	
Inclusion	–	
Exclusion	–	
Neutral Loss	–	
Mass Tags	–	
Dynamic Exclusion	10.0	ppm

Experiment

Full MS / dd-MS² (TopN)

**General**		
Runtime	0 to 135	min
Polarity	Positive	
In-source CID	0.0	eV
Default charge state	2	
Inclusion	–	
Exclusion	–	
Tags	–	
**Full MS**		
Microscans	1	
Resolution	70,000	
AGC target	3e6	
Maximum IT	100	ms
Number of scan ranges	1	
Scan range	310 to 1250	m/z
Spectrum data type	Profile	
**dd-MS² / dd-SIM**		
Microscans	1	
Resolution	17,500	
AGC target	1e5	
Maximum IT	50	ms
Loop count	15	
MSX count	1	
TopN	15	
Isolation window	1.5	m/z
Isolation offset	0.0	m/z
Scan range	200 to 2000	m/z
Fixed first mass	150.0	m/z

(N)CE / stepped (N)CE	nce: 28
Spectrum data type	Centroid
**dd Settings**	
Minimum AGC target	1.00e2
Intensity threshold	2.0e3
Apex trigger	–
Charge exclusion	unassigned, 1
Peptide match	Preferred
Exclude isotopes	on
Dynamic exclusion	35.0 s
If idle ..	do not pick others

Setup

<u>Tunefiles</u>

General

Switch Count	0
Base Tunefile	C:\Xcalibur\methods\Chris_GeneralNanoTune_225kV.mstune

<u>Contact Closure</u>

General

Used	False
Start in Closed	True
Switch Count	0

<u>Syringe</u>

General

Used	False	
Start in OFF	True	
Stop at end of run	False	
Switch Count	0	
**Pump setup**		
Syringe type	Hamilton	
Flow rate	3.000	µL/min
Inner diameter	2.303	mm
Volume	250	µL

<u>Divert Valve A</u>

General

Used	False
Start in 1-2	True
Switch Count	0

<u>Divert Valve B</u>

General

Used	False
Start in 1-2	True
Switch Count	0
