# Supplementary material for: Dysregulation of Exosome Cargo by Mutant Tau Expressed in Human-induced Pluripotent Stem Cell (iPSC) Neurons Revealed by Proteomics Analyses
Source: Mol Cell Proteomics. 2020 Apr 15;19(6):1017–34. doi: 10.1074/mcp.RA120.002079 (PMC7261814; doi:10.1074/mcp.RA120.002079)
Supplement: Supplement 2. PEAKS report [file 160003_0_supp_506362_q8dy2b.pdf]

1. Notes

2. Result Statistics

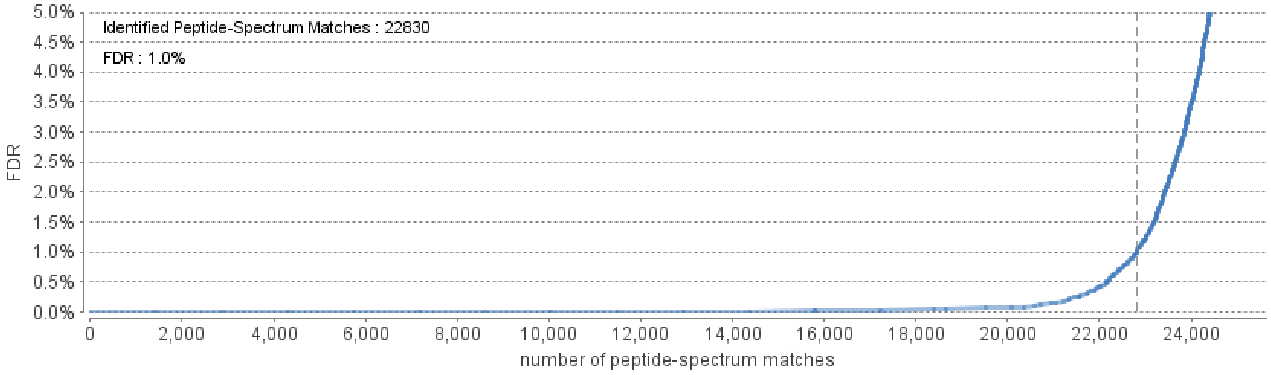

Figure 1. False discovery rate (FDR) curve. X axis is the number of peptide-spectrum matches (PSM) being kept. Y axis is the corresponding FDR.

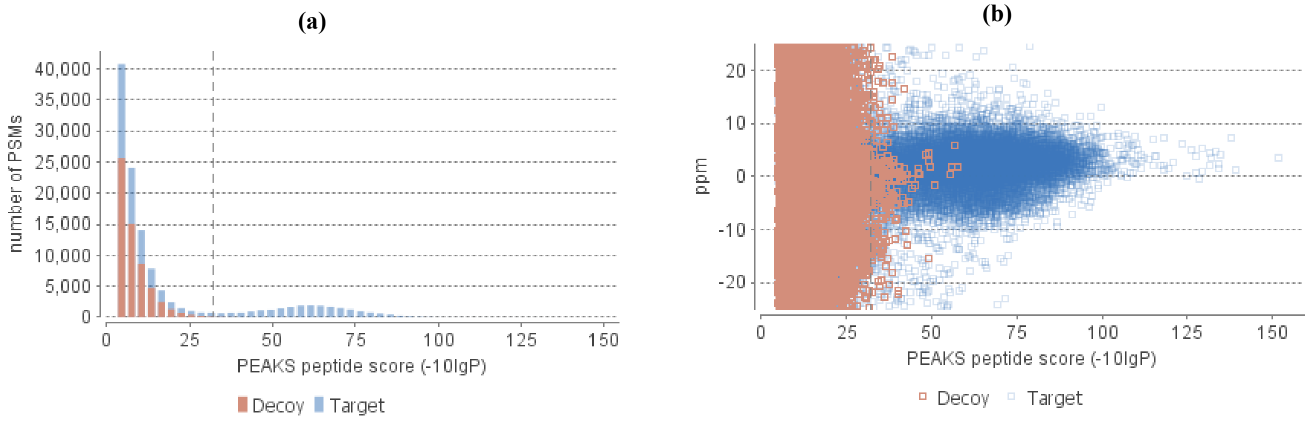

Figure 2. PSM score distribution. **(a)** Distribution of PEAKS peptide score; **(b)** Scatterplot of PEAKS peptide score versus precursor mass error.

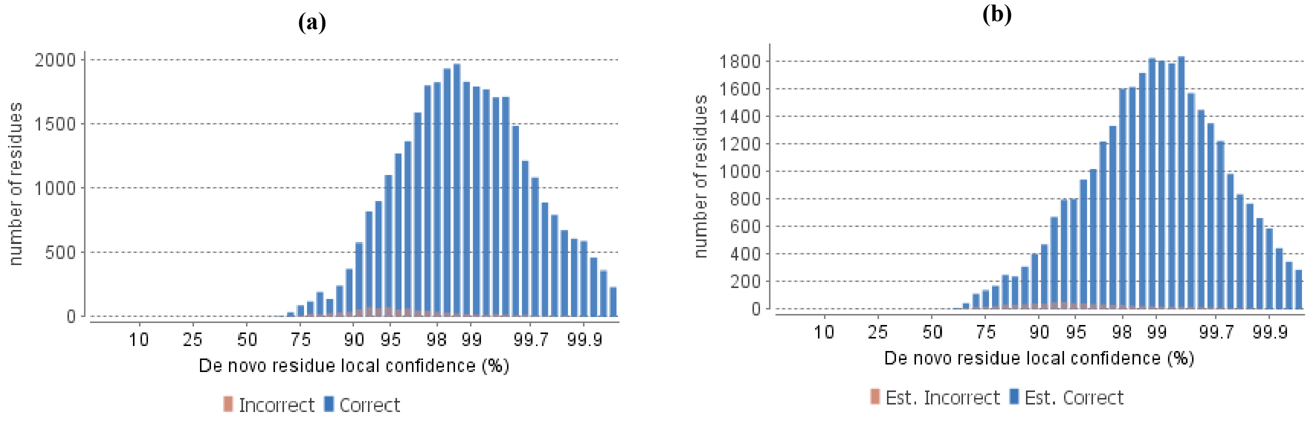

Figure 3. De novo result validation. Distribution of residue local confidence: **(a)** Residues in de novo sequences validated by confident database peptide assignment; **(b)** Residues in "de novo only" sequences.

Table 1. Statistics of data.

# of MS scans	105709
# of MS/MS scans	740752

Table 4. PTM profile.

Name	ΔMass	Position	#PSM	-10lgP	Area	ASco
Carbamidomethyl	57.02	C	5146	151.78	6.65E7	1000.

Oxidation	15.99	M	1571	131.73	5.65E6	1000.
Acetylation	42.01	Protein N-term	339	138.04		1000.
Pyro-glu from Q	-17.03	N-term	197	118.09	1.68E6	1000.
Phosphorylation	79.97	STY	110	79.99	2.61E5	22.

Table 2. Result filtration parameters.

Peptide -10lgP	≥32
Peptide Ascore	≥13
Protein -10lgP	≥55
Proteins unique peptides	≥1
De novo ALC Score	≥95%

Table 3. Statistics of filtered result.

Peptide-Spectrum Matches	22710
Peptide sequences	4484
Protein groups	770
Proteins	1260
Proteins (#Unique Peptides)	496 (>2); 294 (=2); 441 (=1);
FDR (Peptide-Spectrum Matches)	0.9%
FDR (Peptide Sequences)	2.2%
FDR (Protein)	2.4%
De Novo Only Spectra	3125

3. Experiment Control

Figure 4. Precursor mass error of peptide-spectrum matches (PSM) in filtered result. **(a)** Distribution of precursor mass error in ppm; **(b)** Scatterplot of precursor m/z versus precursor mass error in ppm.

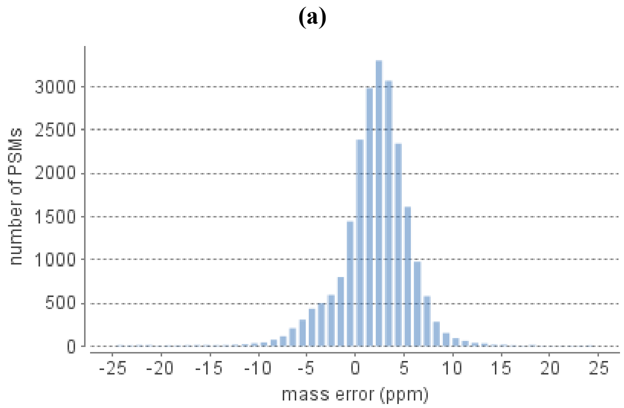

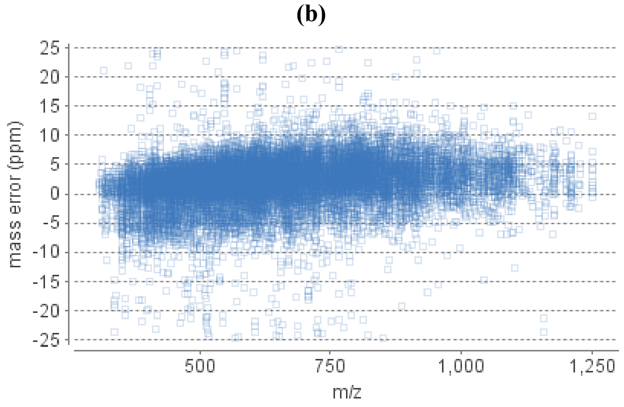

Table 5. Number of identified peptides in each sample by the number of missed cleavages

Missed Cleavages	0	1	2	3	4+
YFP1_1	385	66	7	0	0
YFP1_2	437	85	7	0	0
YFP2_1	381	74	5	0	0
YFP2_2	98	30	3	0	0
YFP3_1	1055	174	16	0	0
YFP3_2	903	183	12	0	0
Tau1_1	100	29	2	0	0
Tau1_2	215	36	1	0	0
Tau2_1	32	10	2	0	0
Tau2_2	45	12	0	0	0
...					

4. Other Information

Table 6. Search parameters.

Search Engine Name: PEAKS
Parent Mass Error Tolerance: 25.0 ppm

Table 7. Instrument parameters.

Fractions: CL_20180217_Exosomes_Y1_1.raw, CL_20180217_Exosomes_Y1_2.raw, CL_20180217_Exosomes_Y2_1.raw, CL_20180217_Exosomes

Fragment Mass Error Tolerance: 0.01 Da
Precursor Mass Search Type: monoisotopic
Enzyme: Trypsin
Max Missed Cleavages: 2
Non-specific Cleavage: none
Fixed Modifications:
 Carbamidomethylation: 57.02
Variable Modifications:
 Oxidation (M): 15.99
 Acetylation (Protein N-term): 42.01
 Pyro-glu from Q: -17.03
 Phosphorylation (STY): 79.97
Max Variable PTM Per Peptide: 3
Database: Hsapiens_MutantTau_20180706
Taxon: All
Searched Entry: 71783
FDR Estimation: Enabled
Different data refine parameters are used for this search:

_Y2_2.raw, CL_20180217_Exosomes_Y3_1.raw, CL_20180217_Exosomes_Y3_2.raw, CL_20180217_Exosomes_T1_1.raw, CL_20180217_Exosomes_T1_2.raw, CL_20180217_Exosomes_T2_1.raw, CL_20180217_Exosomes_T2_2.raw, CL_20180217_Exosomes_T3_1.raw, CL_20180217_Exosomes_T3_2.raw
Ion Source: ESI(nano-spray)
Fragmentation Mode: high energy CID (y and b ions)
MS Scan Mode: FT-ICR/Orbitrap
MS/MS Scan Mode: FT-ICR/Orbitrap
