# Supplementary material for: Dysregulation of Exosome Cargo by Mutant Tau Expressed in Human-induced Pluripotent Stem Cell (iPSC) Neurons Revealed by Proteomics Analyses
Source: Mol Cell Proteomics. 2020 Apr 15;19(6):1017–34. doi: 10.1074/mcp.RA120.002079 (PMC7261814; doi:10.1074/mcp.RA120.002079)

Supplement 5: Annotated spectra for proteins identified by a single peptide

Supplement 6: Annotated spectra of single peptide IDs

Protein Group	Protein ID	Protein Accession	Peptide	Unique	-10lgP	Mass	Length	ppm	m/z	z	RT	Fraction	#Spec	#Spec YFP1_1	#Spec YFP1_2	#Spec YFP2_1	#Spec YFP2_2	#Spec YFP3_1	#Spec YFP3_2	#Spec Tau1_1	#Spec Tau1_2	#Spec Tau2_1	#Spec Tau2_2	#Spec Tau3_1	#Spec Tau3_2	Start	End	PTM
886	1423	sp|O43157|PLXB1_HUMAN	R.RPTVEQGLGQLSNLLNSK.L	Y	92.9	1953.064	18	1.7	652.0298	3	78.91	6	3	0	1	0	0	1	1	0	0	0	0	0	0	1596	1613	
917	1463	sp|Q9HCM3|K1549_HUMAN	K.DDILIIHEPAPLPGPLK.D	Y	81.2	1837.035	17	7.1	613.3566	3	94.38	2	1	0	1	0	0	0	0	0	0	0	0	0	0	1366	1382	
559	1414	sp|P43003|EAA1_HUMAN	R.TTTNVLGDSLGAGIVEHLSR.H	Y	85.94	2039.065	20	0.2	680.6956	3	82.43	6	11	2	1	1	1	2	1	1	1	1	0	0	0	480	499	
671	1547	sp|O00391|QSOX1_HUMAN	K.AHFSPSNIILDFPAAGSAAR.R	Y	85.43	2041.038	20	0.8	681.3538	3	83.09	8	8	0	0	1	1	1	1	1	1	1	1	0	0	533	552	
1216	1528	B1AK88|B1AK88_HUMAN	K.DETVSDC(+57.02)SPHIANIGR.L	Y	80.44	1769.8	16	4.4	590.9433	3	33.54	5	1	0	0	0	0	1	0	0	0	0	0	0	0	229	244	Carbamidomethylation
762	1379	sp|Q07866|KLC1_HUMAN	K.DAANLLNDALAIR.E	Y	80.91	1368.736	13	7.5	685.3804	2	92.05	5	5	0	1	0	0	1	1	1	0	1	0	0	0	273	285	
1103	1643	sp|Q14517|FAT1_HUMAN	K.HFVIDSATGIITLSNLHR.H	Y	83.51	1993.075	18	2.8	665.3673	3	82.66	2	3	1	1	0	0	1	0	0	0	0	0	0	0	2437	2454	
681	1488	sp|Q99460|PSMD1_HUMAN	R.TPEQC(+57.02)PSVVSLLSESYNPHVR.Y	Y	79.6	2398.159	21	1.4	800.3946	3	78	2	6	0	1	0	0	1	1	1	1	1	0	0	0	629	649	Carbamidomethylation
1153	1681	sp|Q15393|SF3B3_HUMAN	K.TPVEEVPAAIAPFQGR.V	Y	87.31	1680.883	16	5.7	841.4538	2	73.44	5	4	1	1	0	0	1	1	0	0	0	0	0	0	943	958	
742	1480	sp|Q9HAR2|AGRL3_HUMAN	K.VYLADPVVFTVK.H	Y	79.16	1349.759	12	1.4	675.8879	2	84.7	5	1	0	0	0	0	1	0	0	0	0	0	0	0	781	792	
648	3053	sp|P24534|EF1B_HUMAN	K.SPAGLQVLNDYLADK.S	Y	100.94	1602.825	15	2.1	802.4216	2	82.42	5	11	1	1	1	1	1	1	1	1	1	1	1	0	8	22	
2887	4386	sp|P47914|RL29_HUMAN	K.AQAAAPASVPAQAPK.R	Y	100.79	1376.741	15	0.2	689.378	2	22.46	5	2	0	0	0	0	1	1	0	0	0	0	0	0	135	149	
712	1987	sp|P00441|SODC_HUMAN	K.GLTEGLHGFHVHEFGDNTAGC(+57.02)TSAGPHFNPLSR.K	Y	91.56	3518.617	33	5.2	704.7344	5	66.1	7	8	1	1	0	1	1	1	1	1	0	0	1	0	38	70	Carbamidomethylation
2040	1866	sp|Q9H7P6|MB12B_HUMAN	R.NHDSSQPTTPSQSSAASTPAPNLPR.H	Y	89.98	2547.195	25	3.5	850.0752	3	31.25	5	2	1	0	0	0	1	0	0	0	0	0	0	0	197	221	
1234	1486	sp|P09486|SPRC_HUMAN	K.LHLDYIGPC(+57.02)K.Y	Y	76.23	1214.612	10	2.6	608.3147	2	38.01	2	1	0	1	0	0	0	0	0	0	0	0	0	0	141	150	Carbamidomethylation
900	1493	sp|O60568|PLOD3_HUMAN	K.LVGPEEALSPGEAR.D	Y	73.12	1423.731	14	2.4	712.8743	2	41.81	3	5	1	1	1	0	1	0	0	0	0	1	0	0	359	372	
675	1525	sp|Q01955|CO4A3_HUMAN	R.ASPFLEC(+57.02)HGR.G	Y	71.18	1172.54	10	3.2	587.279	2	19.5	5	7	1	0	0	0	1	1	1	1	0	1	0	1	1610	1619	Carbamidomethylation
1087	2932	H0YH88|H0YH88_HUMAN	K.YAVLYQPLFDK.R	Y	80	1355.713	11	3	678.8656	2	74.46	8	6	1	0	0	0	1	1	1	1	0	1	0	0	46	56	
1059	1848	sp|Q8WWI5|CTL1_HUMAN	K.LPVPASAPIPFFHR.C	Y	72.42	1547.861	14	-3.1	516.9594	3	90.91	6	4	1	1	0	0	1	1	0	0	0	0	0	0	162	175	
730	1468	sp|P15924|DESP_HUMAN	R.TM(+15.99)IQSPSGVILQEAADVHAR.Y	Y	70.17	2138.079	20	8.9	713.7065	3	66.07	5	1	0	0	0	0	1	0	0	0	0	0	0	0	983	1002	Oxidation (M)
1159	1810	sp|P20073|ANXA7_HUMAN	K.GFGTDEQAIVDVVANR.S	Y	83.26	1689.832	16	2.2	845.9252	2	76.35	5	3	1	0	1	0	1	0	0	0	0	0	0	0	200	215	
972	1494	sp|P12270|TPR_HUMAN	R.ASTALSNEQQAR.R	Y	69.73	1274.622	12	1.4	638.3189	2	8.9	5	1	0	0	0	0	1	0	0	0	0	0	0	0	1054	1065	
2068	2046	sp|Q96A00|PP14A_HUMAN	R.GPGGSPGGLQK.R	Y	92.52	953.493	11	1.9	477.7547	2	9.34	3	3	0	0	1	1	1	0	0	0	0	0	0	0	22	32	
1457	2162	sp|P0DP01|HV108_HUMAN	R.SEDTAVYYC(+57.02)AR	Y	82.05	1333.561	11	1.2	667.7885	2	19.35	5	4	0	0	1	1	1	1	0	0	0	0	0	0	107	117	Carbamidomethylation
744	1670	sp|Q00839|HNRPU_HUMAN	K.SSGPTSLFAVTVAPPGAR.Q	Y	75.12	1713.905	18	1.3	857.9608	2	83.51	6	4	1	1	0	0	1	1	0	0	0	0	0	0	187	204	
1013	1615	sp|P31948|STIP1_HUMAN	R.LAYINPDLALEEK.N	Y	76.42	1487.787	13	2.1	744.9024	2	72.79	6	5	0	1	1	0	1	1	1	0	0	0	0	0	352	364	
2671	1881	sp|Q9ULV4|COR1C_HUMAN	K.TTDTASVQNEAK.L	Y	81.96	1263.594	12	1.4	632.8053	2	7.78	6	1	0	0	0	0	0	1	0	0	0	0	0	0	429	440	
1762	2837	A8MRB1|A8MRB1_HUMAN	K.AMVALIDVFHQYSGR.E	Y	81.87	1705.861	15	2.9	569.6293	3	92.64	6	3	0	0	0	1	1	1	0	0	0	0	0	0	7	21	
388	3835	S4R460|S4R460_HUMAN	EVQLVESGGGLVQPGGSLR.L	Y	89.56	1880.996	19	2.9	941.5078	2	66.03	5	20	2	2	2	1	1	0	2	2	2	2	2	2	1	19	
2811	1771	sp|P12956|XRCC6_HUMAN	R.DSLIFLVDASK.A	Y	75.45	1206.65	11	3.3	604.334	2	85.6	5	1	0	0	0	0	1	0	0	0	0	0	0	0	36	46	
1755	3393	sp|P01591|IGJ_HUMAN	R.FVYHLSDLC(+57.02)K.K	Y	78.96	1280.622	10	1.7	641.3195	2	41.72	3	2	0	0	1	0	0	1	0	0	0	0	0	0	83	92	Carbamidomethylation
1236	3776	sp|A0A0C4DH31|HV118_HUMAN	R.SDDTAVYYC(+57.02)AR	Y	79.98	1319.545	11	1	660.7805	2	19.13	3	4	0	0	1	1	1	0	0	0	0	0	1	0	107	117	Carbamidomethylation
787	3775	sp|P05204|HMGN2_HUMAN	R.LSAKPAPPKPEPKPK.K	Y	87.21	1583.94	15	2.9	528.9888	3	11.15	5	9	2	0	0	1	2	2	1	1	0	0	0	0	28	42	
958	2325	sp|P01594|KV133_HUMAN	K.LLIYDASNLETGVPSR.F	Y	77.71	1746.915	16	2.8	874.4673	2	73.35	3	7	0	0	1	1	0	0	1	1	0	1	1	1	68	83	
1246	1529	sp|P08621|RU17_HUMAN	R.DPIPYLPPLEK.L	Y	59.41	1280.702	11	3.7	641.3604	2	83.23	5	2	1	0	0	0	1	0	0	0	0	0	0	0	17	27	
1191	1394	sp|Q8TF72|SHRM3_HUMAN	R.SSPATADKR.Q	Y	58.51	931.4723	9	1.5	466.7441	2	9.18	5	3	1	0	1	0	1	0	0	0	0	0	0	0	1242	1250	
2920	2740	sp|P98172|EFNB1_HUMAN	K.GGSGTAGTEPSDIIIPLR.T	Y	85.83	1739.905	18	1.6	870.9613	2	73.94	5	2	0	0	0	0	1	1	0	0	0	0	0	0	290	307	
1871	1787	sp|Q12882|DPYD_HUMAN	K.QEYVGGLSTSEIPQFR.L	Y	75.22	1809.89	16	9.9	905.9611	2	72.73	6	1	0	0	0	0	0	1	0	0	0	0	0	0	220	235	
2125	2298	sp|Q6FHJ7|SFRP4_HUMAN	R.SGC(+57.02)NEVTTVVDVK.E	Y	85.34	1406.671	13	5	704.3464	2	35.73	6	3	1	0	0	0	1	1	0	0	0	0	0	0	209	221	Carbamidomethylation
909	2432	sp|Q15121|PEA15_HUMAN	K.SEEITTGSAWFSFLESHNK.L	Y	76.7	2169.002	19	4.5	724.011	3	92.31	2	6	1	1	1	0	1	1	1	0	0	0	0	0	36	54	
954	2774	sp|Q9UHY7|ENOPH_HUMAN	K.AEFFADVVPAVR.K	Y	84.06	1319.687	12	2.7	660.8527	2	82.48	5	8	1	1	0	0	1	1	1	1	1	1	0	0	129	140	
1448	2434	sp|P61019|RAB2A_HUMAN	K.TASNVEEAFINTAK.E	Y	83.7	1493.736	14	3.6	747.8781	2	45.81	8	4	0	0	0	1	0	1	1	1	0	0	0	0	152	165	
2124	1766	sp|Q8IWA5|CTL2_HUMAN	K.NENKPYLFYFNIVK.C	Y	72.31	1787.925	14	3.5	596.9842	3	85.29	6	2	0	0	0	0	1	1	0	0	0	0	0	0	81	94	
1228	2468	sp|O60701|UGDH_HUMAN	R.VLIGGDETPEGQR.A	Y	83.56	1369.684	13	1.1	685.8499	2	27.22	3	6	0	1	1	0	1	1	0	0	0	1	1	0	178	190	
4574	4665	sp|P80748|LV321_HUMAN	R.FSGSNSGNTATLTISR.V	Y	83.48	1611.785	16	7.3	806.9058	2	32.29	5	1	0	0	0	0	1	0	0	0	0	0	0	0	80	95	
1773	3461	sp|P09497|CLCB_HUMAN	K.VAQLC(+57.02)DFNPK.S	Y	75.2	1190.575	10	4.2	596.2974	2	34.95	6	2	0	0	0	0	1	1	0	0	0	0	0	0	195	204	Carbamidomethylation
1975	2058	sp|Q9H6R3|ACSS3_HUMAN	R.VLAEHGVAALFTAPTAIR.A	Y	75.63	1836.026	18	2.7	613.0175	3	81.42	5	2	0	0	0	1	1	0	0	0	0	0	0	0	381	398	
1378	1435	G3V0E5|G3V0E5_HUMAN	K.VSASPLLYTLIEK.T	Y	61.78	1432.818	13	7.2	717.4213	2	93.89	6	3	0	1	1	0	0	1	0	0	0	0	0	0	415	427	
1249	3501	A0A075B7B8|A0A075B7B8_HUMAN	R.VEDTAVYYC(+57.02)AR	Y	82	1345.597	11	3.7	673.8083	2	23.89	6	6	0	0	1	1	1	1	0	0	0	1	1	0	107	117	Carbamidomethylation
1248	3398	sp|Q9BT88|SYT11_HUMAN	R.NLLVDAAEAGLLSR.D	Y	73.59	1440.794	14	6.6	721.4088	2	89.36	5	5	1	1	1	0	1	1	0	0	0	0	0	0	93	106	
858	2973	sp|P53999|TCP4_HUMAN	K.GISLNPEQWSQLK.E	Y	68.38	1498.778	13	2.6	750.3982	2	79.46	5	7	0	1	0	0	1	1	1	1	1	1	0	0	102	114	
2186	2072	sp|P22061|PIMT_HUMAN	K.ALDVGSGSGILTAC(+57.02)FAR.M	Y	68.21	1693.846	17	2.3	847.9321	2	79.48	6	2	0	1	0	0	0	1	0	0	0	0	0	0	82	98	Carbamidomethylation
2760	2240	sp|Q14108|SCRB2_HUMAN	K.DEVLYVFPSDFC(+57.02)R.S	Y	68.22	1645.745	13	6.7	823.8851	2	92.05	5	1	0	0	0	0	1	0	0	0	0	0	0	0	263	275	Carbamidomethylation
1725	2013	sp|Q00688|FKBP3_HUMAN	K.SEETLDEGPPK.Y	Y	65.99	1200.551	11	-6.5	601.2789	2	13.95	8	1	0	0	0	0	0	0	0	1	0	0	0	0	100	110	
2042	2090	sp|Q9H9H4|VP37B_HUMAN	R.SLAEGNLLYQPQLDTLK.A	Y	79.23	1902.01	17	1.8	952.0139	2	82.59	6	3	1	1	0	0	0	1	0	0	0	0	0	0	54	70	
2881	3612	sp|P62266|RS23_HUMAN	K.ANPFGGASHAK.G	Y	78.98	1055.515	11	2.5	528.766	2	11.01	6	2	0	0	0	0	1	1	0	0	0	0	0	0	38	48	
1473	3855	sp|Q9Y3E1|HDGR3_HUMAN	K.GFNEGLWEIENNPGVK.F	Y	78.64	1801.864	16	4.6	901.9432	2	73.47	5	5	1	0	0	0	1	1	1	1	0	0	0	0	80	95	
386	2409	sp|P17096|HMGA1_HUMAN	R.KQPPVSPGTALVGSQKEPSEVPTPK.R	Y	77.88	2557.375	25	4.9	640.3542	4	41.3	6	20	2	2	2	2	1	2	1	2	2	2	1	1	31	55	
1714	3140	sp|P02792|FRIL_HUMAN	K.LNQALLDLHALGSAR.T	Y	77.76	1590.884	15	2.3	531.3032	3	69.39	5	4	0	0	0	0	1	0	0	1	1	1	0	0	107	121	
1390	1591	sp|P06576|ATPB_HUMAN	K.VLDSGAPIKIPVGPETLGR.I	Y	64.76	1918.089	19	3	640.3721	3	78.88	2	4	1	1	0	0	1	1	0	0	0	0	0	0	125	143	
1534	1729	sp|O75475|PSIP1_HUMAN	R.VDEVPDGAVKPPTNK.L	Y	61.46	1564.81	15	4.2	522.6127	3	25.93	1	1	1	0	0	0	0	0	0	0	0	0	0	0	25	39	
443	3620	sp|P09382|LEG1_HUMAN	M.A(+42.01)C(+57.02)GLVASNLNLKPGEC(+57.02)LR.V	Y	77.21	2013.014	18	3.3	672.014	3	78.49	6	15	2	1	0	0	2	1	1	2	2	2	1	1	2	19	Acetylation (Protein N-term); Carbamidomethylation
1435	3446	sp|P54578|UBP14_HUMAN	K.VSIVTPEDILR.L	Y	68.65	1240.703	11	5.4	621.3619	2	81.17	5	4	1	1	0	0	1	1	0	0	0	0	0	0	455	465	
1039	1927	sp|O75110|ATP9A_HUMAN	R.LPTAADLLQIR.S	Y	66.4	1209.708	11	2.1	605.8626	2	84.64	6	6	1	1	1	1	1	1	0	0	0	0	0	0	210	220	
3356	3555	sp|P41732|TSN7_HUMAN	R.TYTDAMQTYNGNDER.S	Y	76.12	1777.721	15	2.7	889.8704	2	21.27	5	1	0	0	0	0	1	0	0	0	0	0	0	0	123	137	
2814	2404	sp|Q9H0C2|ADT4_HUMAN	R.YFPTQALNFAFK.D	Y	75.91	1445.734	12	5.4	723.8783	2	94.58	6	2	0	1	0	0	0	1	0	0	0	0	0	0	93	104	
1756	3698	sp|Q6EEV6|SUMO4_HUMAN	K.VAGQDGSVVQFK.I	Y	74.76	1233.635	12	5.7	617.8284	2	30.38	5	4	1	1	0	0	1	1	0	0	0	0	0	0	22	33	
1765	4153	sp|P04278|SHBG_HUMAN	K.QAEISASAPTSLR.S	Y	74.47	1329.689	13	3.2	665.8538	2	33.12	5	4	0	0	1	1	1	1	0	0	0	0	0	0	203	215	
2171	3229	sp|Q9NRX4|PHP14_HUMAN	K.QGC(+57.02)DC(+57.02)EC(+57.02)LGGGR.I	Y	74	1367.502	12	2.1	684.7596	2	9.17	5	3	1	0	0	0	1	1	0	0	0	0	0	0	67	78	Carbamidomethylation
2989	4768	sp|P01766|HV313_HUMAN	R.AGDTAVYYC(+57.02)AR	Y	73.95	1245.545	11	6.6	623.7838	2	18.86	3	2	0	0	1	0	0	1	0	0	0	0	0	0	106	116	Carbamidomethylation
699	1691	J3KTF8|J3KTF8_HUMAN	R.AEEYEFLTPVEEAPK.G	Y	56.6	1750.83	15	-3	876.4197	2	69.76	2	6	1	2	0	0	0	1	1	1	0	0	0	0	153	167	
1727	2343	H3BN14|H3BN14_HUMAN	R.LLPVQENFLLK.F	Y	65.74	1312.775	11	1.9	657.3962	2	85.37	6	3	0	0	0	0	1	1	0	1	0	0	0	0	179	189	
1609	1649	sp|Q9Y4F1|FARP1_HUMAN	K.DLIGIDNLVVPGR.E	Y	55.97	1379.777	13	1.8	690.8971	2	94.66	7	1	0	0	0	0	0	0	1	0	0	0	0	0	746	758	

Protein Group	Protein ID	Protein Accession	Peptide	Unique	-10lgP	Mass	Length	ppm	m/z	z	RT	Fraction	#Spec	#Spec YFP1_1	#Spec YFP1_2	#Spec YFP2_1	#Spec YFP2_2	#Spec YFP3_1	#Spec YFP3_2	#Spec Tau1_1	#Spec Tau1_2	#Spec Tau2_1	#Spec Tau2_2	#Spec Tau3_1	#Spec Tau3_2	Start	End	PTM
1704	2503	sp|O00231|PSD11_HUMAN	K.LYDNLLEQNLIR.V	Y	63.59	1502.809	12	4	752.4149	2	81.4	2	3	1	1	0	0	0	0	0	0	1	0	0	0	326	337	
2955	3535	A0A0C4DH35|A0A0C4DH35_HUMAN	R.AEDTAVYYC(+57.02)VR	Y	73.02	1345.597	11	5.4	673.8095	2	30.08	4	2	0	0	0	1	0	1	0	0	0	0	0	0	107	117	Carbamidomethylation
460	1910	sp|Q8IUG5|MY18B_HUMAN	K.QMVLHEK.Q	Y	53.97	883.4586	7	-4.2	442.7347	2	34.44	8	9	1	1	1	1	0	0	1	1	1	1	1	0	1769	1775	
2041	1999	sp|Q9NRX5|SERC1_HUMAN	K.LTLTSDESTLIEDGGAR.S	Y	72.62	1776.874	17	3.8	889.4477	2	58.9	2	3	0	1	0	0	0	0	0	1	1	0	0	0	344	360	
2149	2127	Q5T8U3|Q5T8U3_HUMAN	R.LKVPPAINQFTQALDR.Q	Y	61.92	1810.01	16	2.2	604.3453	3	83.62	2	1	0	1	0	0	0	0	0	0	0	0	0	0	101	116	
220	1409	sp|Q6W2J9|BCOR_HUMAN	R.LSNGKYPK.A	Y	46.49	905.497	8	-5.2	453.7534	2	18.67	11	6	1	1	1	0	1	1	0	0	0	0	1	0	388	395	
1766	4288	sp|O75340|PDCD6_HUMAN	K.AGVNFSEFTGVWK.Y	Y	71.3	1440.704	13	0.9	721.3598	2	85.28	3	4	0	1	1	0	1	1	0	0	0	0	0	0	78	90	
3682	2084	sp|P16278|BGAL_HUMAN	K.TVGAALDILC(+57.02)PSGPIK.S	Y	71.13	1610.87	16	6.6	806.4476	2	85.25	6	1	0	0	0	0	0	1	0	0	0	0	0	0	384	399	Carbamidomethylation
2126	2350	sp|A0A0A0MS15|HV349_HUMAN	K.GLEWVGFIR.S	Y	71.04	1075.581	9	1.3	538.7987	2	85.54	3	3	0	0	1	1	1	0	0	0	0	0	0	0	63	71	
2201	1997	sp|P21926|CD9_HUMAN	K.KDVLETFTVK.S	Y	57.57	1178.655	10	4	590.337	2	42.5	2	2	0	1	0	0	1	0	0	0	0	0	0	0	170	179	
392	1582	sp|Q5VST9|OBSCN_HUMAN	R.IEAAGC(+57.02)MR.Q	Y	32.25	906.4052	8	7.4	454.2132	2	6.13	6	1	0	0	0	0	0	1	0	0	0	0	0	0	1306	1313	Carbamidomethylation
4445	4439	sp|P67870|CSK2B_HUMAN	K.YQQGDFGYC(+57.02)PR.V	Y	70.29	1389.577	11	7.5	695.8011	2	25.98	5	1	0	0	0	0	1	0	0	0	0	0	0	0	101	111	Carbamidomethylation
2821	3515	sp|P35325|SPR2B_HUMAN	K.C(+57.02)PEPC(+57.02)PPPK.C	Y	70.08	1080.473	9	1.1	541.2444	2	10.75	6	2	0	0	0	0	1	1	0	0	0	0	0	0	23	31	Carbamidomethylation
2457	1745	A0A087WZE9|A0A087WZE9_HUMAN	R.LSAKPAPPKPEPKPR.K	Y	58.98	1611.946	15	0.8	538.323	3	11.53	5	1	0	0	0	0	1	0	0	0	0	0	0	0	30	44	
2170	3043	sp|P43007|SATT_HUMAN	K.ETVDSFLDLAR.N	Y	69.98	1264.63	11	9.2	633.3281	2	76.14	8	3	0	0	0	0	0	0	0	1	1	1	0	0	166	176	
4302	4755	sp|Q9UBP4|DKK3_HUMAN	R.SAVEEMEAEEAAAK.A	Y	69.91	1463.645	14	6.4	732.8344	2	28.96	2	1	0	1	0	0	0	0	0	0	0	0	0	0	67	80	
1741	3874	sp|O75348|VATG1_HUMAN	M.A(+42.01)SQSQGIQQLLQAEK.R	Y	69.87	1669.864	15	1.4	835.9402	2	74.42	6	3	1	0	0	0	1	1	0	0	0	0	0	0	2	16	Acetylation (Protein N-term)
2872	2029	sp|Q15599|NHRF2_HUMAN	R.LLVVDPETDEHFK.R	Y	62.1	1540.777	13	1.8	514.6006	3	49.77	1	1	1	0	0	0	0	0	0	0	0	0	0	0	225	237	
698	1576	sp|P32004|L1CAM_HUMAN	R.LVLSDLHLLTQSQVR.V	Y	49.51	1720.984	15	4.9	574.6713	3	80.8	2	4	0	1	0	1	1	1	0	0	0	0	0	0	618	632	
1638	1624	sp|P43146|DCC_HUMAN	R.VVVLPSGALQISR.L	Y	49.5	1337.803	13	6.7	669.9133	2	72.5	6	1	0	0	0	0	0	1	0	0	0	0	0	0	189	201	
2433	2109	sp|P00734|THRB_HUMAN	R.SGIEC(+57.02)QLWR.S	Y	68.59	1147.544	9	2.9	574.7811	2	42.15	2	2	1	1	0	0	0	0	0	0	0	0	0	0	125	133	Carbamidomethylation
2958	4110	sp|Q96P70|IPO9_HUMAN	M.A(+42.01)AAAAAGAASGLPGPVAQGLK.E	Y	68.4	1789.969	21	0.6	597.6638	3	81.6	6	2	1	0	0	0	0	1	0	0	0	0	0	0	2	22	Acetylation (Protein N-term)
1080	2364	sp|P53985|MOT1_HUMAN	K.EEETSIDVAGKPNEVTK.A	Y	60.15	1844.9	17	3.5	615.9762	3	25.93	3	6	1	1	1	1	1	1	0	0	0	0	0	0	463	479	
2483	2020	sp|Q24JP5|T132A_HUMAN	R.SPLSDSILGEQALAVTDDK.V	Y	59.53	1957.984	19	-0.3	979.9992	2	81.43	10	1	0	0	0	0	0	0	0	0	0	1	0	0	615	633	
2039	1824	sp|P62993|GRB2_HUMAN	K.ATADDELSFK.R	Y	53.61	1095.508	10	1	548.762	2	29.26	2	1	0	1	0	0	0	0	0	0	0	0	0	0	11	20	
2642	1964	sp|P00387|NB5R3_HUMAN	R.STPAITLESPDIKYPLR.L	Y	59.86	1900.031	17	2.6	634.3524	3	73.73	5	1	0	0	0	0	1	0	0	0	0	0	0	0	30	46	
2882	4164	sp|P54920|SNAA_HUMAN	K.NSQSFFSGLFGGSSK.I	Y	67.73	1548.721	15	11.6	775.3767	2	84.52	5	2	0	0	0	0	1	1	0	0	0	0	0	0	23	37	
1227	2440	sp|Q9NUI1|DECR2_HUMAN	K.VAFITGGGSGIGFR.I	Y	67.32	1337.709	14	2.5	669.8635	2	69.43	6	6	0	0	1	1	1	1	0	1	0	0	0	1	30	43	
2988	3384	sp|O75822|EIF3J_HUMAN	K.VLTPEEQLADKLR.L	Y	58.41	1510.835	13	1	504.6196	3	49.77	1	1	1	0	0	0	0	0	0	0	0	0	0	0	107	119	
2021	2909	sp|P04792|HSPB1_HUMAN	R.VSLDVNHFAPDELTVK.T	Y	57.29	1782.915	16	1.8	595.3134	3	71.18	8	2	0	0	0	0	0	1	0	1	0	0	0	0	97	112	
2375	1605	sp|P19367|HXK1_HUMAN	K.KLPVGFTFSFPC(+57.02)QQSK.I	Y	51.91	1869.945	16	2.7	624.3239	3	81.67	1	1	1	0	0	0	0	0	0	0	0	0	0	0	147	162	Carbamidomethylation
1112	2443	sp|Q96M86|DNHD1_HUMAN	K.LEDM(+15.99)R.G	Y	32.06	678.3007	5	2.3	340.1584	2	8.37	5	1	0	0	0	0	1	0	0	0	0	0	0	0	732	736	Oxidation (M)
1764	3354	sp|O15240|VGF_HUMAN	K.AYQGVAAPFPK.A	Y	56.67	1147.603	11	4.2	574.811	2	39.08	6	1	0	0	0	0	0	1	0	0	0	0	0	0	268	278	
3894	3101	sp|Q7Z3B1|NEGR1_HUMAN	K.VVVNFAPTIQEIK.S	Y	65.41	1456.829	13	5.9	729.4261	2	77.54	6	1	0	0	0	0	0	1	0	0	0	0	0	0	219	231	
2643	2308	sp|Q96AQ6|PBIP1_HUMAN	K.DSHDPLPSWAELLRPK.Y	Y	65.35	1859.953	16	23.6	621.0062	3	84.16	1	2	1	0	0	0	0	0	1	0	0	0	0	0	566	581	
2203	3388	sp|A0A0C4DH38|HV551_HUMAN	K.KPGESLK.I	Y	49.86	757.4333	7	0.3	379.7241	2	6.11	6	1	0	0	0	0	0	1	0	0	0	0	0	0	32	38	
876	1765	F8W8V9|F8W8V9_HUMAN	R.VS(+79.97)LGNDTRTFYQFEAAWDSSM(+15.99)HNSLLLNR.V	Y	40.17	3467.561	29	-23.6	1156.834	3	93.43	1	2	1	0	0	0	0	0	1	0	0	0	0	0	1337	1365	Phosphorylation (STY); Oxidation (M)
1735	2129	sp|Q13740|CD166_HUMAN	R.SSPSFSSLHYQDAGNYVC(+57.02)ETALQEVEGLKK.R	Y	57.48	3343.567	30	0.4	836.8993	4	88.75	5	2	0	0	0	0	1	0	0	0	1	0	0	0	375	404	Carbamidomethylation
1743	4179	sp|P12273|PIP_HUMAN	R.ELGIC(+57.02)PDDAAVIPIK.N	Y	64.67	1609.839	15	10.1	805.9346	2	84.3	4	4	0	0	1	1	1	1	0	0	0	0	0	0	119	133	Carbamidomethylation
4575	4805	sp|Q9ULC3|RAB23_HUMAN	K.VVAEVGDIPTVLVQNK.I	Y	64.21	1679.946	16	1.4	840.9813	2	79.83	5	1	0	0	0	0	1	0	0	0	0	0	0	0	107	122	
2768	3932	sp|P58546|MTPN_HUMAN	K.GPDGLTAFEATDNQAIK.A	Y	64.17	1746.842	17	5	874.4329	2	57.37	5	2	0	0	0	0	1	0	1	0	0	0	0	0	98	114	
2218	4812	sp|P35237|SPB6_HUMAN	K.IAELLSPGSVDPLTR.L	Y	63.86	1566.862	15	2.1	784.4398	2	80.67	7	3	0	0	0	0	0	0	1	1	0	1	0	0	146	160	
4442	4826	sp|Q9NR31|SAR1A_HUMAN	K.LVFLGLDNAGK.T	Y	63.56	1145.644	11	5.5	573.8326	2	71.65	5	1	0	0	0	0	1	0	0	0	0	0	0	0	28	38	
2187	2119	sp|P36578|RL4_HUMAN	R.IEEVPELPLVVEDK.V	Y	50.09	1607.866	14	2.2	804.9419	2	89.09	5	1	0	0	0	0	1	0	0	0	0	0	0	0	144	157	
3684	2266	sp|O43237|DC1L2_HUMAN	K.TYGFHFTTPALVVEK.D	Y	63.43	1708.882	15	0	570.6348	3	78.5	5	1	0	0	0	0	1	0	0	0	0	0	0	0	291	305	
2759	2210	sp|P15586|GNS_HUMAN	K.IQEPNTFPAILR.S	Y	63.18	1397.767	12	2.8	699.8925	2	71.69	8	2	0	0	0	0	0	0	1	1	0	0	0	0	126	137	
3778	3183	sp|P25705|ATPA_HUMAN	R.VVDALGNAIDGK.G	Y	62.39	1170.624	12	2.3	586.3208	2	38.86	5	1	0	0	0	0	1	0	0	0	0	0	0	0	150	161	
2762	2770	sp|P16298|PP2BB_HUMAN	K.LFEVGGSPANTR.Y	Y	62.09	1246.631	12	3.1	624.3245	2	34.97	6	2	0	0	0	1	0	1	0	0	0	0	0	0	110	121	
2155	3807	sp|P01782|HV309_HUMAN	R.AEDTALYYC(+57.02)AK.D	Y	61.39	1303.575	11	-2.7	652.7932	2	24.01	4	2	0	0	1	1	0	0	0	0	0	0	0	0	107	117	Carbamidomethylation
2572	1981	sp|Q14697|GANAB_HUMAN	R.YRVPDVLVADPPIAR.L	Y	53.65	1679.936	15	3.1	560.9876	3	72.93	6	1	0	0	0	0	0	1	0	0	0	0	0	0	113	127	
2957	3860	sp|P04424|ARLY_HUMAN	R.SRNDQVVTDLR.L	Y	61.04	1301.669	11	3.7	651.8441	2	19.01	5	2	0	0	0	1	1	0	0	0	0	0	0	0	112	122	
2991	4861	sp|Q9UM00|TMCO1_HUMAN	K.KETITESAGR.Q	Y	60.71	1090.562	10	2	546.2892	2	7.22	6	2	0	0	0	1	0	1	0	0	0	0	0	0	54	63	
462	1696	sp|Q92616|GCN1_HUMAN	R.TLGDLVR.K	Y	43.38	772.4443	7	0.5	387.2296	2	31.51	6	11	1	1	1	1	1	1	1	1	1	1	1	0	1948	1954	
2571	1662	sp|O94985|CSTN1_HUMAN	K.VIDC(+57.02)LYTC(+57.02)K.E	Y	45.46	1170.541	9	6	586.2814	2	30.29	4	1	0	0	0	1	0	0	0	0	0	0	0	0	554	562	Carbamidomethylation
2990	4848	sp|P62304|RUXE_HUMAN	K.VM(+15.99)VQPINLIFR.Y	Y	60.11	1344.759	11	4	673.3893	2	92.72	6	2	0	0	0	1	0	1	0	0	0	0	0	0	13	23	Oxidation (M)
1450	2841	sp|P10155|RO60_HUMAN	K.LGLENAEALIR.L	Y	59.86	1197.672	11	3.3	599.8451	2	72.54	2	5	0	1	0	0	0	0	1	1	1	1	0	0	53	63	
1728	3897	sp|P62314|SMD1_HUMAN	K.NREPVQLETLSIR.G	Y	59.82	1553.853	13	-2	518.9571	3	47.93	6	4	0	1	0	0	1	1	0	1	0	0	0	0	49	61	
2202	2260	sp|O75347|TBCA_HUMAN	R.RLEAAYLDLQR.I	Y	46.32	1346.731	11	13.7	449.9236	3	41.64	2	1	0	1	0	0	0	0	0	0	0	0	0	0	70	80	
4576	4853	E9PJD9|E9PJD9_HUMAN	K.TGAAPIIDVVR.S	Y	59.65	1110.64	11	0	556.3271	2	53.29	8	1	0	0	0	0	0	0	0	1	0	0	0	0	38	48	
1415	2315	sp|Q8TD26|CHD6_HUMAN	R.EQADFYR.T	Y	40.74	927.4086	7	-2	464.7107	2	12.19	3	1	0	0	1	0	0	0	0	0	0	0	0	0	1454	1460	
1258	4896	sp|P10114|RAP2A_HUMAN	K.VPVILVGNK.V	Y	59.03	937.596	9	-0.3	469.8051	2	44.24	2	6	1	1	1	0	1	1	1	0	0	0	0	0	109	117	
2992	4868	sp|Q13822|ENPP2_HUMAN	K.AGTFFWSVVIPHER.R	Y	58.95	1644.841	14	5.5	549.2907	3	92.7	6	2	0	0	0	1	0	1	0	0	0	0	0	0	271	284	
3590	2339	sp|Q9P2W7|B3GA1_HUMAN	R.ELVTLNDLEPK.A	Y	58.9	1269.682	11	7.5	635.8528	2	67.94	3	1	0	0	1	0	0	0	0	0	0	0	0	0	290	300	
2396	1732	sp|Q9UHD8|SEPT9_HUMAN	K.STLINTLFK.S	Y	50.57	1035.596	9	-1.8	518.8046	2	71.97	8	1	0	0	0	0	0	0	0	1	0	0	0	0	312	320	
4439	3626	sp|Q9UHB9|SRP68_HUMAN	R.FETFC(+57.02)LDPSLVTK.Q	Y	58.47	1555.759	13	7.4	778.8926	2	85.32	5	1	0	0	0	0	1	0	0	0	0	0	0	0	558	570	Carbamidomethylation
1487	4891	sp|P31949|S10AB_HUMAN	K.ISSPTETER.C	Y	58.35	1018.493	9	7.3	510.2576	2	9.8	6	5	1	0	0	0	1	1	1	1	0	0	0	0	4	12	
3591	2476	sp|O14910|LIN7A_HUMAN	K.ATVAAFAASEGHSHPR.V	Y	57.95	1607.78	16	-7.6	536.93	3	17.85	8	1	0	0	0	0	0	0	0	1	0	0	0	0	92	107	
3683	2249	sp|Q99829|CPNE1_HUMAN	R.LYGPTNFAPIINHVAR.F	Y	57.67	1781.958	16	7.4	594.9976	3	77.44	5	1	0	0	0	0	1	0	0	0	0	0	0	0	385	400	
2103	1692	sp|P23471|PTPRZ_HUMAN	K.AIIDGVESVSR.F	Y	42.64	1144.609	11	4.9	573.3145	2	38.38	1	1	1	0	0	0	0	0	0	0	0	0	0	0	195	205	
2876	2539	sp|P26022|PTX3_HUMAN	R.ALAAVLEELR.Q	Y	57.55	1083.629	10	2.3	542.8229	2	75.12	6	2	1	0	0	0	0	1	0	0	0	0	0	0	148	157	
2921	2892	sp|Q92832|NELL1_HUMAN	K.AFLFQDIER.E	Y	57.3	1137.582	9	2	569.7993	2	68.61	9	2	0	0	0	0	0	0	0	0	1	1	0	0	57	65	
2395	1672	sp|Q7L576|CYFP1_HUMAN	R.LGTPQQIAIAR.E	Y	45.29	1166.677	11	-0.1	584.3458	2	39.04	5	1	0	0	0	0	1	0	0	0	0	0	0	0	1066	1076	
2879	3091	sp|O75781|PALM_HUMAN	R.LLEDSVSR.L	Y	56.74	917.4818	8	1.6	459.7489	2	18.73	6	2	0	0	0	0	1	1	0	0	0	0	0	0	83	90	
2204	3872	sp|Q15293|RCN1_HUMAN	K.RYIFDNVAK.V	Y	56.69	1124.598	9	0	563.3062	2	30.59	6	2	1	0	0	0	0	1	0	0	0	0	0	0	114	122	
1754	2828	sp|A0A0J9YXX1|HV5X1_HUMAN	K.KPGESLR.I	Y	47.38	785.4395	7	1.2	393.7275	2	6.26	3	3	0	0	1	1	0	1	0	0	0	0	0	0	32	38	
2216	3035	sp|P61970|NTF2_HUMAN	K.LSSLPFQK.I	Y	42.64	918.5175	8	1.4	460.2667	2	38.87	1	1	1	0	0	0	0	0	0	0	0	0	0	0	56	63	
1021	1878	F1T0I1|F1T0I1_HUMAN	K.APGDLPAAGGPPSGAMPFYNPAQLAQAC(+57.02)AT(+79.97)S(+79.97)GSSR.L	Y	37.05	3531.499	35	-19.2	883.8651	4	82.22	7	1	0	0	0	0	0	0	1	0	0	0	0	0	2287	2321	Carbamidomethylation; Phosphorylation (STY)
3506	2507	sp|Q92945|FUBP2_HUMAN	R.IGGGIDVPVPR.H	Y	55.69	1078.613	11	6.2	540.3173	2	53.12	5	1	0	0	0	0	1	0	0	0	0	0	0	0	321	331	
4440	3640	sp|O75973|C1QRF_HUMAN	R.TDGGDALSEQSGAPPPSTLVQGPQGKPGR.T	Y	55.68	2803.374	29	8.7	935.4732	3	43.9	6	1	0	0	0	0	0	1	0	0	0	0	0	0	46	74	
956	3919	sp|Q6VVX0|CP2R1_HUMAN	R.YFGYGQK.S	Y	55.39	861.4021	7	1.5	431.709	2	18.71	3	5	1	0	1	1	1	1	0	0	0	0	0	0	146	152	
2418	1947	sp|Q8TE60|ATS18_HUMAN	R.TAEEKIQR.Y	Y	46.79	973.5192	8	0.6	487.7672	2	6.19	3	1	0	0	1	0	0	0	0	0	0	0	0	0	206	213	
2526	2100	sp|P12259|FA5_HUMAN	K.LSEGASYLDHTFPAEK.M	Y	47.57	1763.837	16	3.3	588.9547	3	44.12	3	1	0	0	1	0	0	0	0	0	0	0	0	0	122	137	
2105	2242	sp|Q9HCJ6|VAT1L_HUMAN	R.AVVLAGFGGLNK.L	Y	46.83	1144.66	12	1.7	573.3384	2	64.84	5	2	0	0	0	1	1	0	0	0	0	0	0	0	44	55	

sp|O43157|PLXB1_HUMAN

R.RPTVEQGLGQLSNLLNSK.L

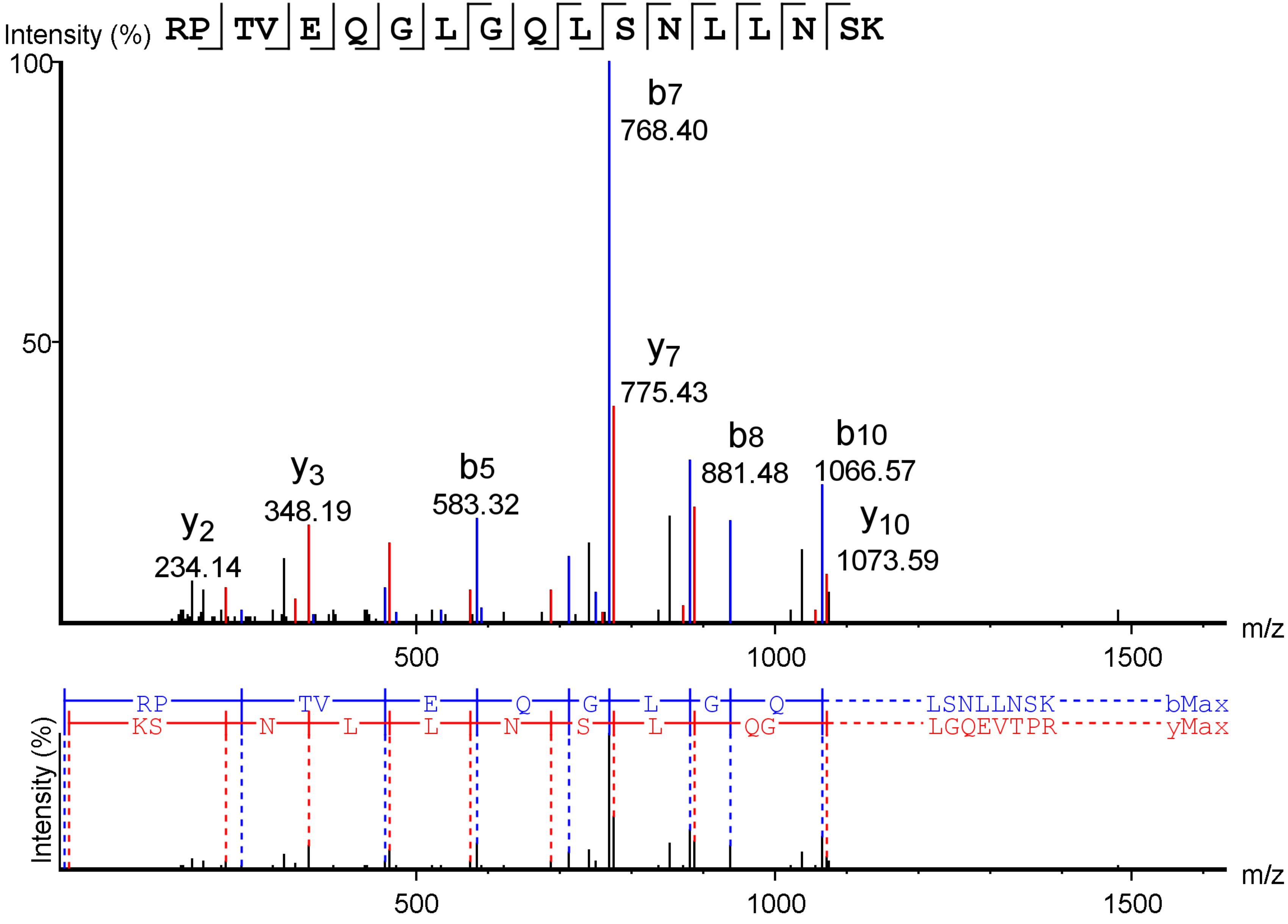

sp|Q9HCM3|K1549_HUMAN

K.DDILIIHEPAPLPGPLK.D

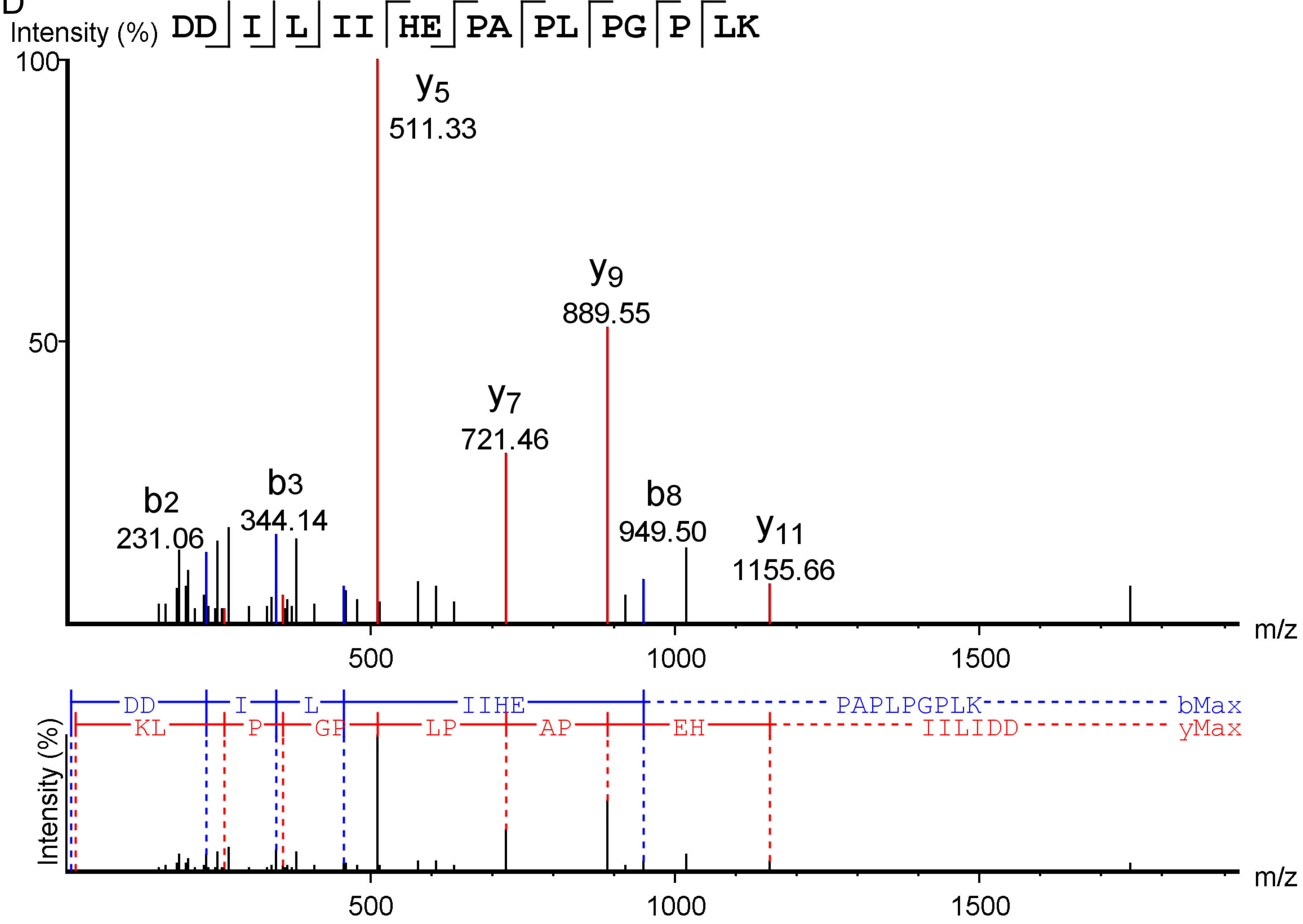

sp|P43003|EAA1_HUMAN

R.TTTNVLGDSLGAGIVEHLSR.H

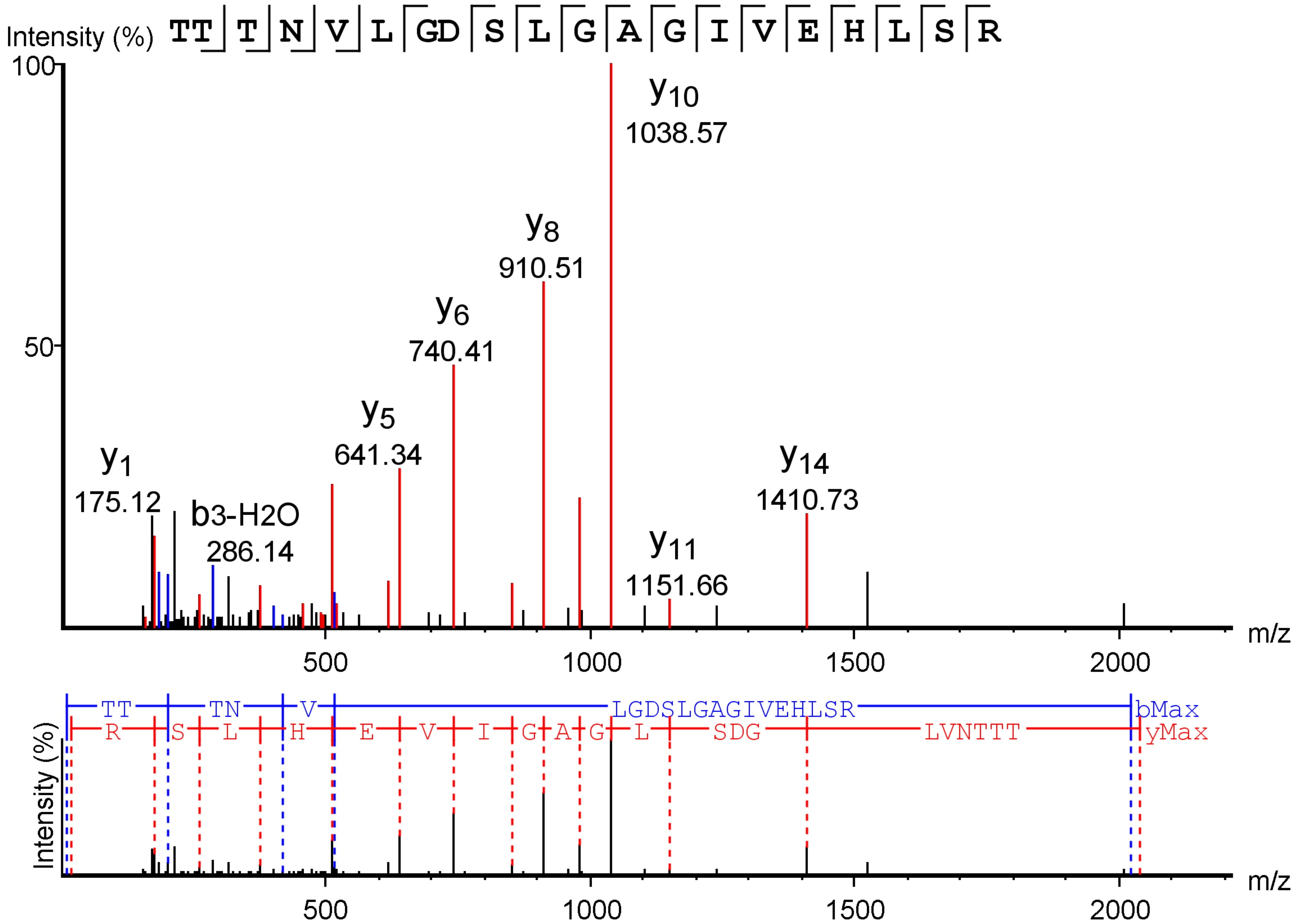

sp|O00391|QSOX1_HUMAN

K.AHFSPSNIILDFPAAGSAAR.R

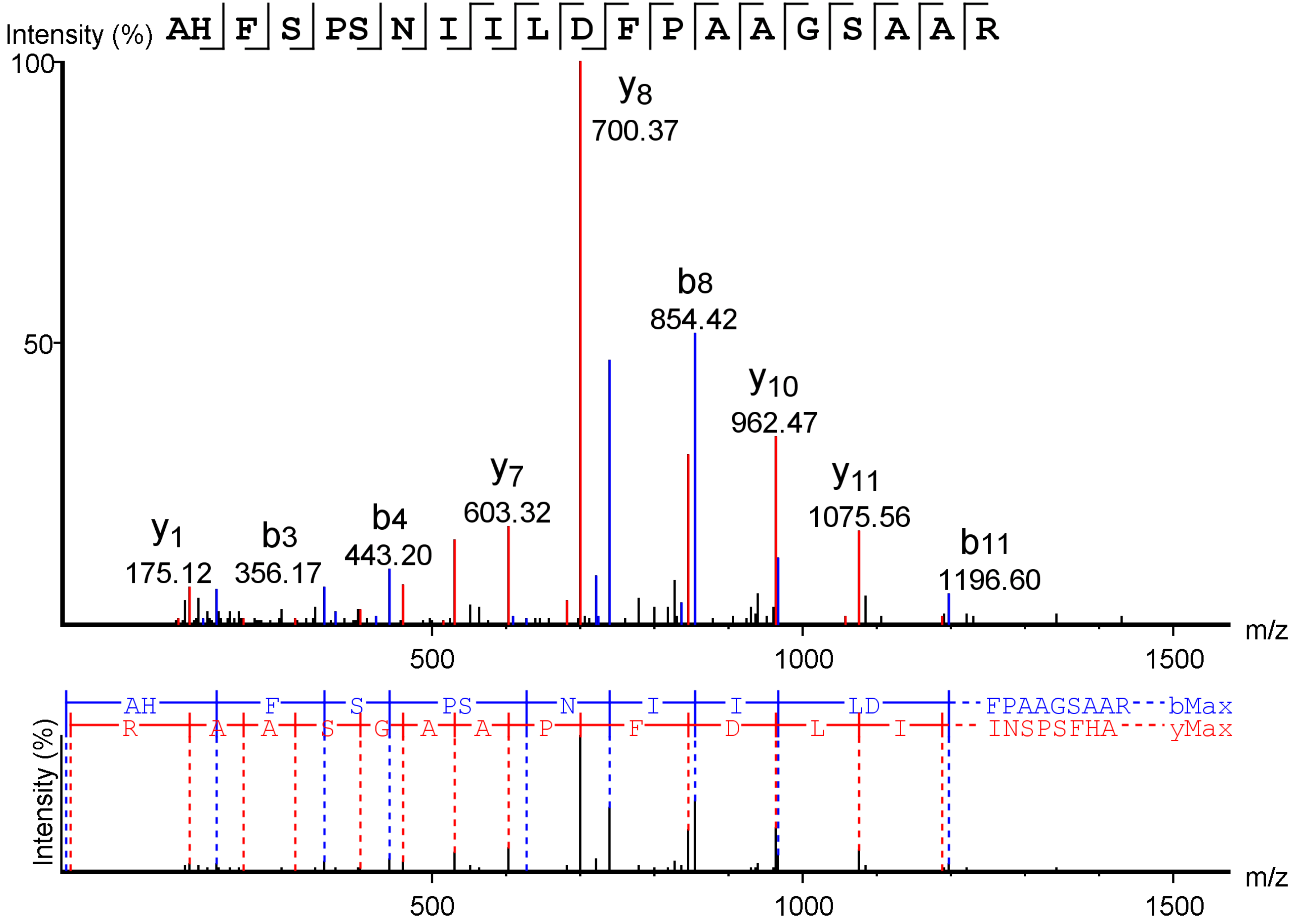

B1AK88|B1AK88_HUMAN

K.DETVSDC(+57.02)SPHIANIGR.L

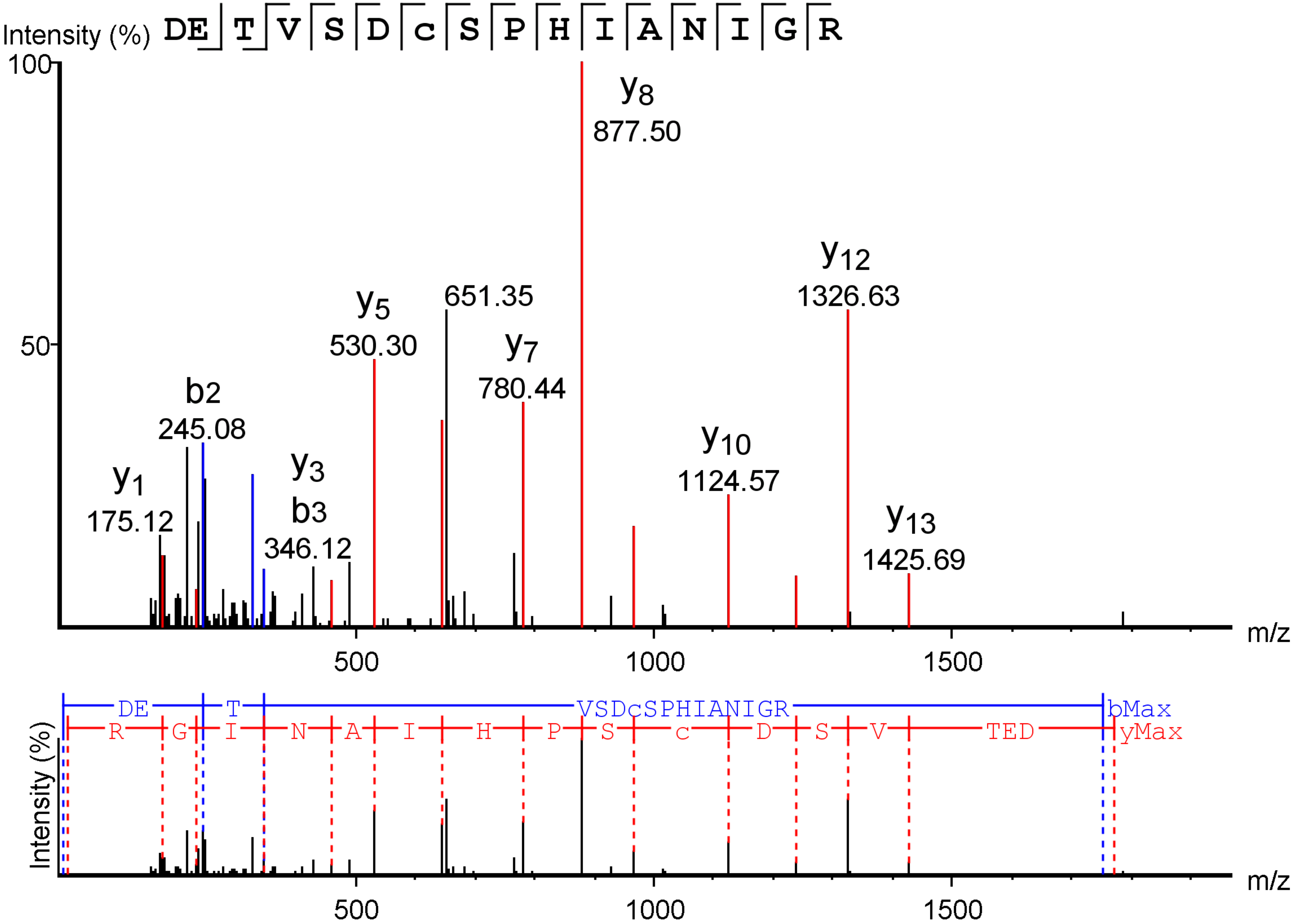

sp|Q07866|KLC1_HUMAN
K.DAANLLNDALAIR.E

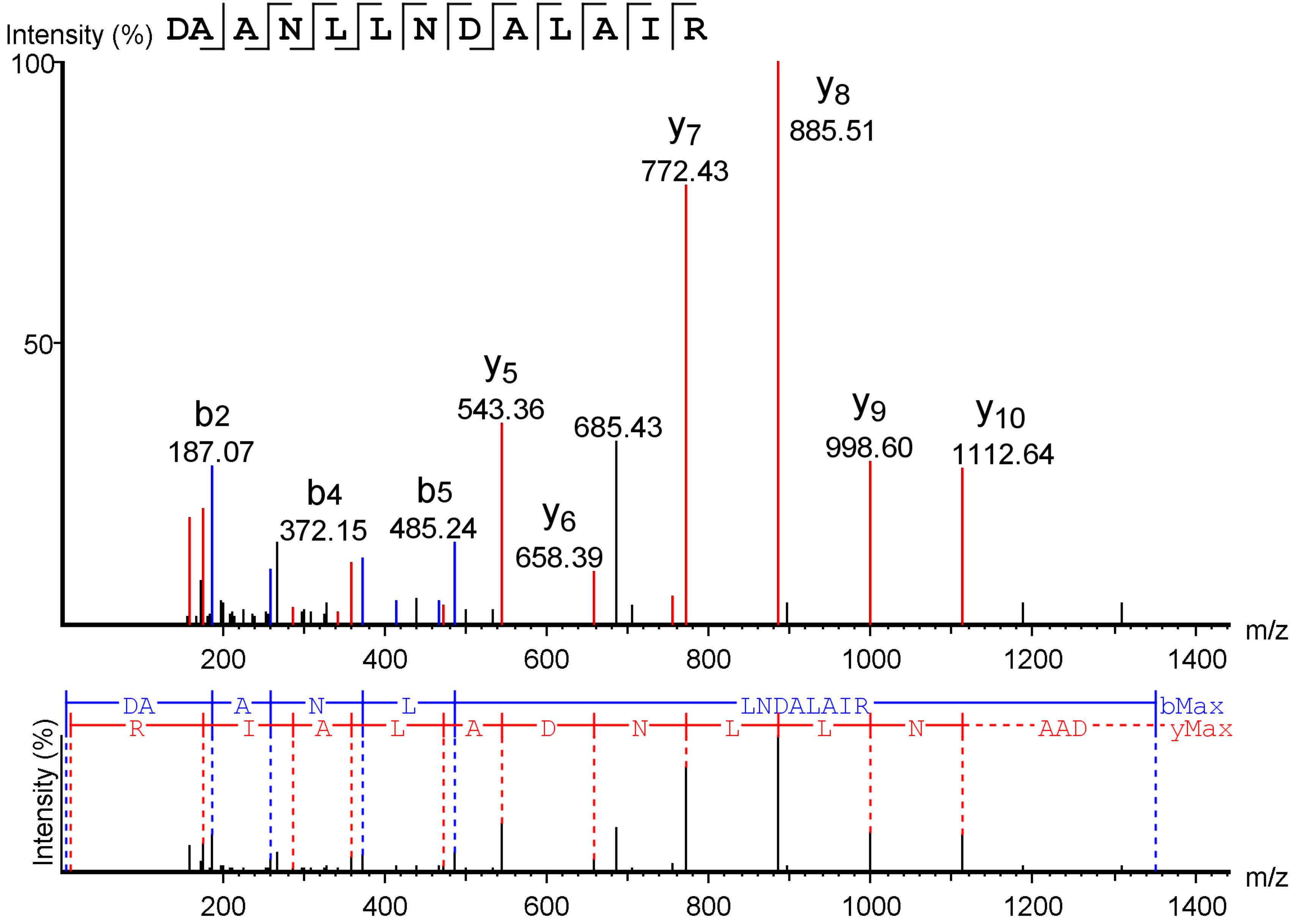

sp|Q14517|FAT1_HUMAN

K.HFVIDSATGIITLSNLHR.H

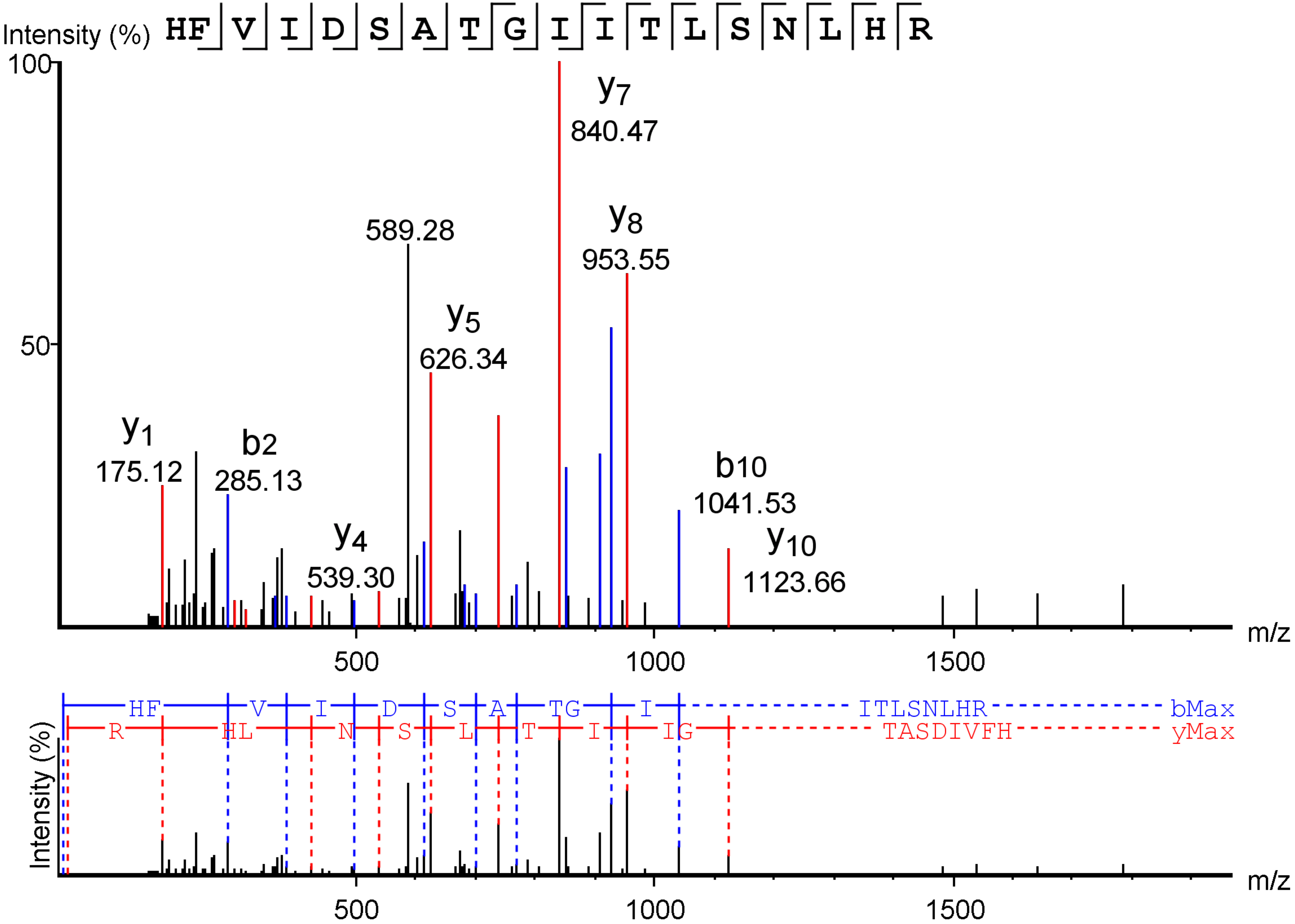

sp|Q99460|PSMD1_HUMAN

R.TPEQC(+57.02)PSVVSLLSESYNPHVR.Y

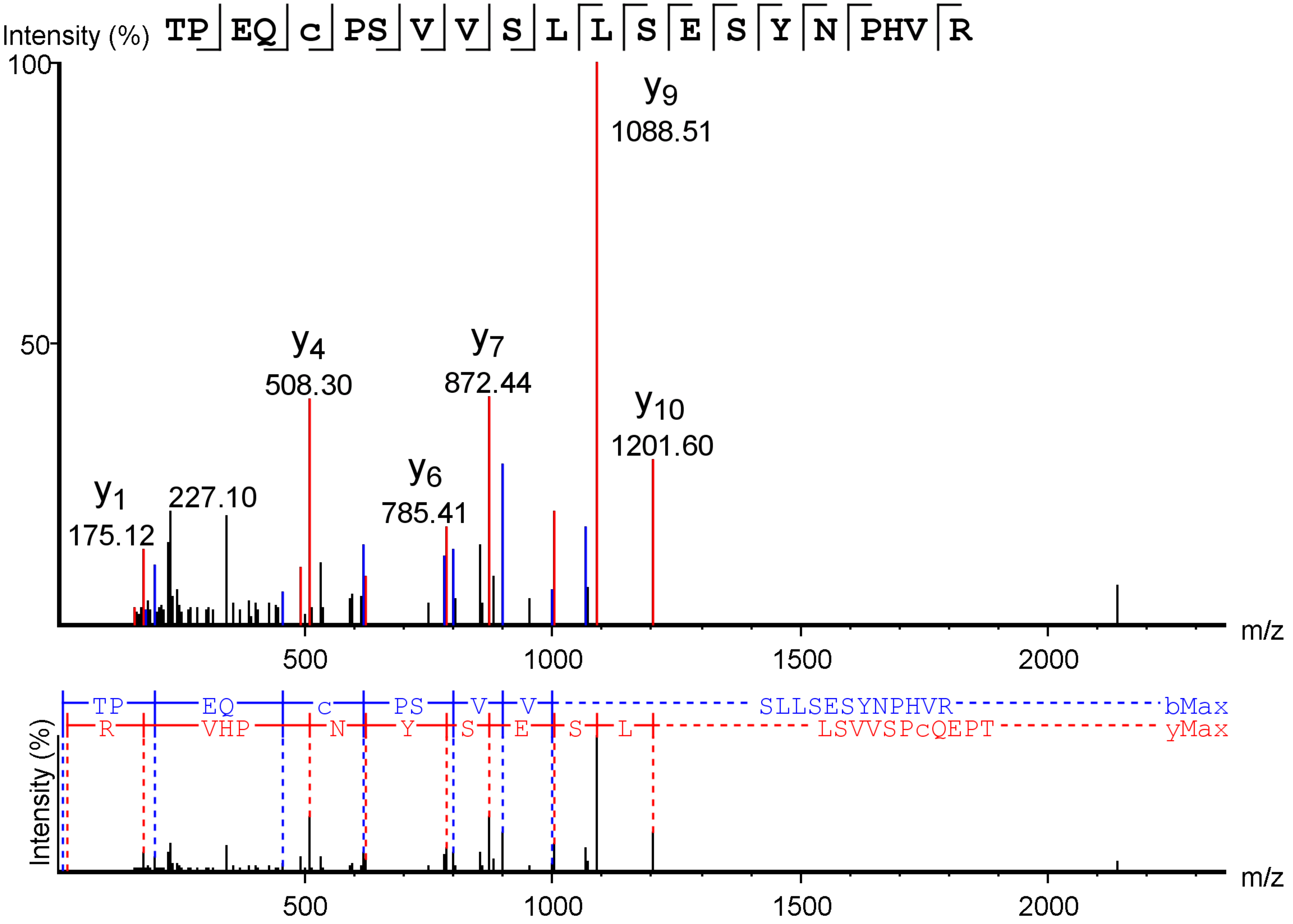

sp|Q15393|SF3B3_HUMAN
K.TPVEEVPAAIAPFQGR.V

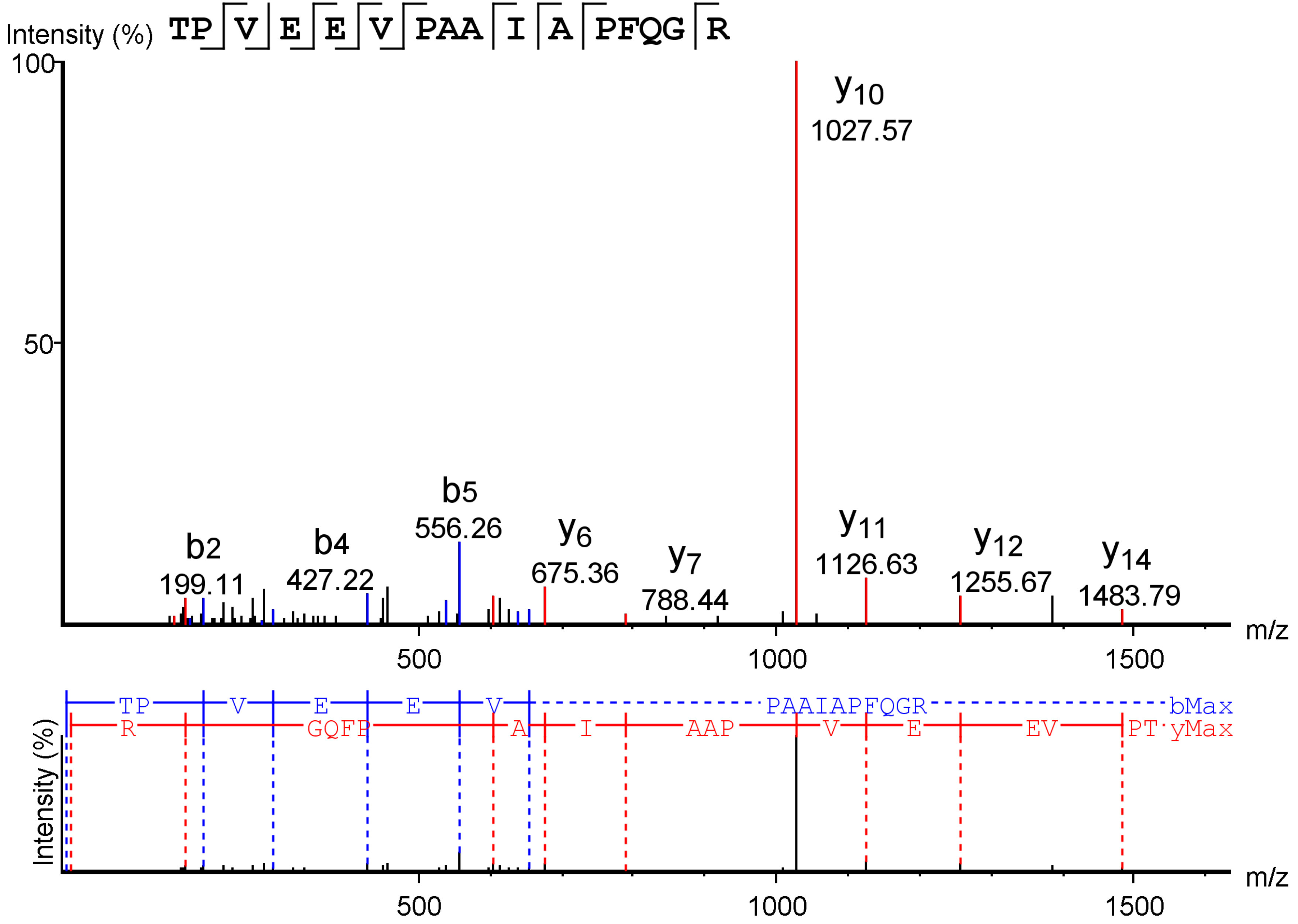

sp|Q9HAR2|AGRL3_HUMAN

K.VYLADPVVFTVK.H

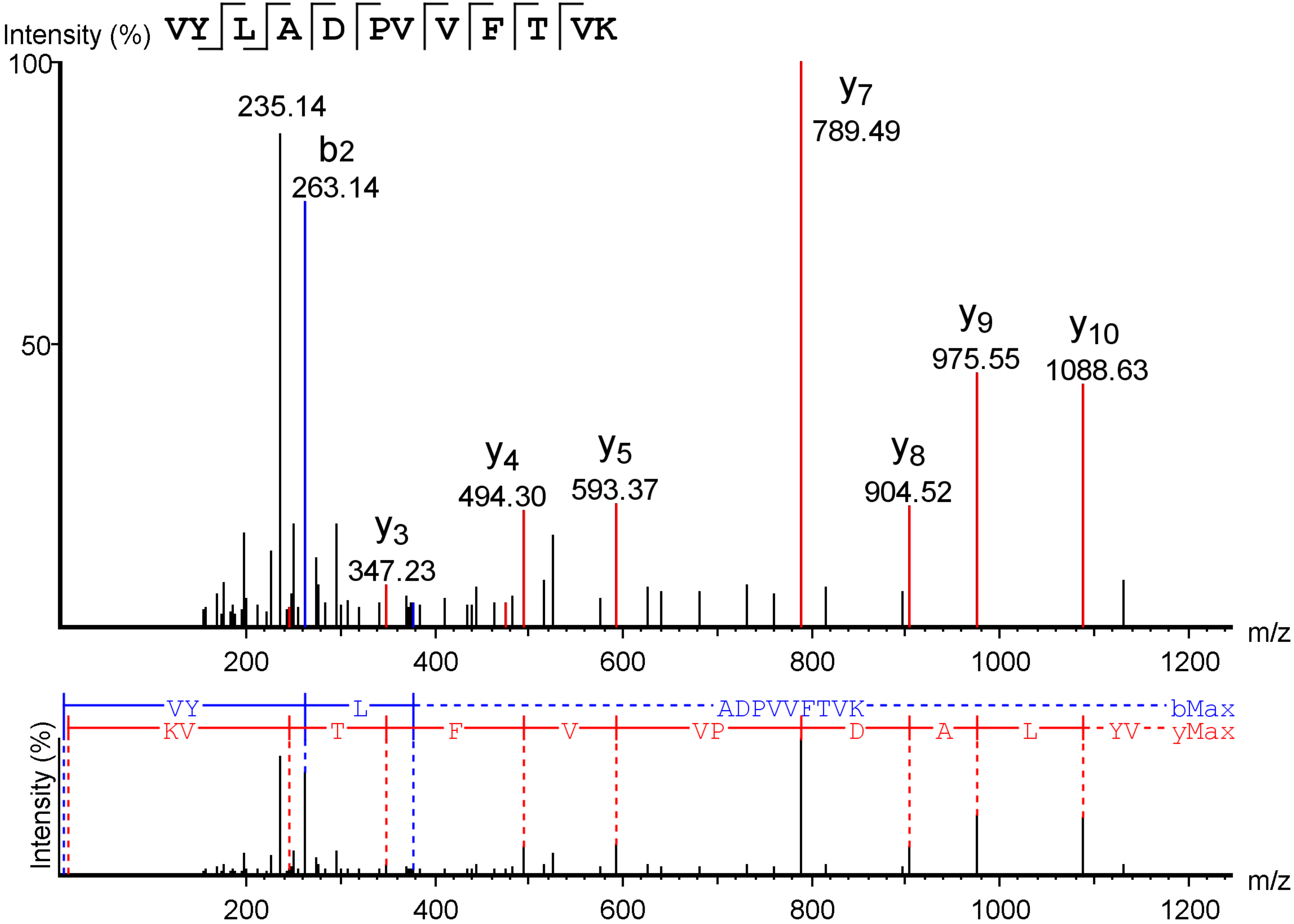

sp|P24534|EF1B_HUMAN
K.SPAGLQVLNDYLADK.S

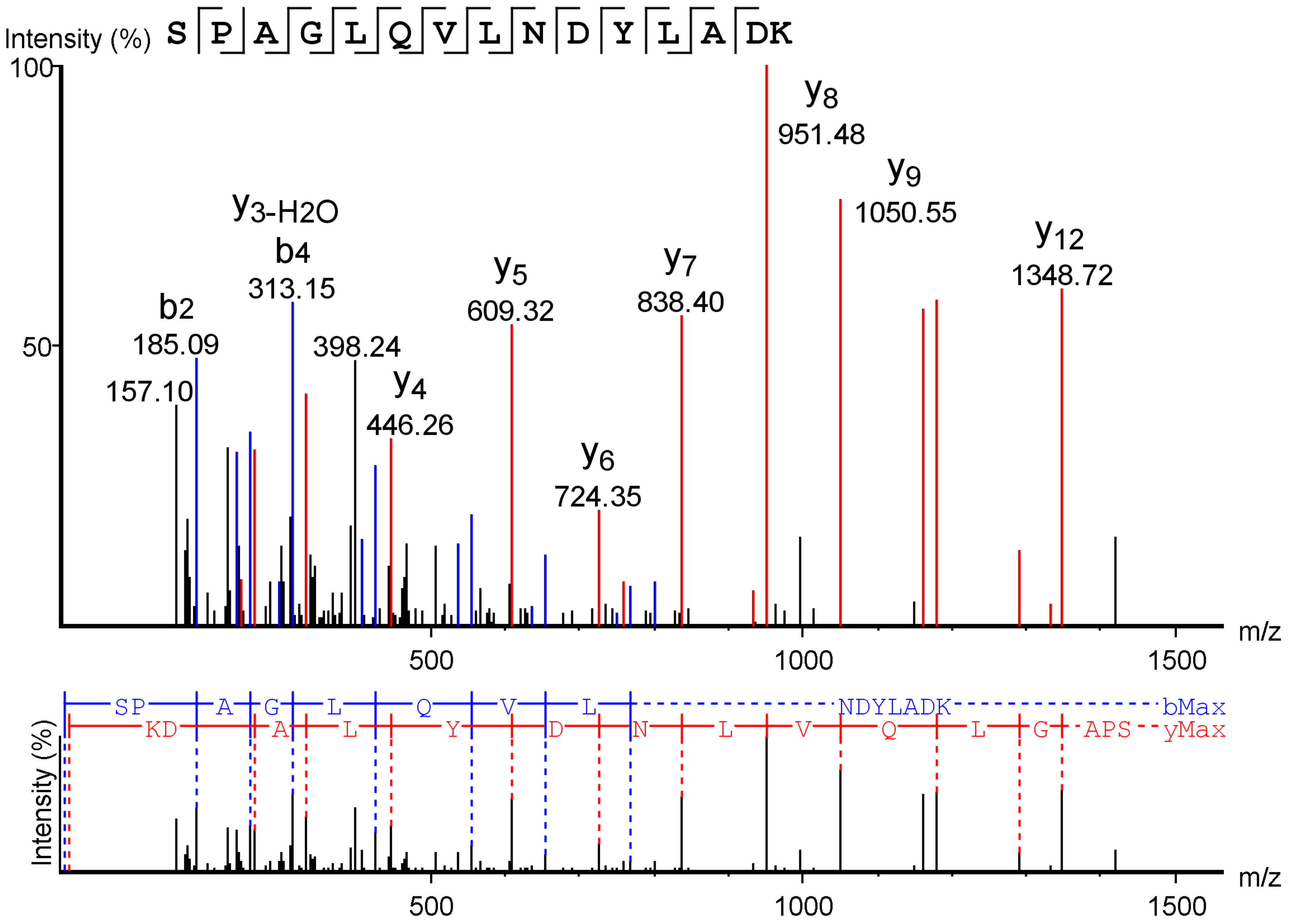

sp|P47914|RL29_HUMAN
K.AQAAAPASVPAQAPK.R

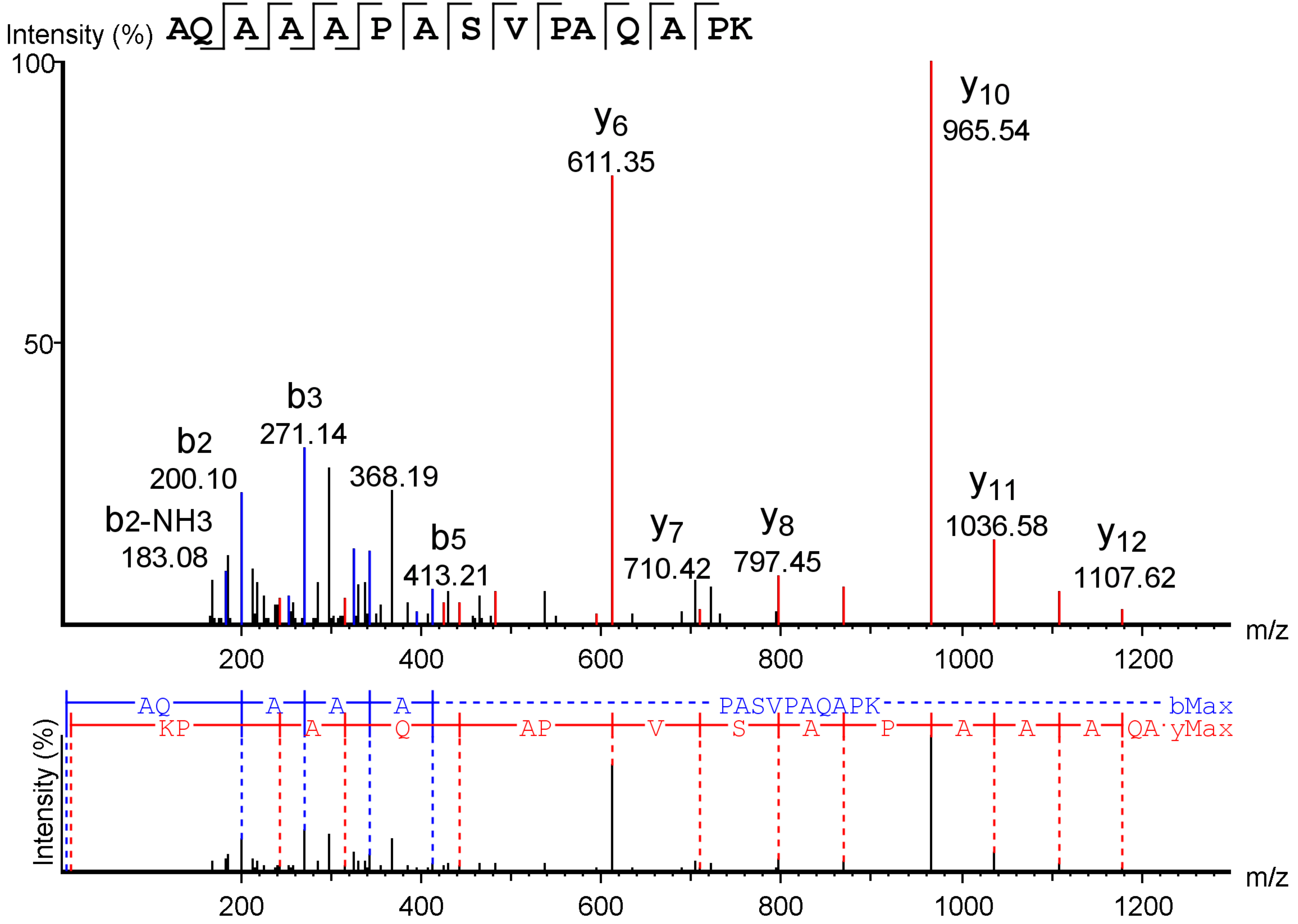

sp|P00441|SODC_HUMAN

K.GLTEGLHGFHVHEFGDNTAGC(+57.02)TSAGPHFNPLSR.K

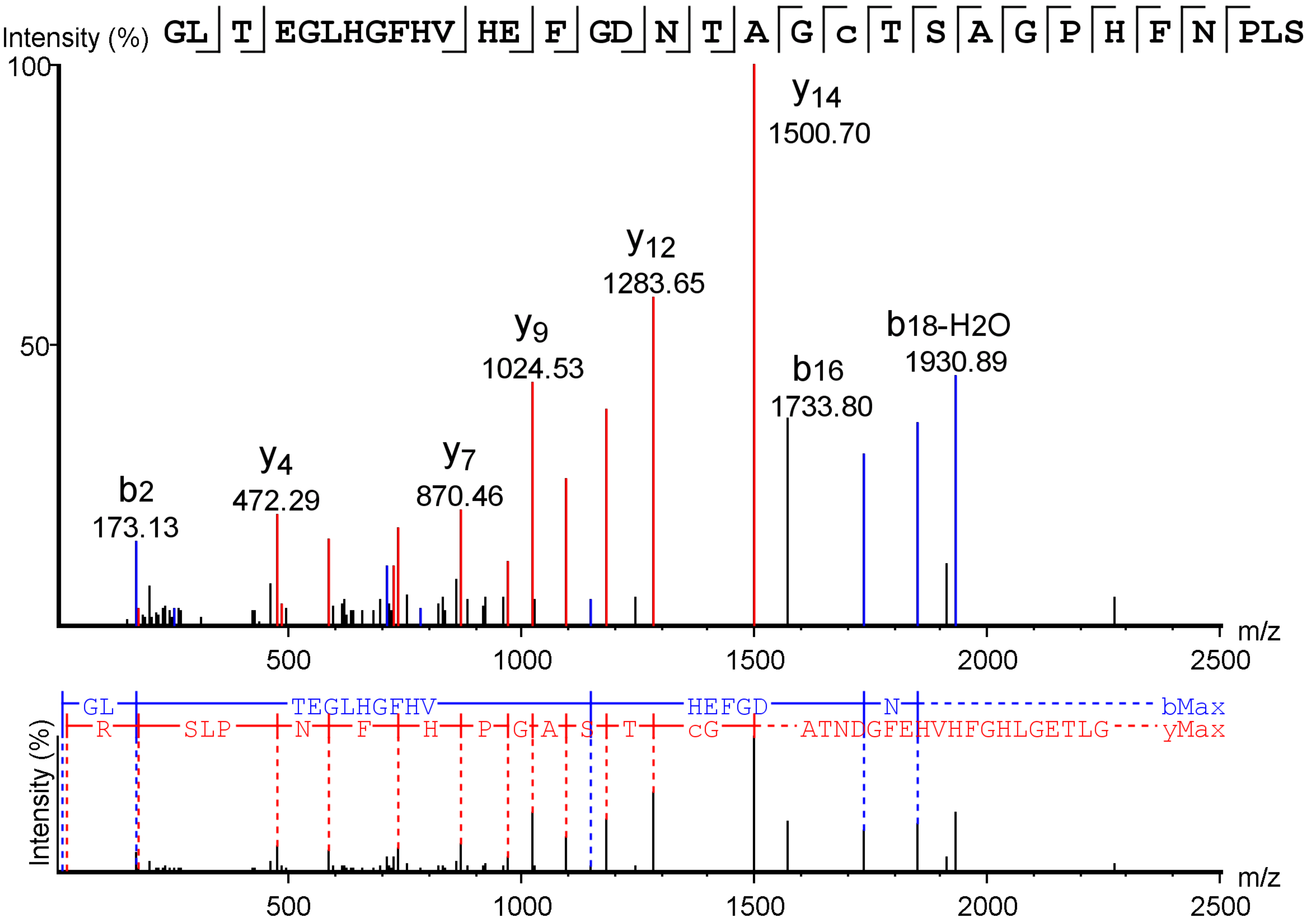

sp|Q9H7P6|MB12B_HUMAN

R.NHDSSQPTTPSQSSAASTPAPNLPR.H

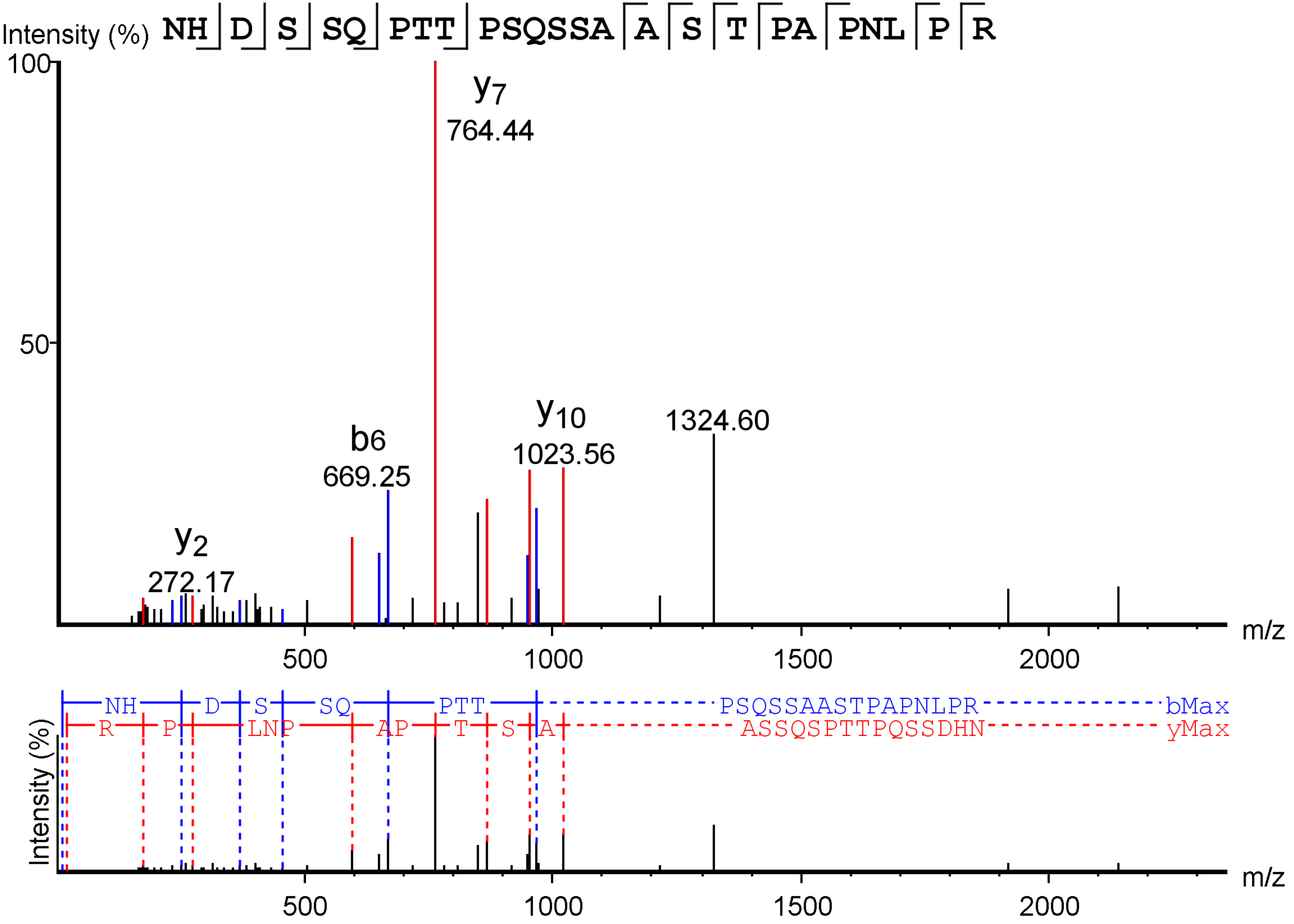

sp|P09486|SPRC_HUMAN

K.LHLDYIGPC(+57.02)K.Y

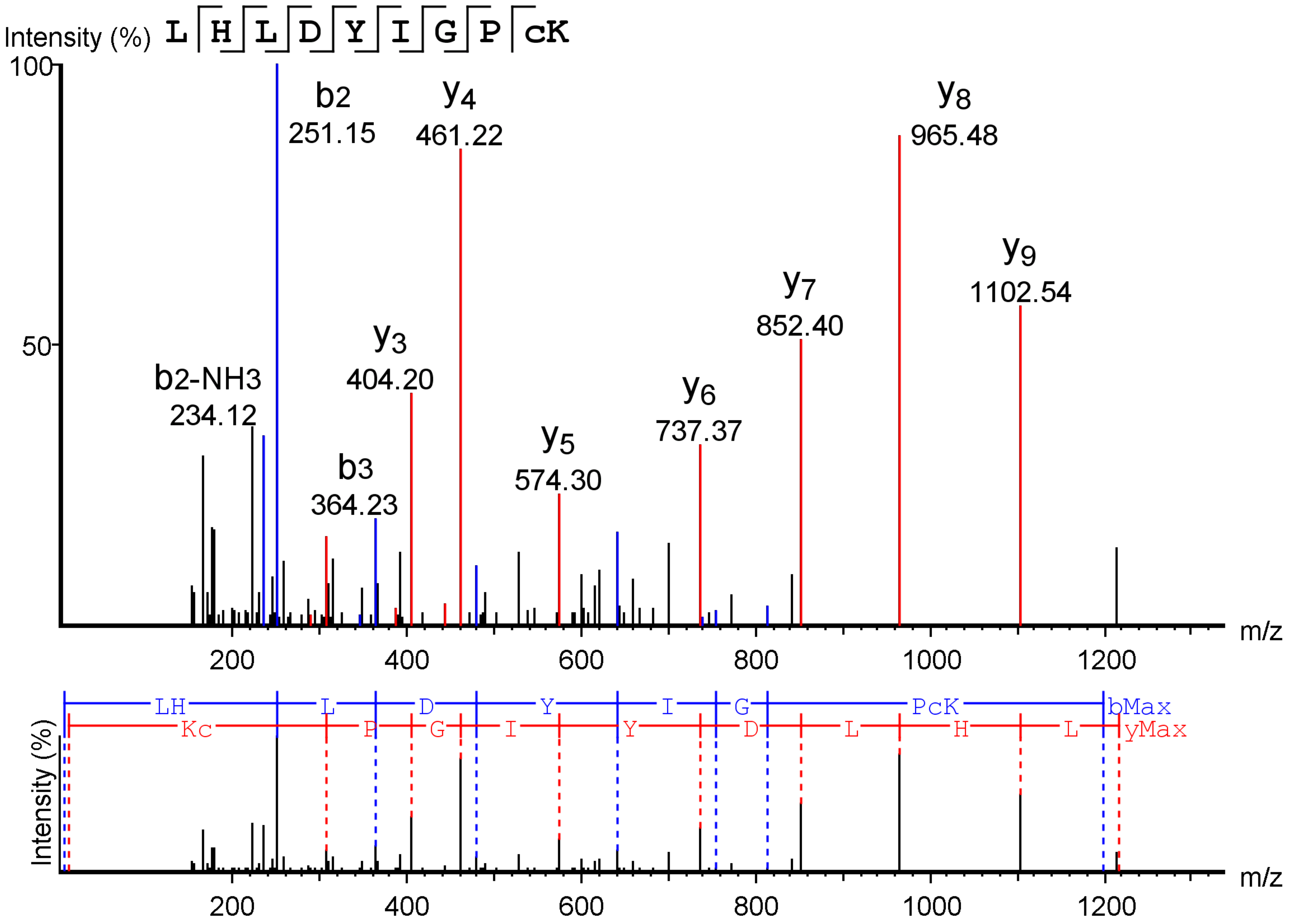

sp|O60568|PLOD3_HUMAN

K.LVGPEEALSPGEAR.D

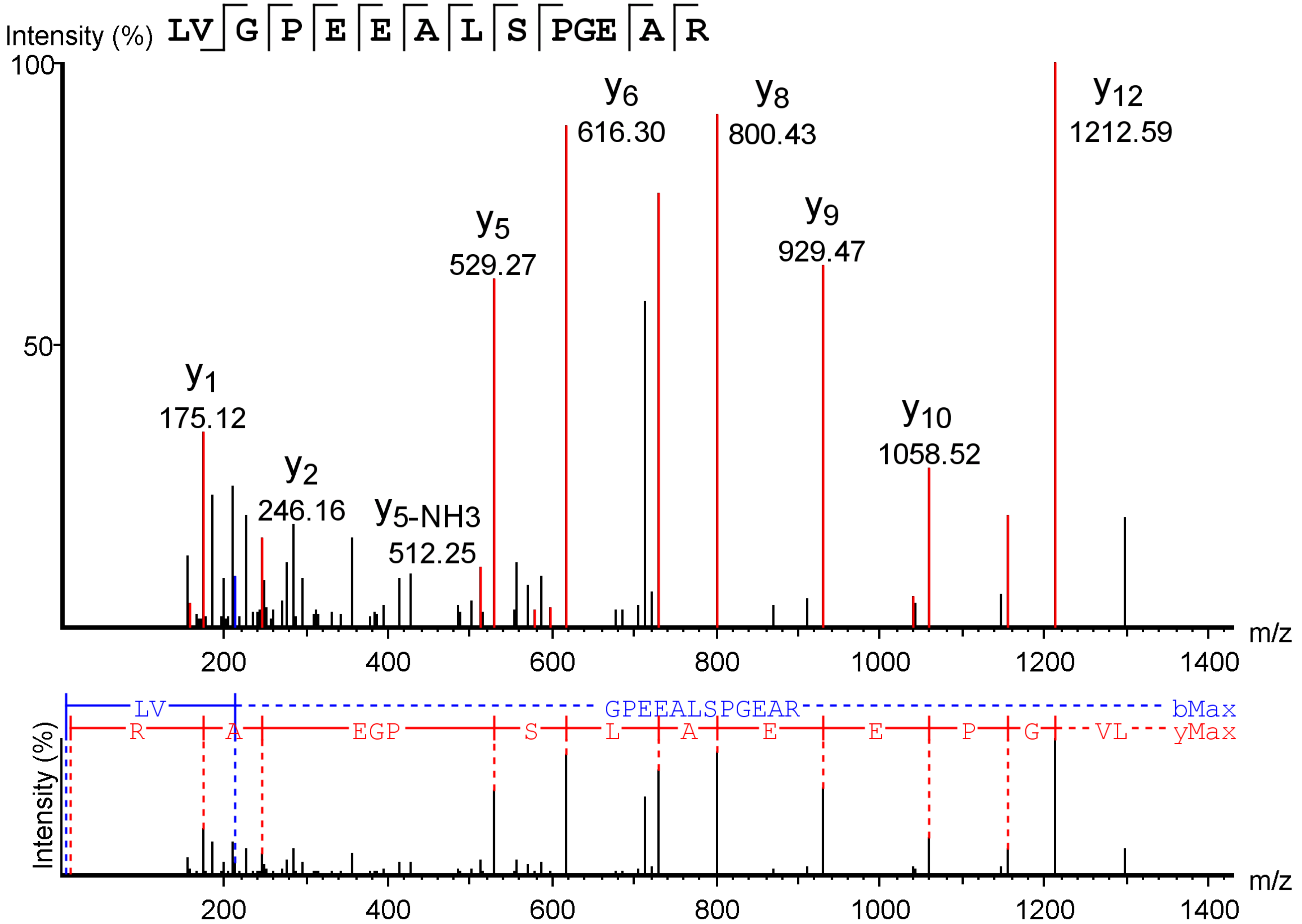

sp|Q01955|CO4A3_HUMAN
R.ASPFLEC(+57.02)HGR.G

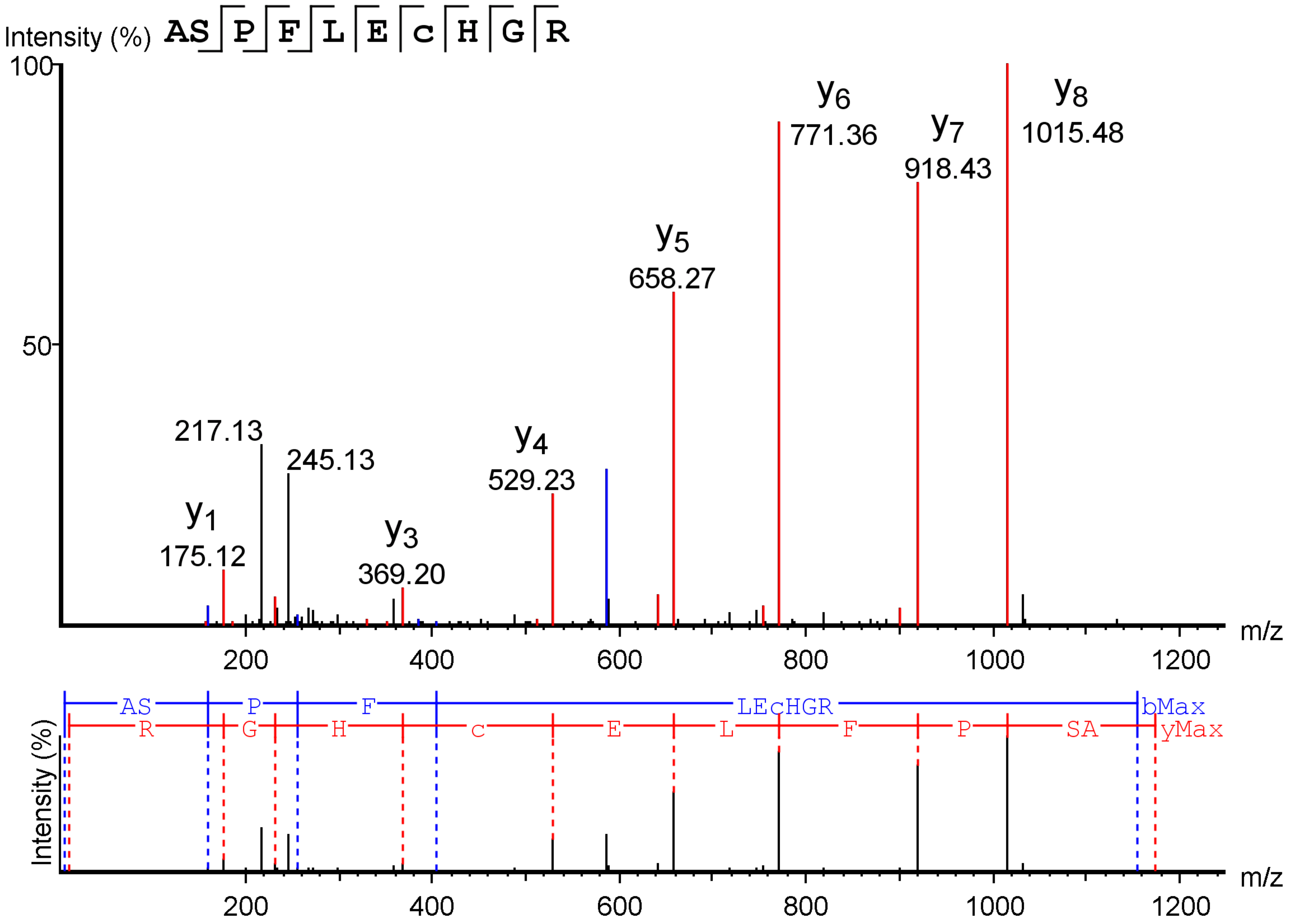

H0YH88|H0YH88_HUMAN

K.YAVLYQPLFDK.R

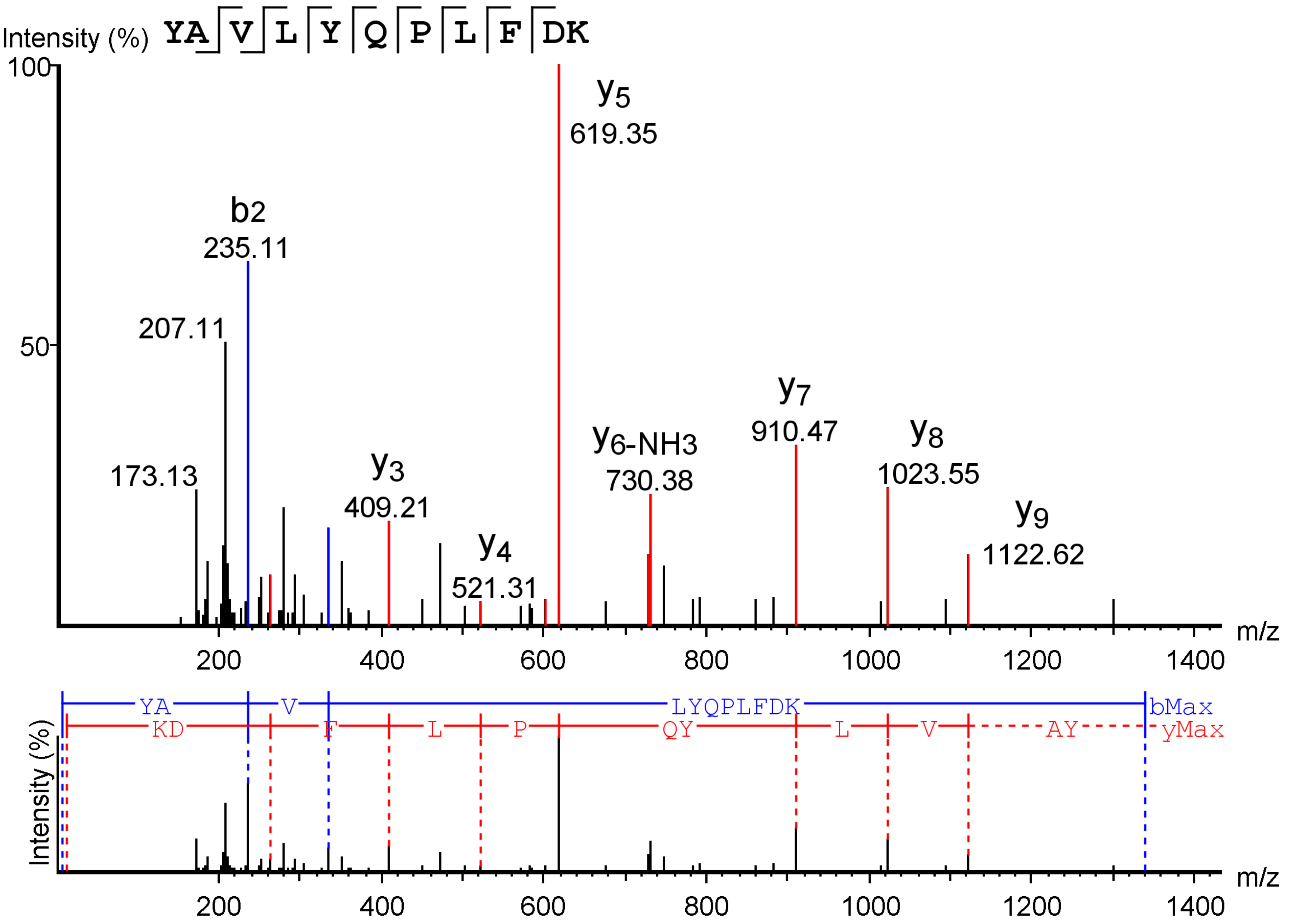

sp|Q8WWI5|CTL1_HUMAN
K.LPVPASAPIPFFHR.C

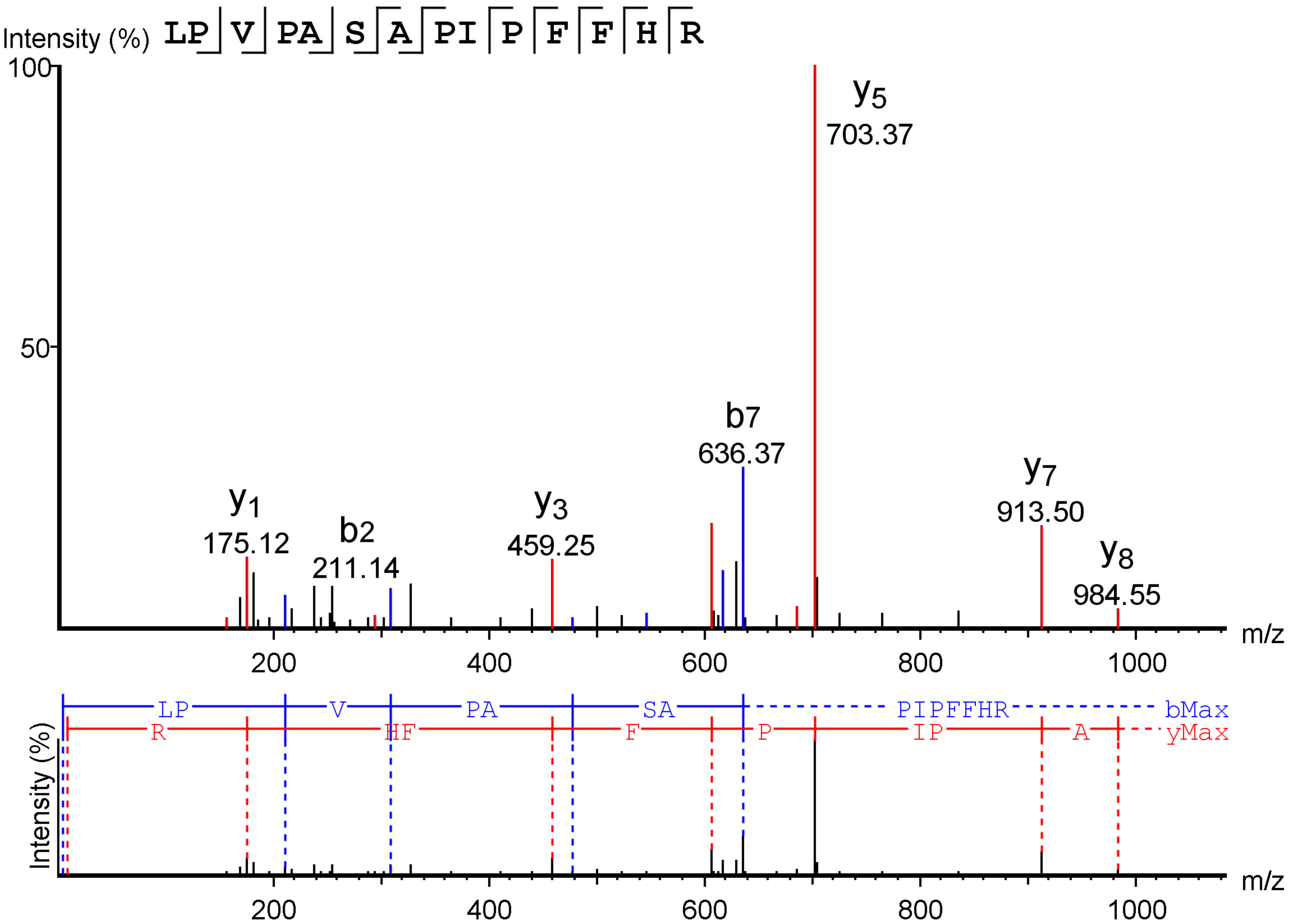

sp|P15924|DESP_HUMAN

R.TM(+15.99)IQSPSGVILQEAADVHAR.Y

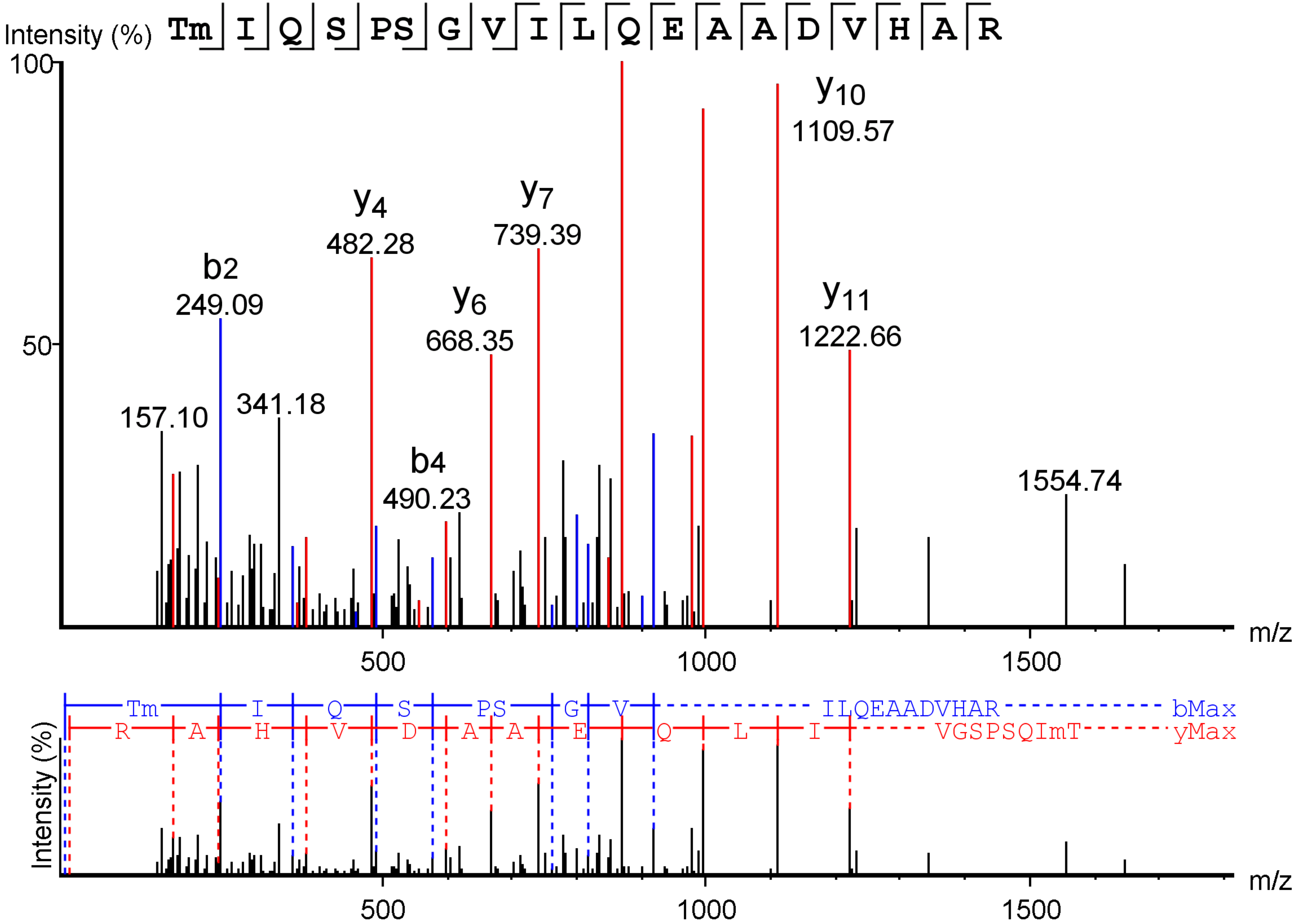

sp|P20073|ANXA7_HUMAN
K.GFGTDEQAIVDVVANR.S

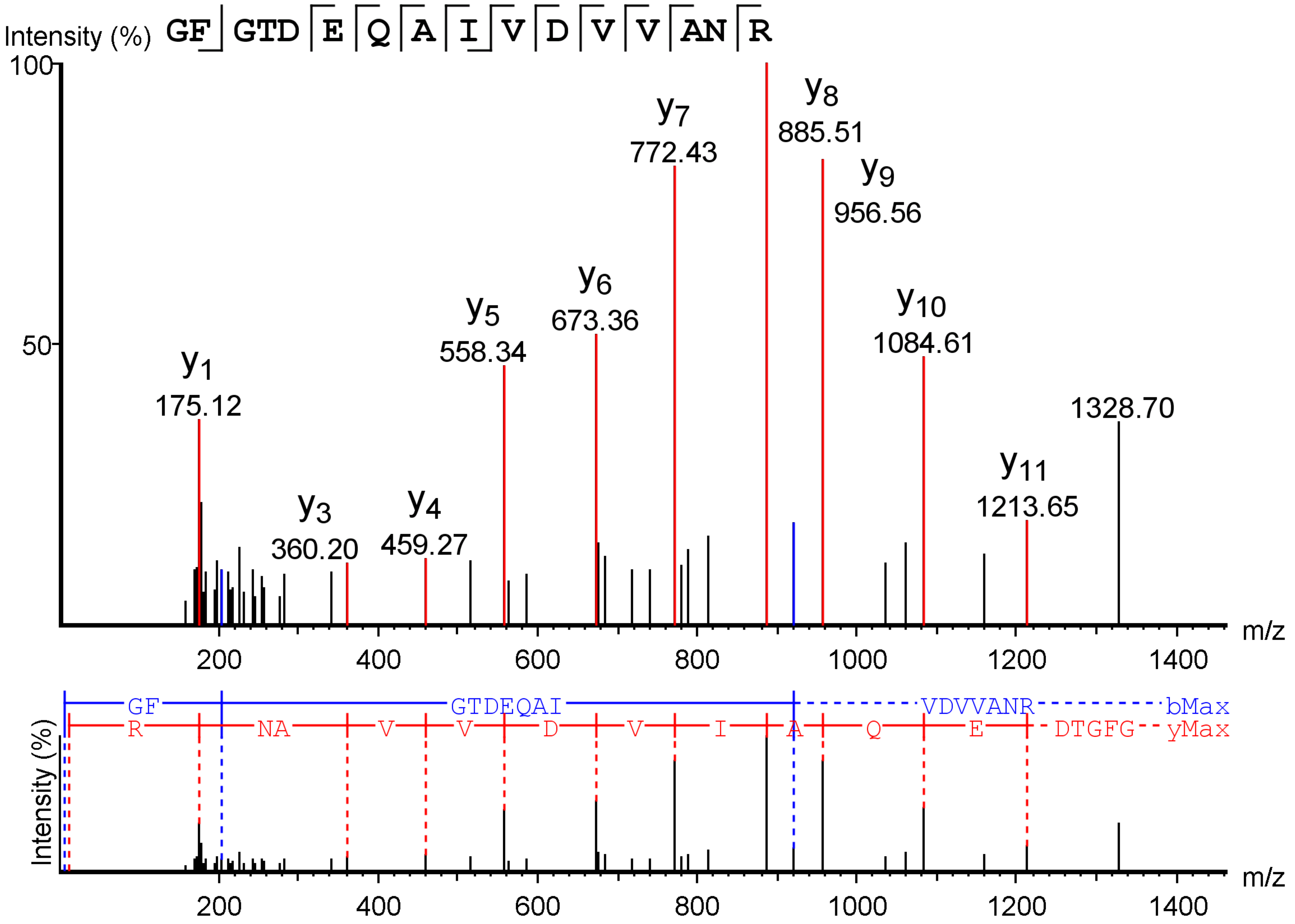

sp|P12270|TPR_HUMAN
R.ASTALSNEQQAR.R

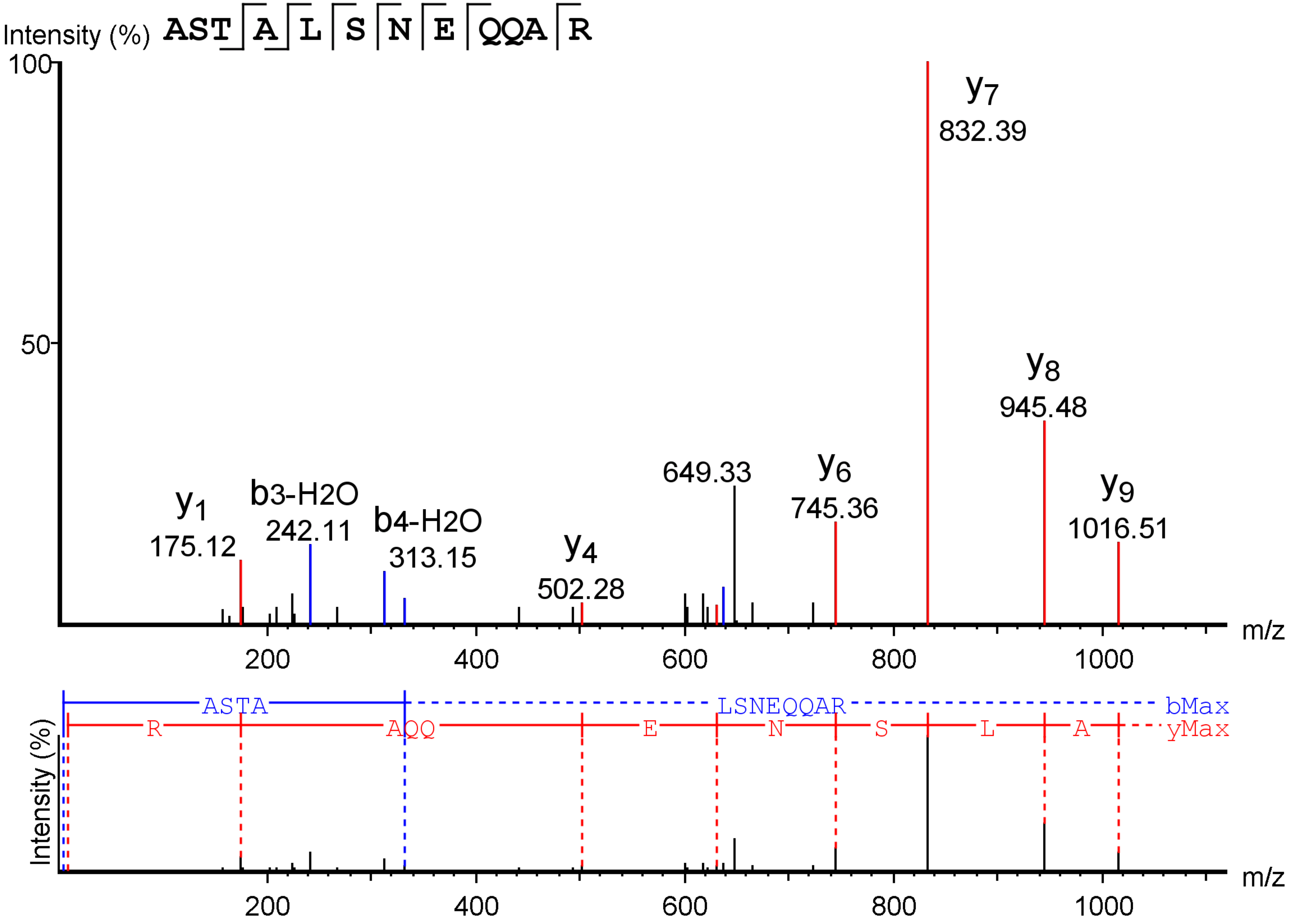

sp|Q96A00|PP14A_HUMAN
R.GPGGSPGGLQK.R

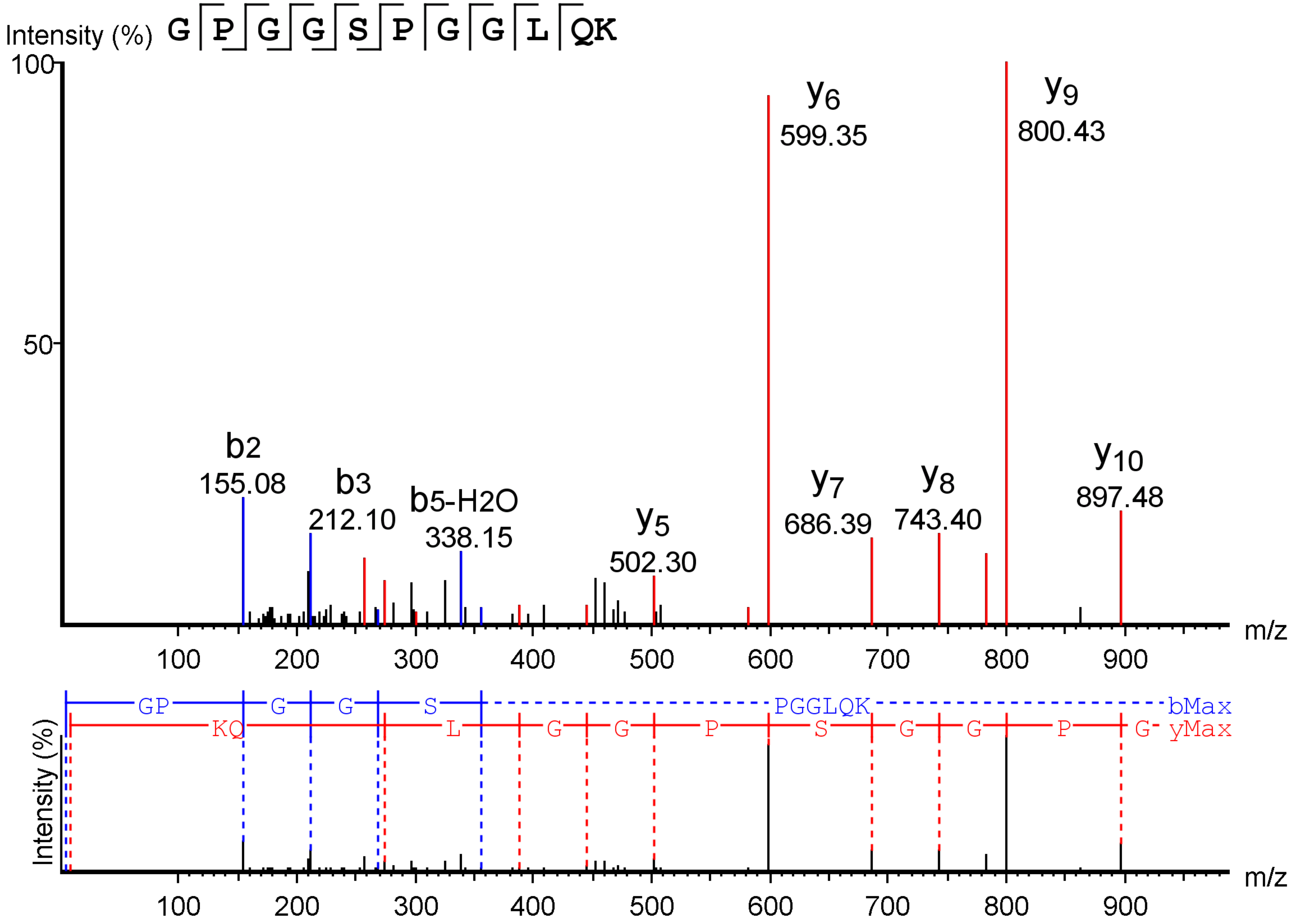

sp|P0DP01|HV108_HUMAN

R.SEDTAVYYC(+57.02)AR

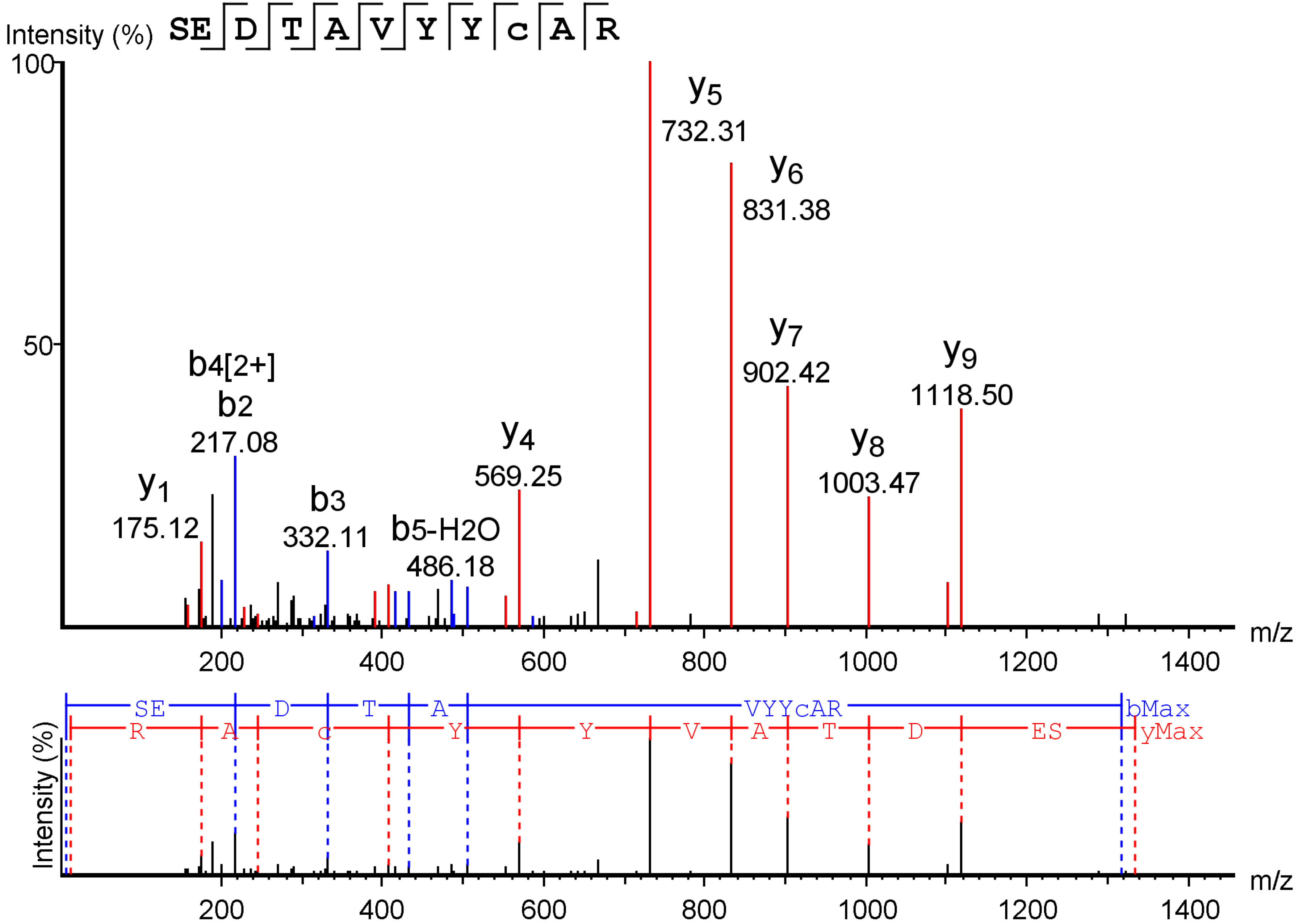

sp|Q00839|HNRPU_HUMAN

K.SSGPTSLFAVTVAPPGAR.Q

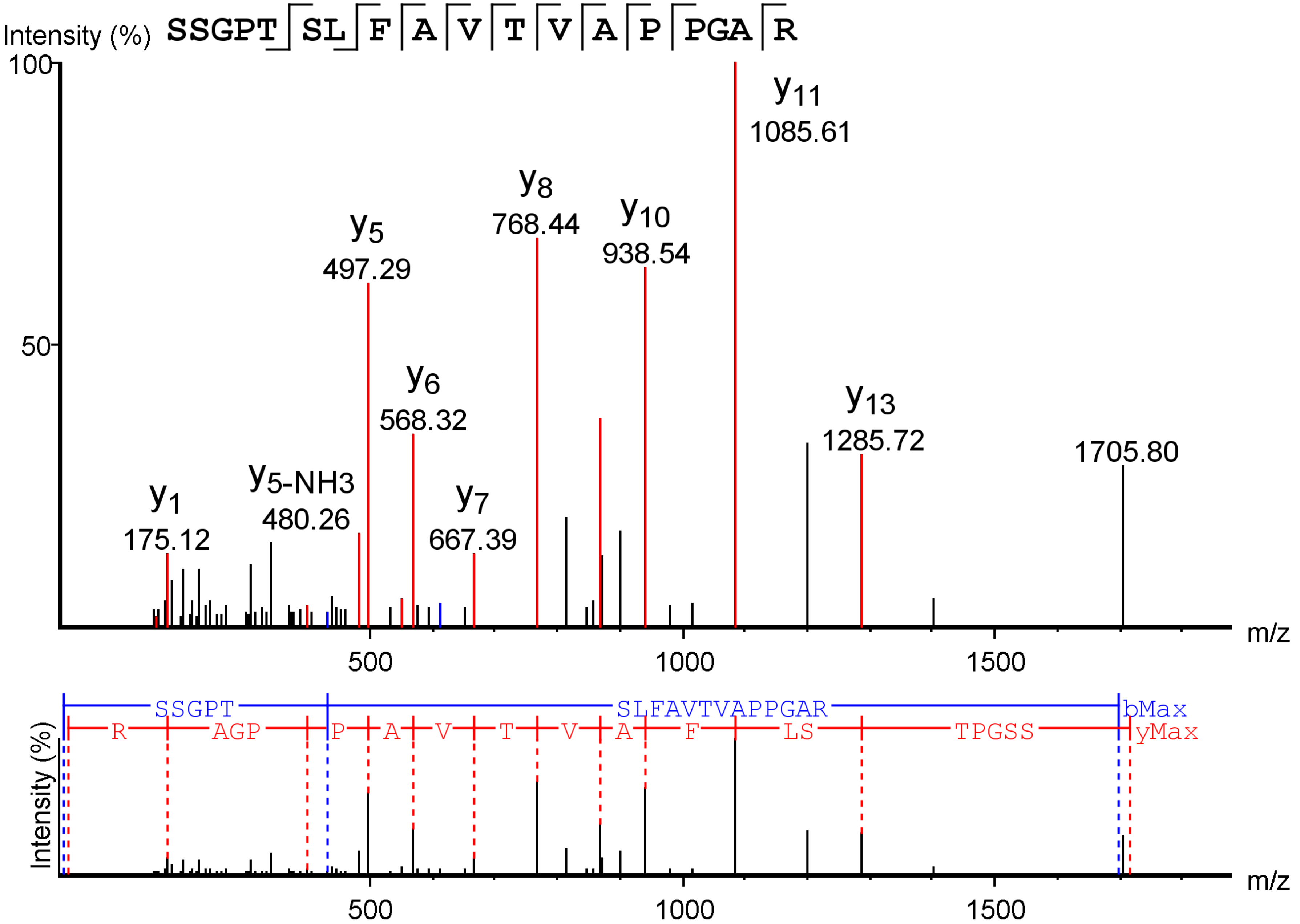

sp|P31948|STIP1_HUMAN

R.LAYINPDLALEEK.N

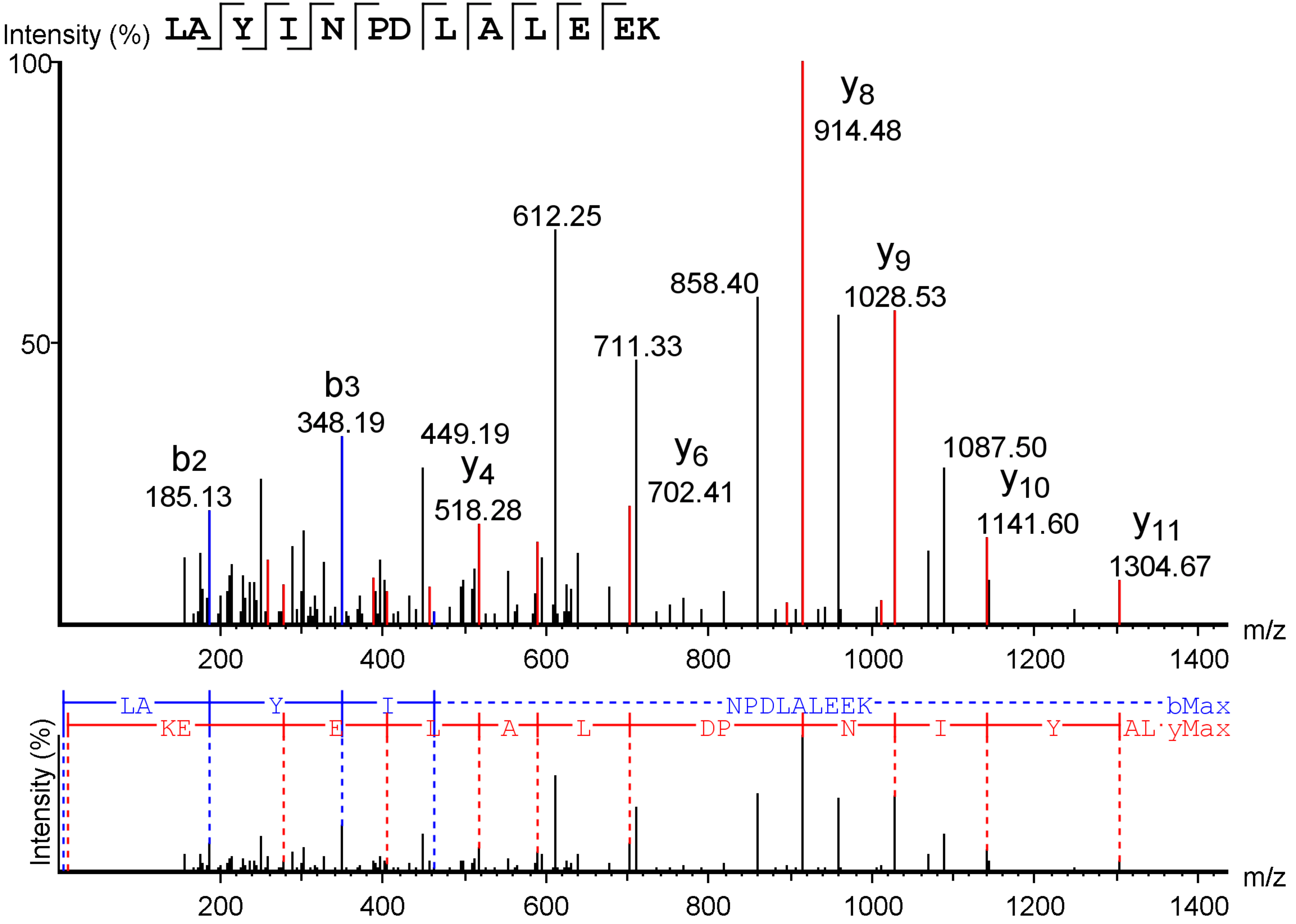

sp|Q9ULV4|COR1C_HUMAN
K.TTDTASVQNEAK.L

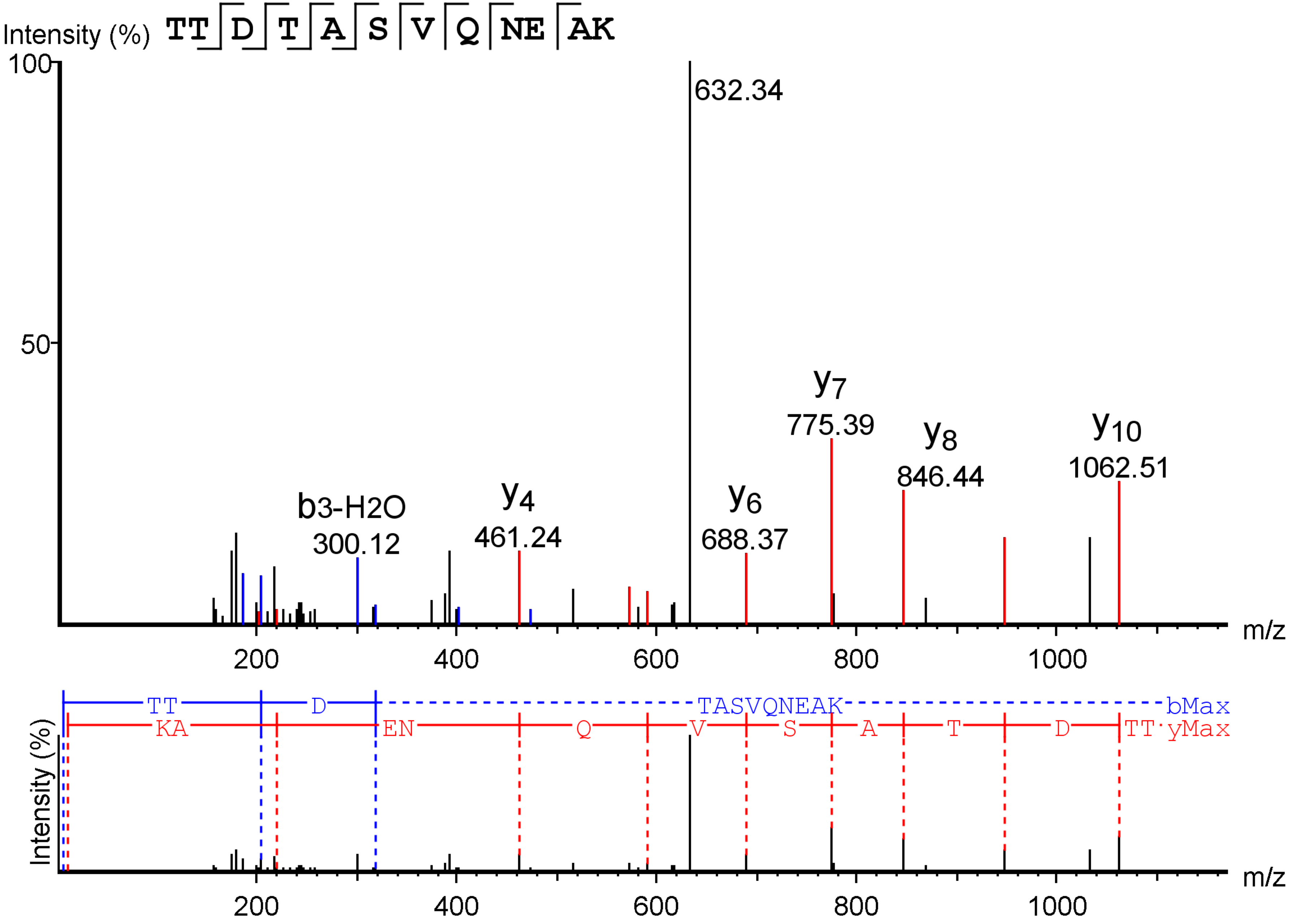

A8MRB1|A8MRB1_HUMAN
K.AMVALIDVFHQYSGR.E

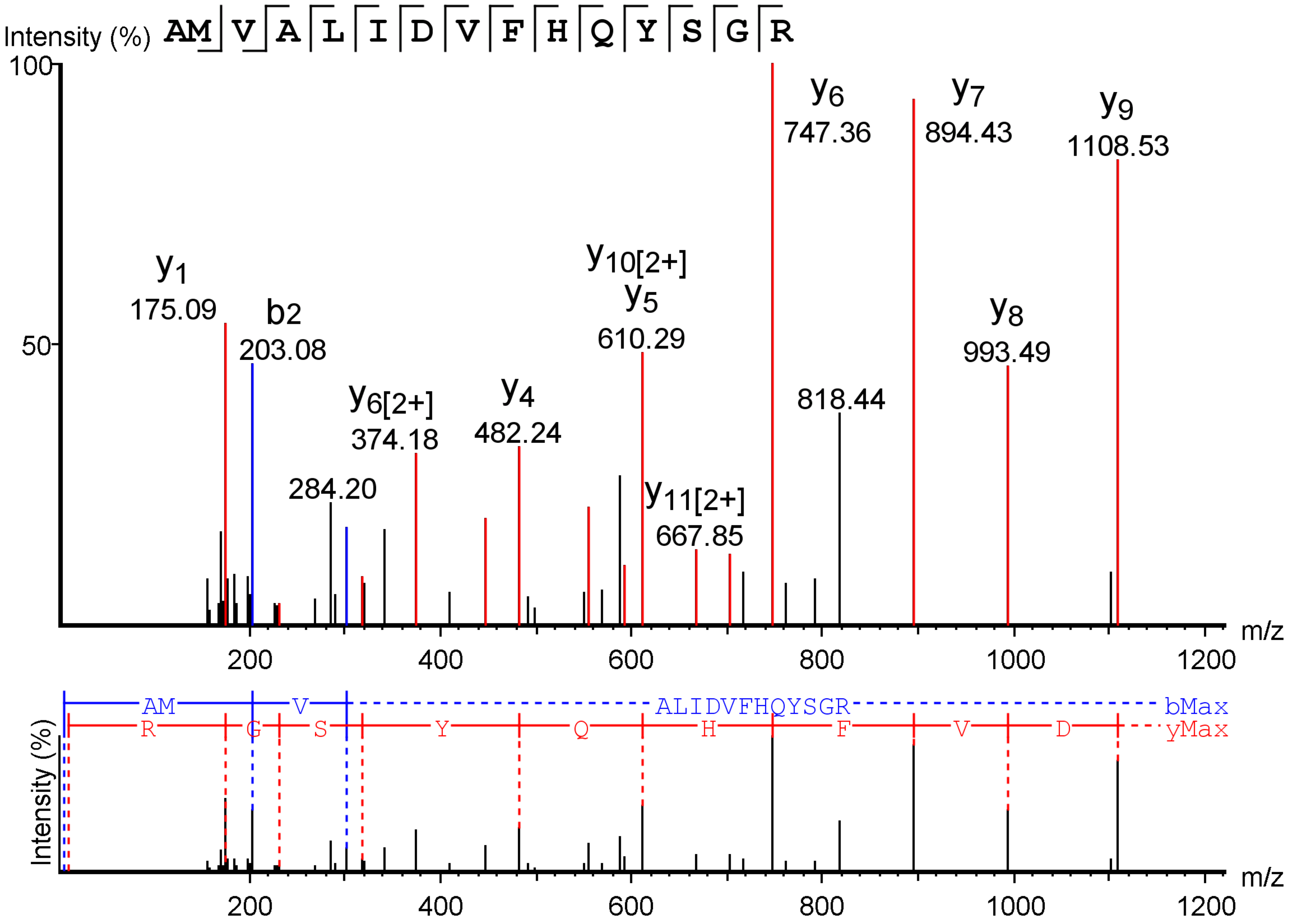

S4R460|S4R460_HUMAN

EVQLVESGGGLVQPGGSLR.L

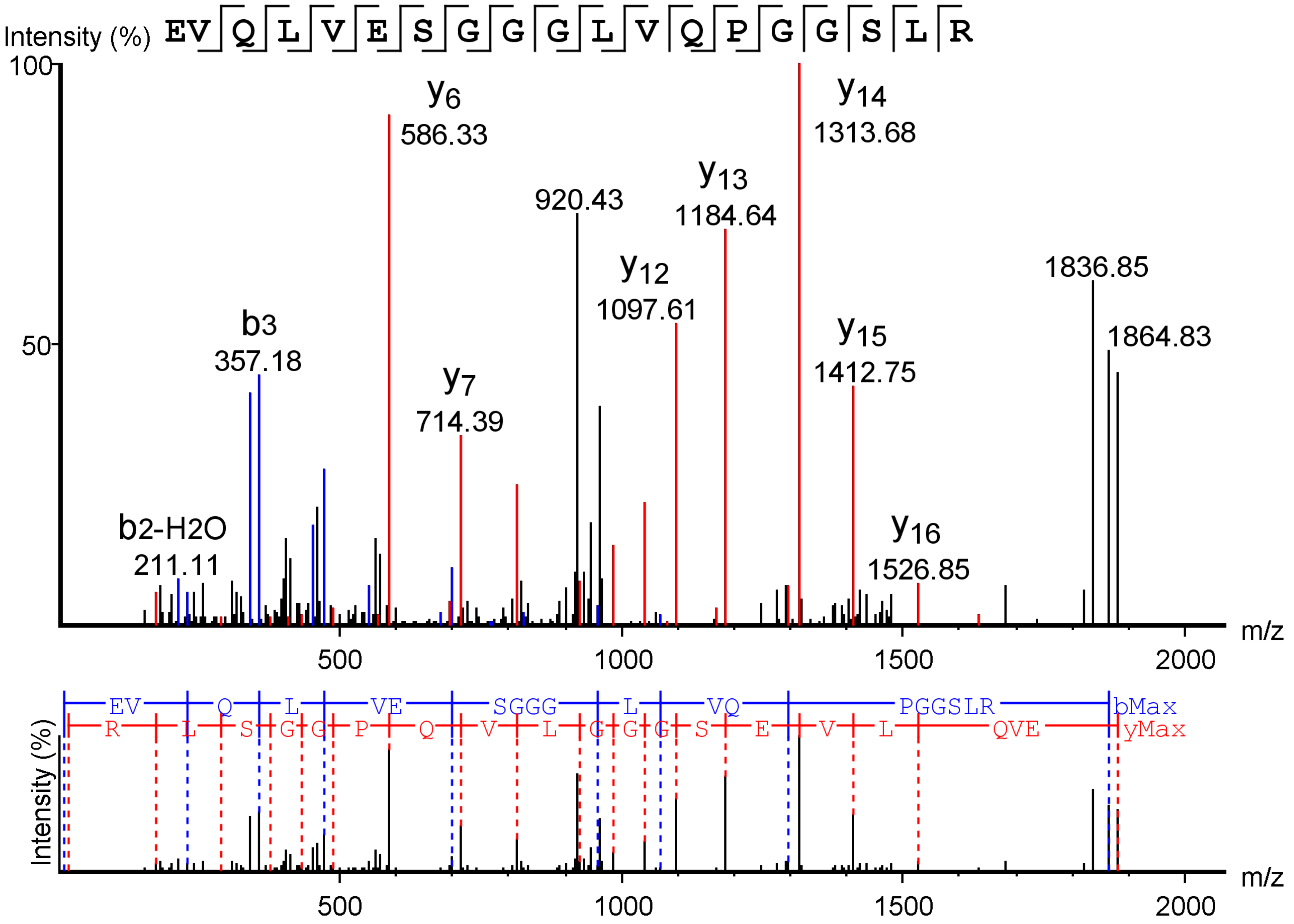

sp|P12956|XRCC6_HUMAN

R.DSLIFLVDASK.A

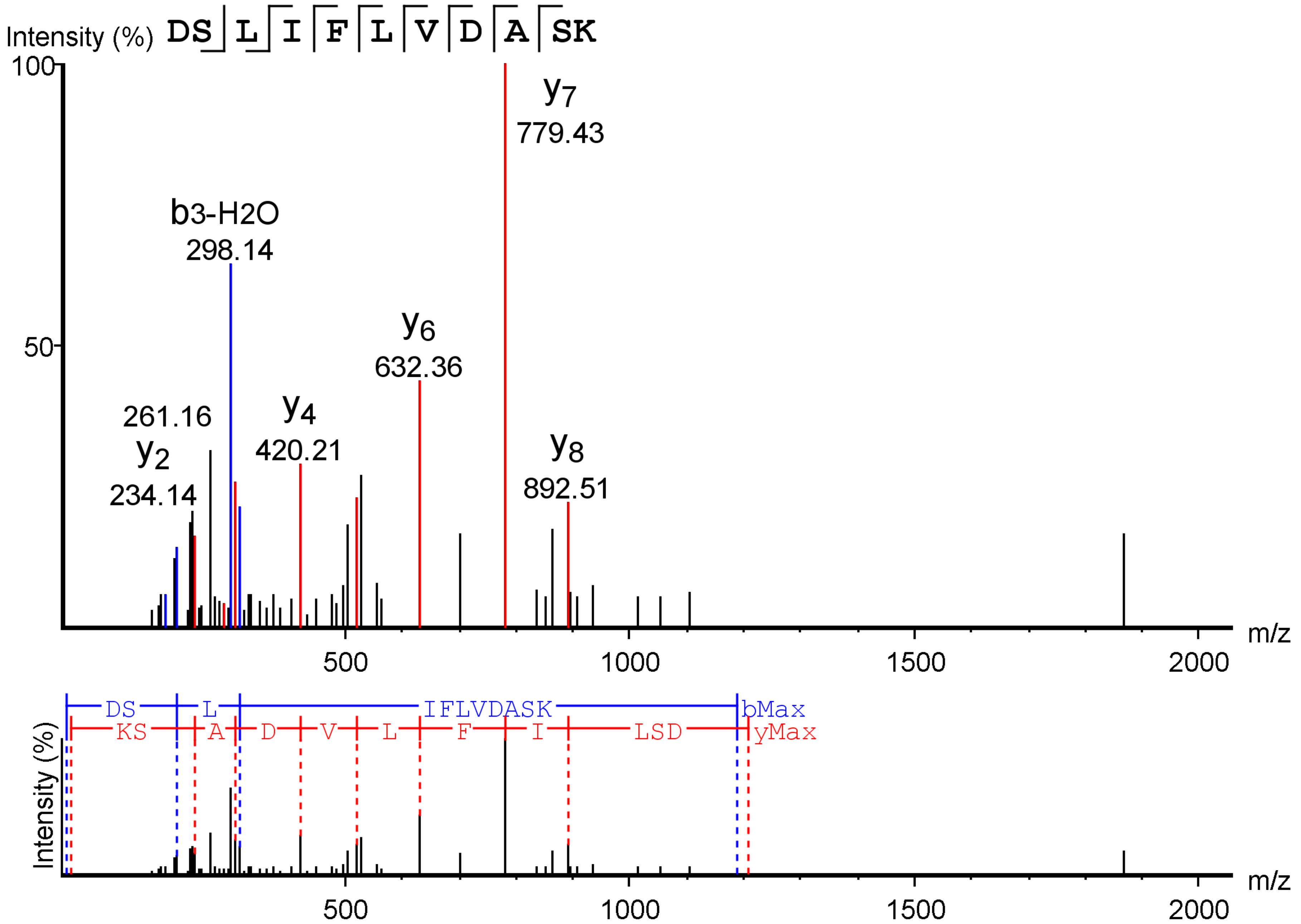

sp|P01591|IGJ_HUMAN

R.FVYHLSDLC(+57.02)K.K

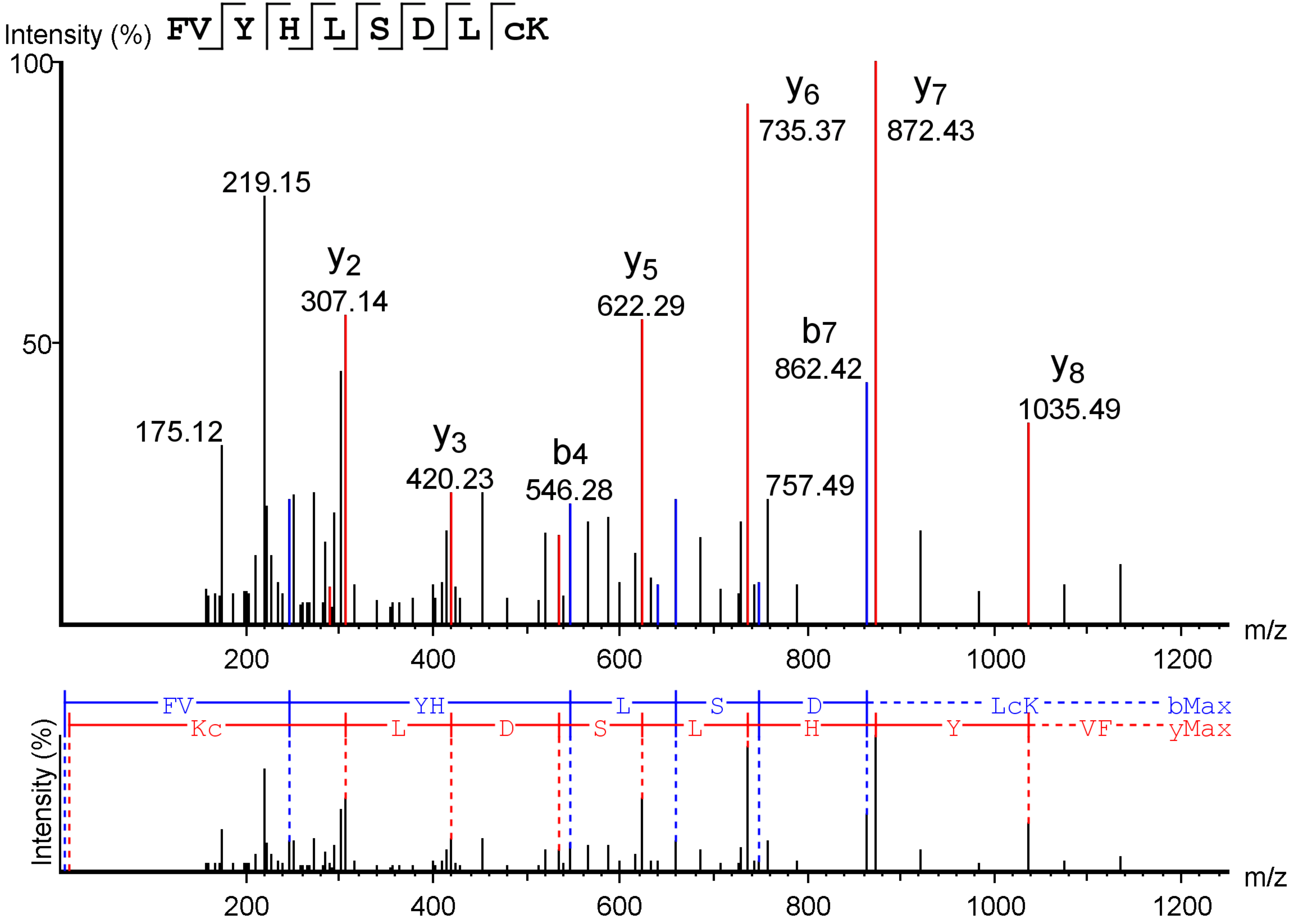

sp|A0A0C4DH31|HV118_HUMAN

R.SDDTAVYYC(+57.02)AR

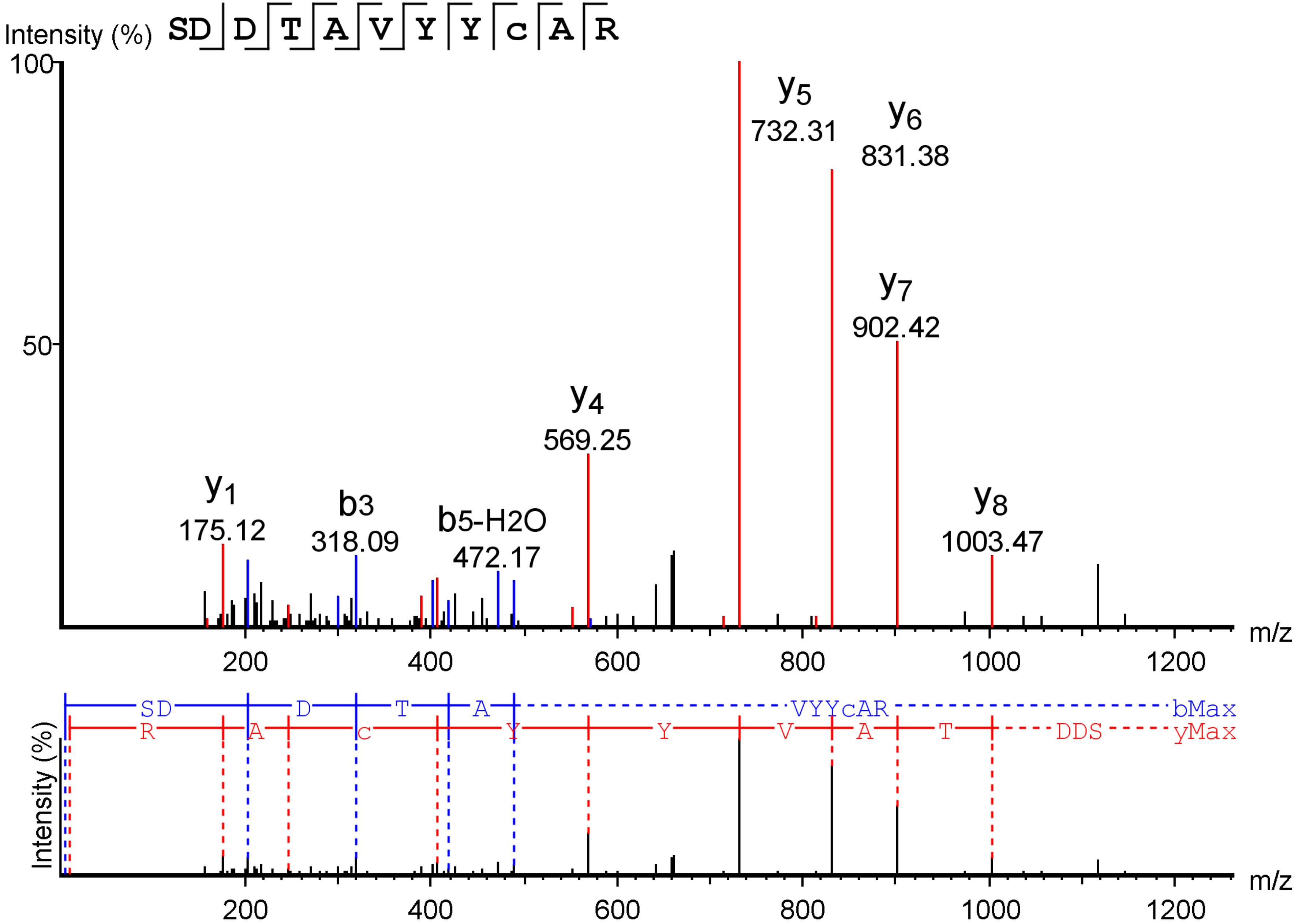

sp|P05204|HMGN2_HUMAN
R.LSAKPAPPKPEPKPK.K

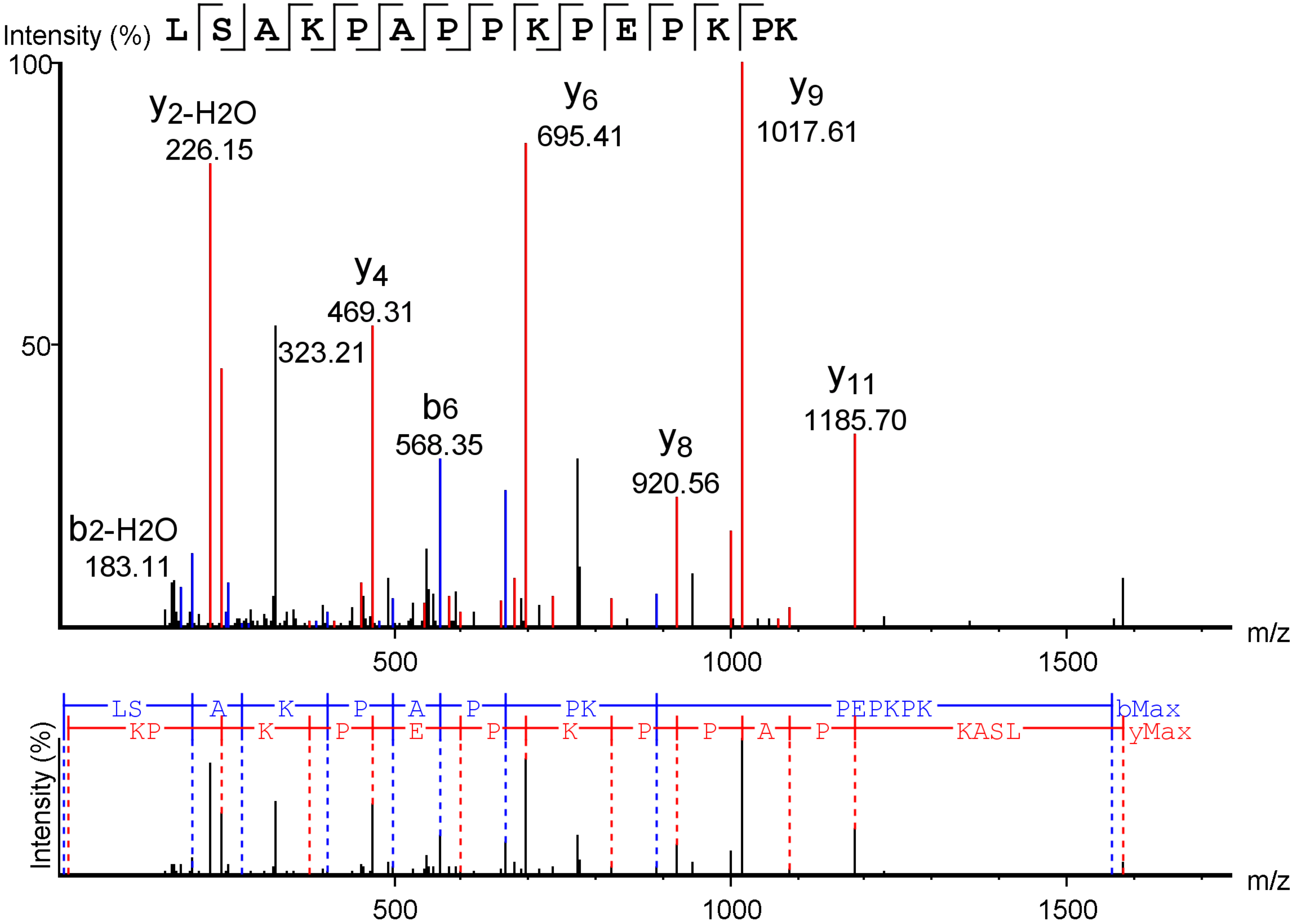

sp|P01594|KV133_HUMAN

K.LLIYDASNLETGVPSR.F

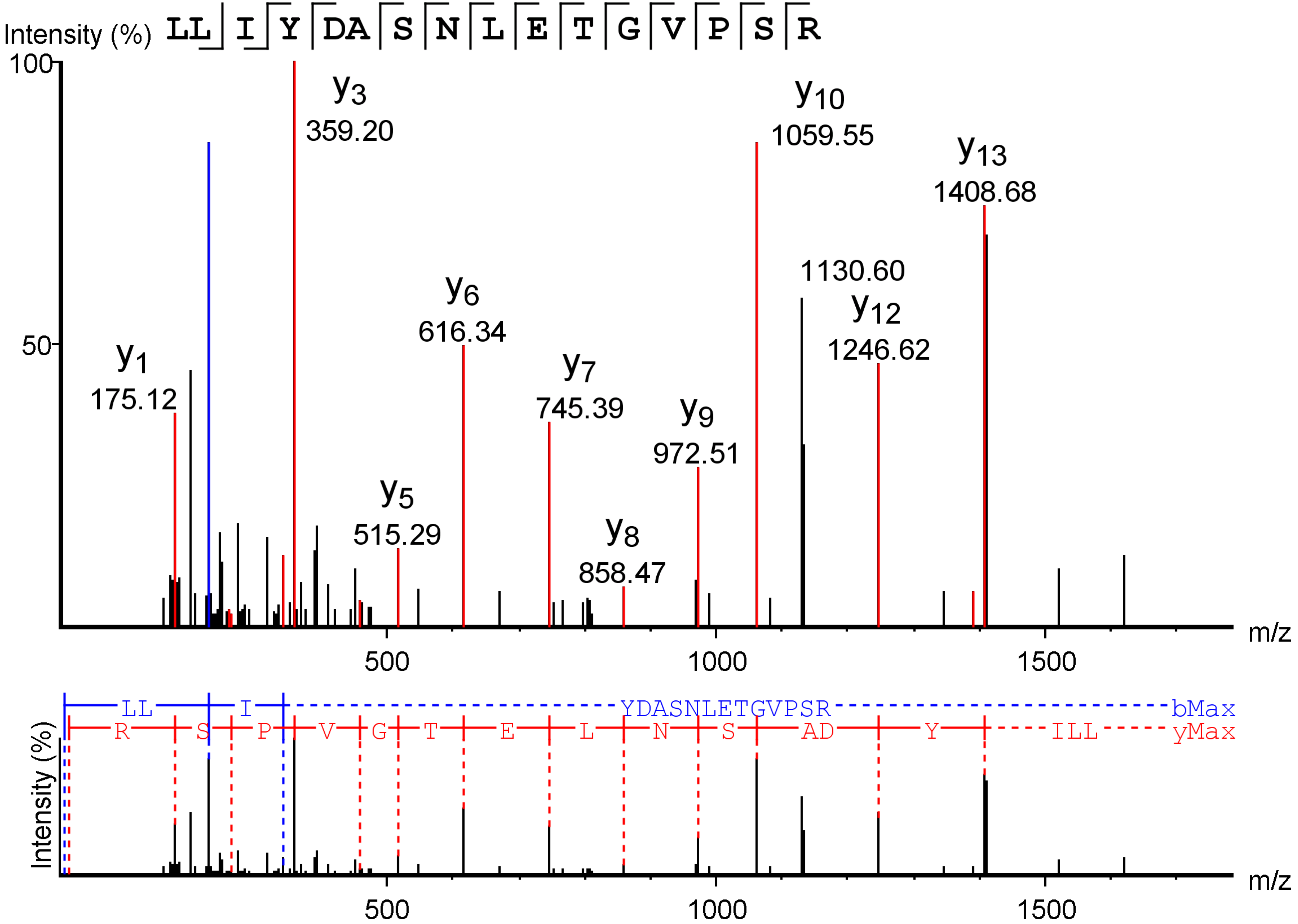

sp|P08621|RU17_HUMAN

R.DPIPYLPPLEK.L

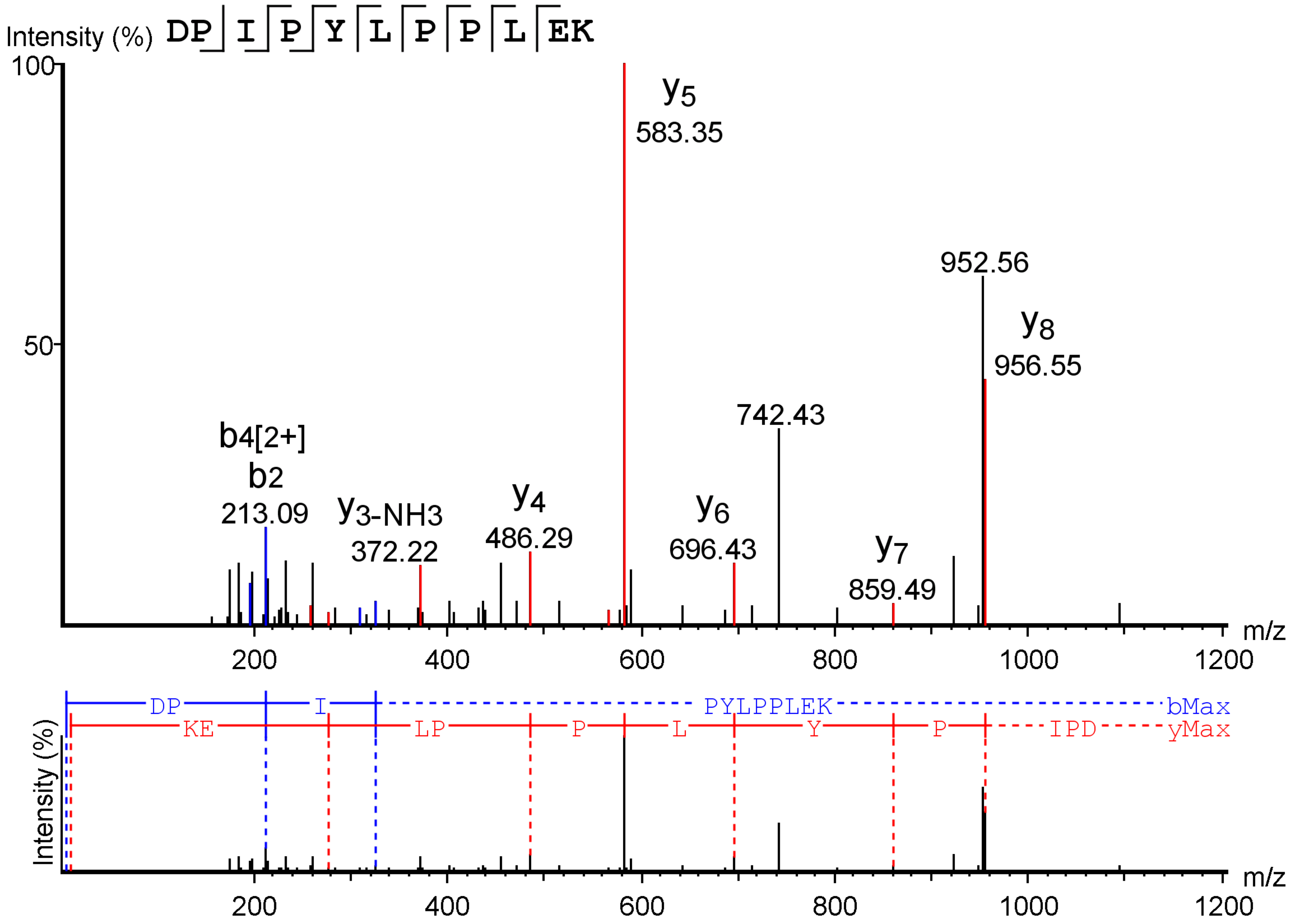

sp|Q8TF72|SHRM3_HUMAN
R.SSPATADKR.Q

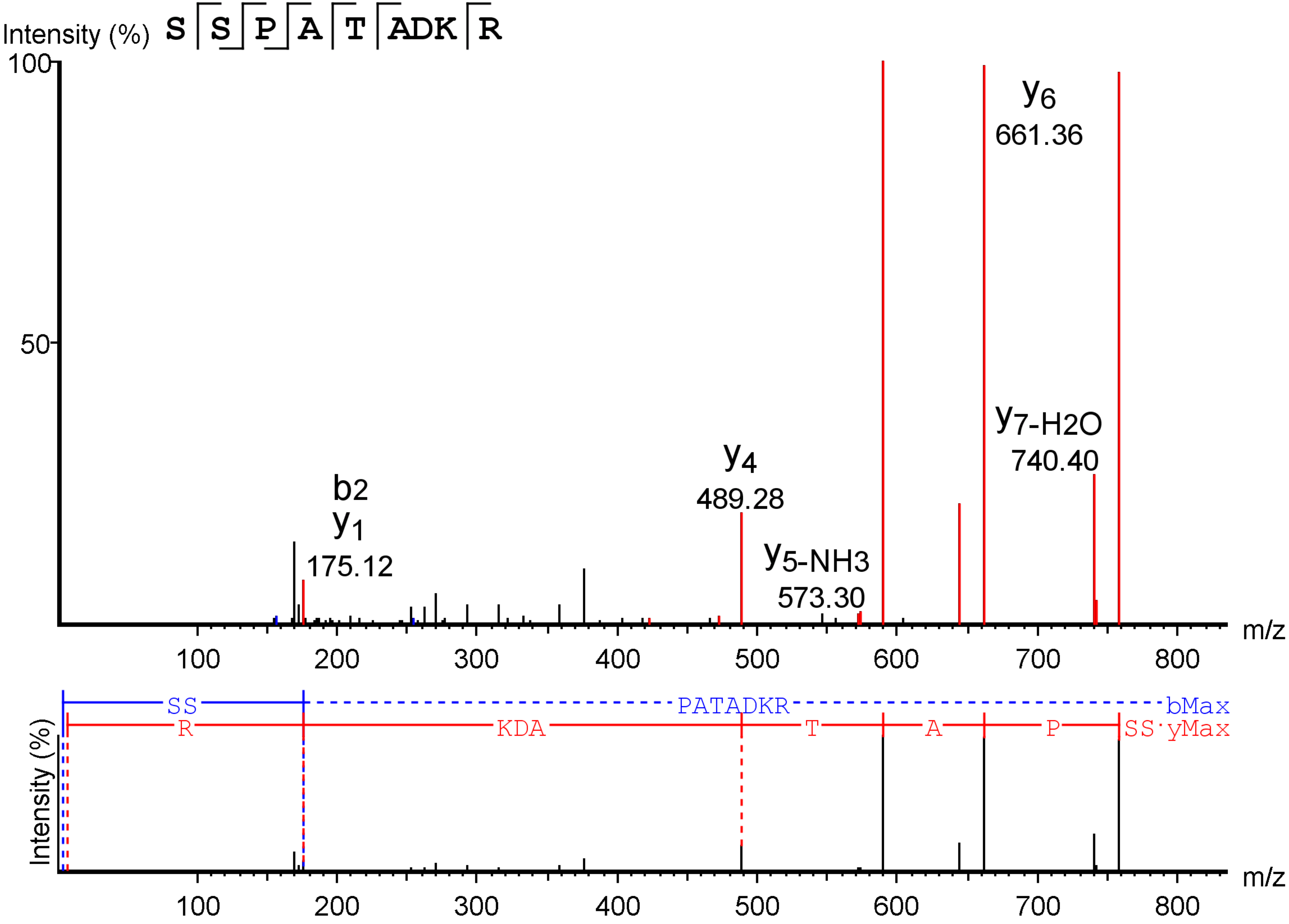

sp|P98172|EFNB1_HUMAN

K.GGSGTAGTEPSDIIIPLR.T

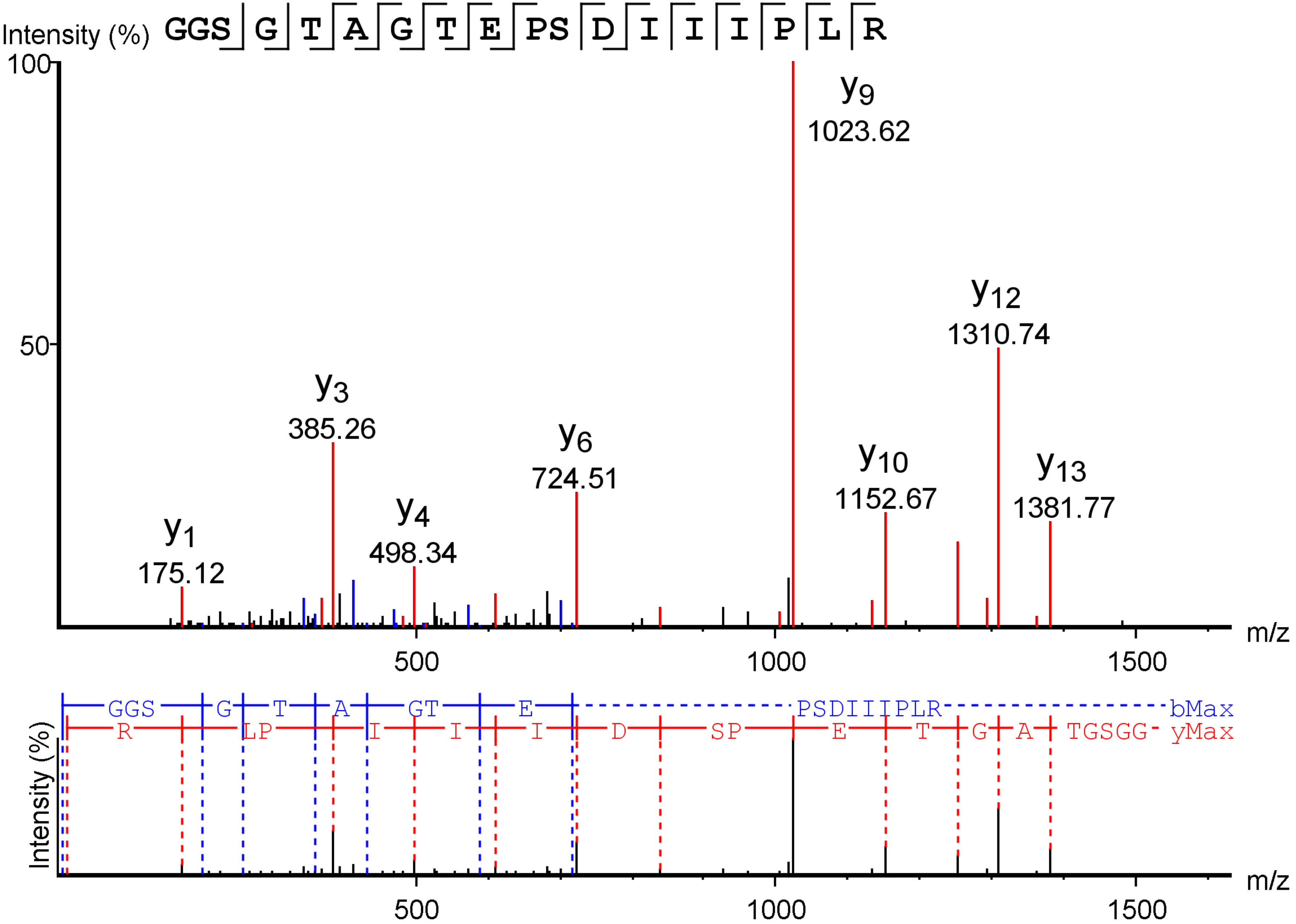

sp|Q12882|DPYD_HUMAN

K.QEYVGGLSTSEIPQFR.L

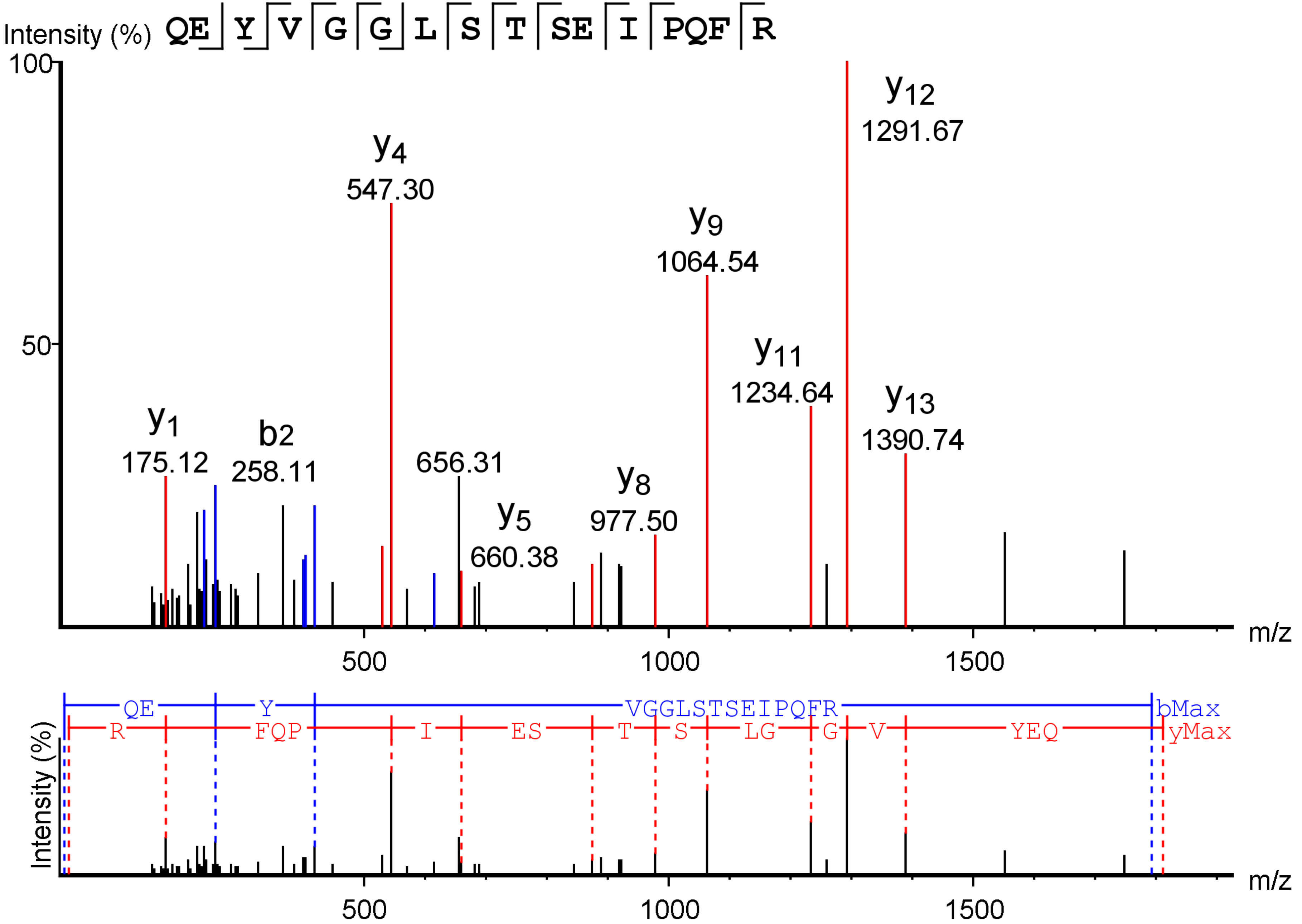

sp|Q6FHJ7|SFRP4_HUMAN

R.SGC(+57.02)NEVTTVVDVK.E

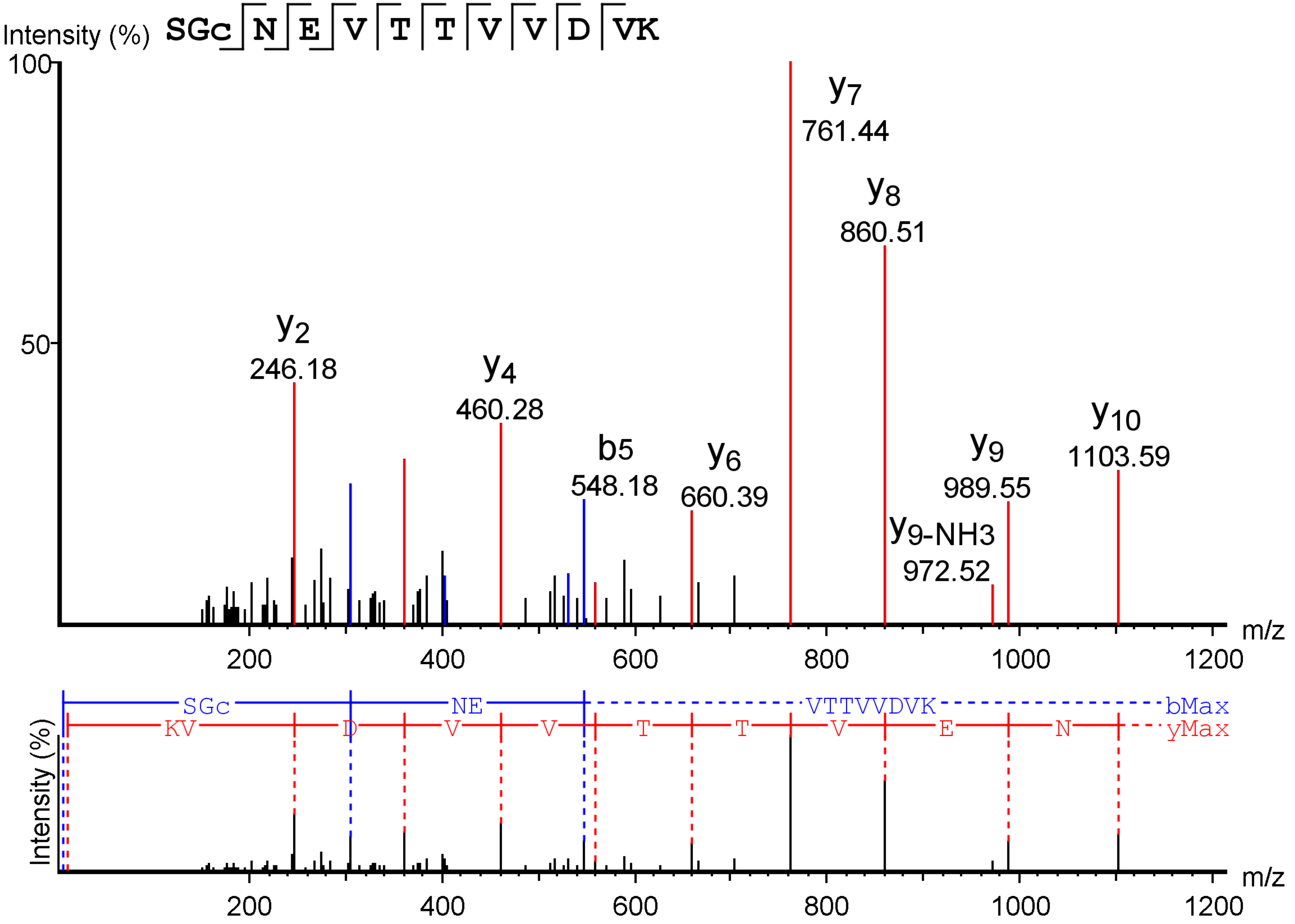

sp|Q15121|PEA15_HUMAN

K.SEEITTGSAWFSFLESHNK.L

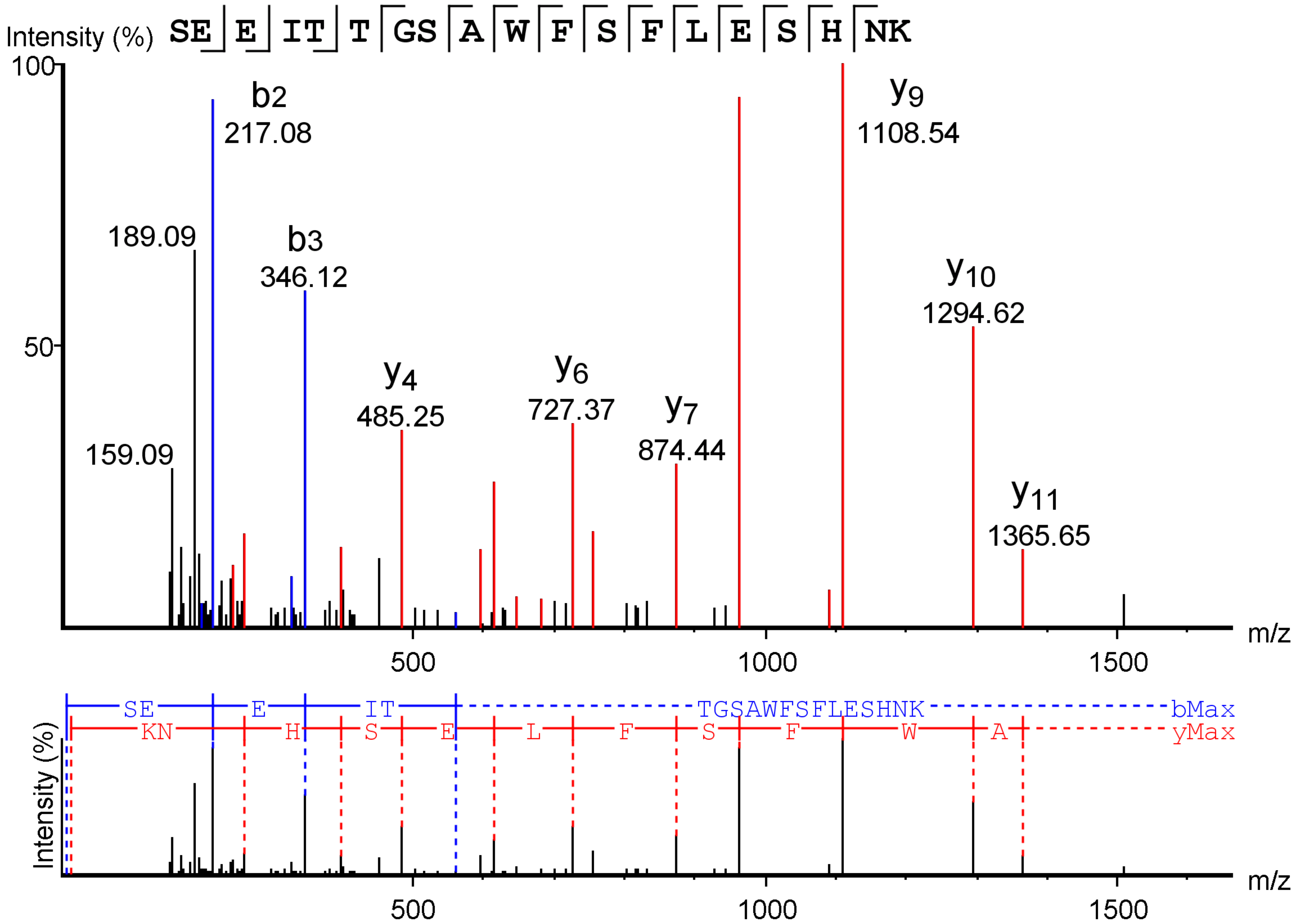

sp|Q9UHY7|ENOPH_HUMAN
K.AEFFADVVPAVR.K

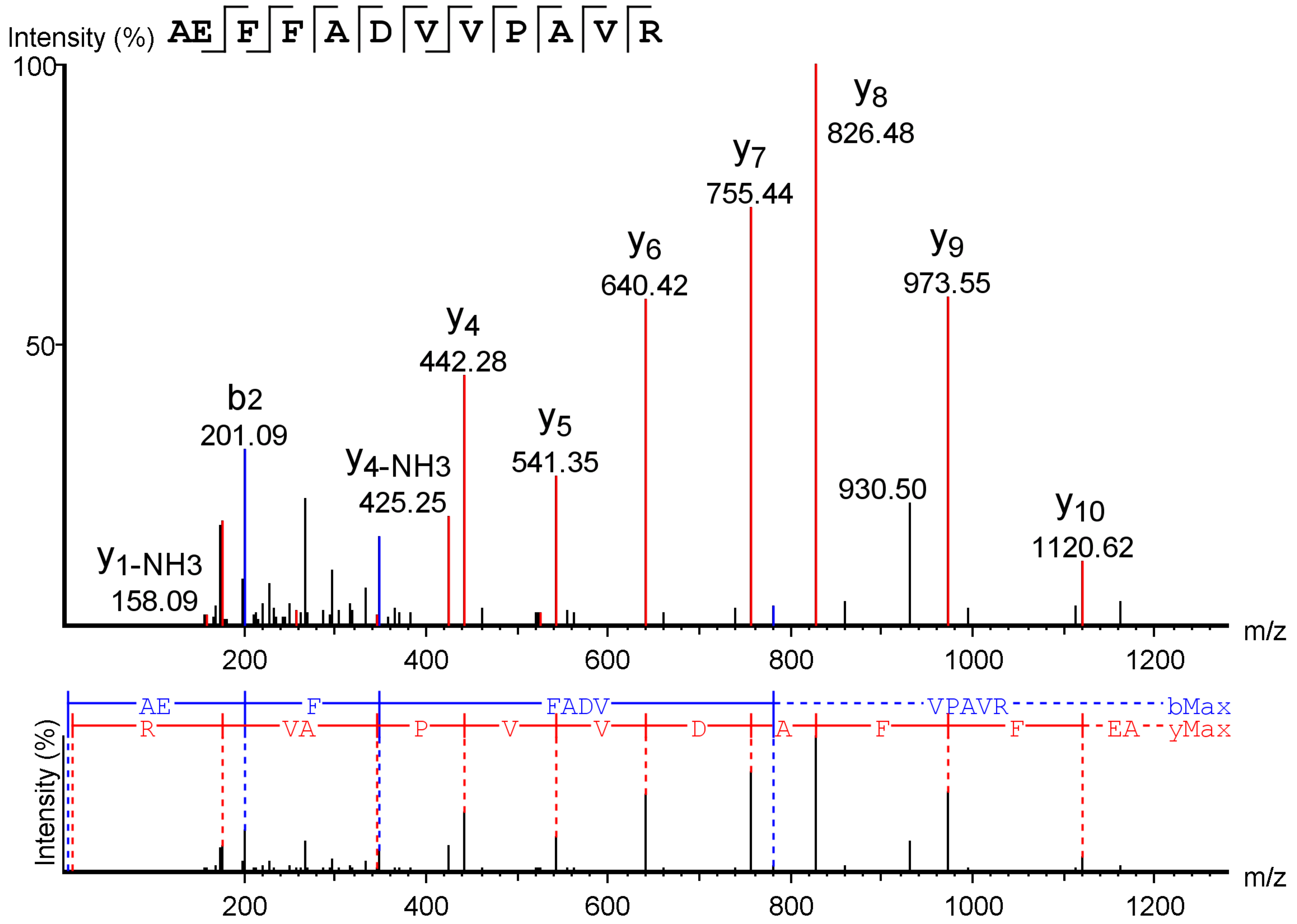

sp|P61019|RAB2A_HUMAN
K.TASNVEEAFINTAK.E

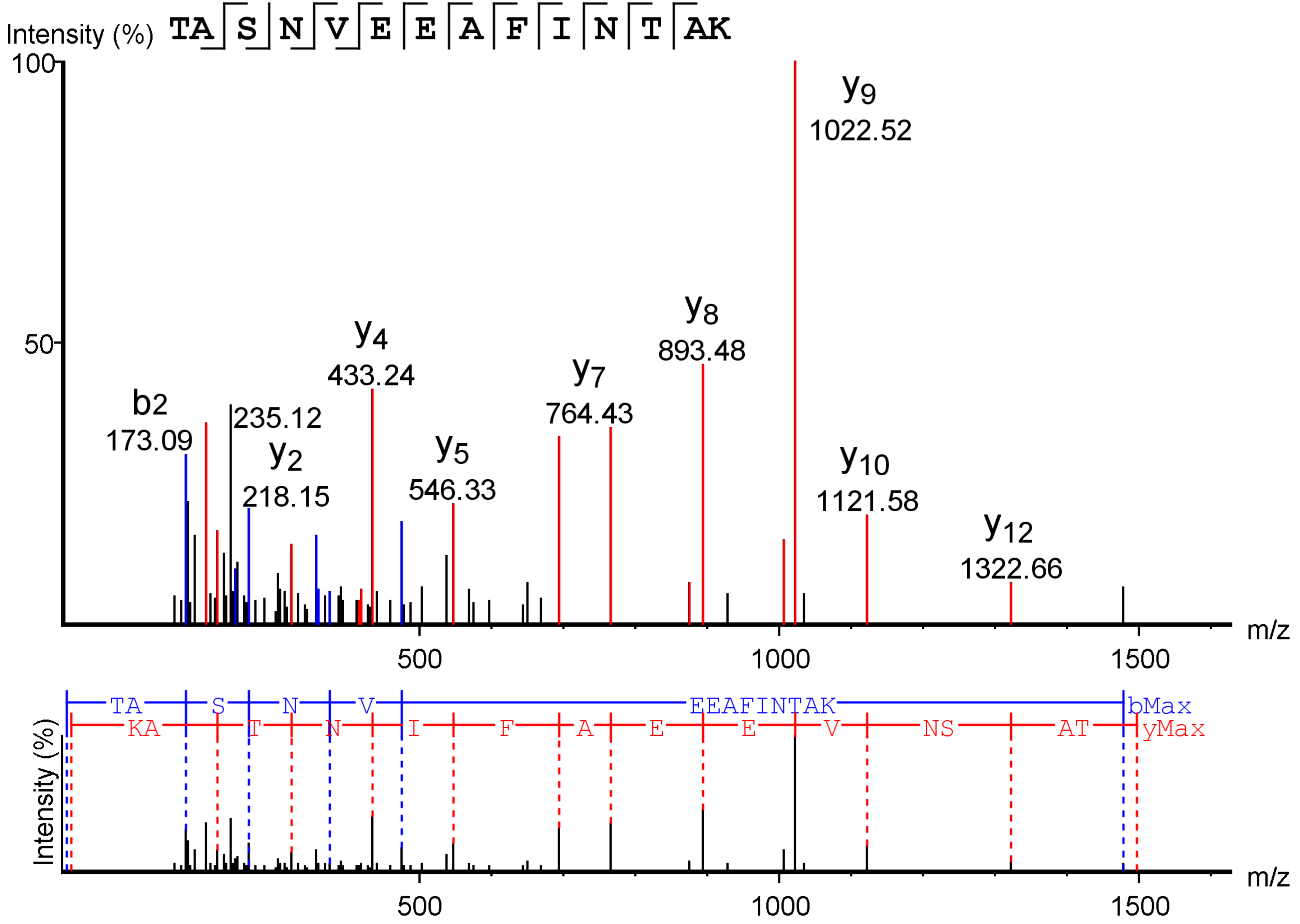

sp|Q8IWA5|CTL2_HUMAN
K.NENKPYLFYFNIVK.C

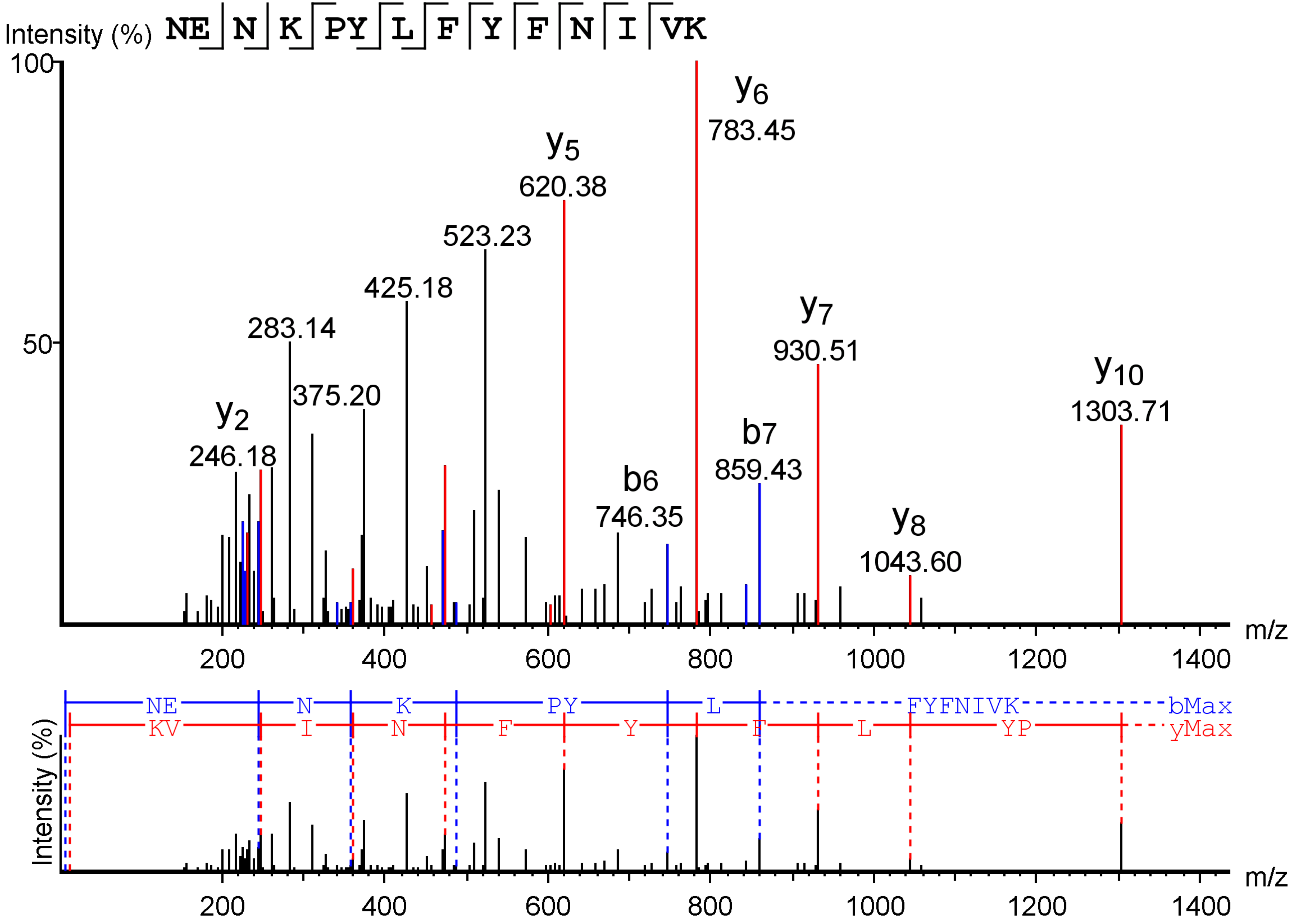

sp|O60701|UGDH_HUMAN

R.VLIGGDETPEGQR.A

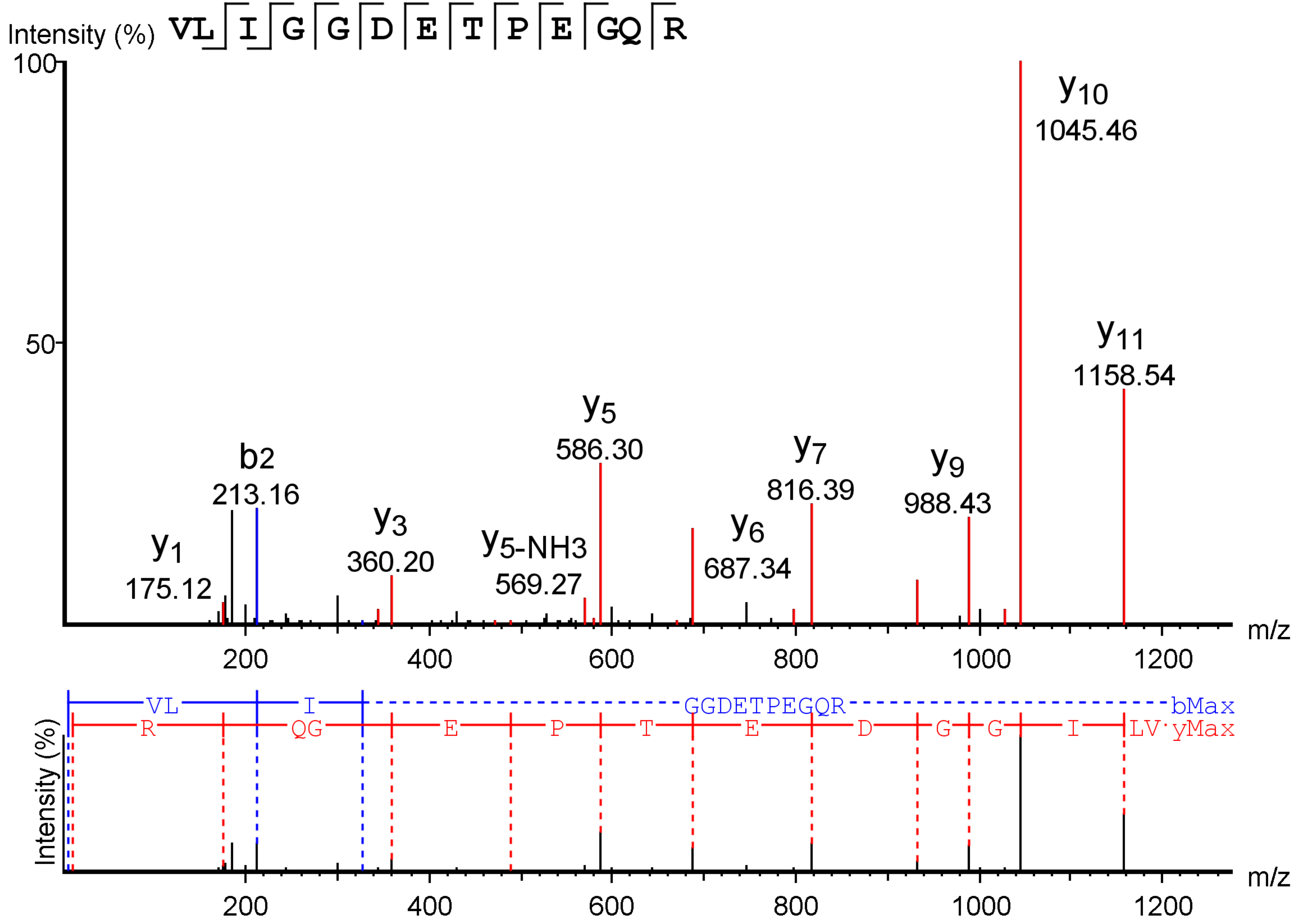

sp|P80748|LV321_HUMAN

R.FSGSNSGNTATLTISR.V

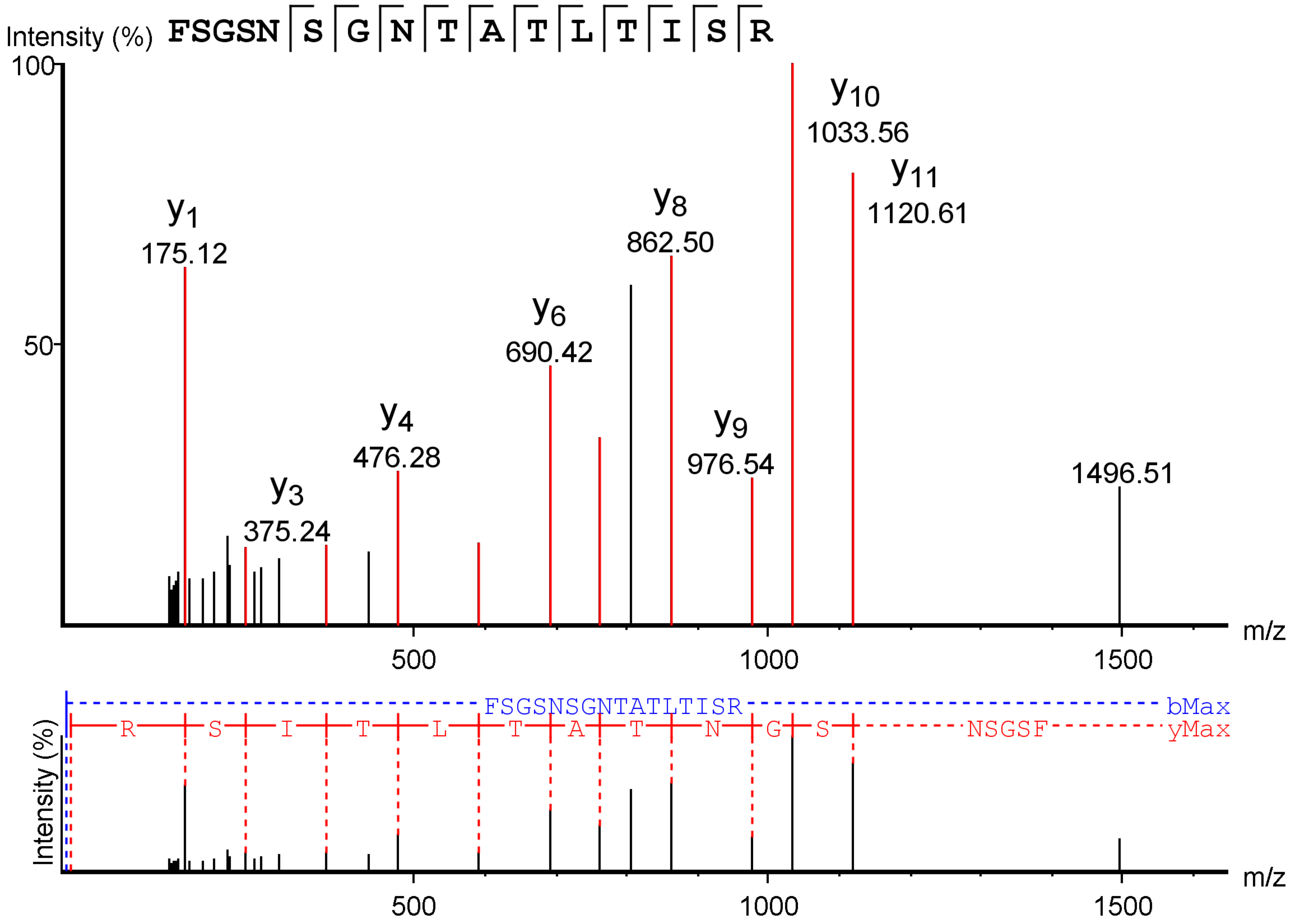

sp|P09497|CLCB_HUMAN

K.VAQLC(+57.02)DFNPK.S

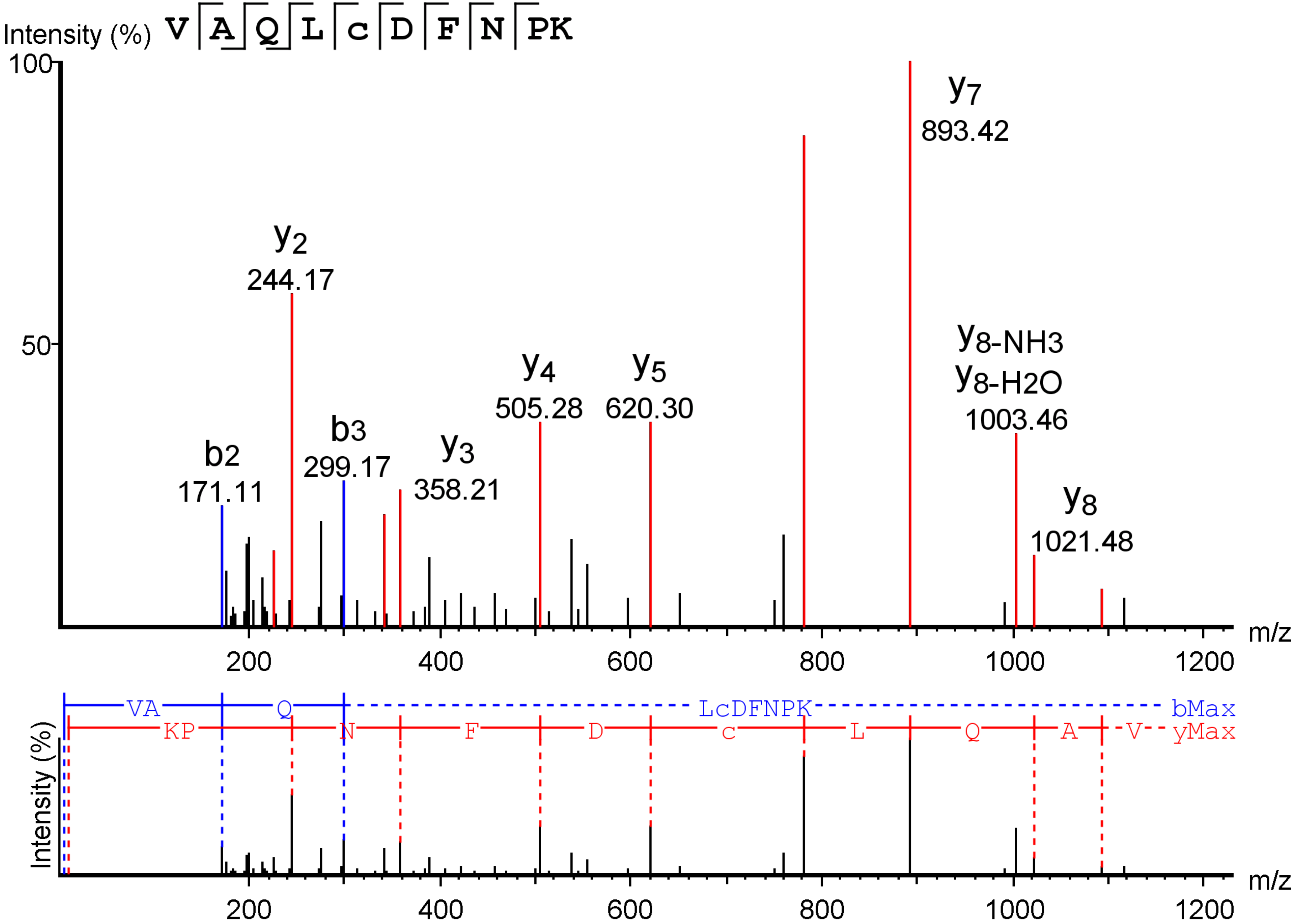

sp|Q9H6R3|ACSS3_HUMAN

R.VLAEHGVAALFTAPTAIR.A

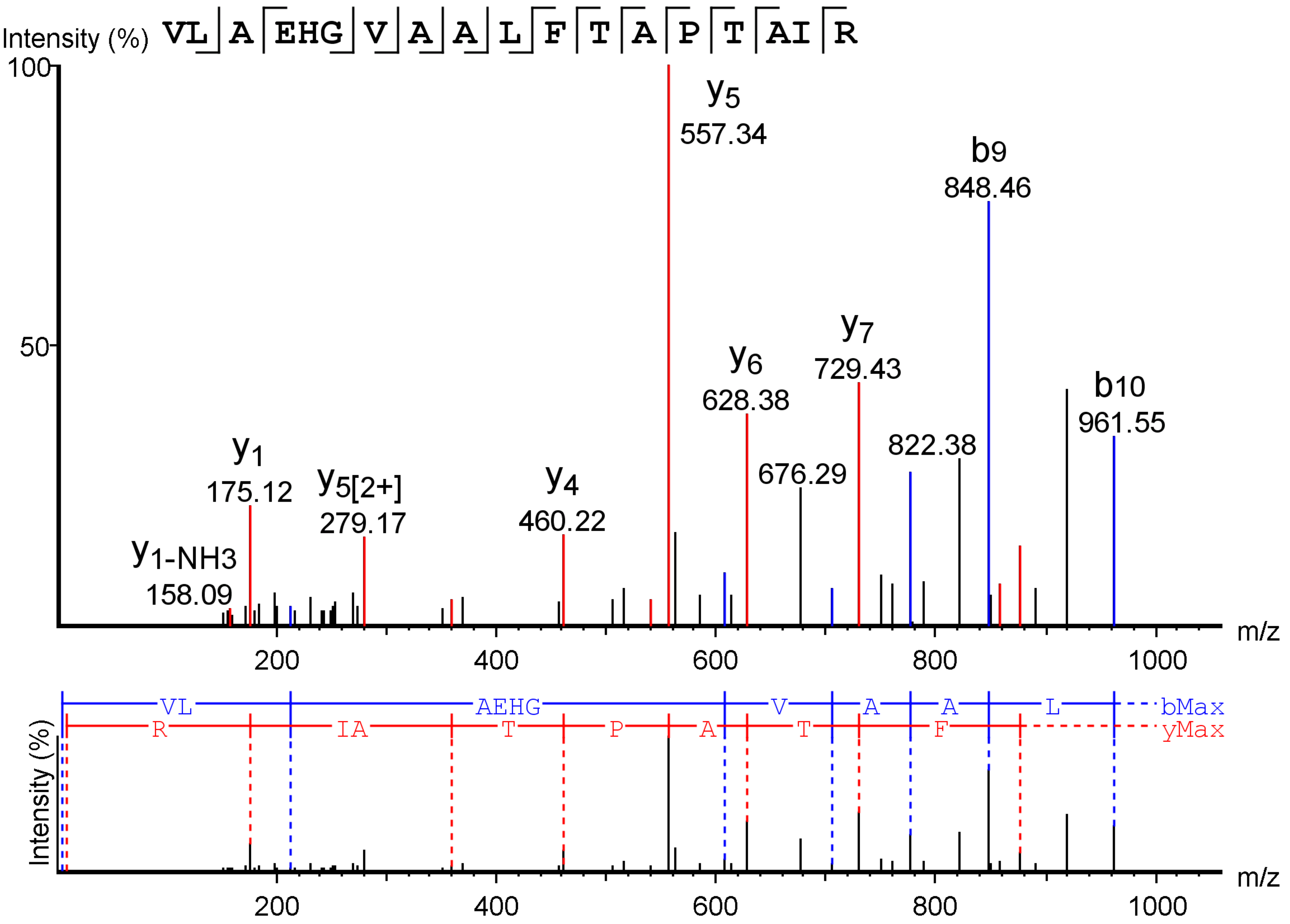

G3V0E5|G3V0E5_HUMAN
K.VSASPLLYTLIEK.T

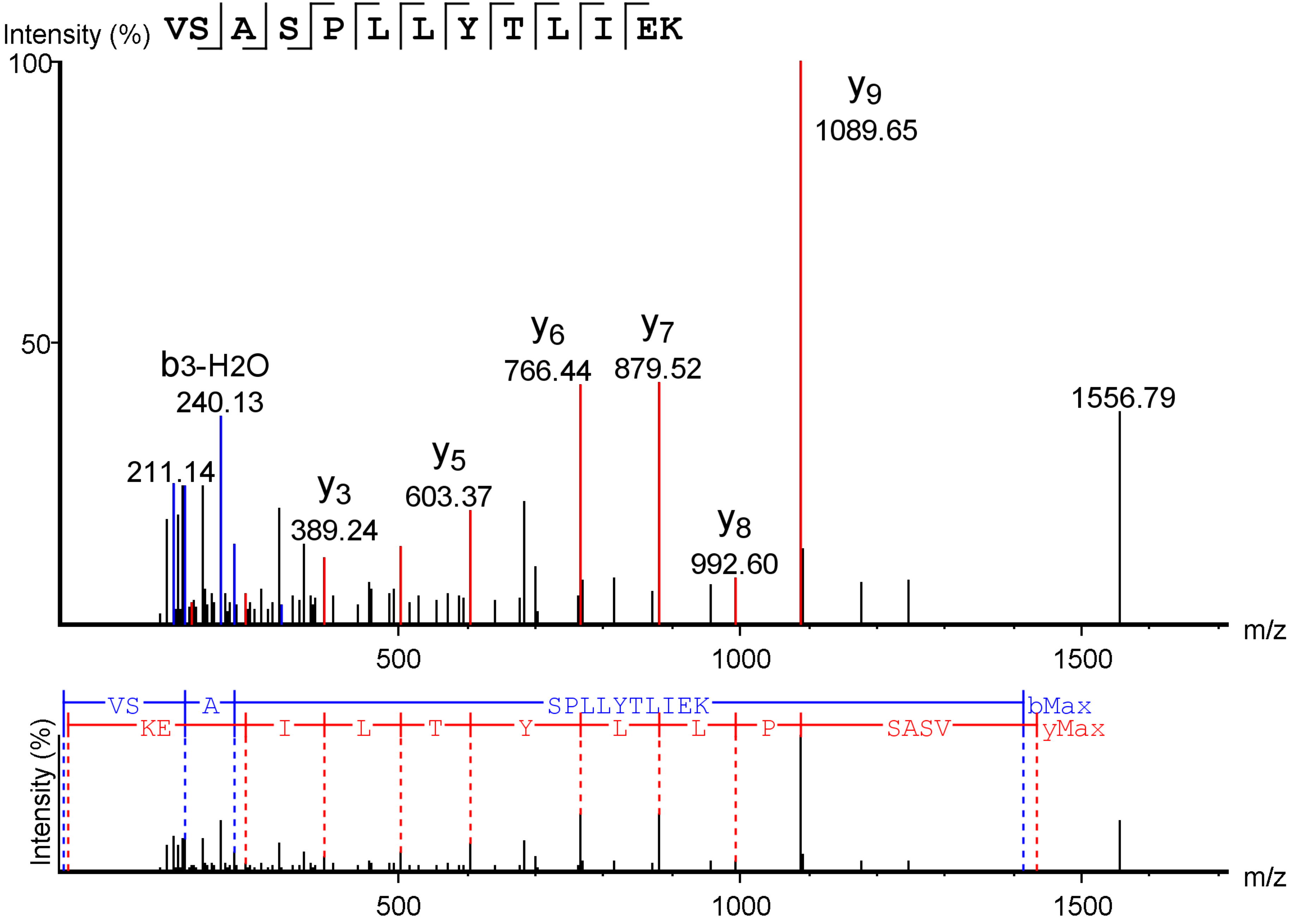

A0A075B7B8|A0A075B7B8_HUMAN

R.VEDTAVYYC(+57.02)AR

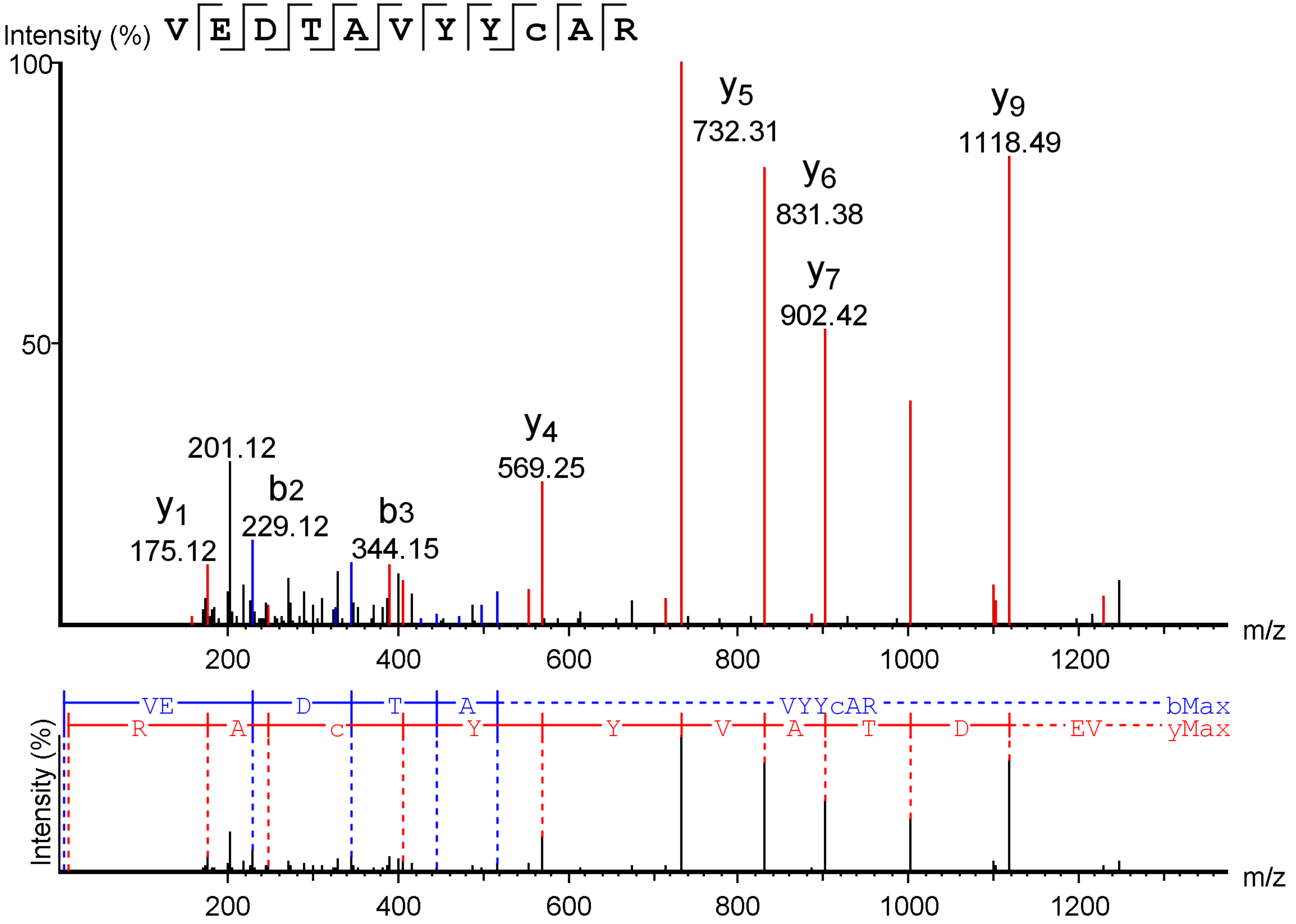

sp|Q9BT88|SYT11_HUMAN
R.NLLVDAAEAGLLSR.D

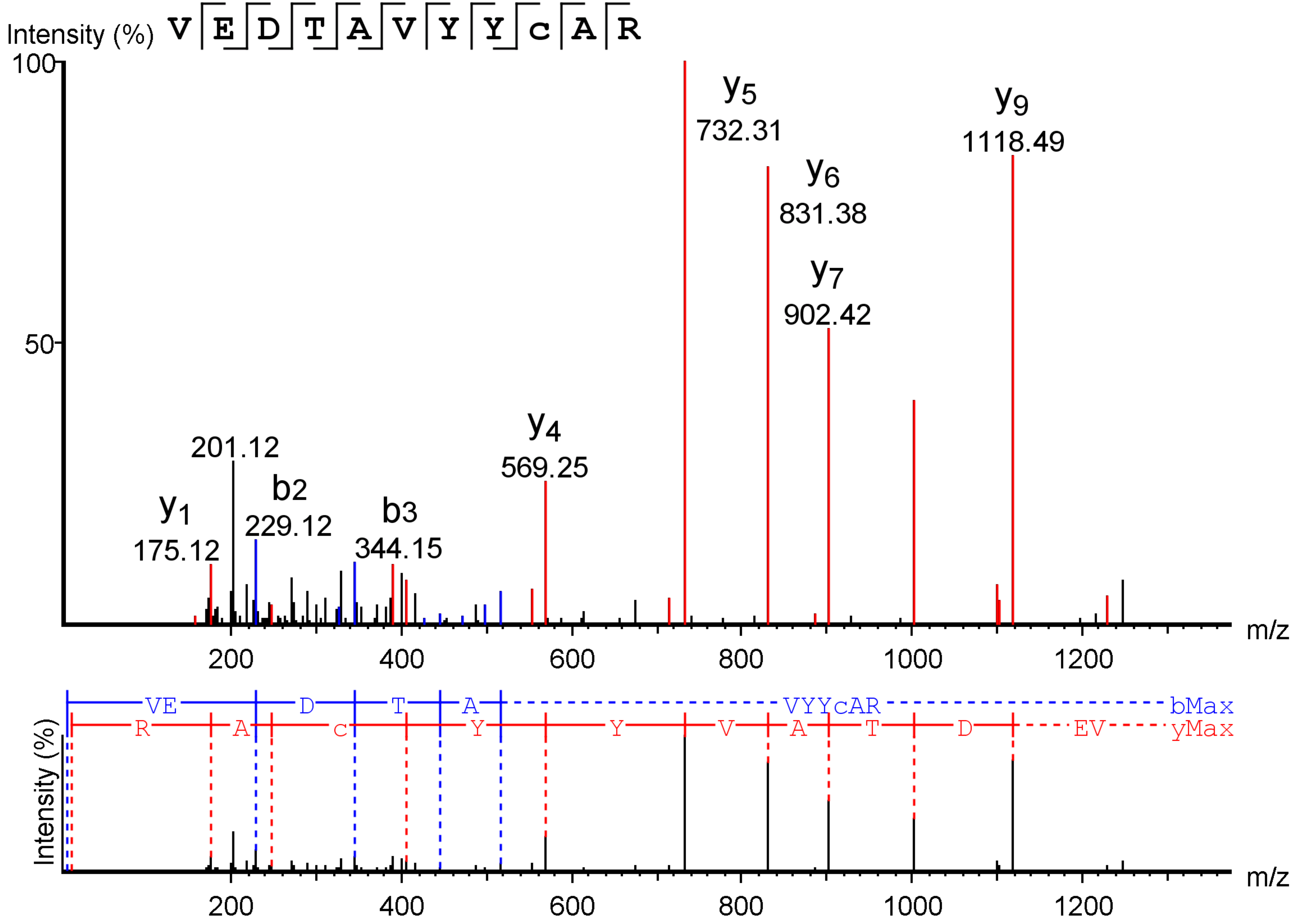

sp|P53999|TCP4_HUMAN

K.GISLNPEQWSQLK.E

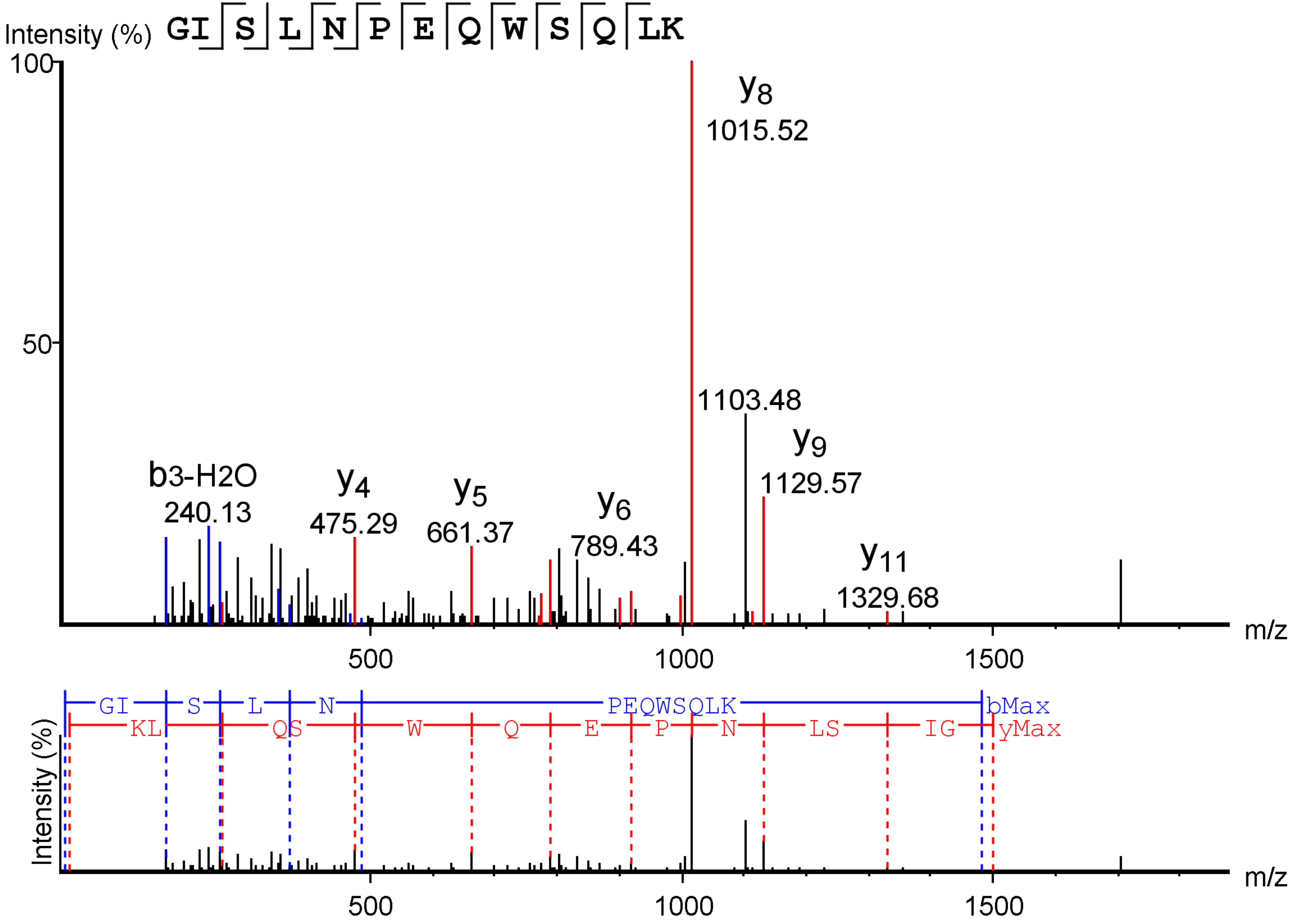

sp|P22061|PIMT_HUMAN

K.ALDVGSGSGILTAC(+57.02)FAR.M

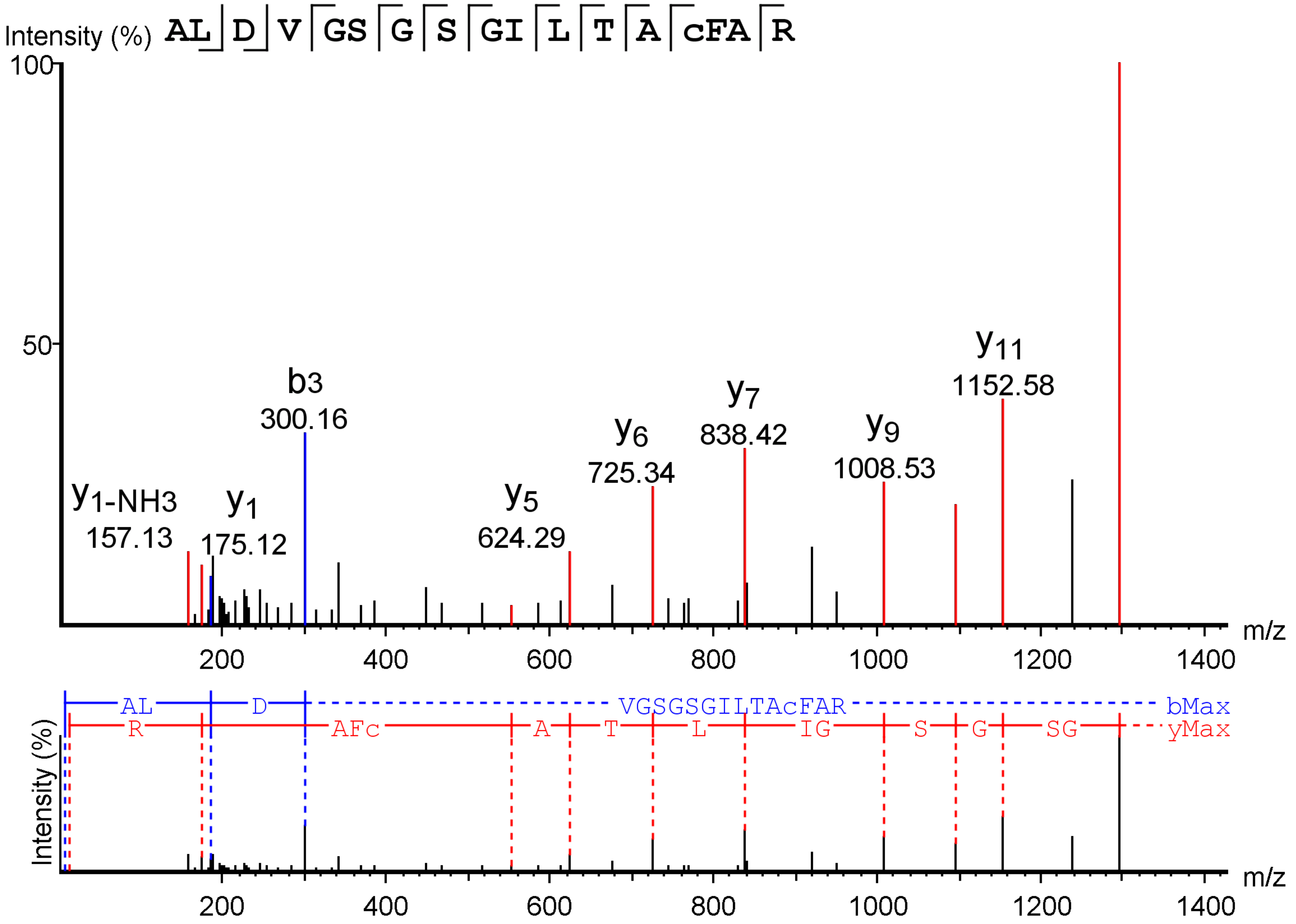

sp|Q14108|SCRB2_HUMAN

K.DEVLYVFPSDFC(+57.02)R.S

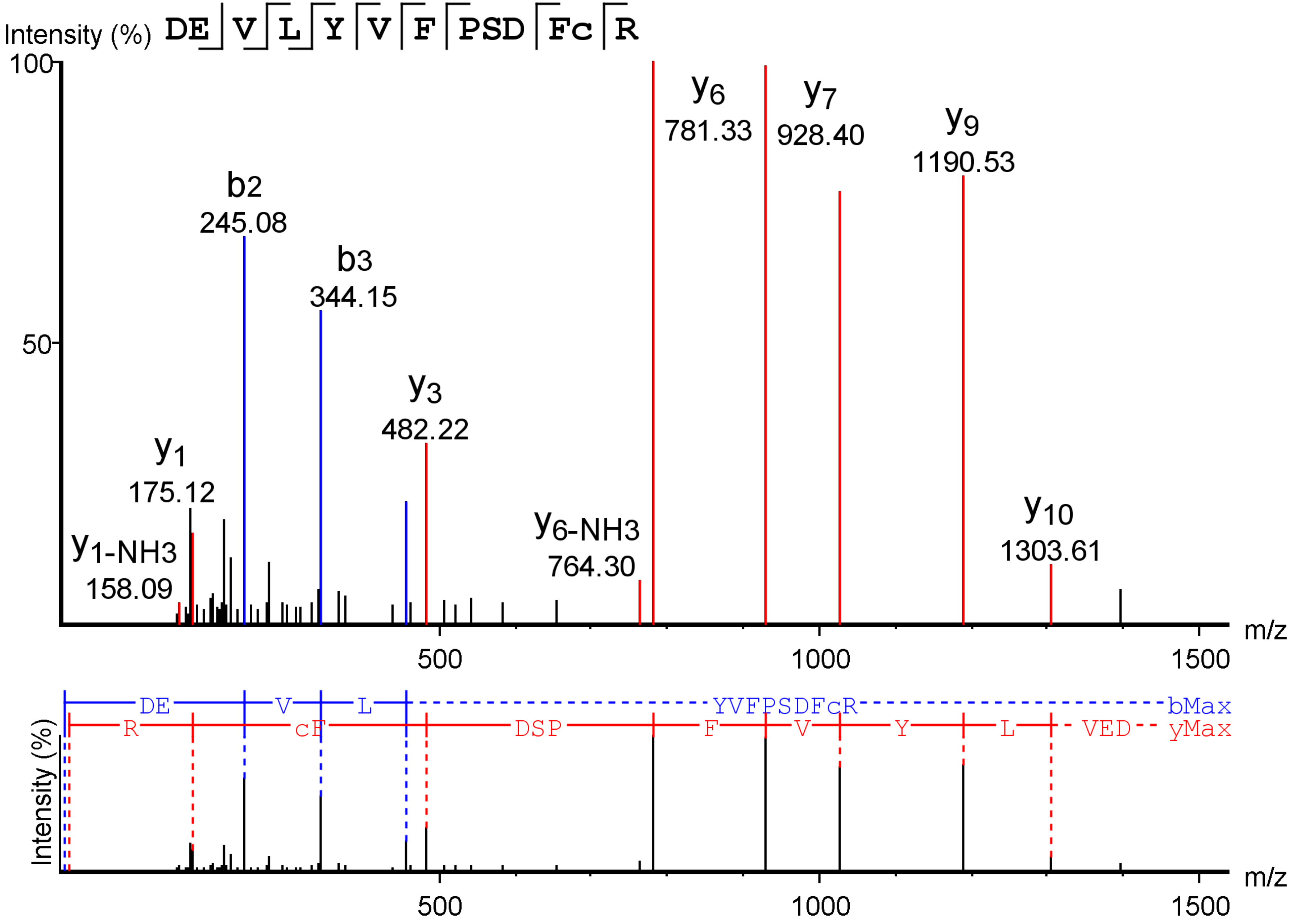

sp|Q00688|FKBP3_HUMAN
K.SEETLDEGPPK.Y

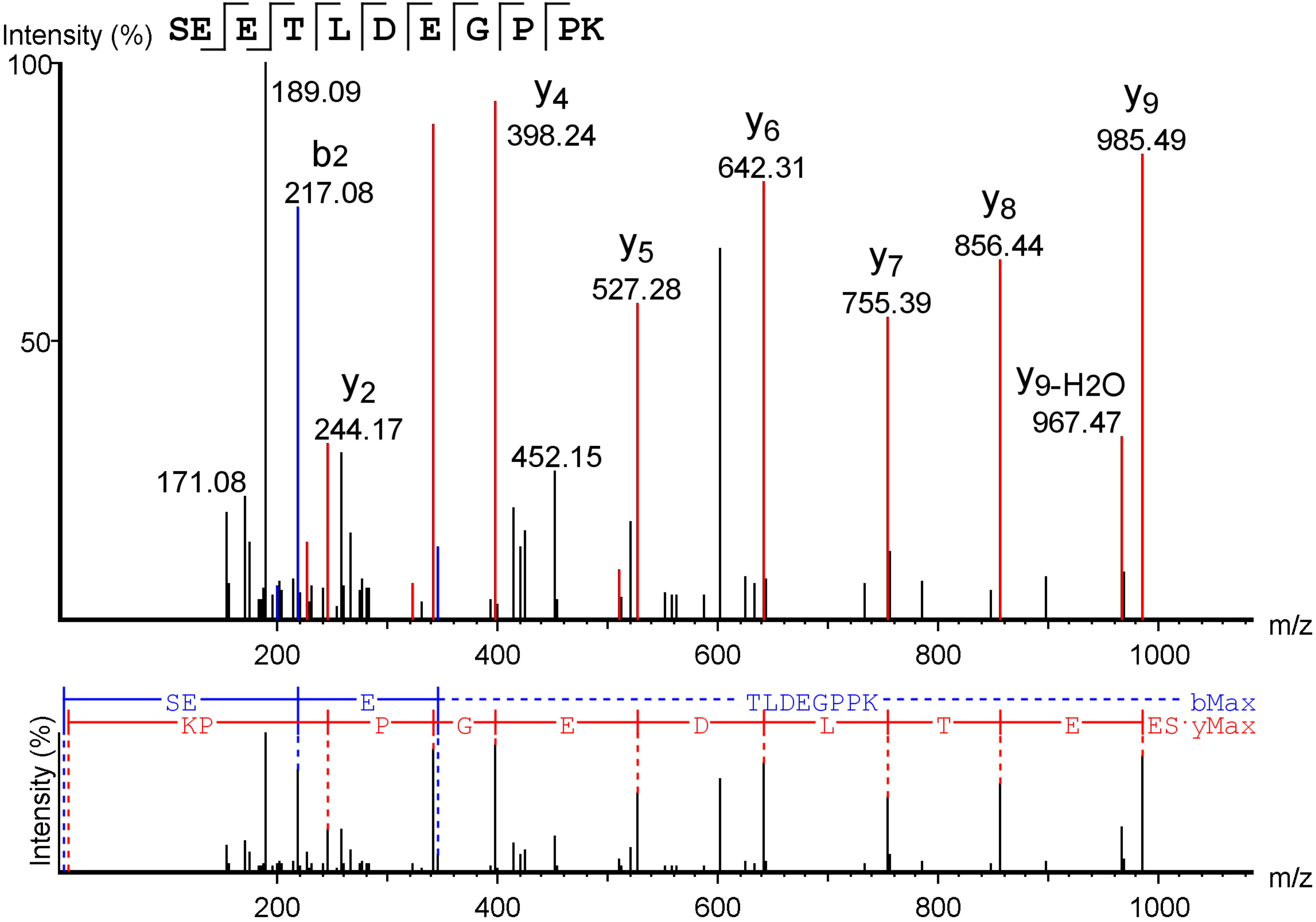

sp|Q9H9H4|VP37B_HUMAN
R.SLAEGNLLYQPQLDTLK.A

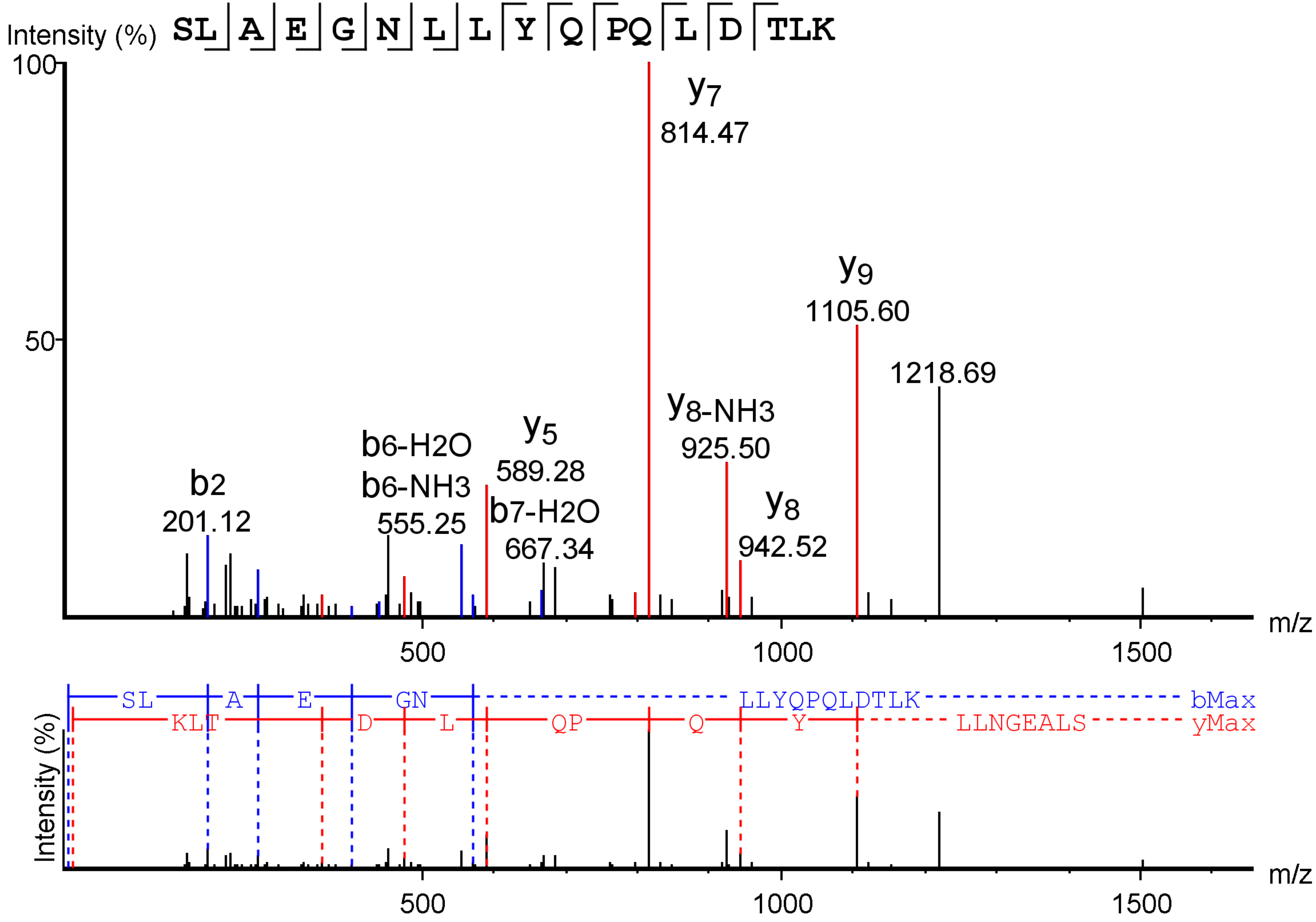

sp|P62266|RS23_HUMAN
K.ANPFGGASHAK.G

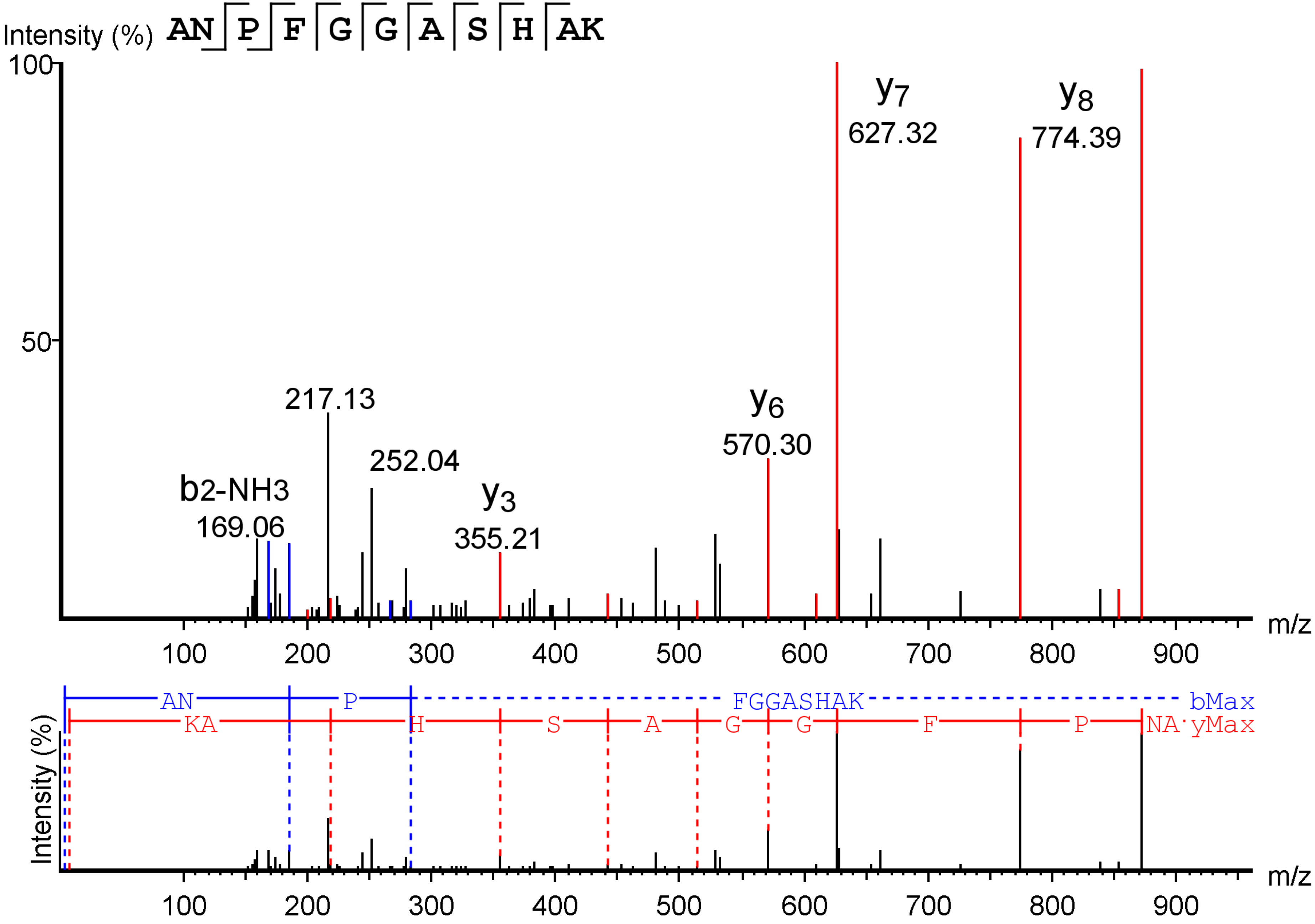

sp|Q9Y3E1|HDGR3_HUMAN
K.GFNEGLWEIENNPGVK.F

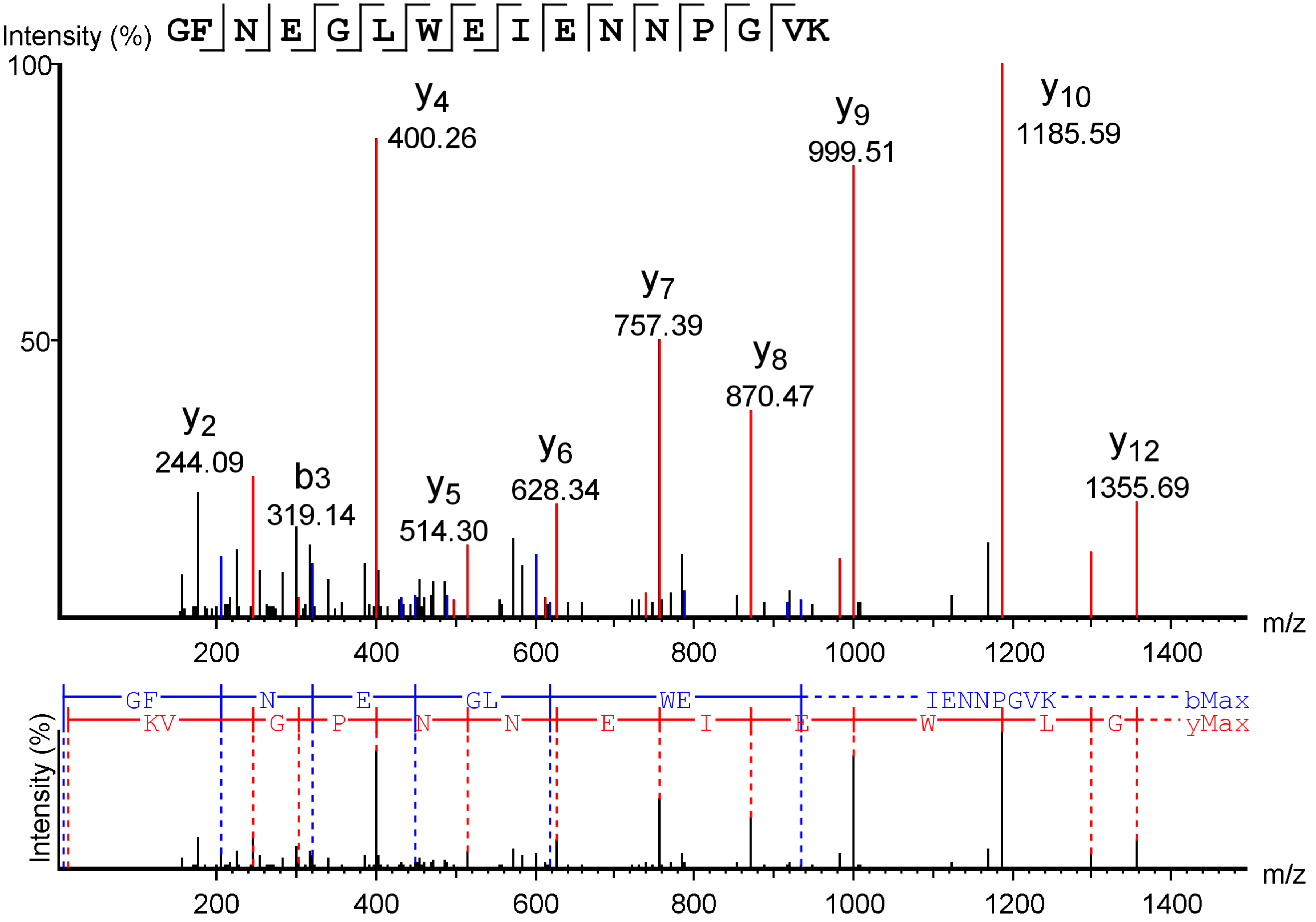

sp|P17096|HMGA1_HUMAN
R.KQPPVSPGTALVGSQKEPSEVPTPK.R

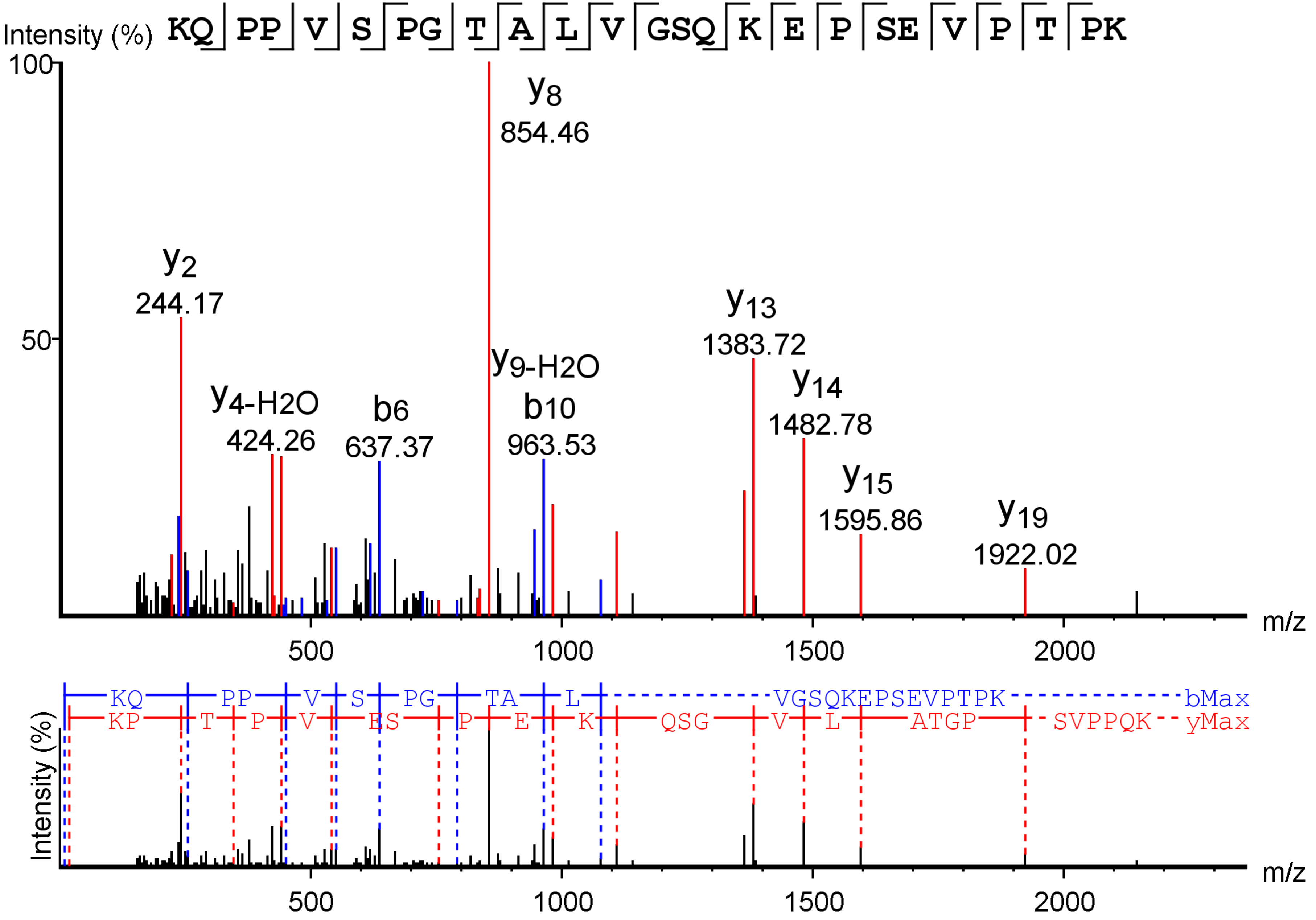

sp|P02792|FRIL_HUMAN
K.LNQALLDLHALGSAR.T

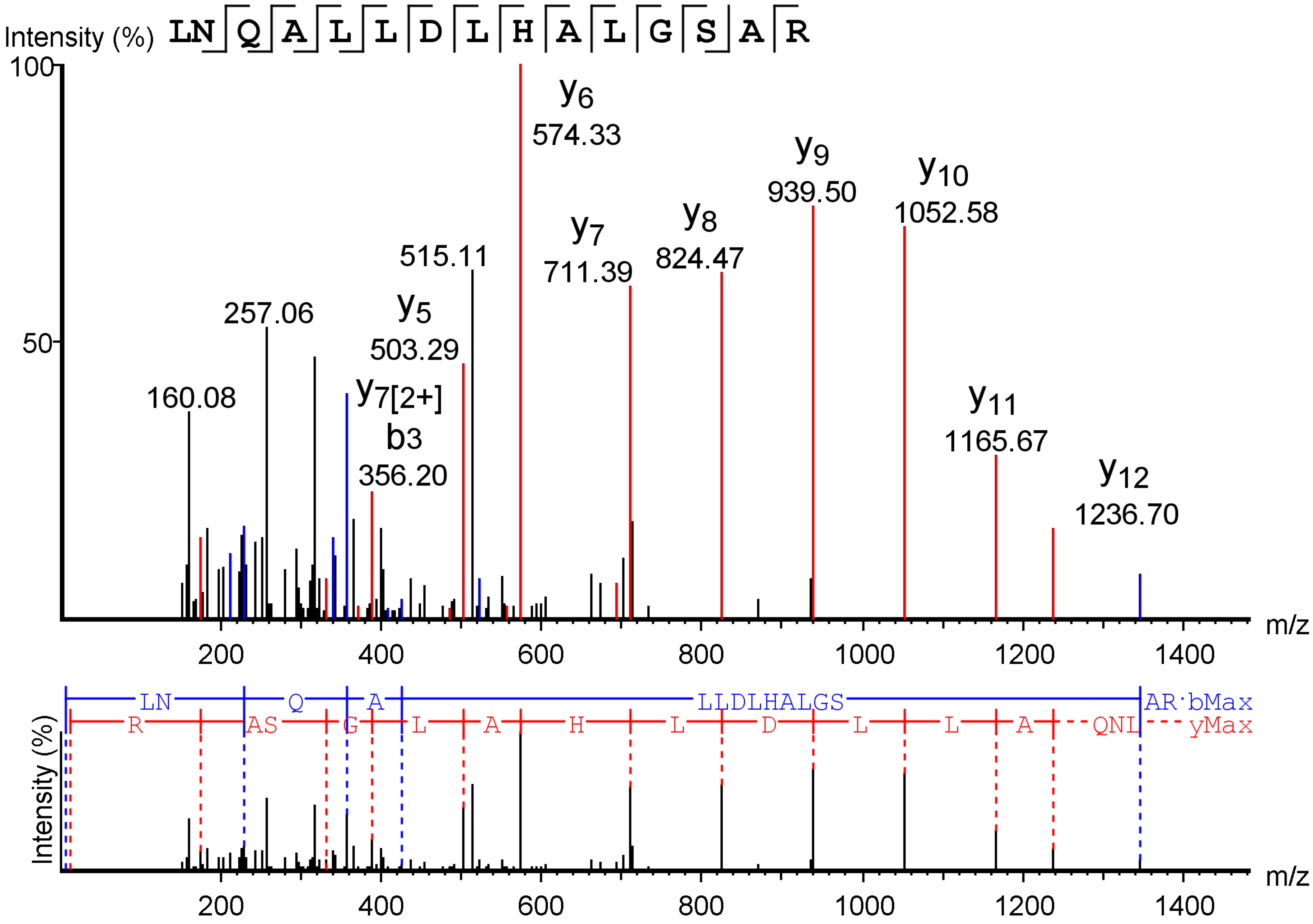

sp|P06576|ATPB_HUMAN
K.VLDSGAPIKIPVGPETLGR.I

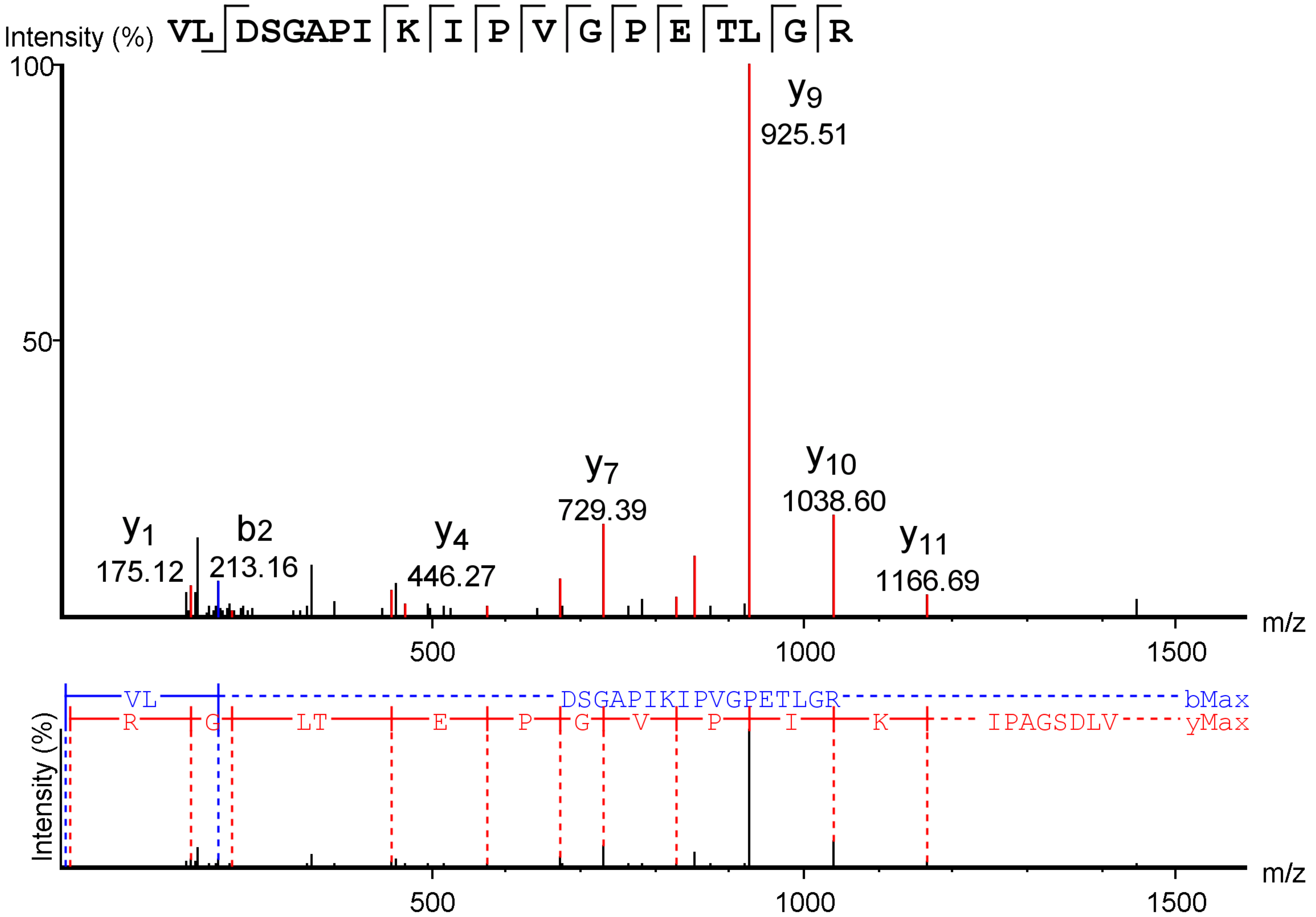

sp|O75475|PSIP1_HUMAN
R.VDEVPDGAVKPPTNK.L

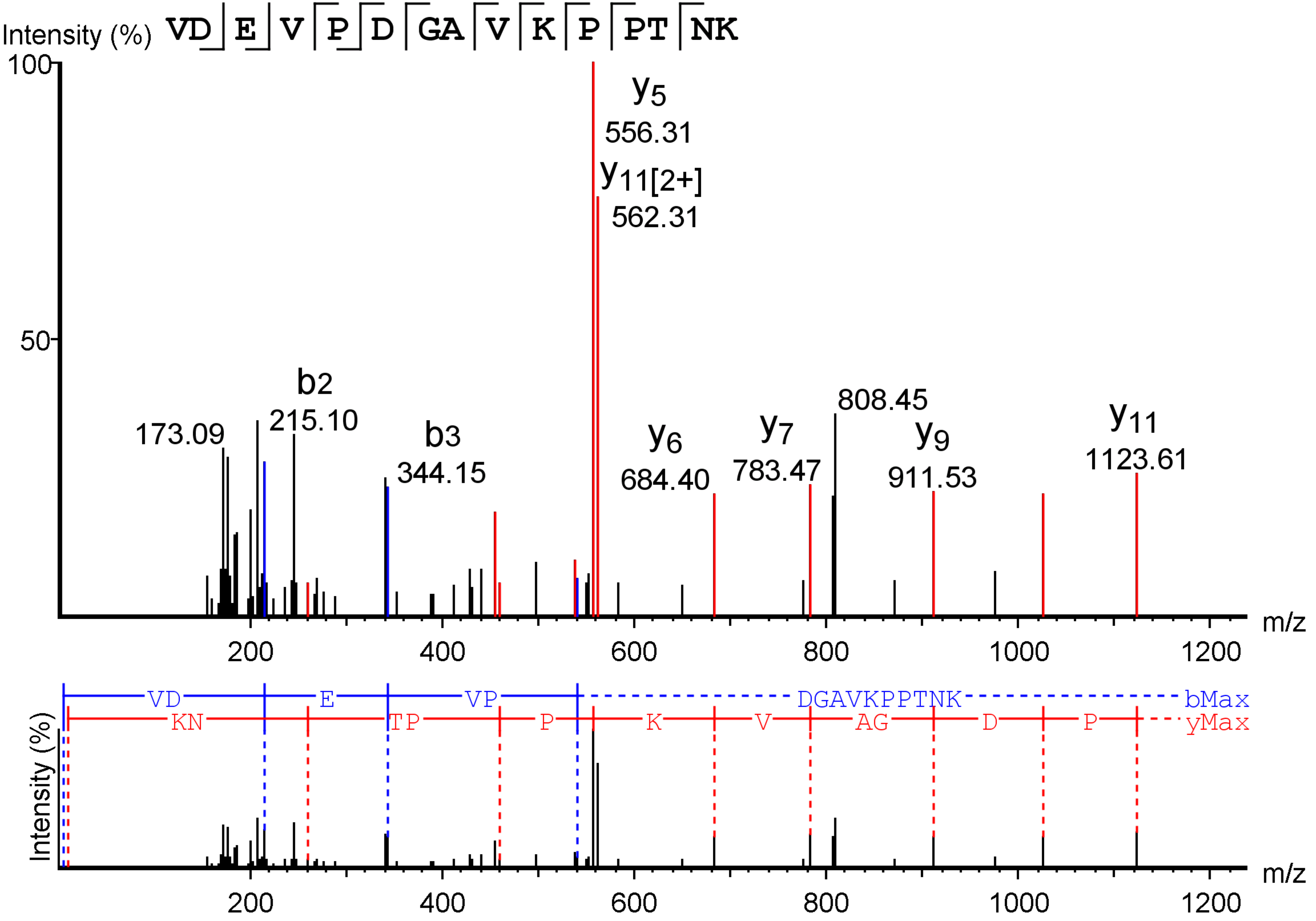

sp|P09382|LEG1_HUMAN

M.A(+42.01)C(+57.02)GLVASNLNLKPGEC(+57.02)LR.V

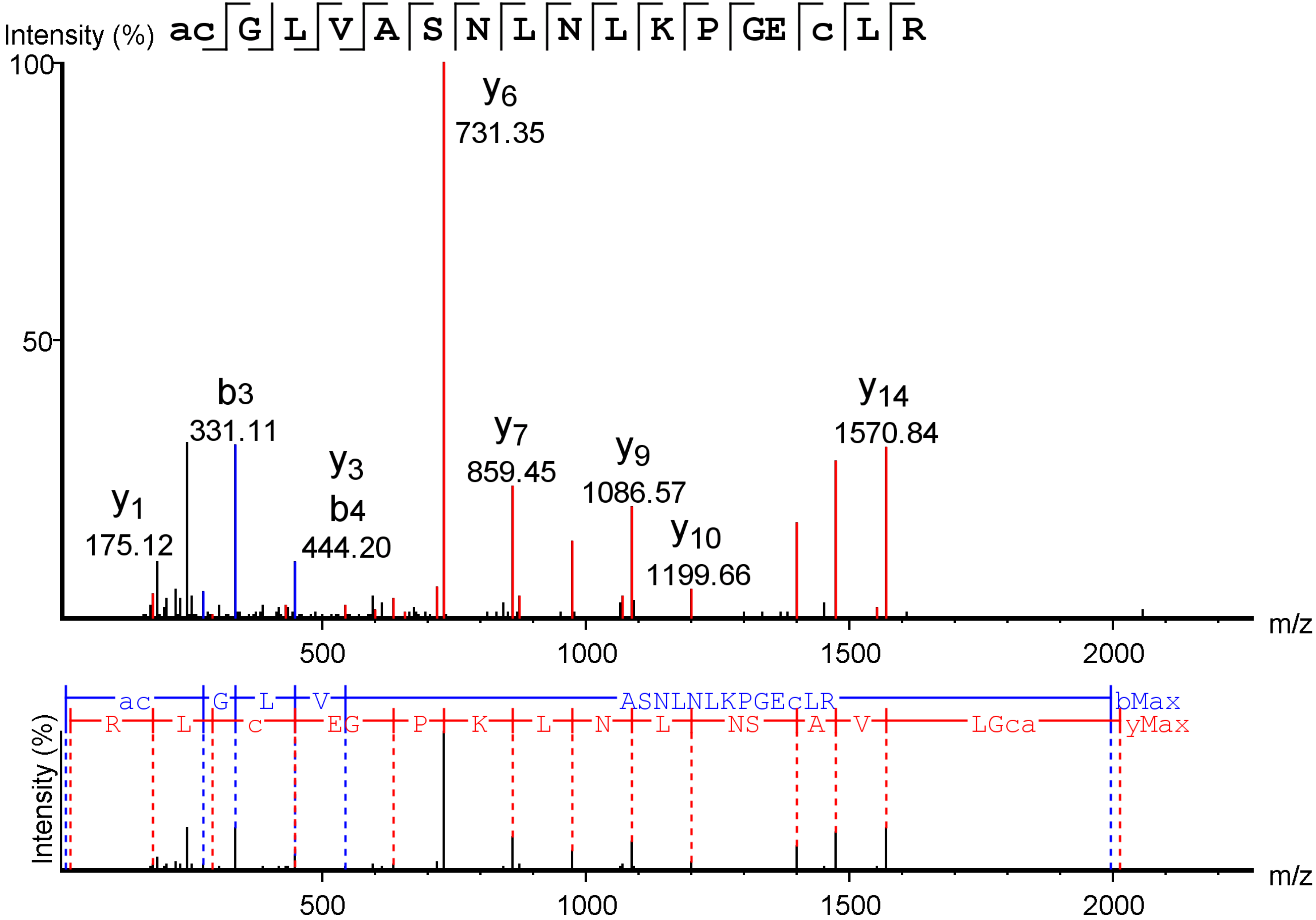

sp|P54578|UBP14_HUMAN
K.VSIVTPEDILR.L

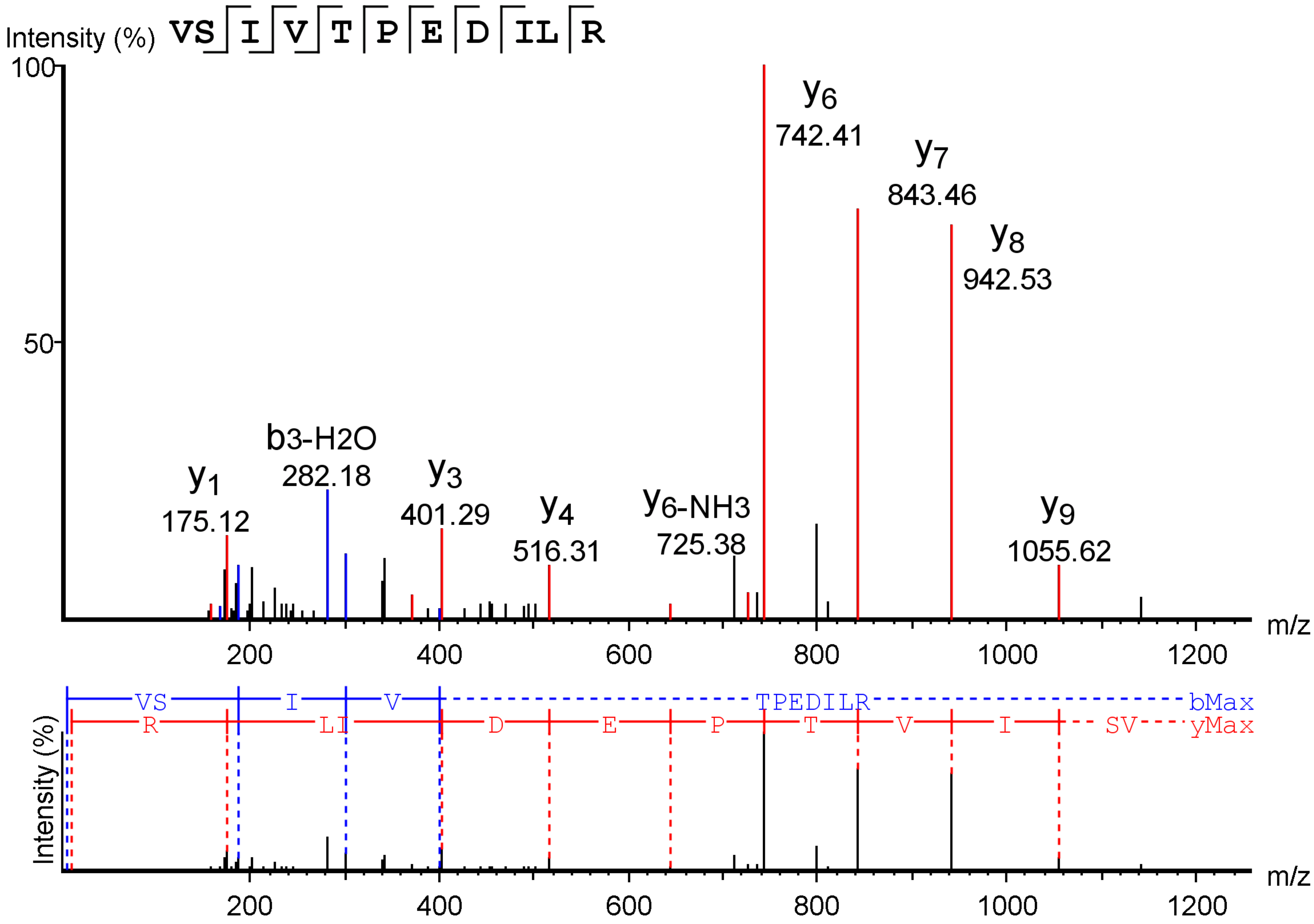

sp|O75110|ATP9A_HUMAN
R.LPTAADLLQIR.S

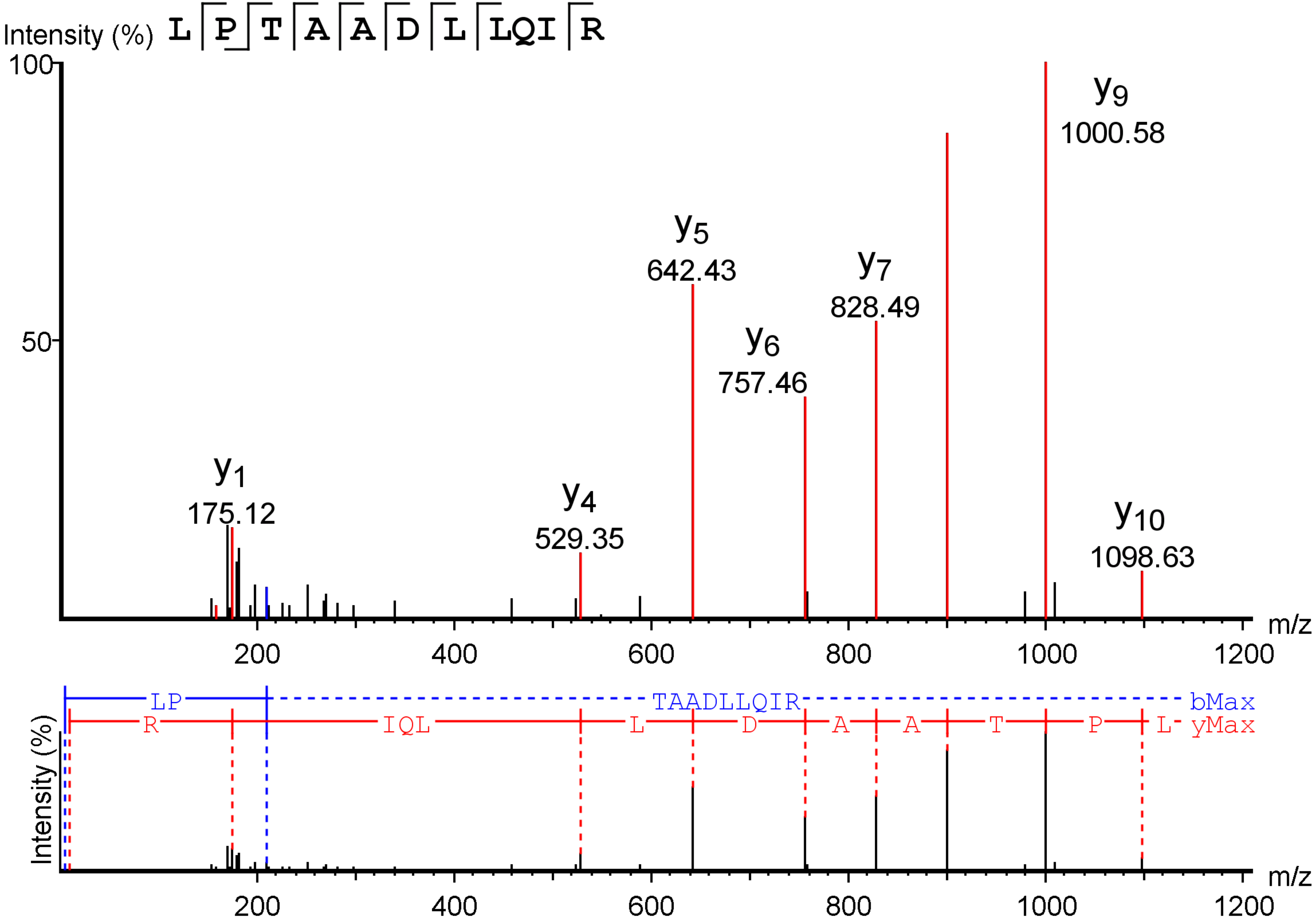

sp|P41732|TSN7_HUMAN
R.TYTDAMQTYNGNDER.S

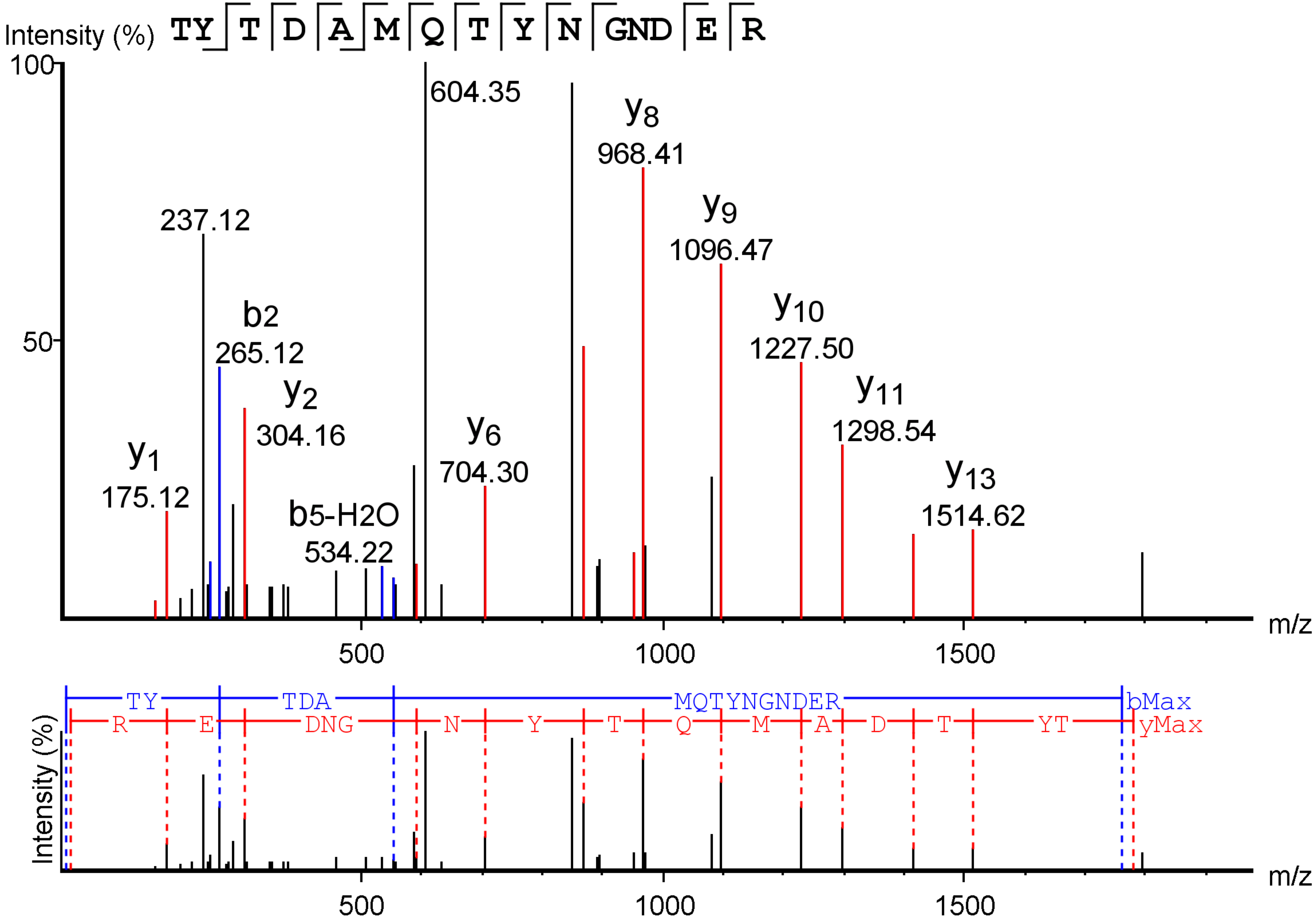

sp|Q9H0C2|ADT4_HUMAN
R.YFPTQALNFAFK.D

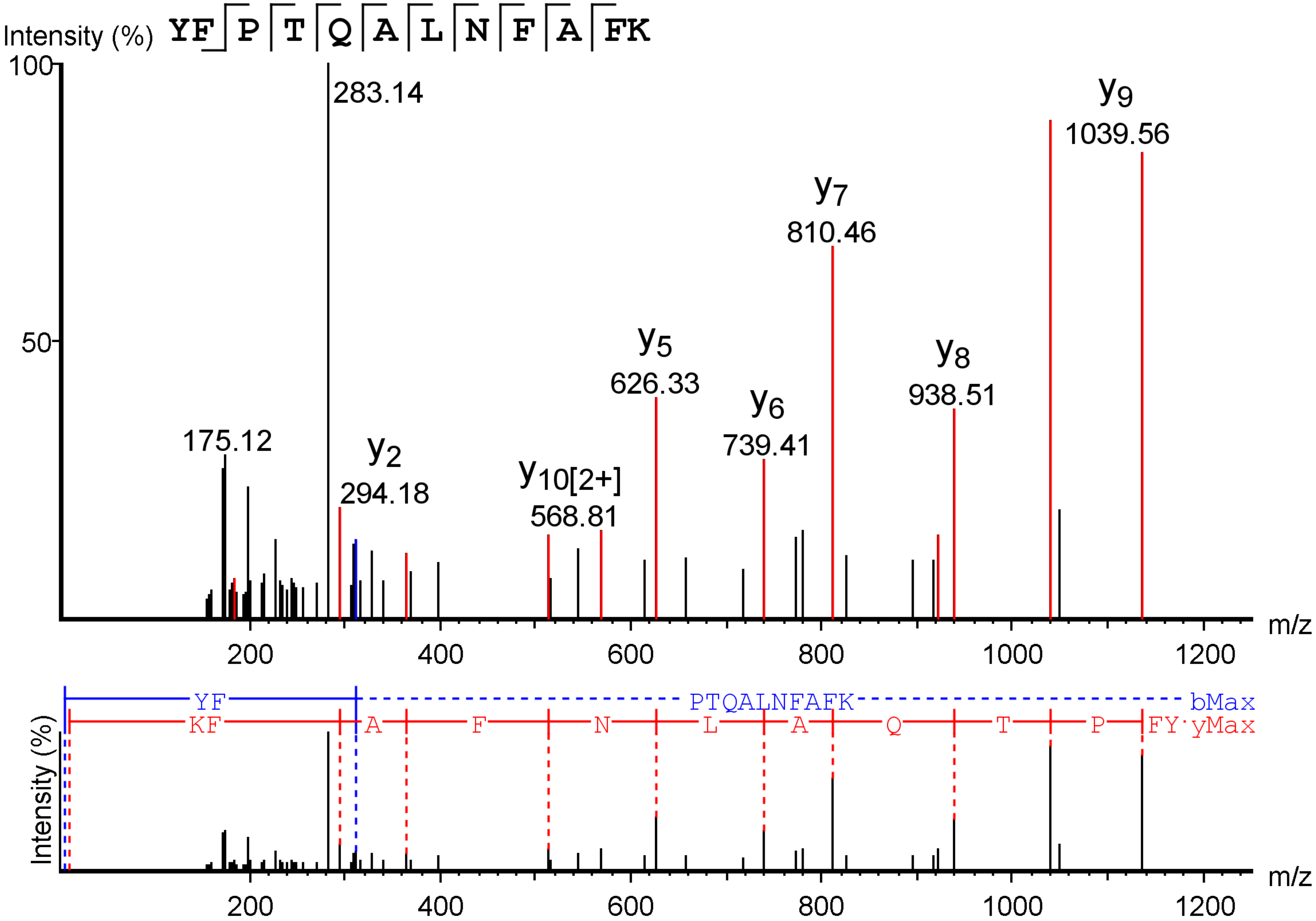

sp|Q6EEV6|SUMO4_HUMAN
K.VAGQDGSVVQFK.I

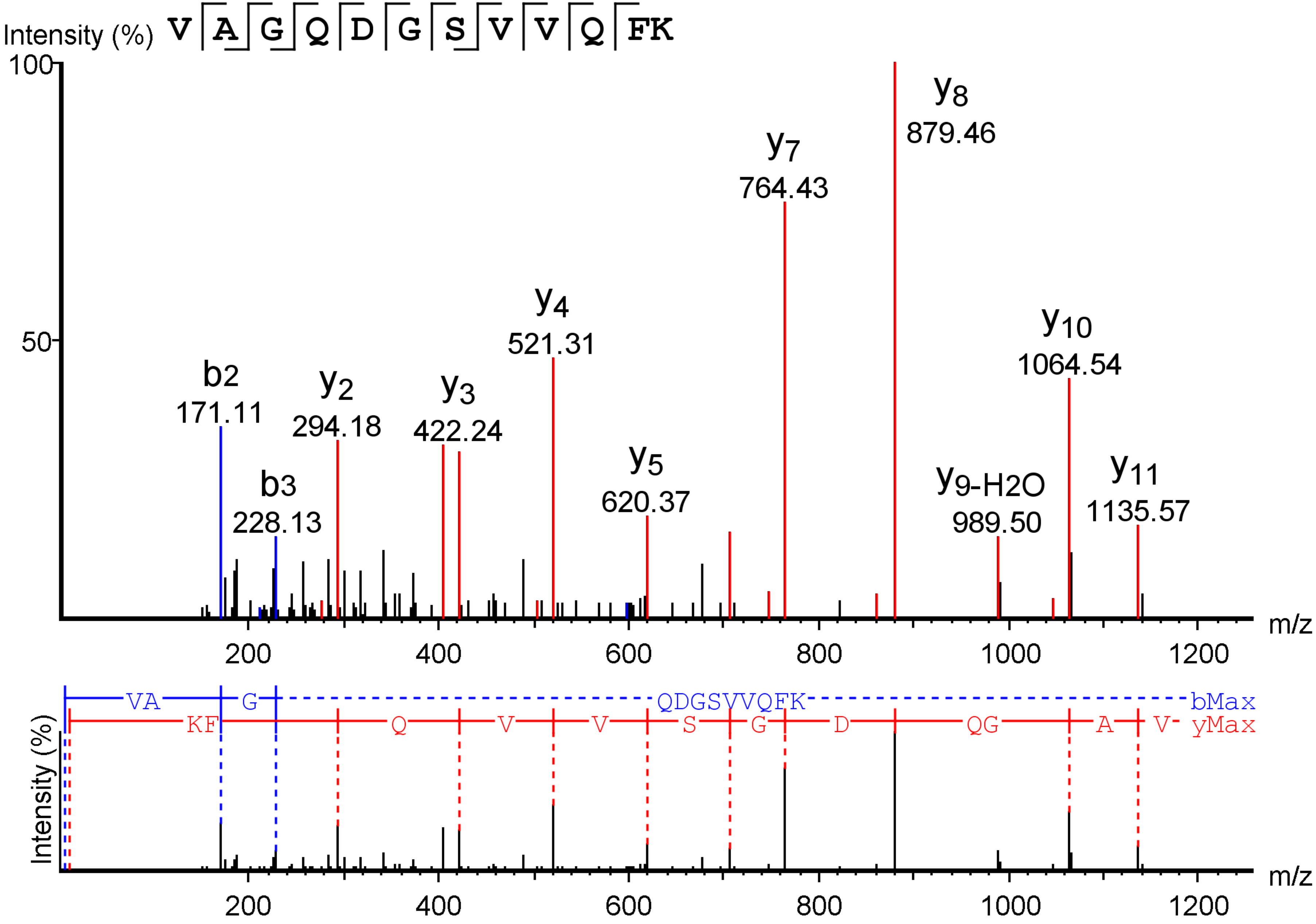

sp|P04278|SHBG_HUMAN
K.QAEISASAPTSLR.S

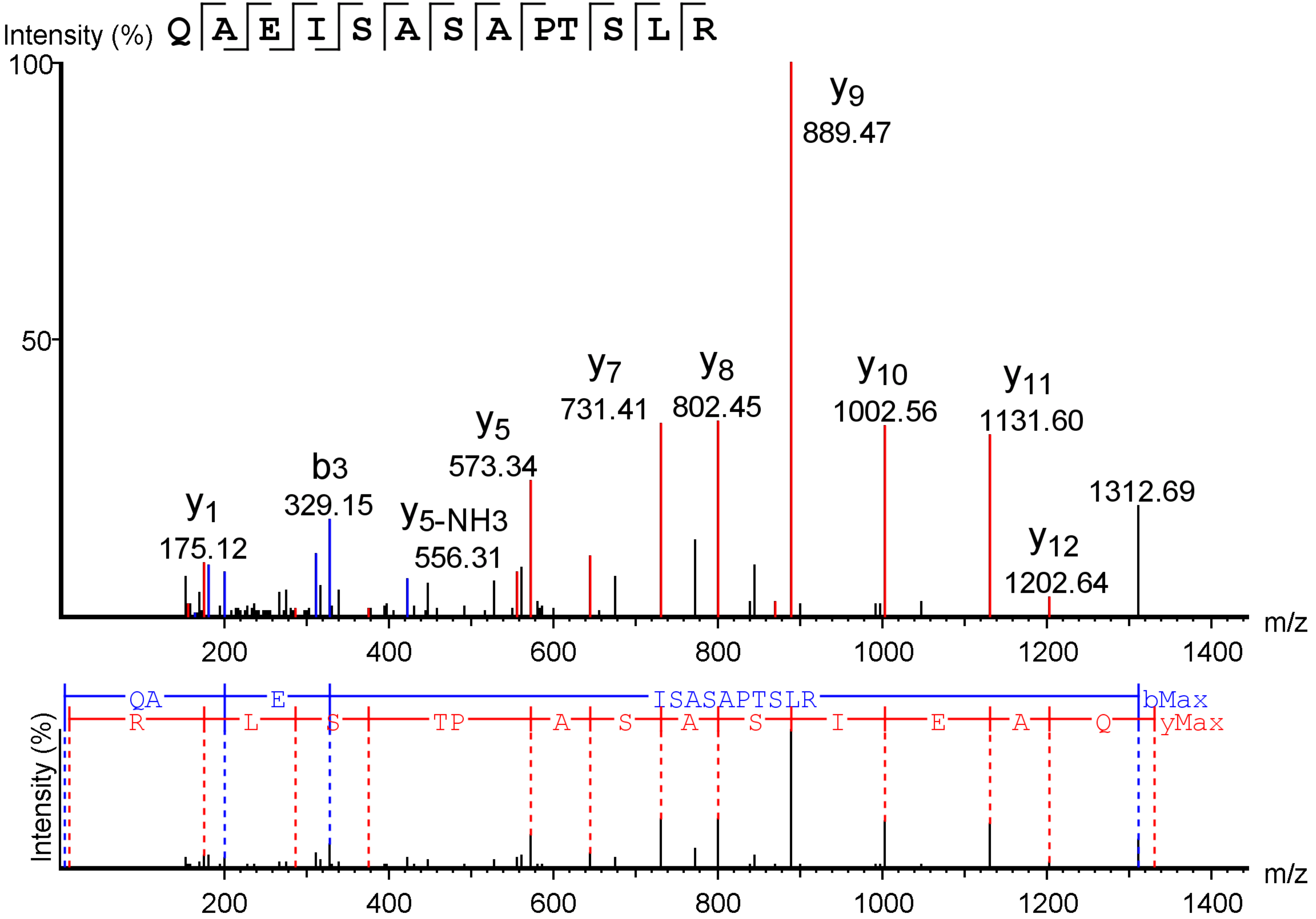

sp|Q9NRX4|PHP14_HUMAN
K.QGC(+57.02)DC(+57.02)EC(+57.02)LGGGR.I

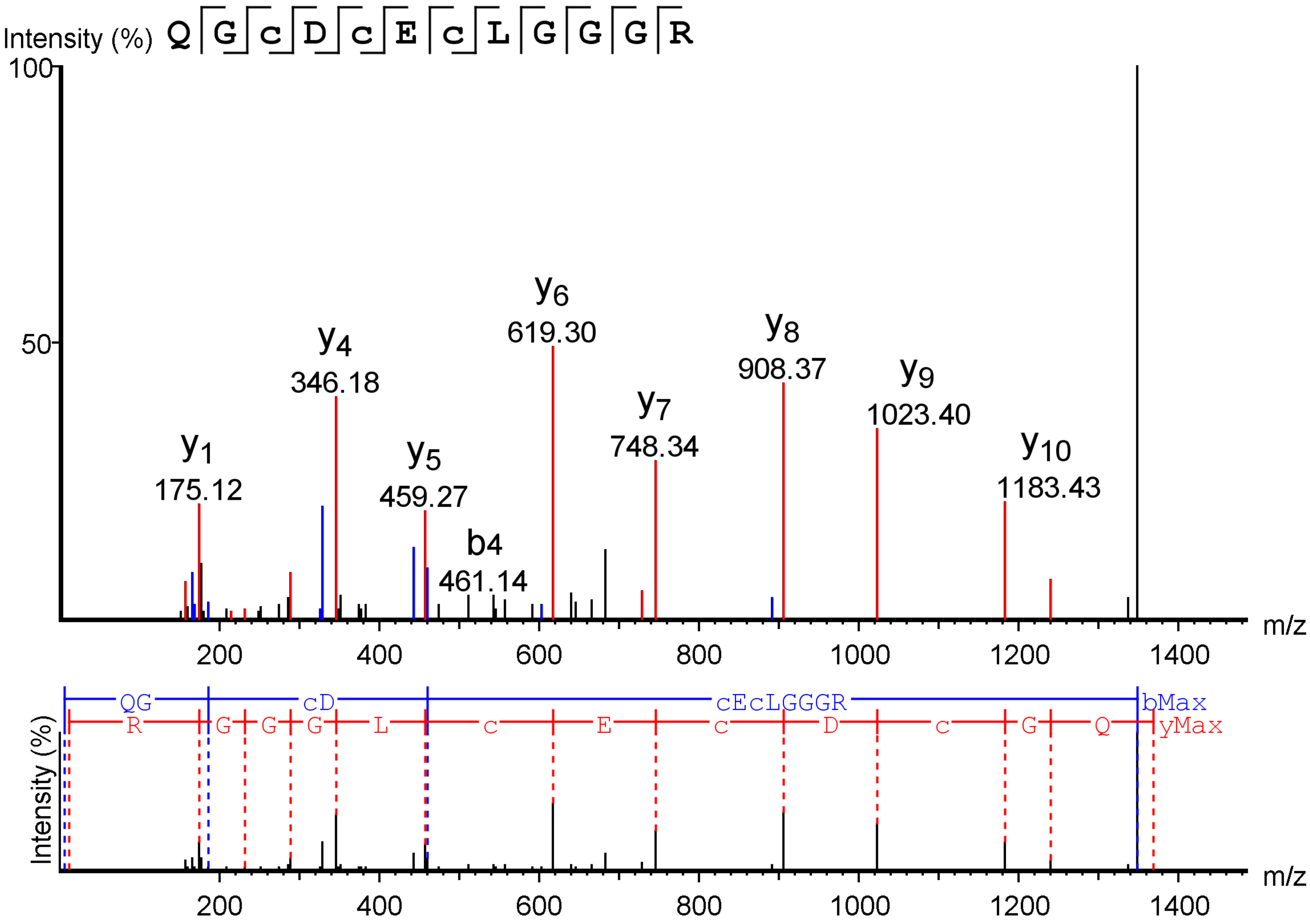

sp|P01766|HV313_HUMAN
R.AGDTAVYYC(+57.02)AR

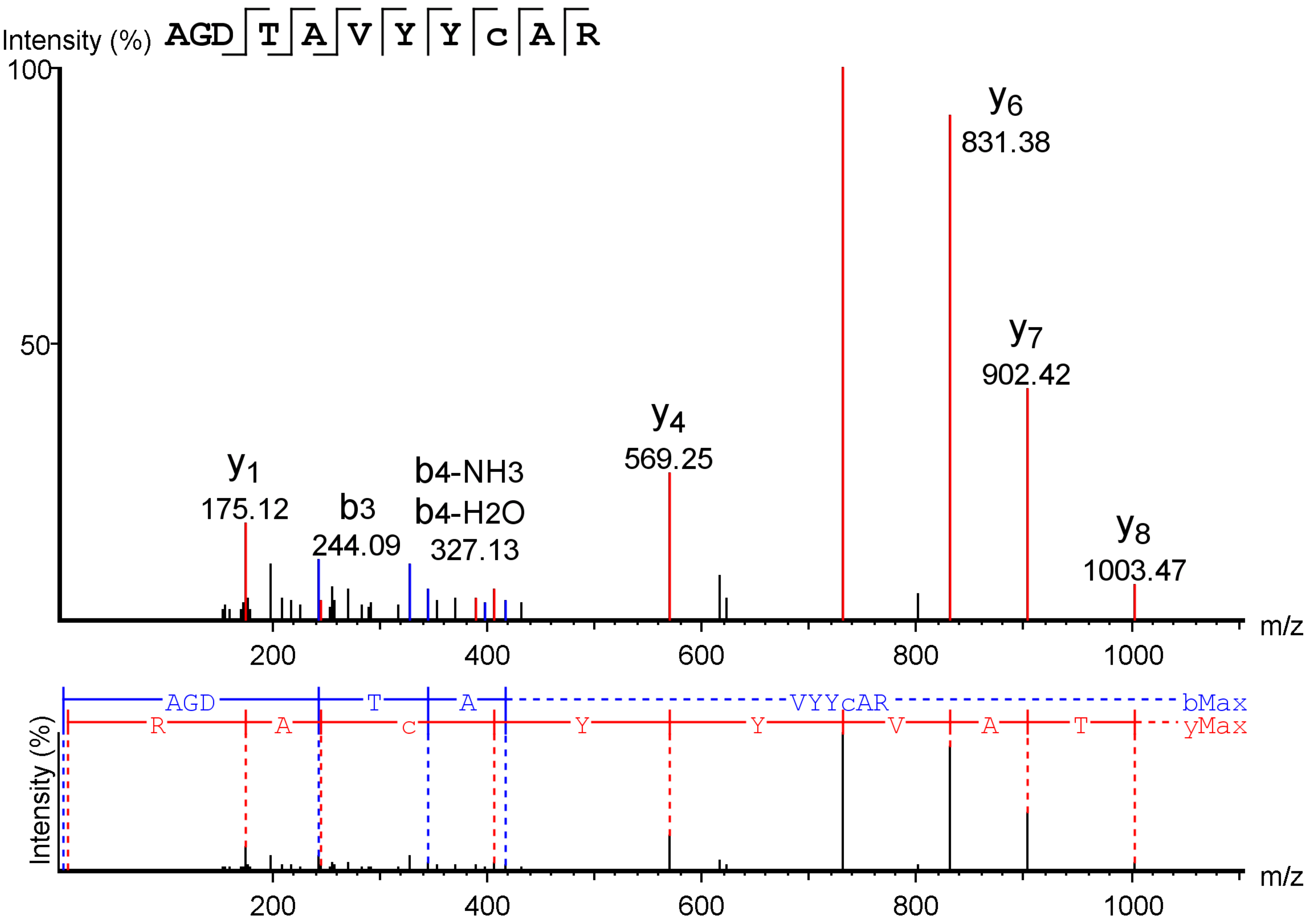

J3KTF8|J3KTF8_HUMAN
R.AEEYEFLTPVEEAPK.G

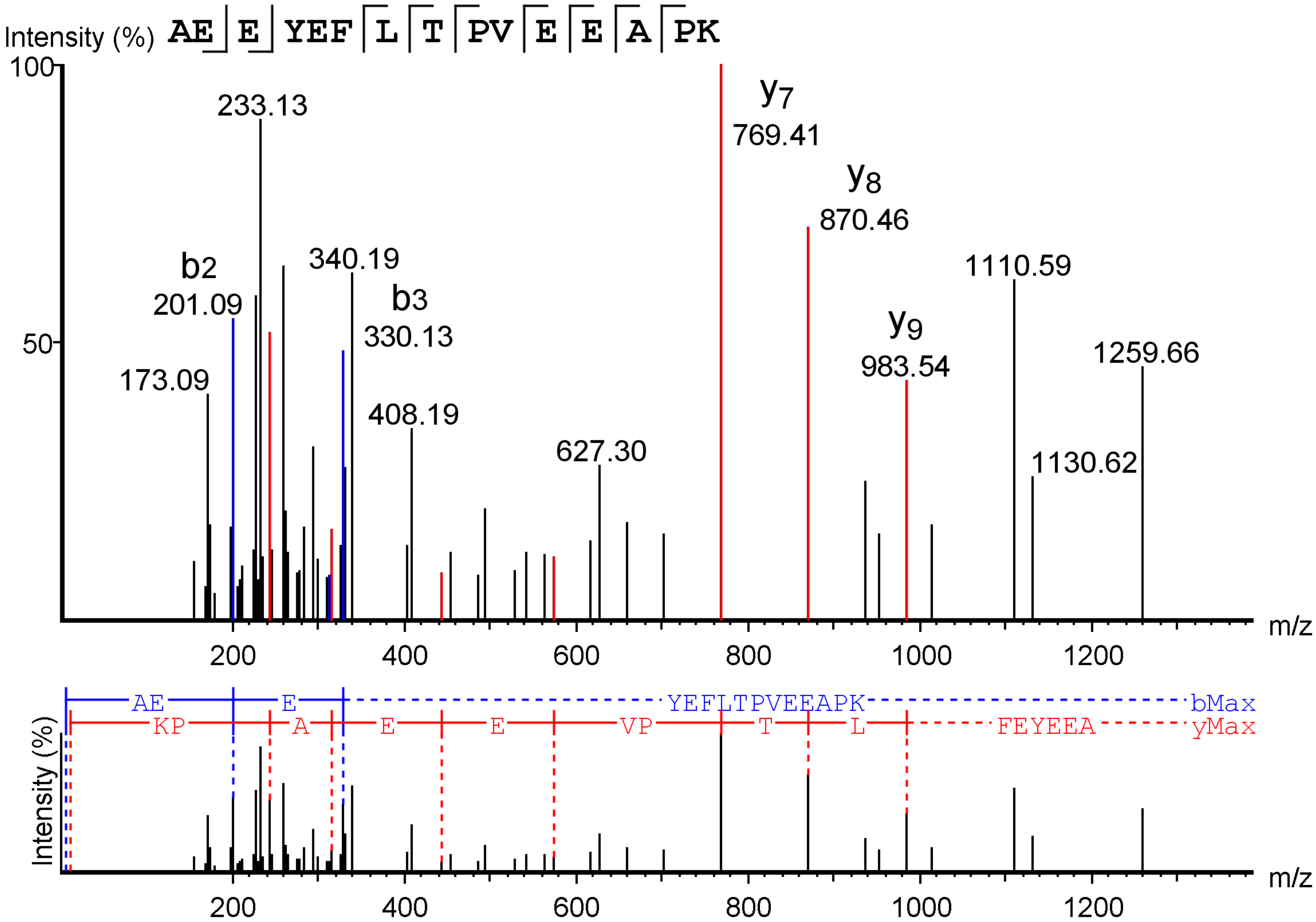

H3BN14|H3BN14_HUMAN
R.LLPVQENFLLK.F

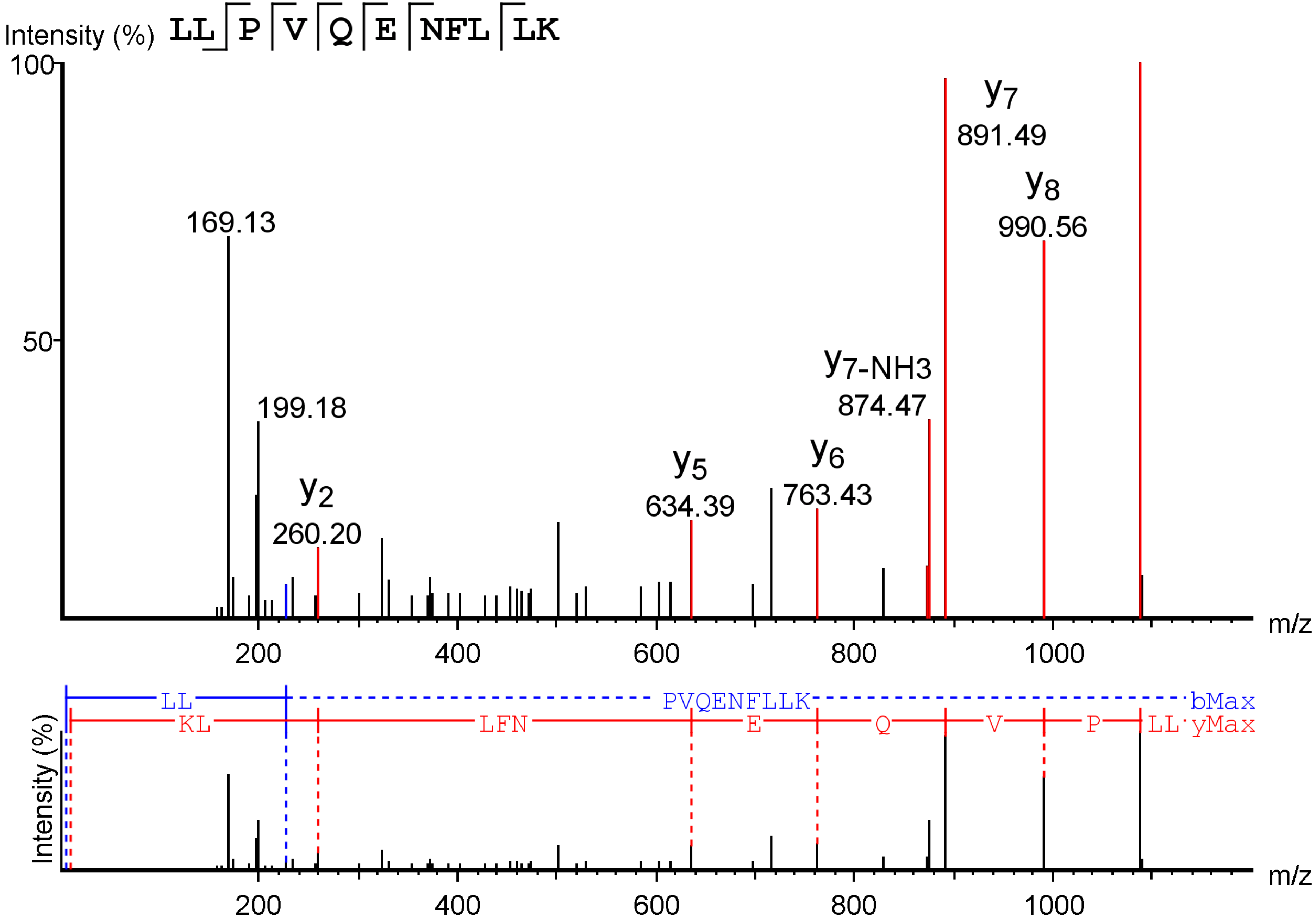

sp|Q9Y4F1|FARP1_HUMAN
K.DLIGIDNLVVPGR.E

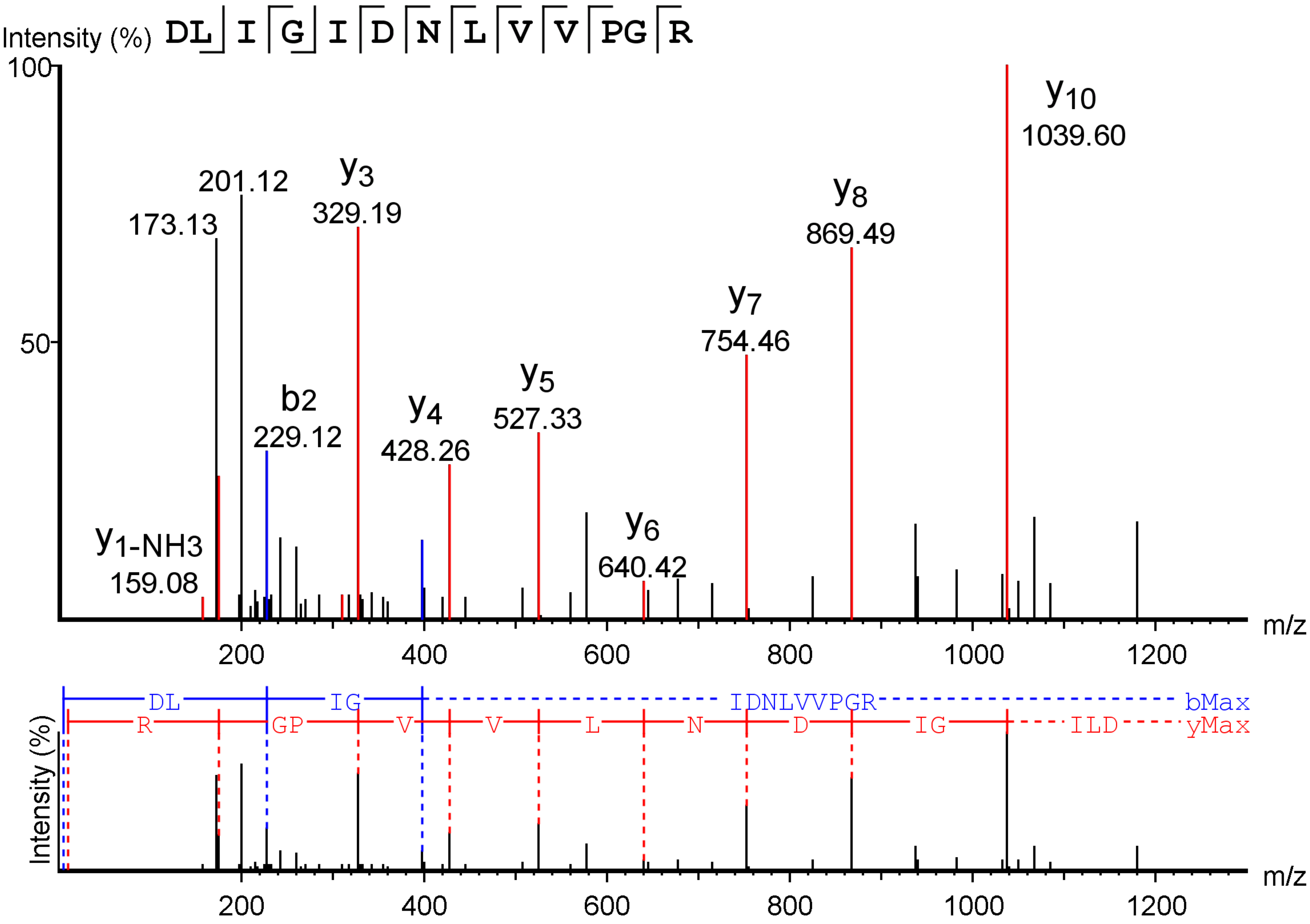

sp|O00231|PSD11_HUMAN
K.LYDNLLEQNLIR.V

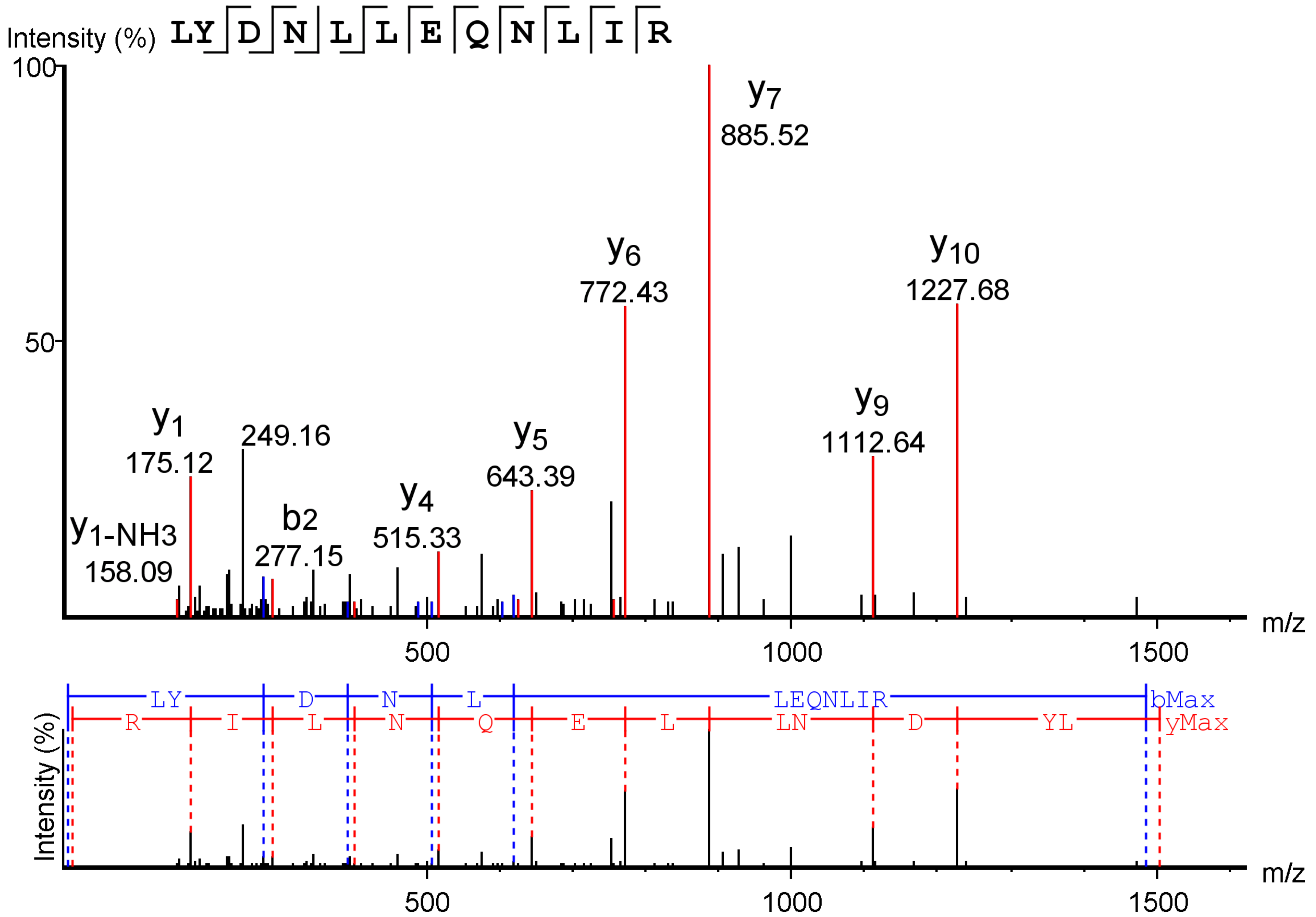

A0A0C4DH35|A0A0C4DH35_HUMAN
R.AEDTAVYYC(+57.02)VR

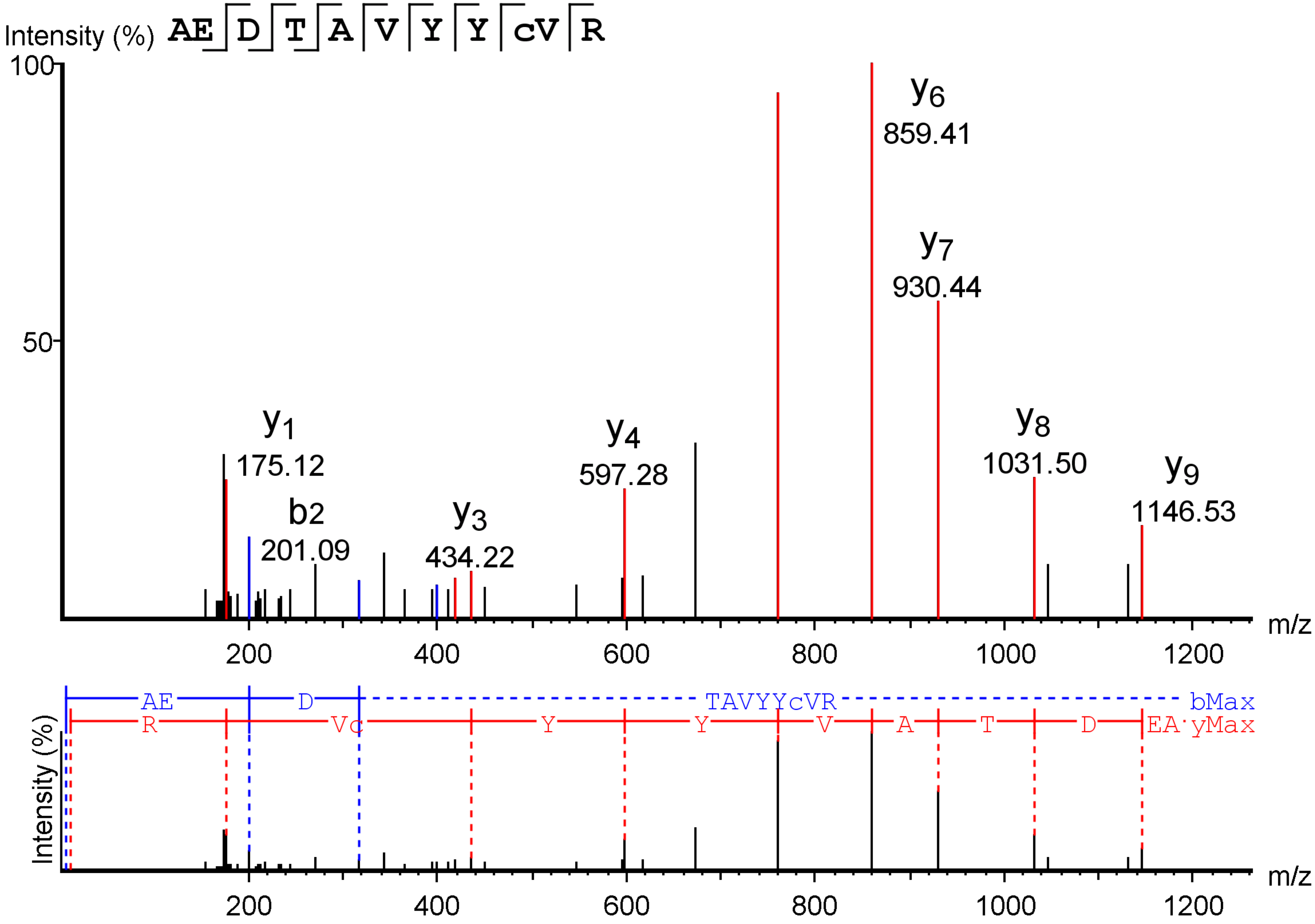

sp|Q8IUG5|MY18B_HUMAN
K.QMVLHEK.Q

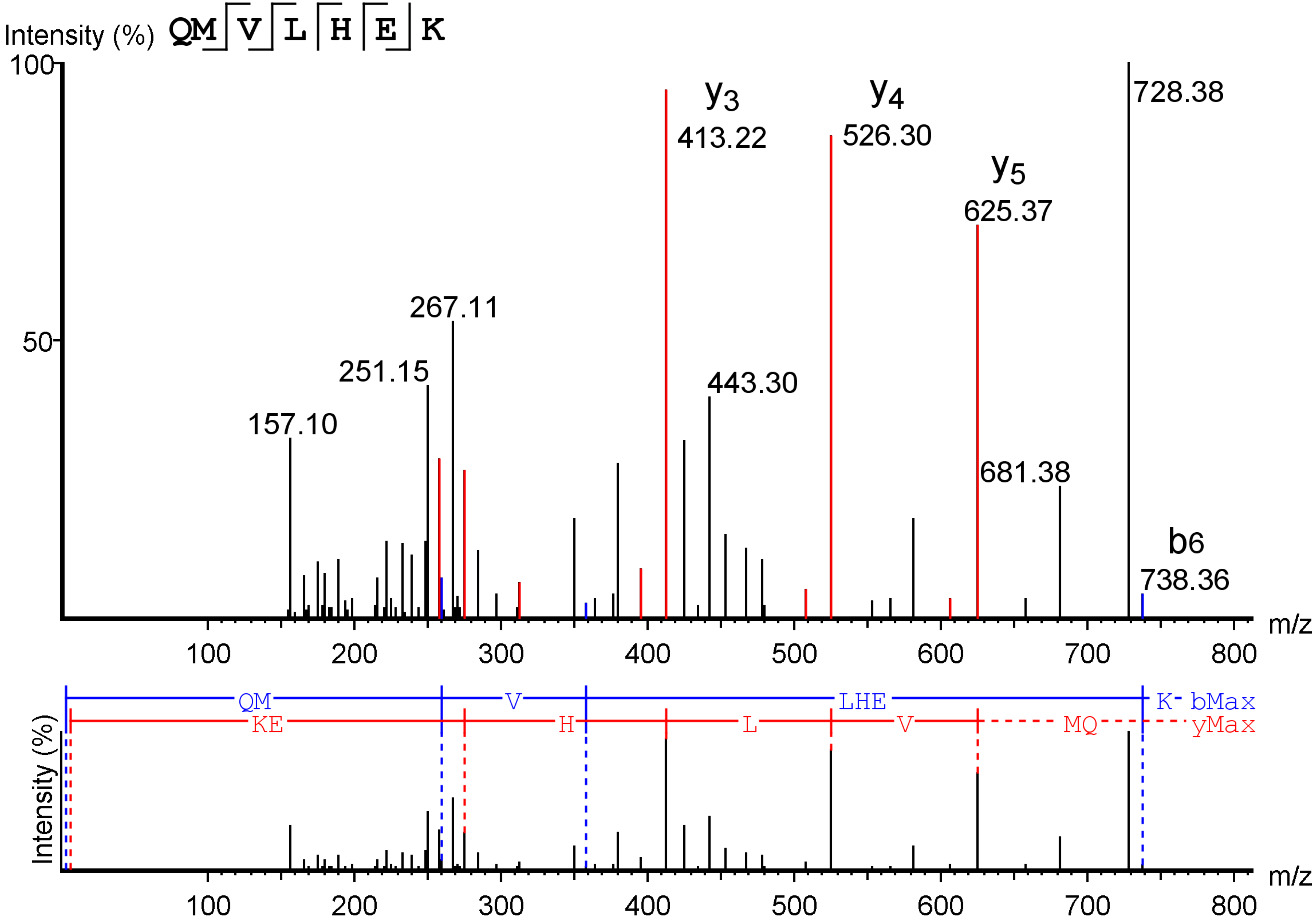

sp|Q9NRX5|SERC1_HUMAN
K.LTLTSDESTLIEDGGAR.S

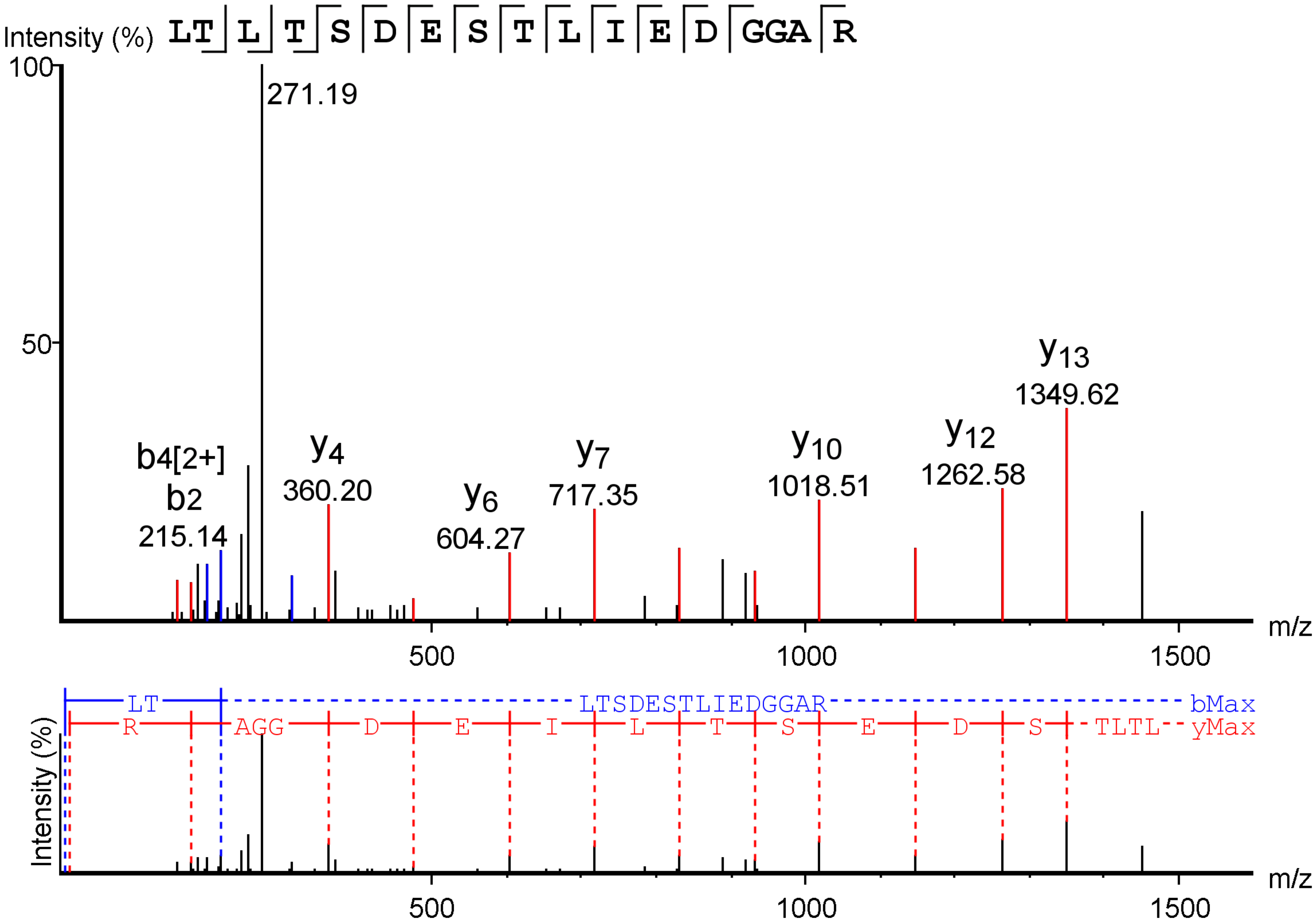

Q5T8U3|Q5T8U3_HUMAN
R.LKVPPAINQFTQALDR.Q

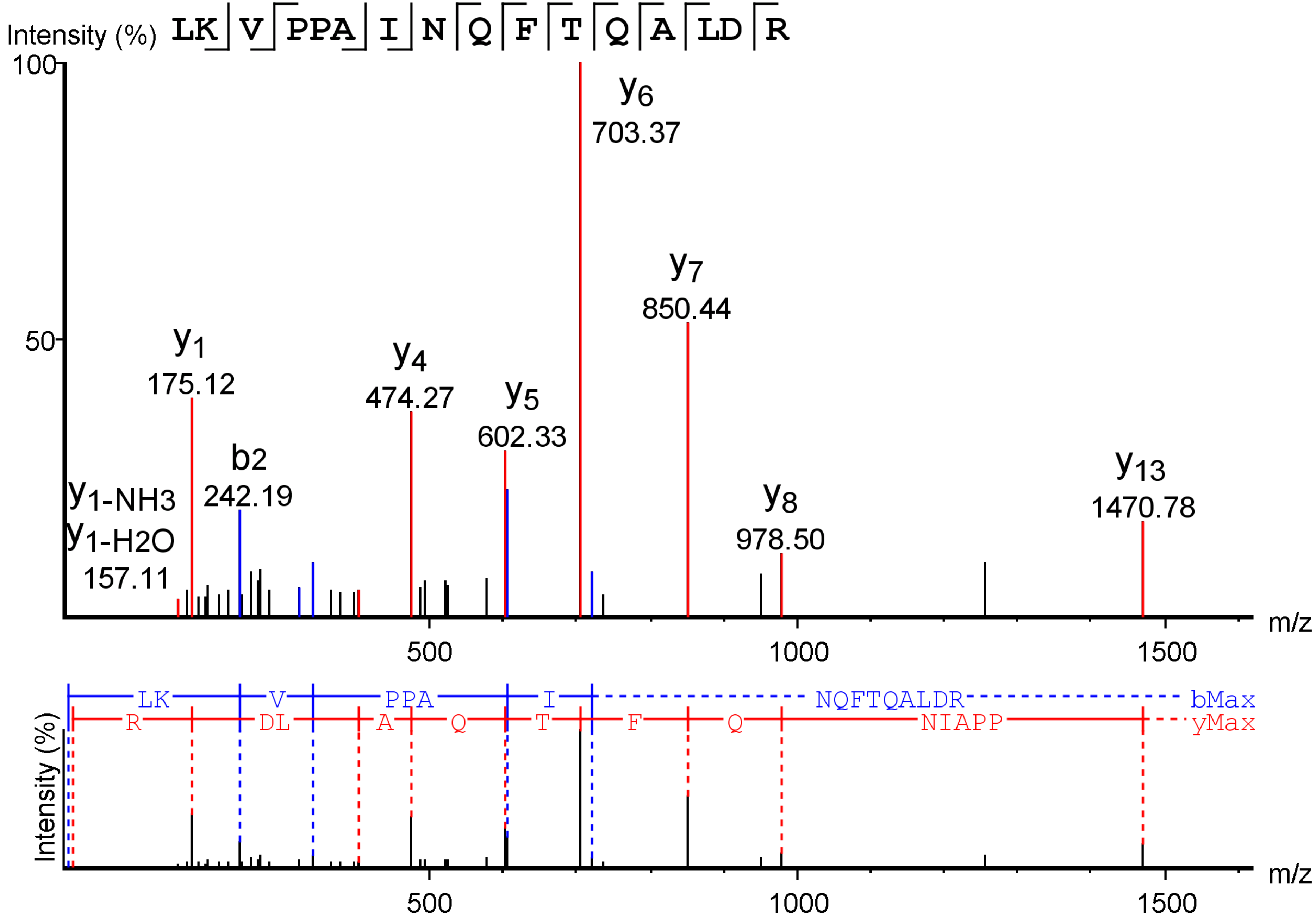

sp|Q6W2J9|BCOR_HUMAN
R.LSNGKYPK.A

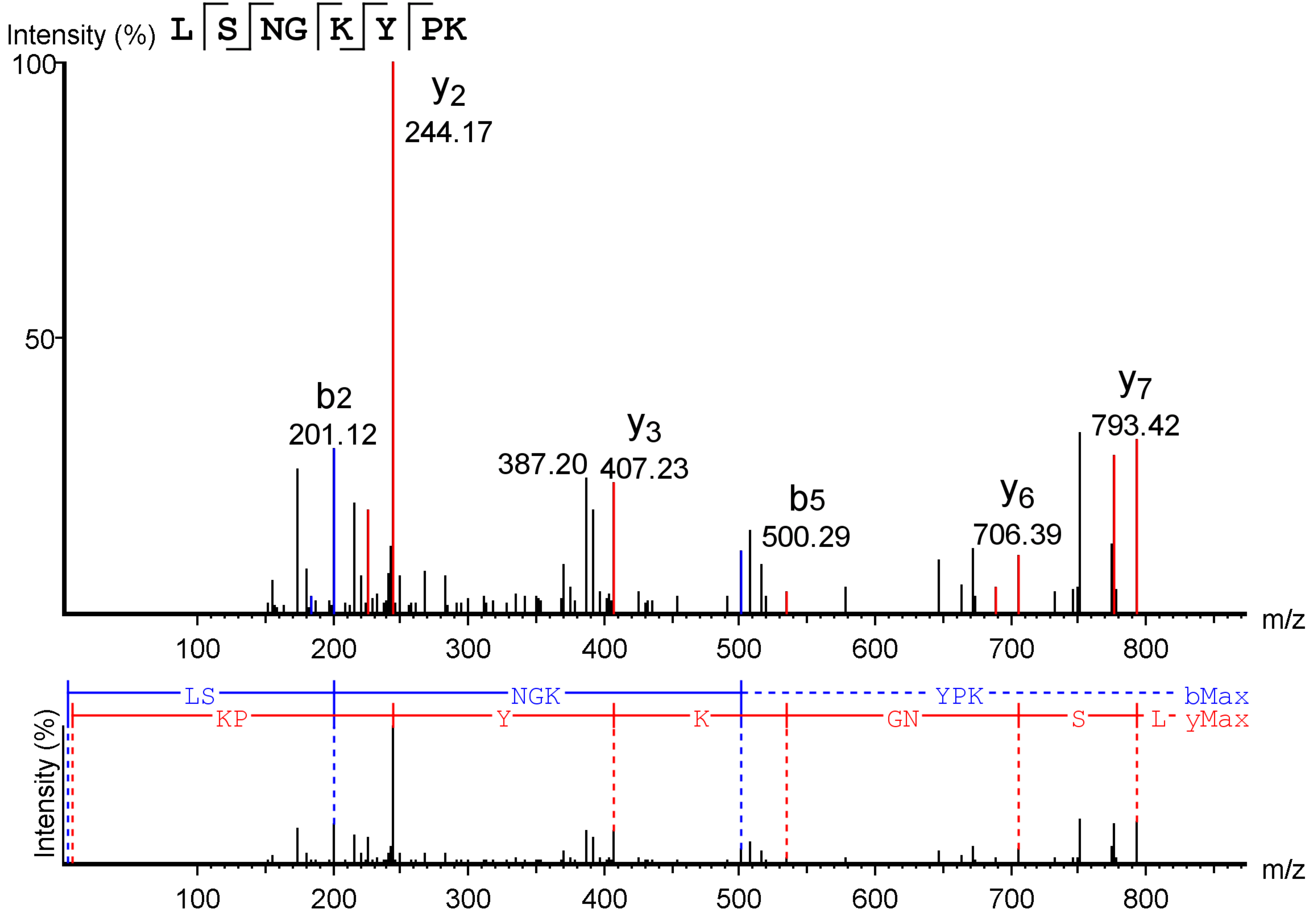

sp|O75340|PDCD6_HUMAN
K.AGVNFSEFTGVWK.Y

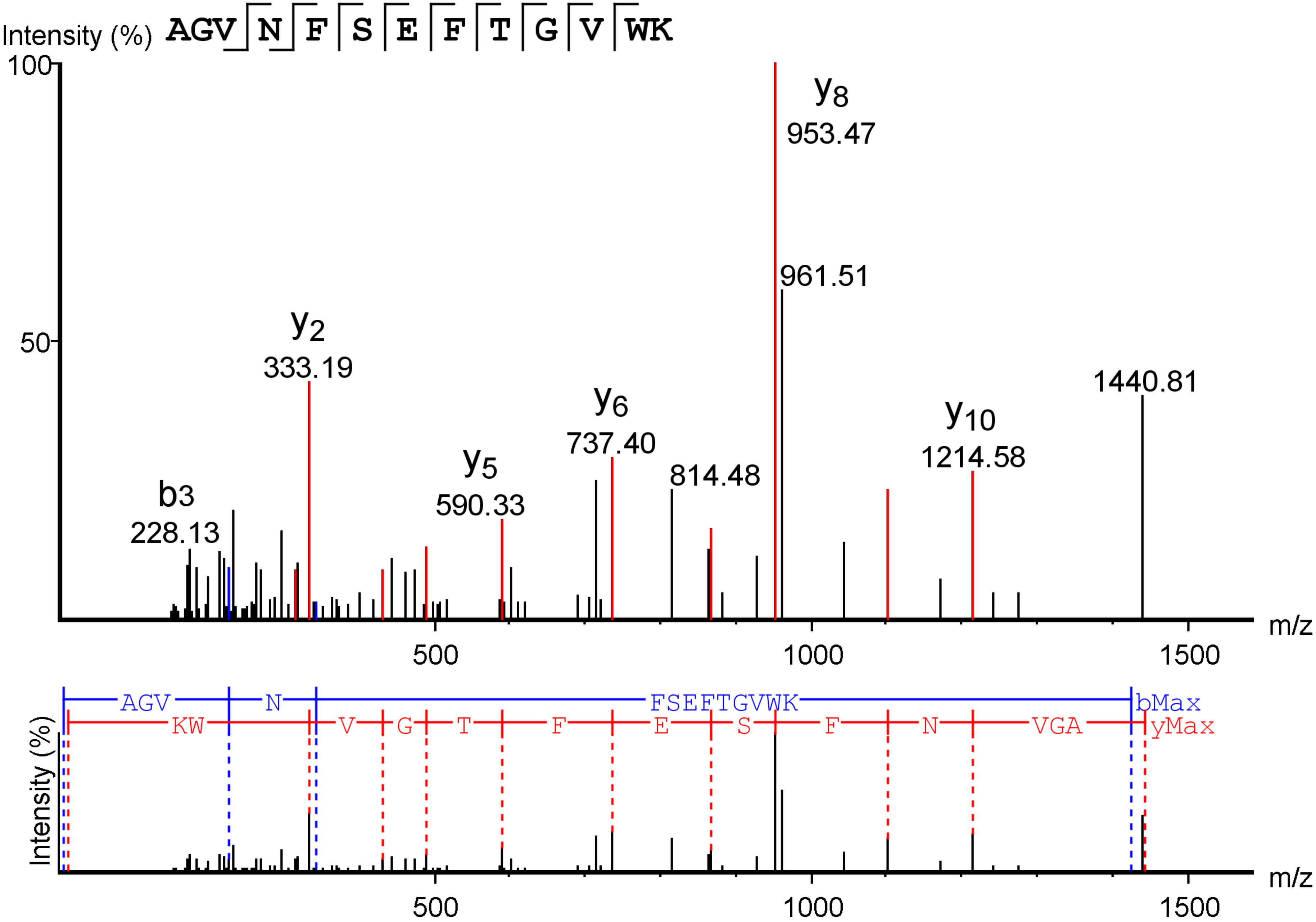

sp|P16278|BGAL_HUMAN
K.TVGAALDILC(+57.02)PSGPIK.S

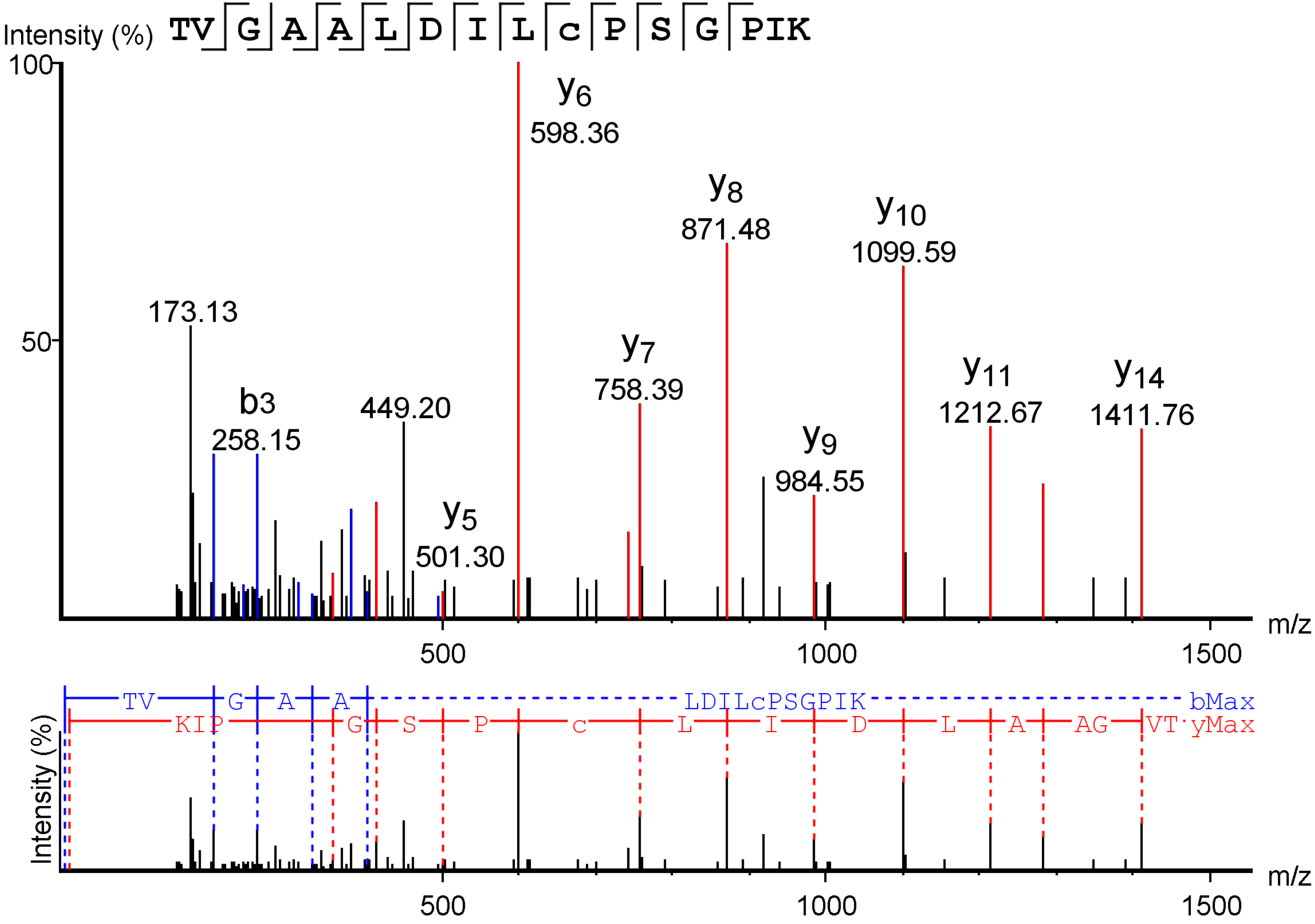

sp|A0A0A0MS15|HV349_HUMAN
K.GLEWVGFIR.S

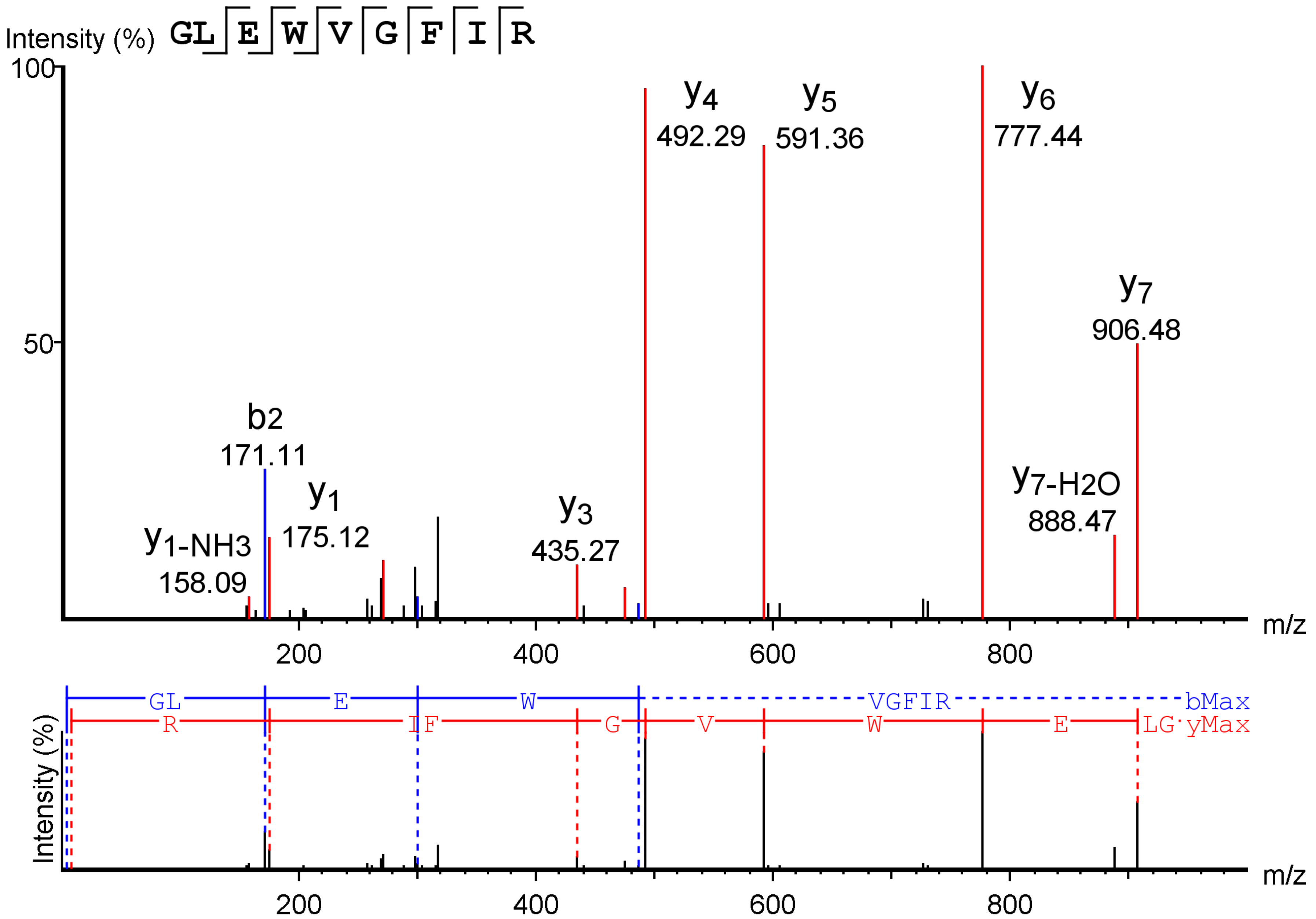

sp|P21926|CD9_HUMAN
K.KDVLETFTVK.S

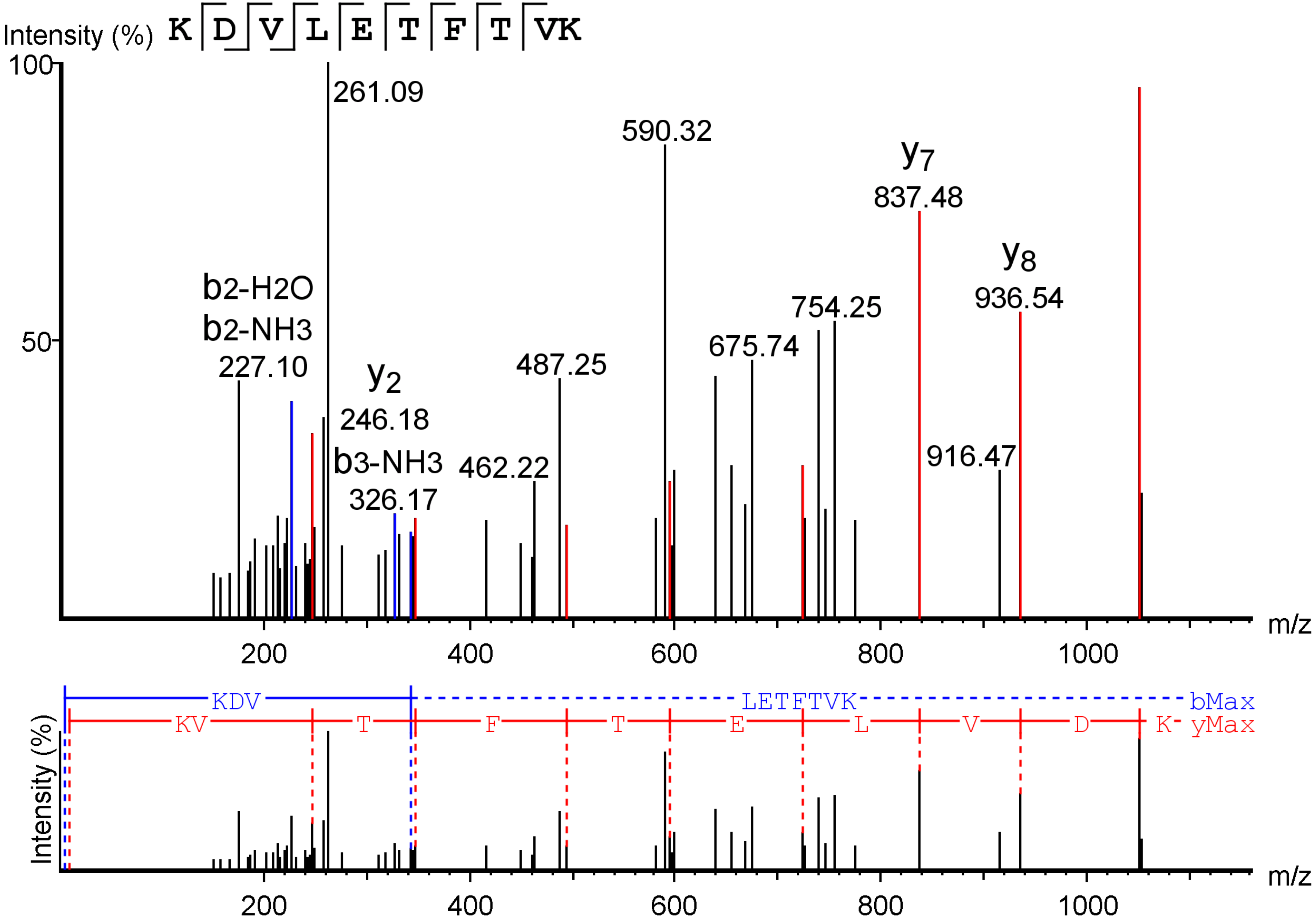

sp|Q5VST9|OBSCN_HUMAN
R.IEAAGC(+57.02)MR.Q

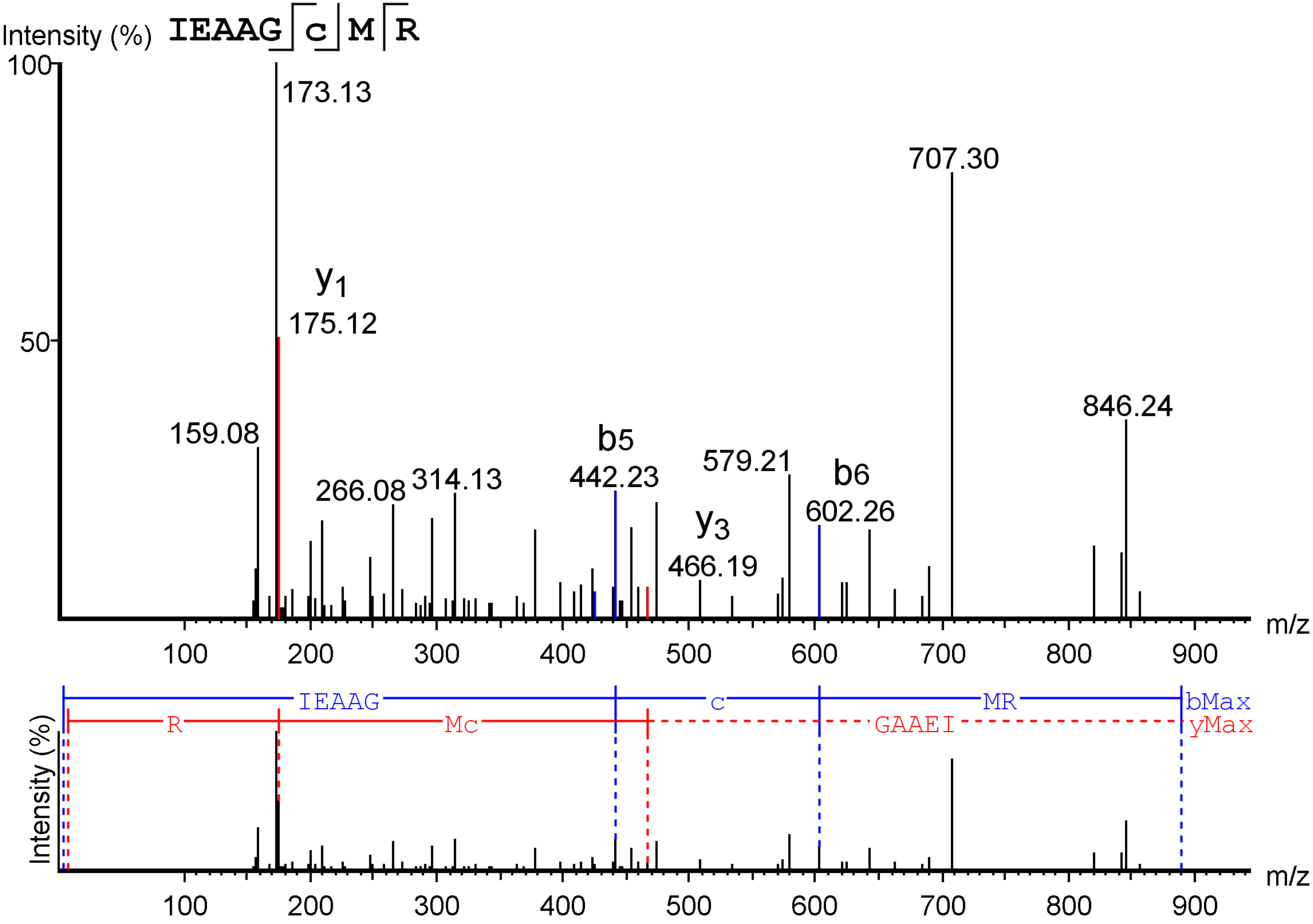

sp|P67870|CSK2B_HUMAN
K.YQQGDFGYC(+57.02)PR.V

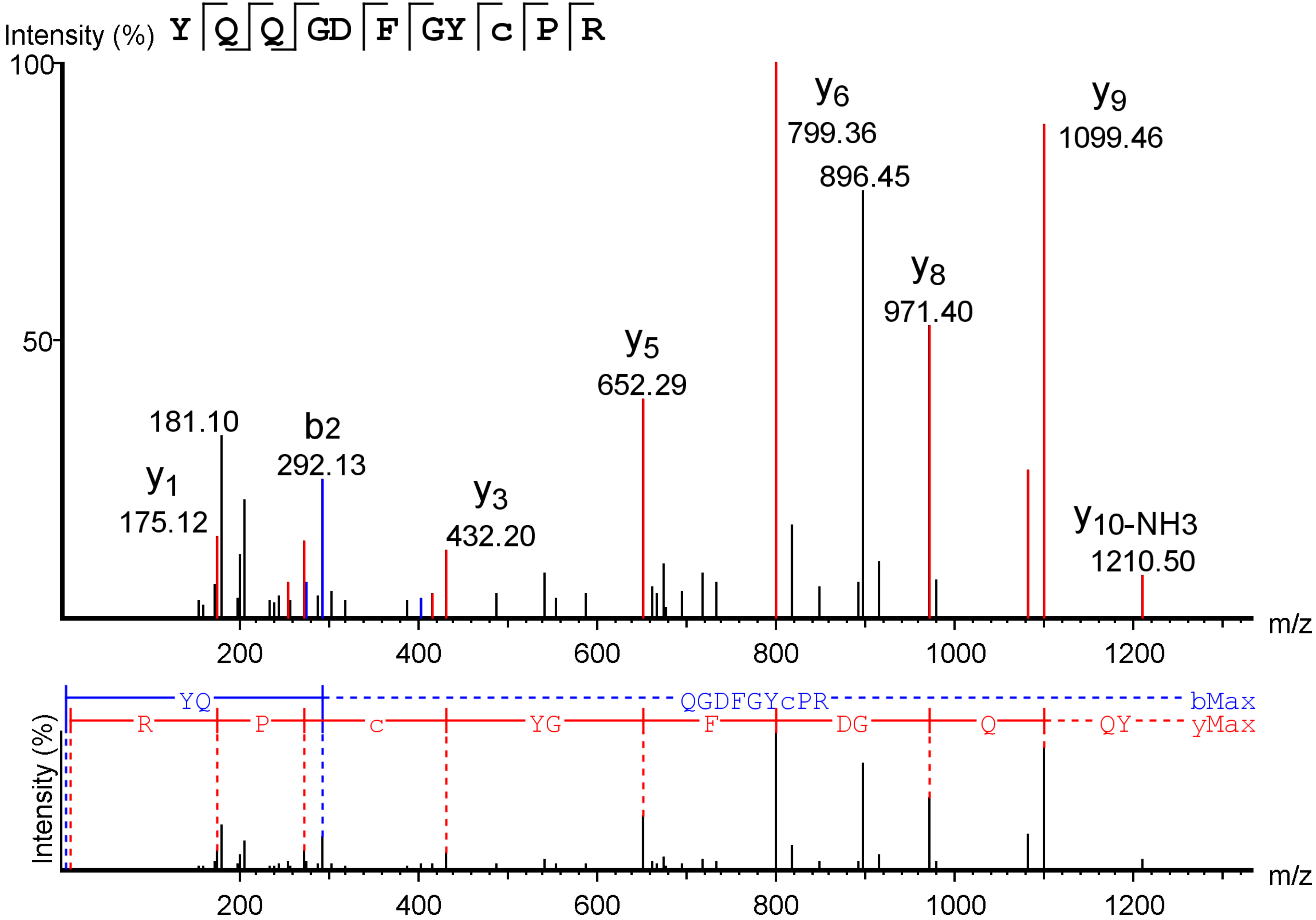

sp|P35325|SPR2B_HUMAN
K.C(+57.02)PEPC(+57.02)PPPK.C

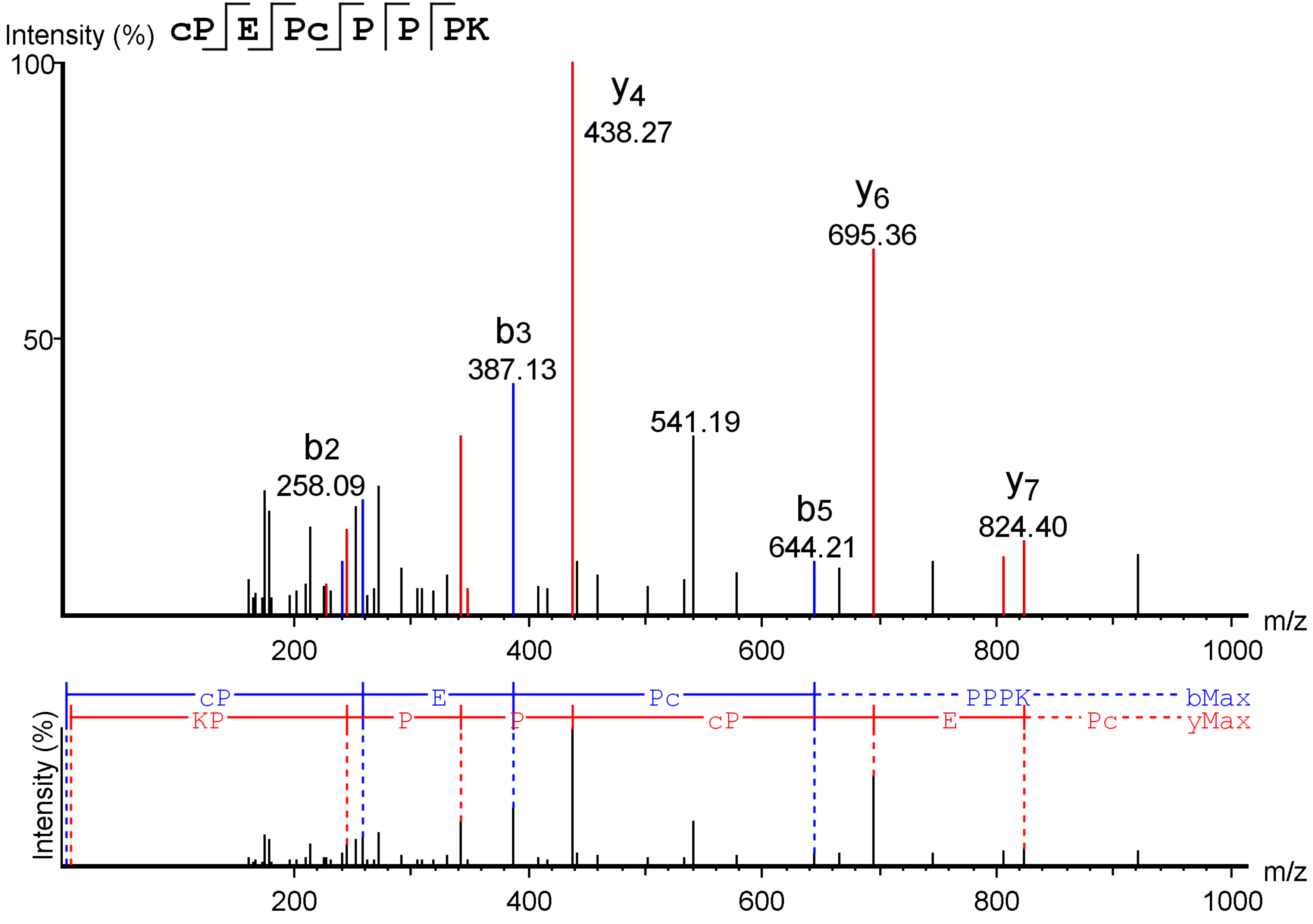

A0A087WZE9|A0A087WZE9_HUMAN
R.LSAKPAPPKPEPKPR.K

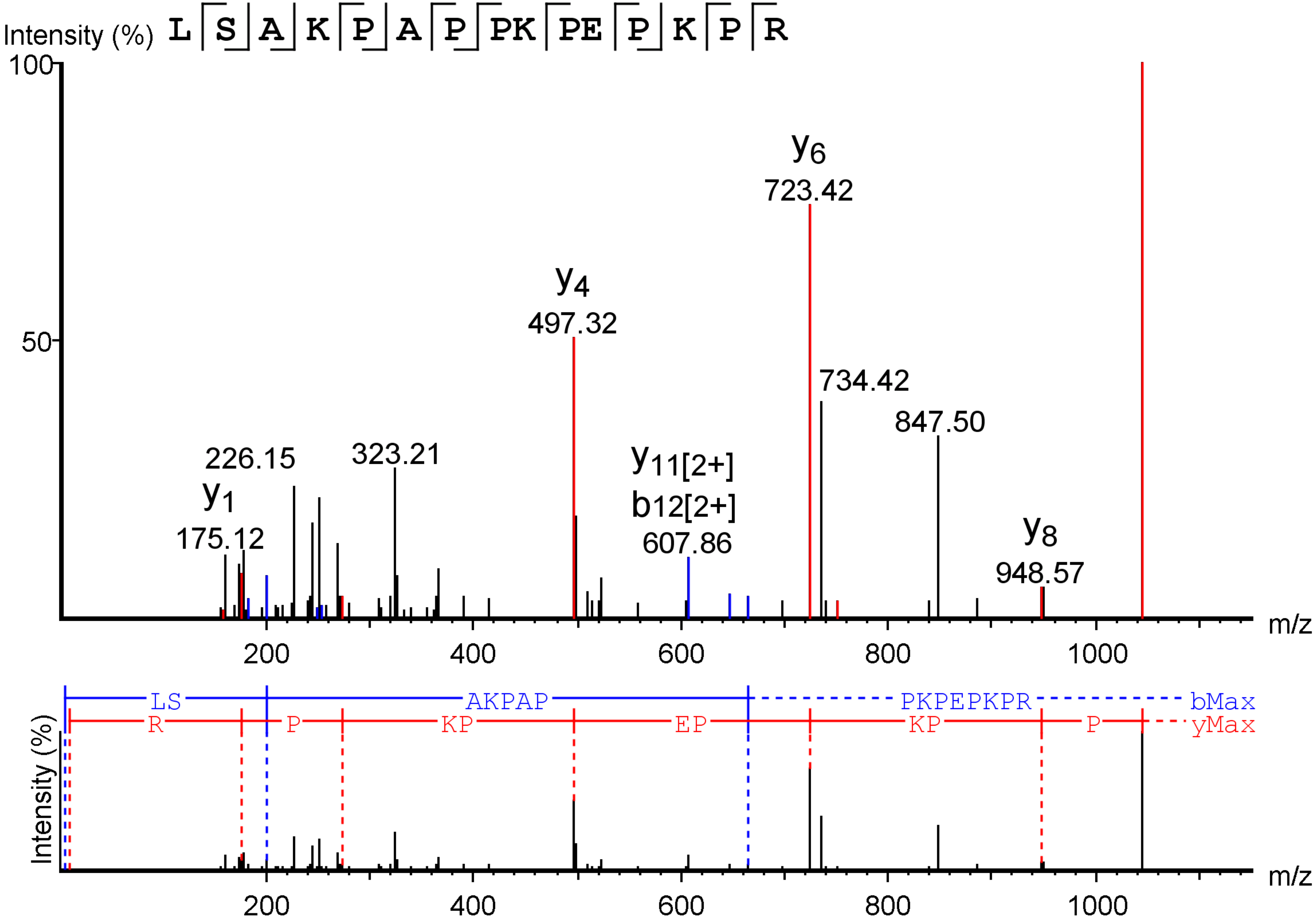

sp|P43007|SATT_HUMAN
K.ETVDSFLDLAR.N

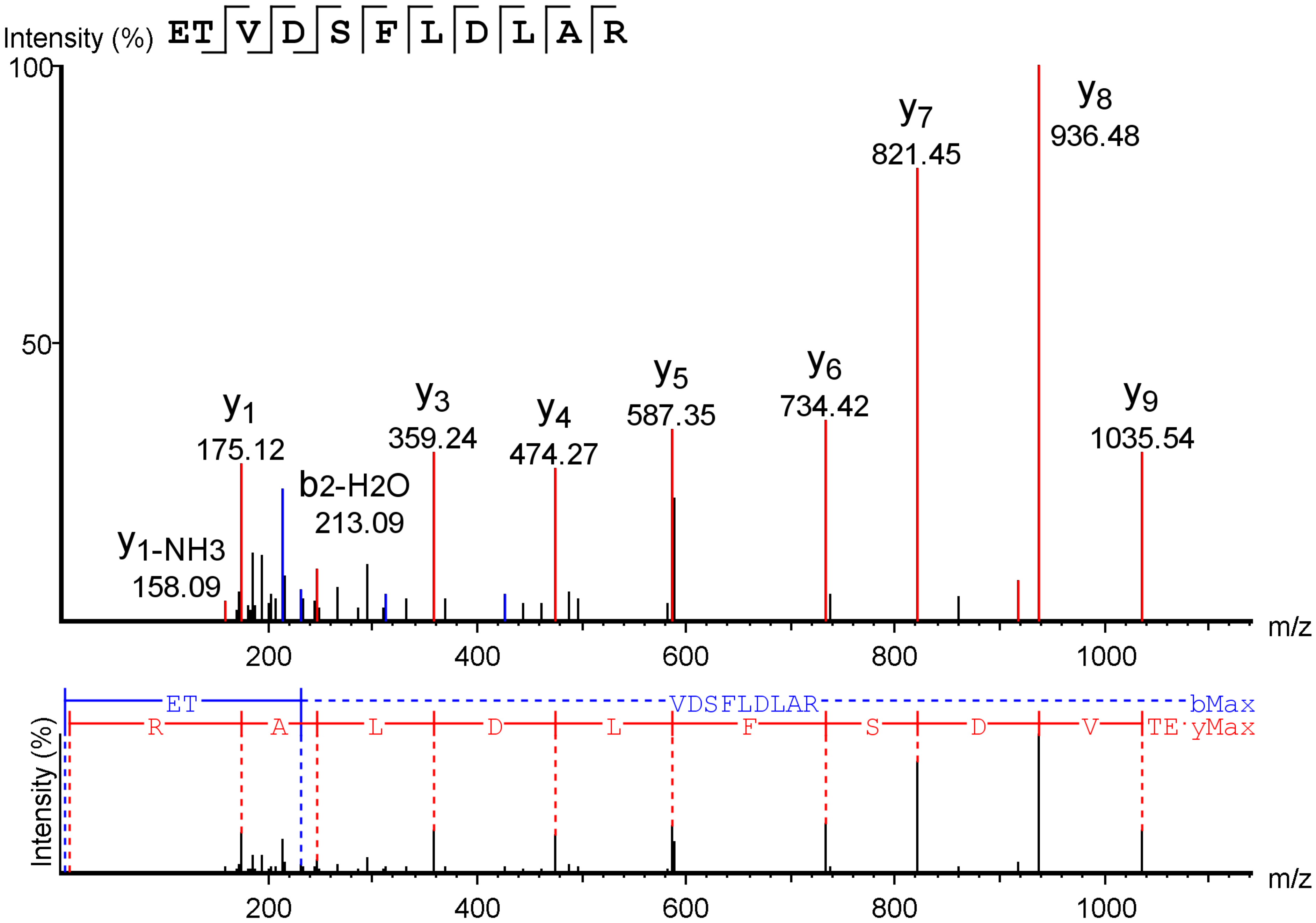

sp|Q9UBP4|DKK3_HUMAN
R.SAVEEMEAEEAAAK.A

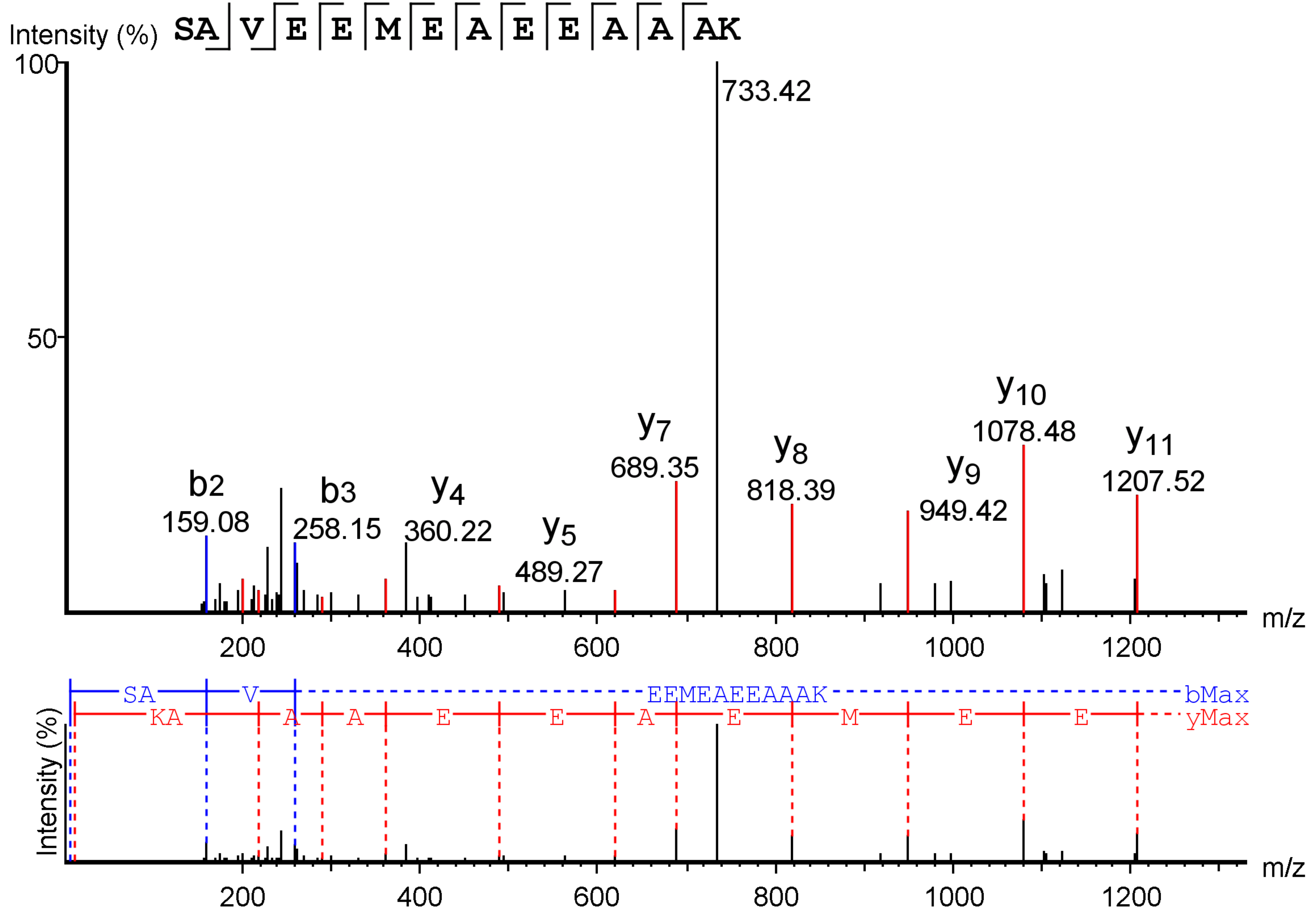

sp|O75348|VATG1_HUMAN
M.A(+42.01)SQSQGIQQLLQAEK.R

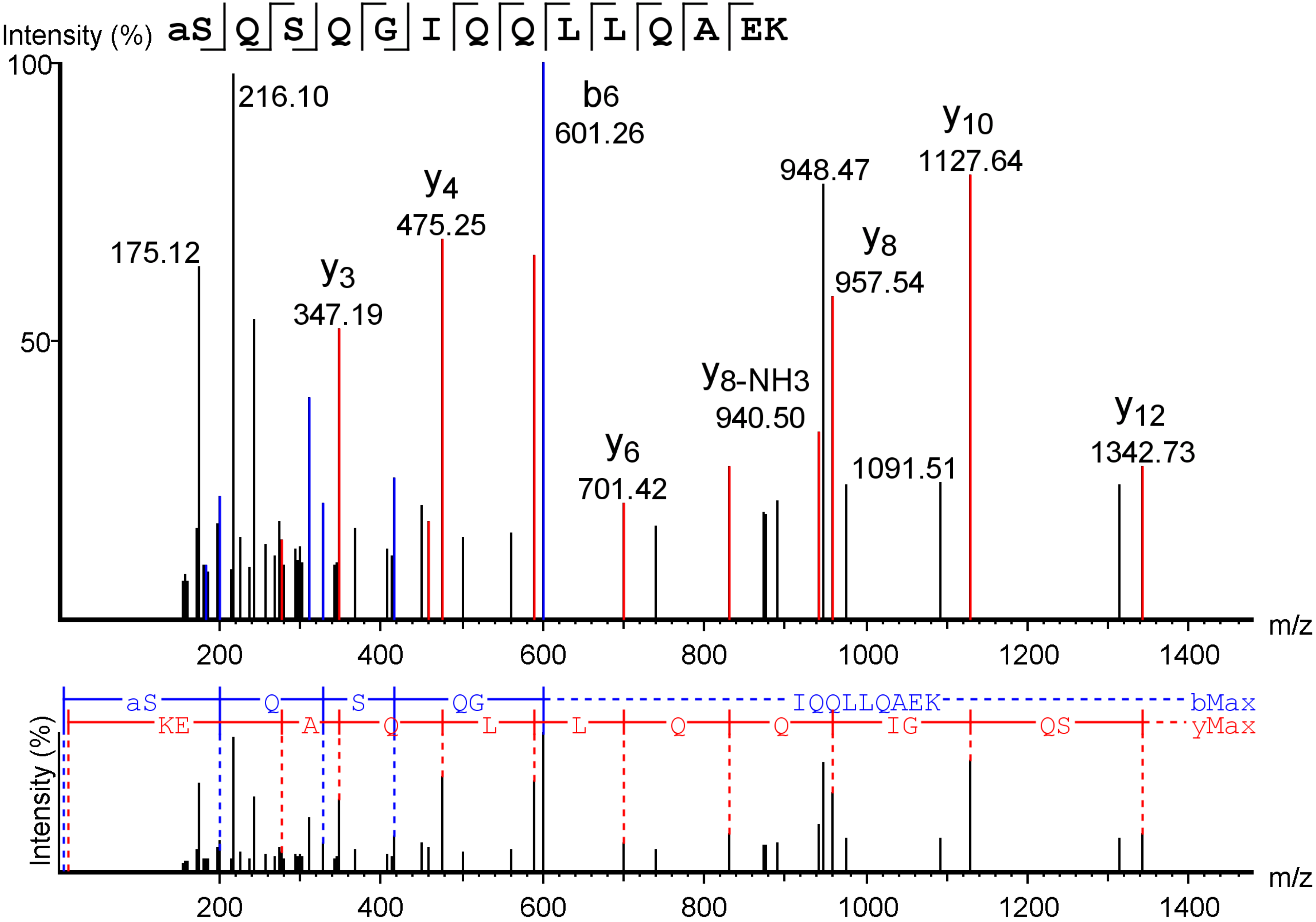

sp|Q15599|NHRF2_HUMAN
R.LLVVDPETDEHFK.R

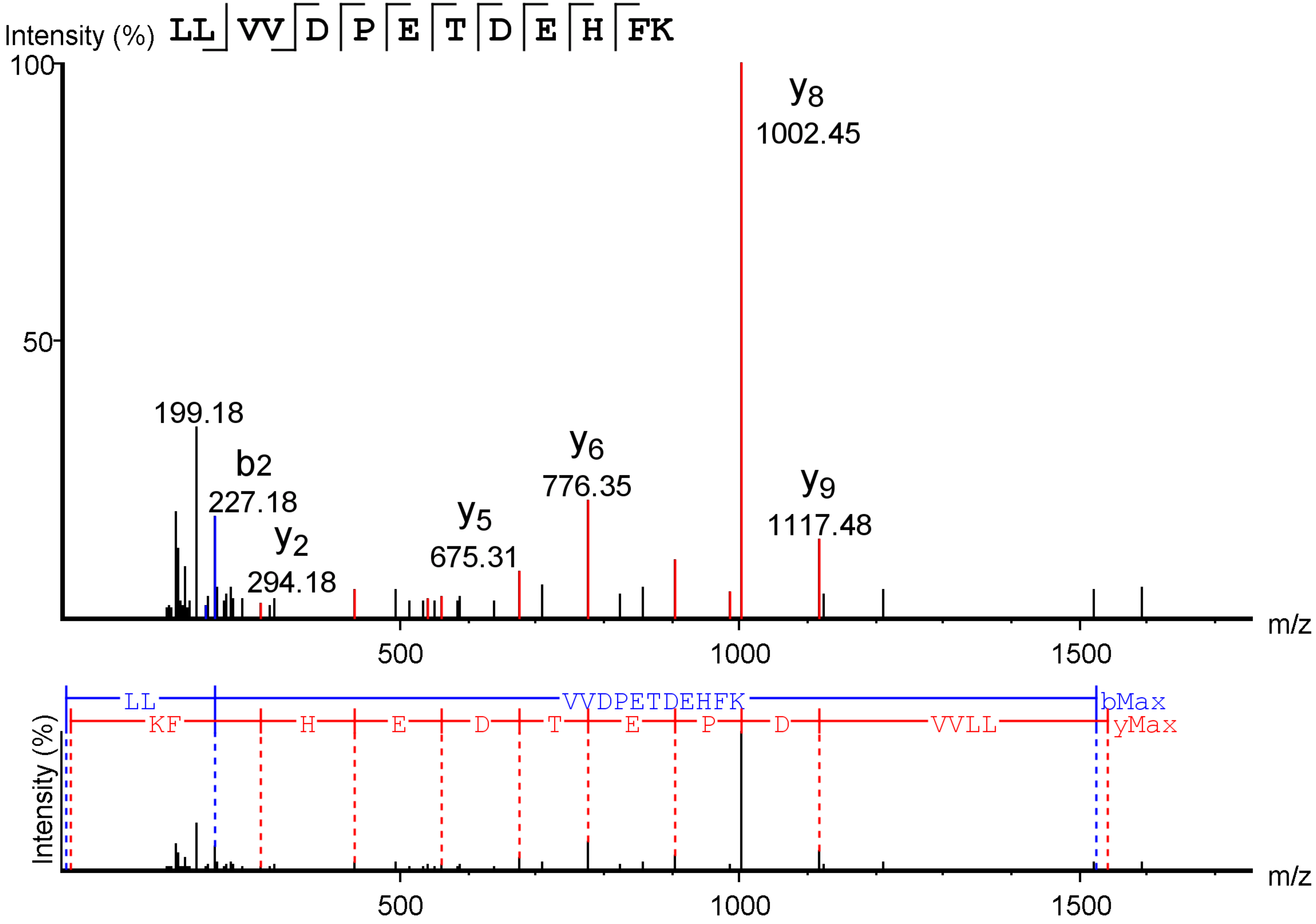

sp|P32004|L1CAM_HUMAN
R.LVLSDLHLLTQSQVR.V

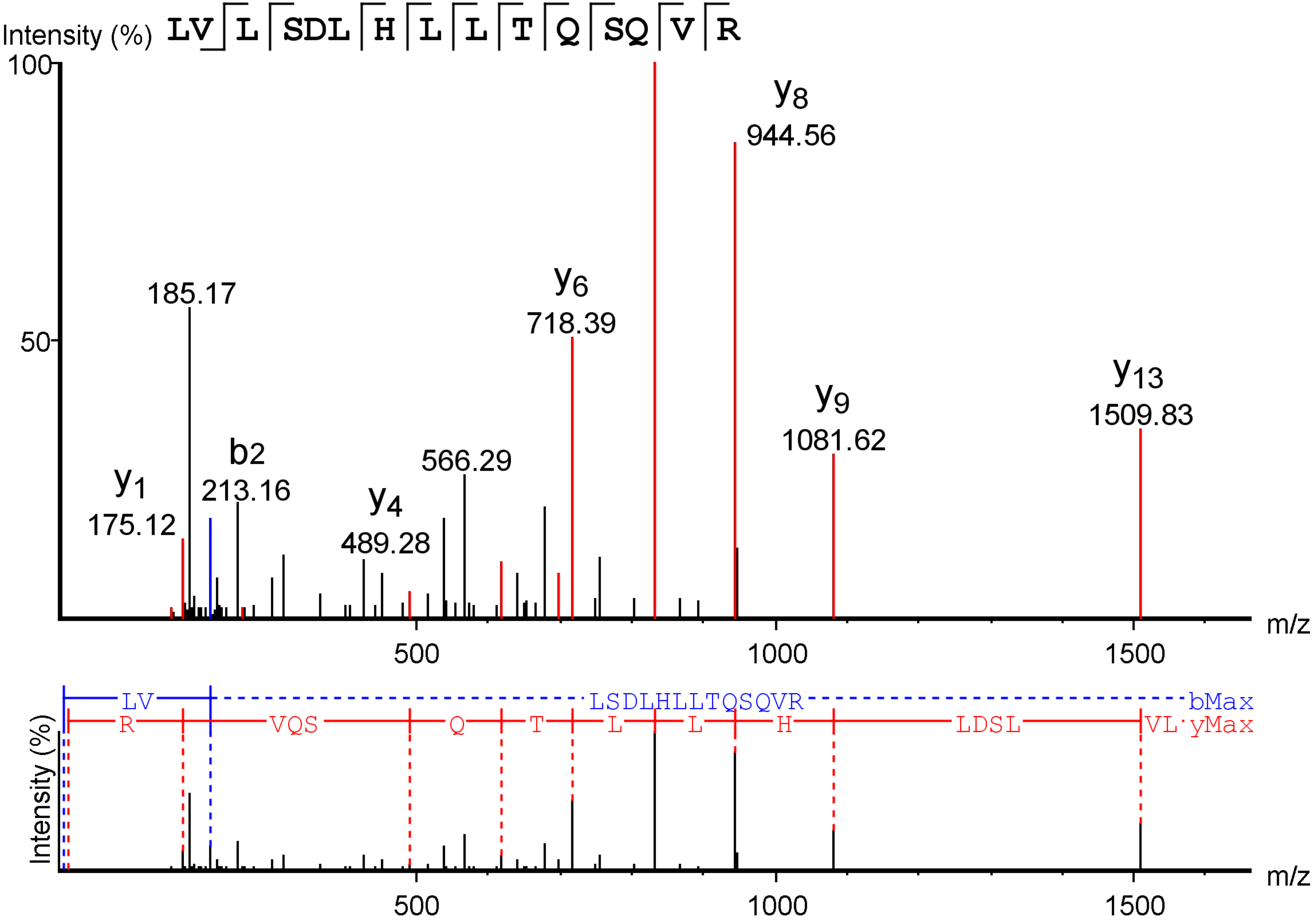

sp|P43146|DCC_HUMAN
R.VVVLPSGALQISR.L

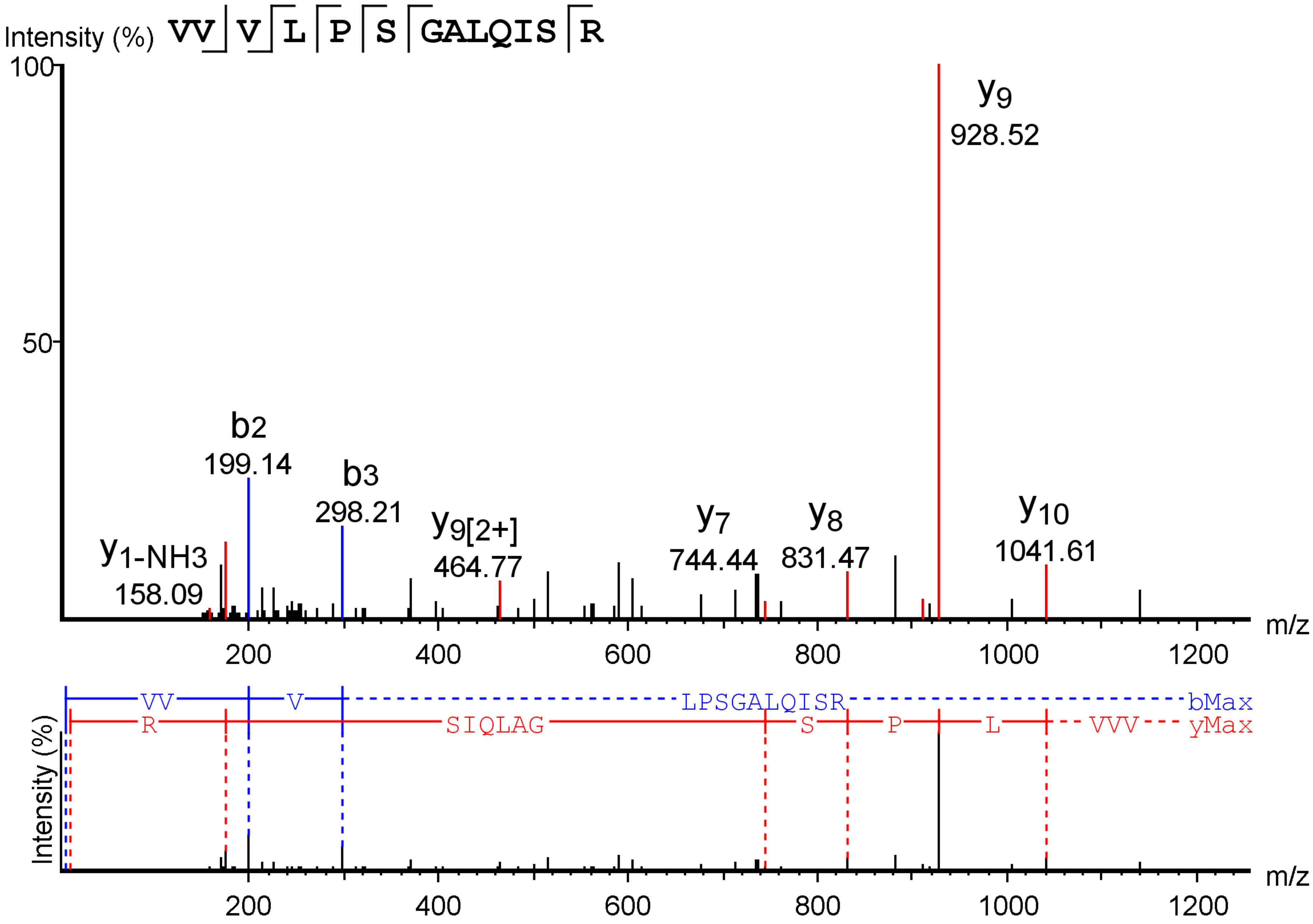

sp|P00734|THRB_HUMAN
R.SGIEC(+57.02)QLWR.S

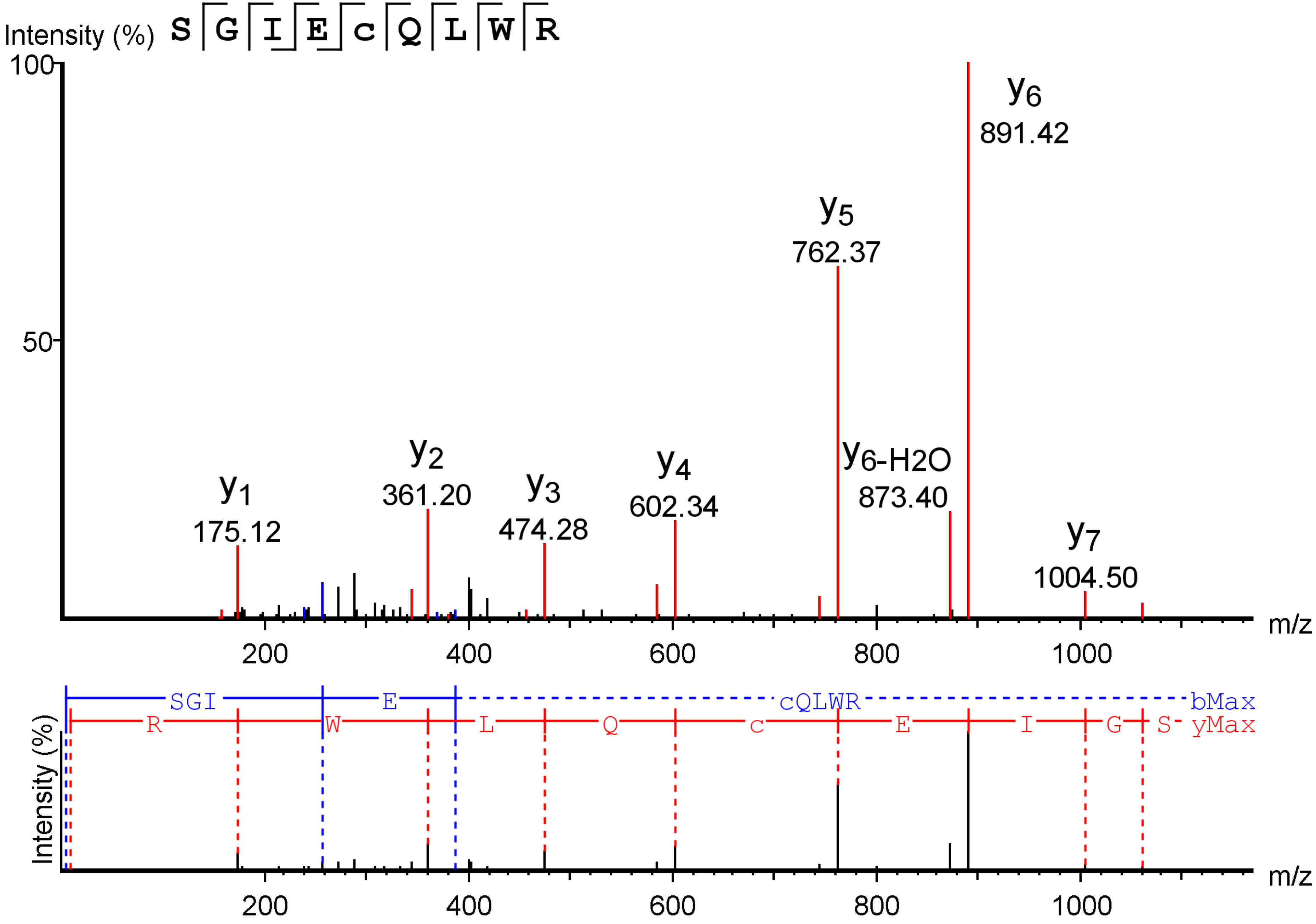

sp|Q96P70|IPO9_HUMAN
M.A(+42.01)AAAAAGAASGLPGPVAQGLK.E

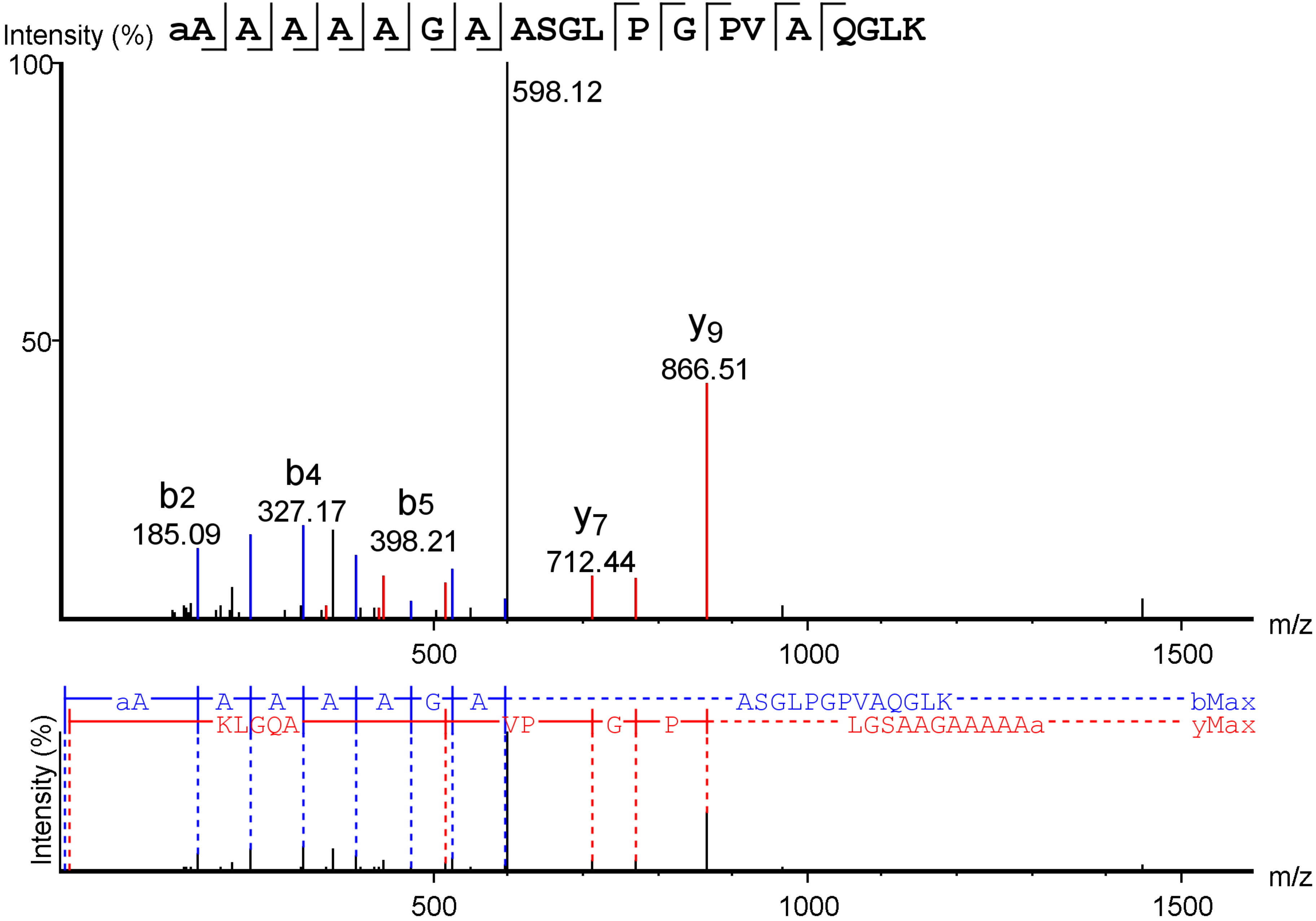

sp|P53985|MOT1_HUMAN
K.EEETSIDVAGKPNEVTK.A

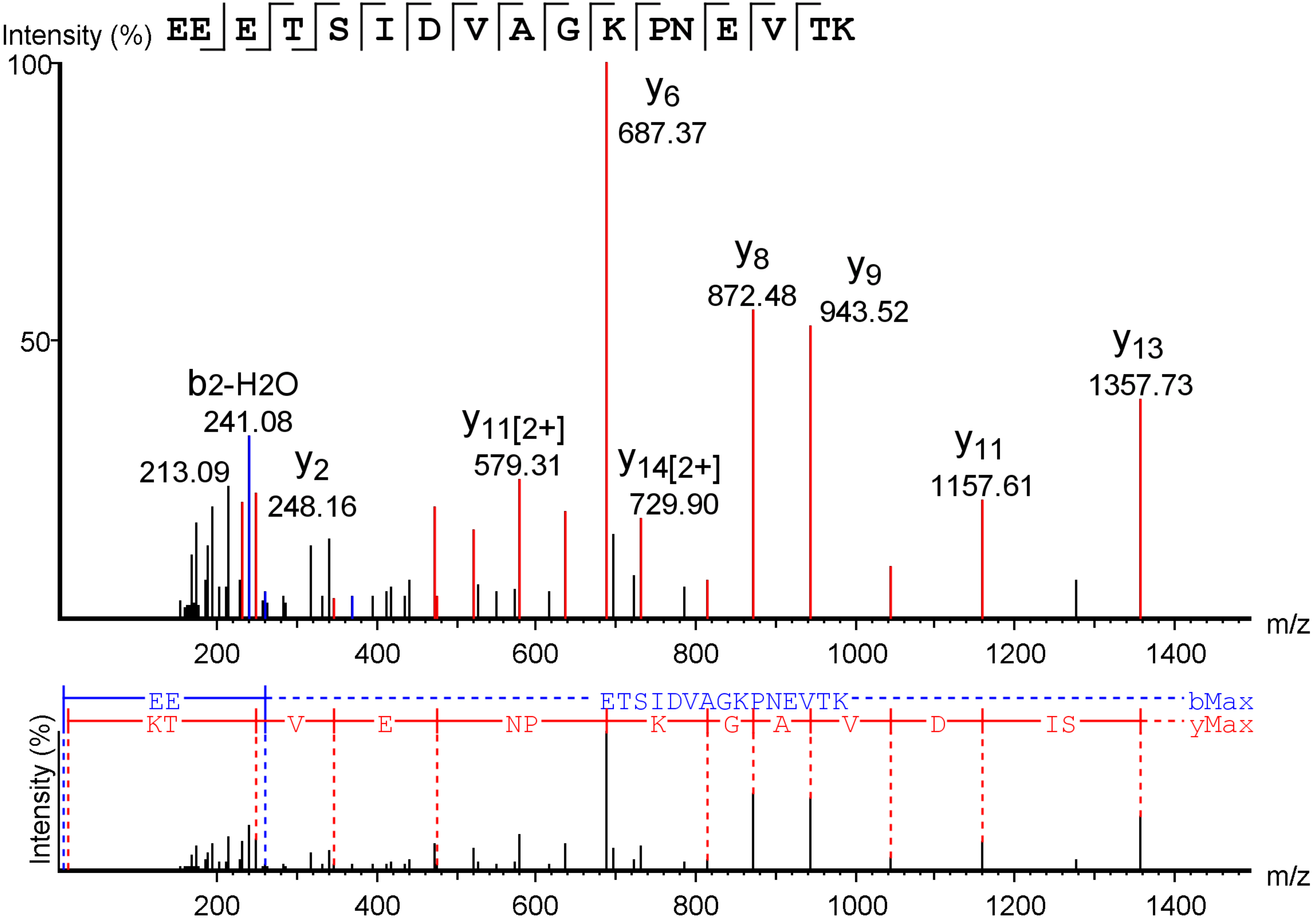

sp|Q24JP5|T132A_HUMAN
R.SPLSDSILGEQALAVTDDK.V

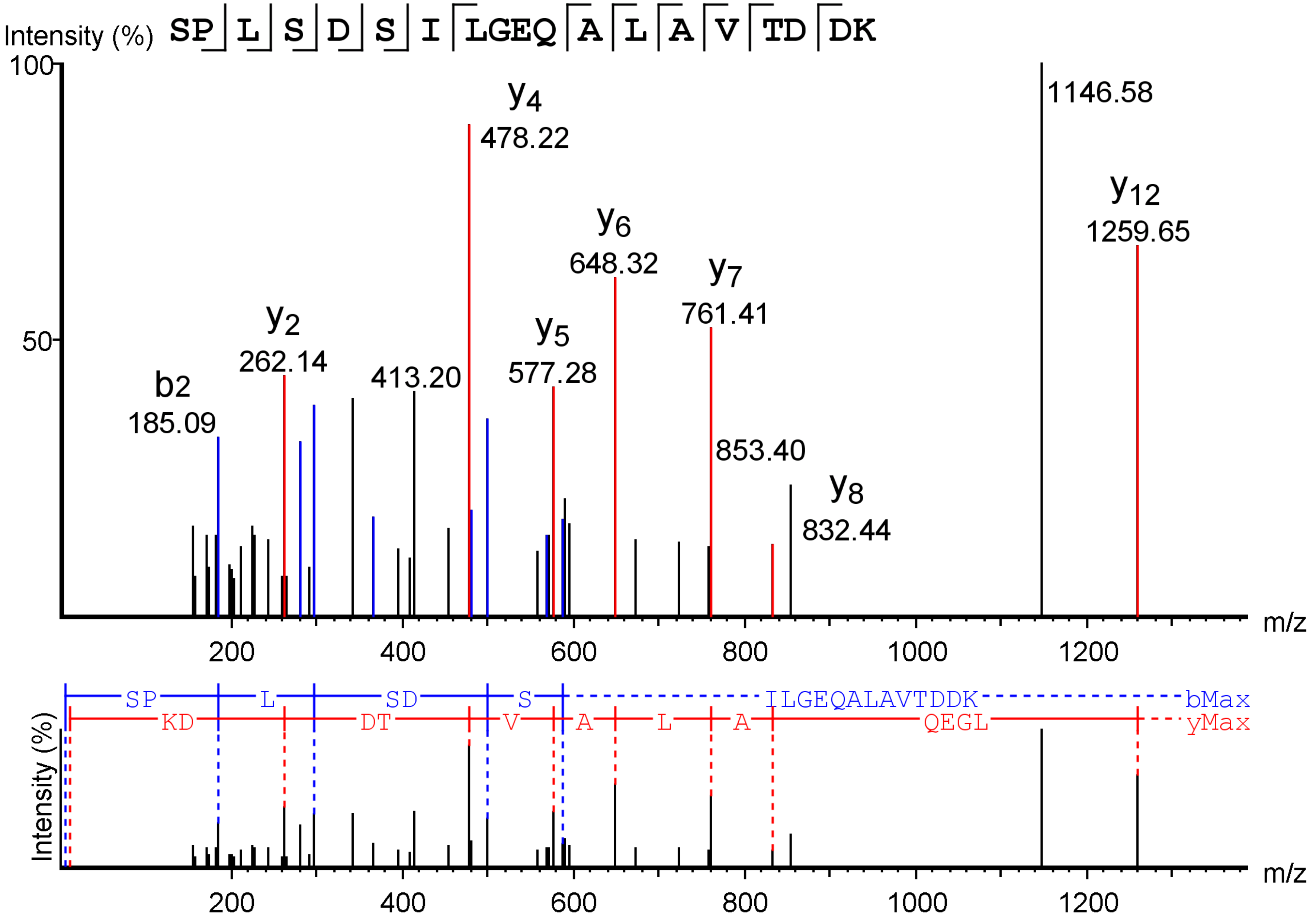

sp|P62993|GRB2_HUMAN
K.ATADDELSFK.R

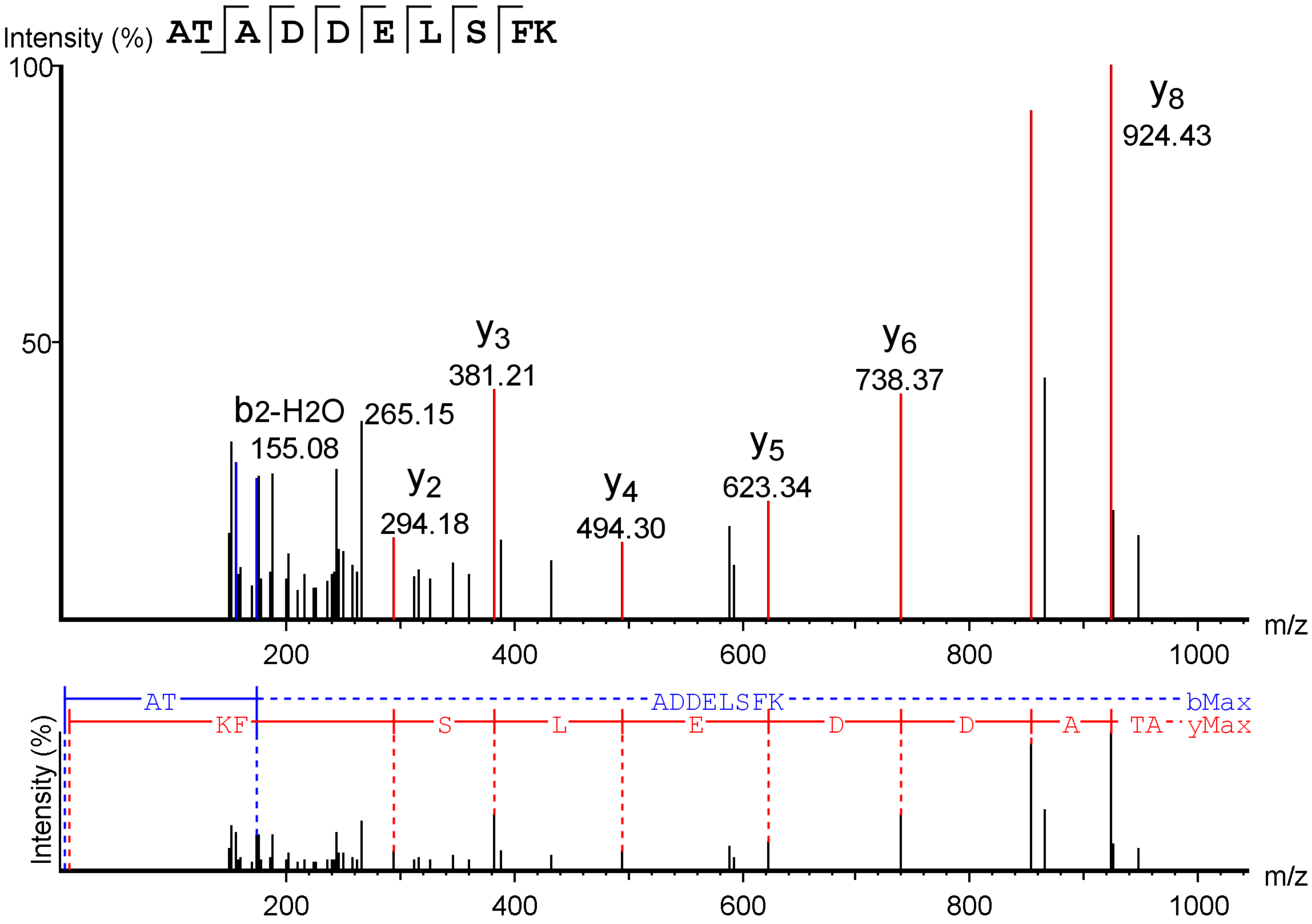

sp|P00387|NB5R3_HUMAN
R.STPAITLESPDIKYPLR.L

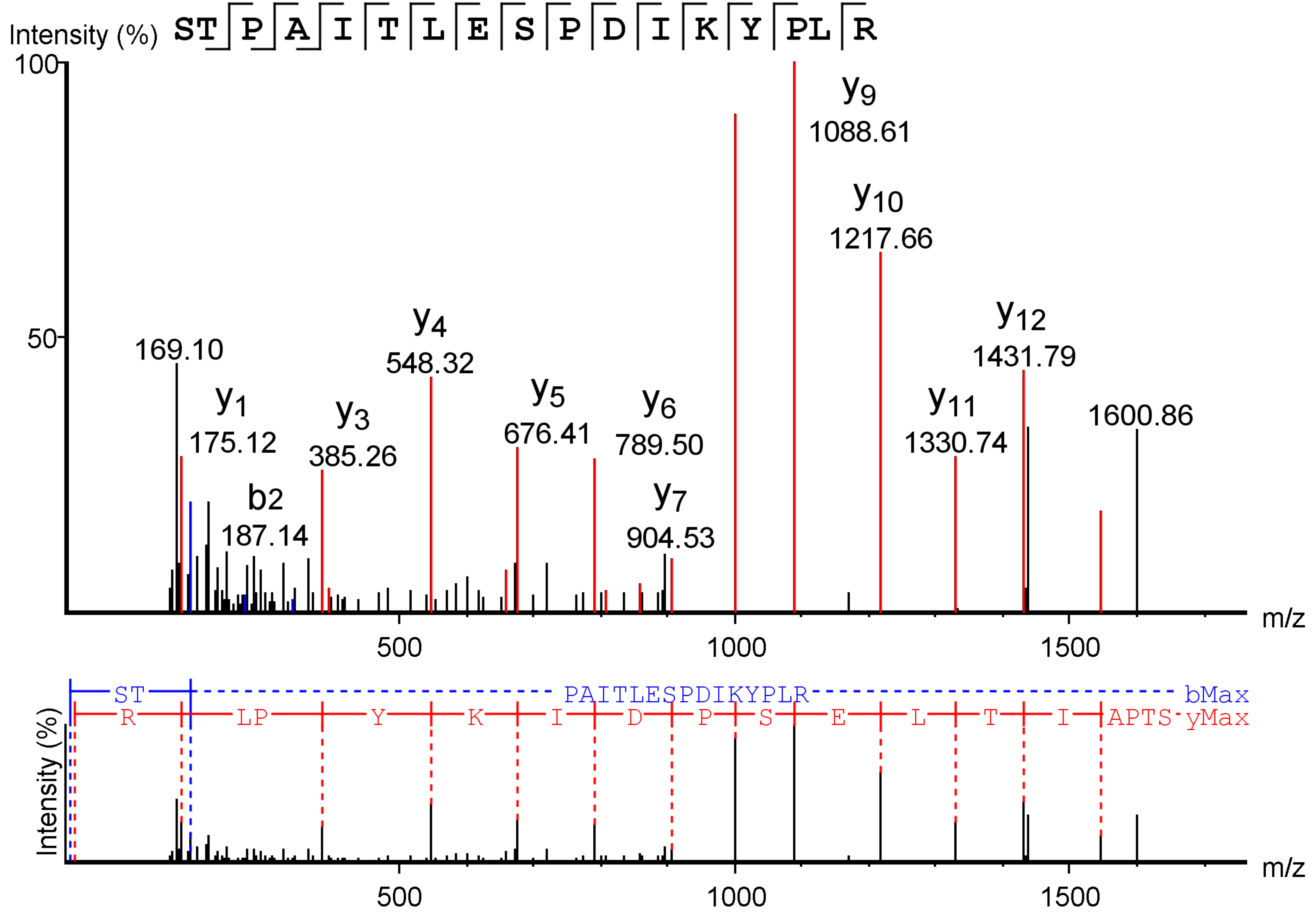

sp|P54920|SNAA_HUMAN
K.NSQSFFSGLFGGSSK.I

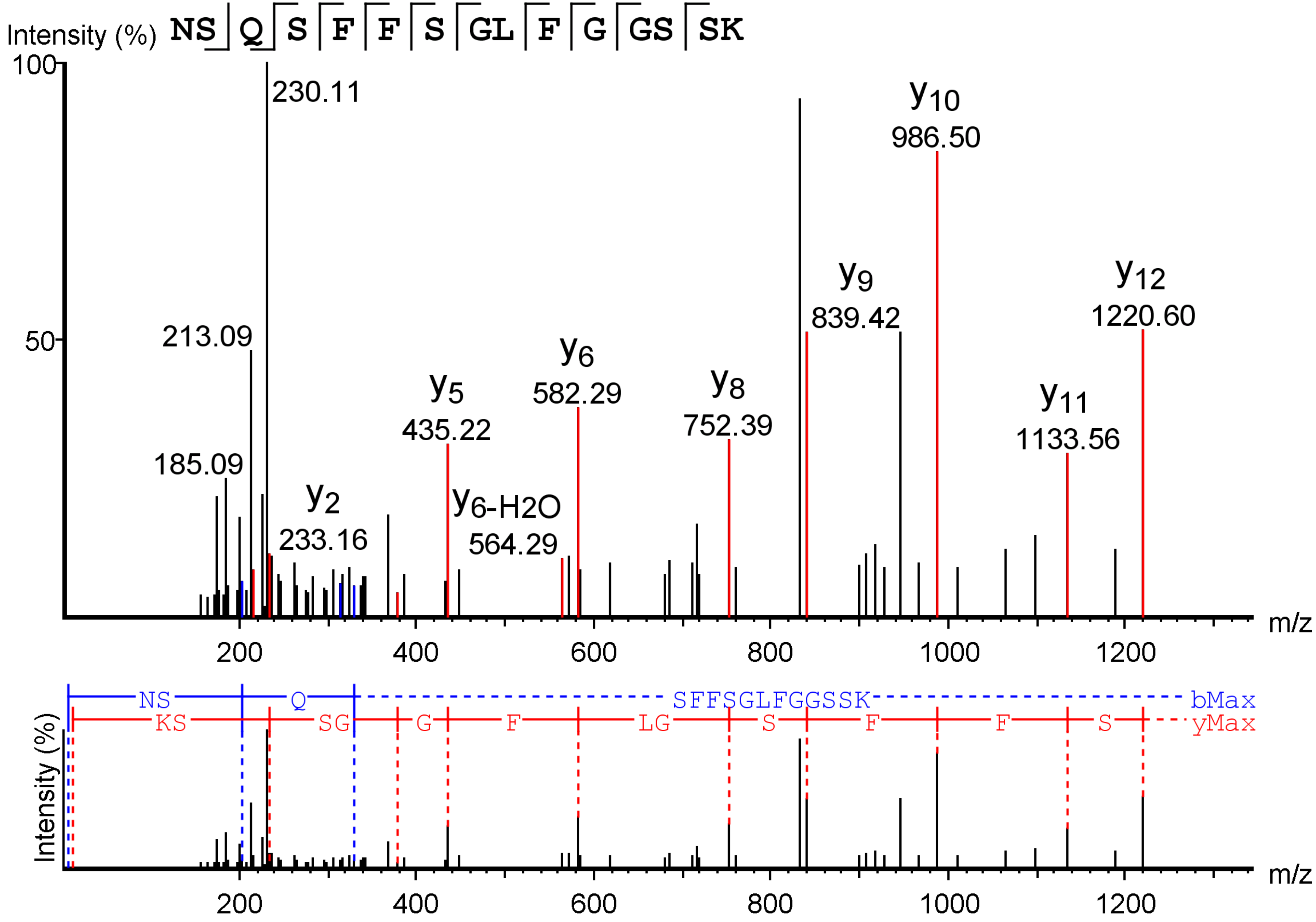

sp|Q9NUI1|DECR2_HUMAN
K.VAFITGGGSGIGFR.I

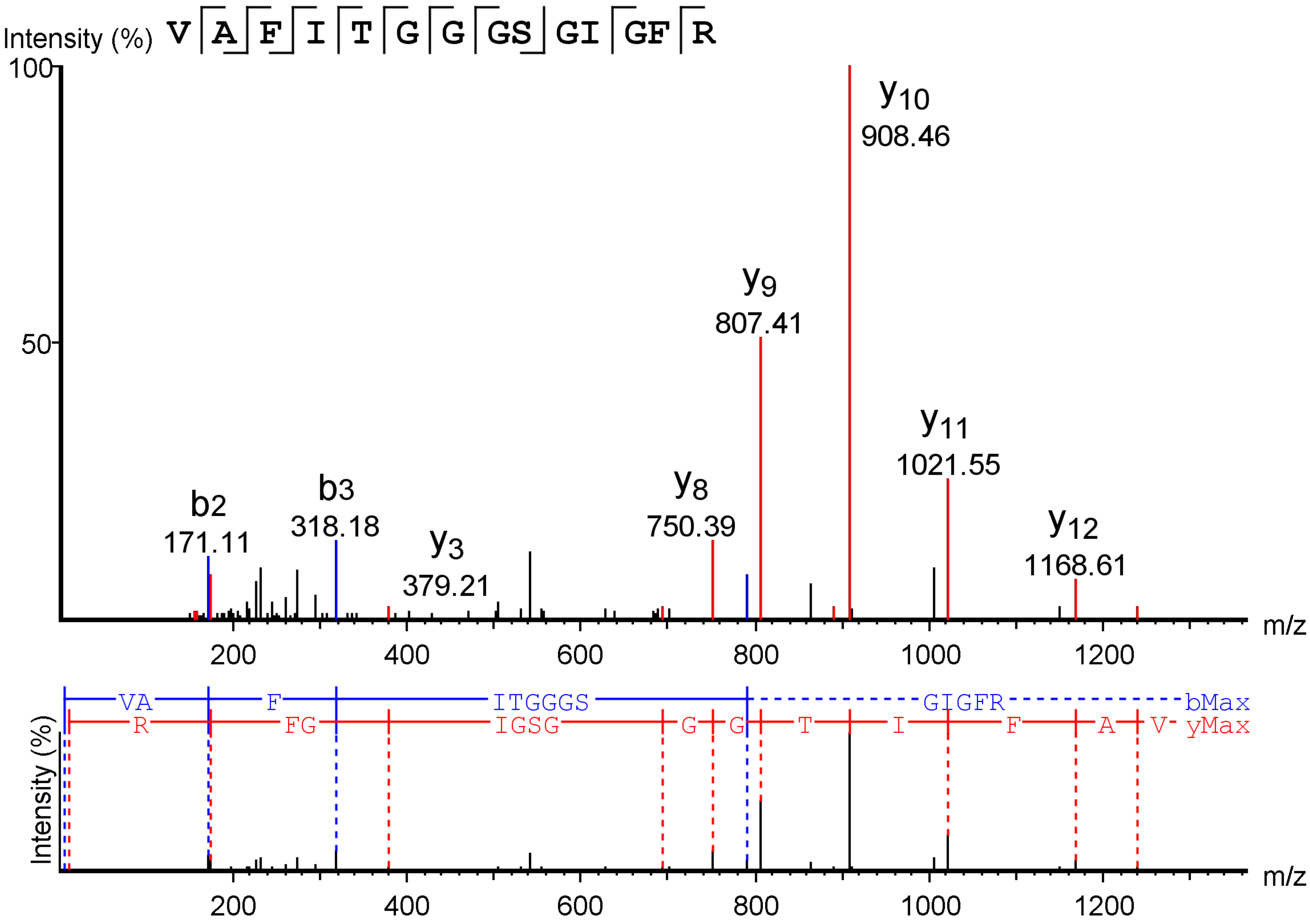

sp|O75822|EIF3J_HUMAN
K.VLTPEEQLADKLR.L

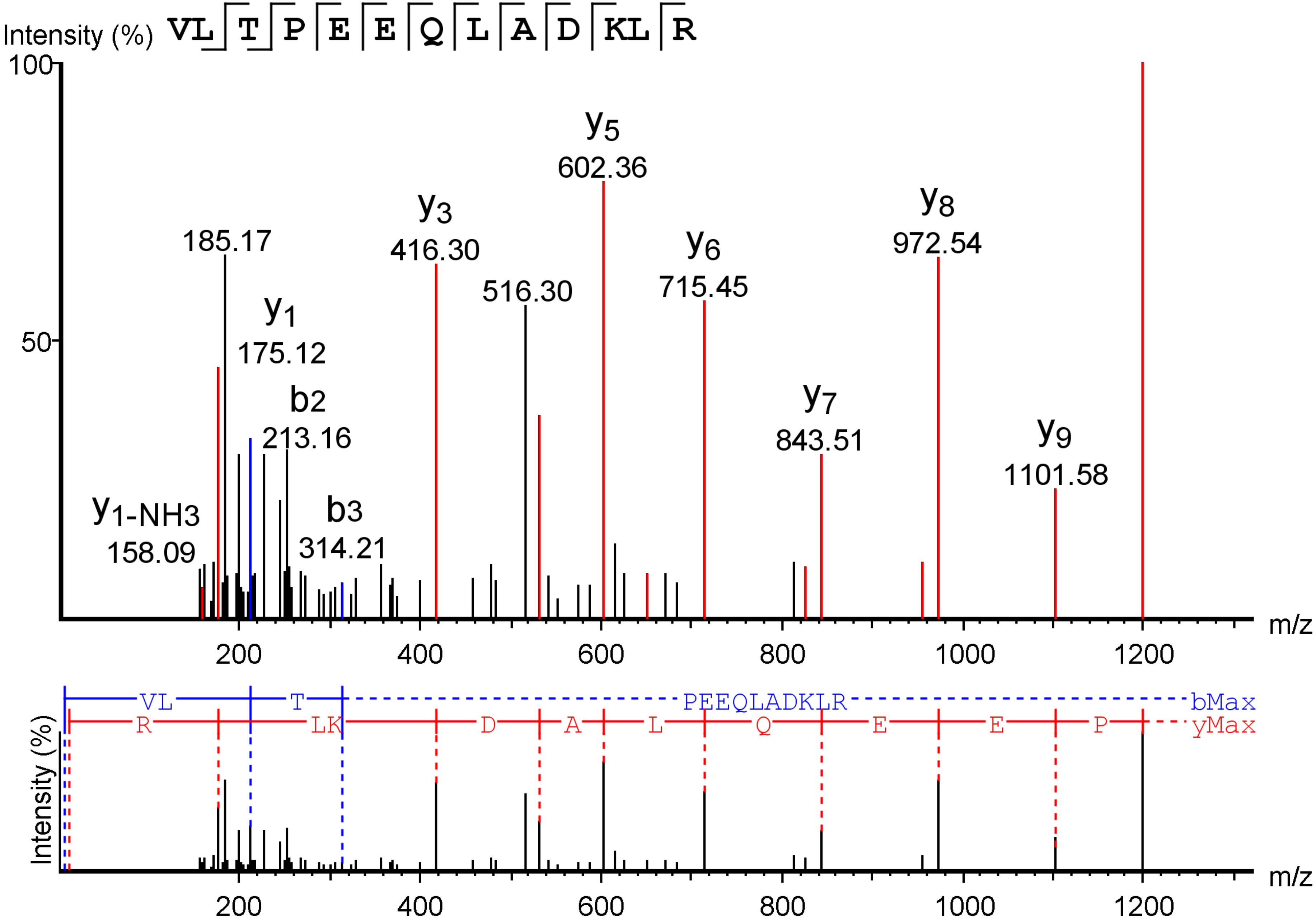

sp|P04792|HSPB1_HUMAN
R.VSLDVNHFAPDELTVK.T

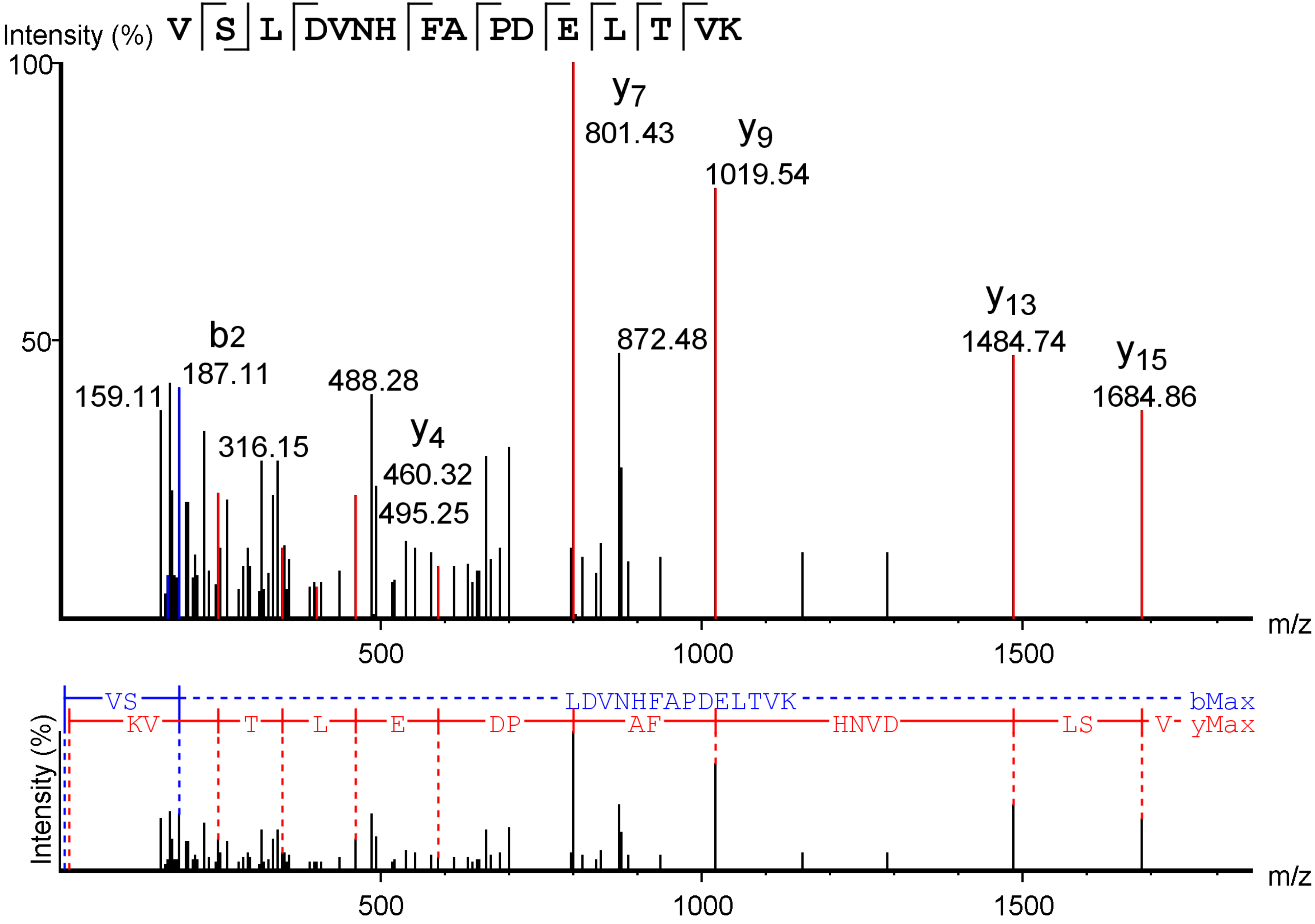

sp|P19367|HXK1_HUMAN
K.KLPVGFTFSFPC(+57.02)QQSK.I

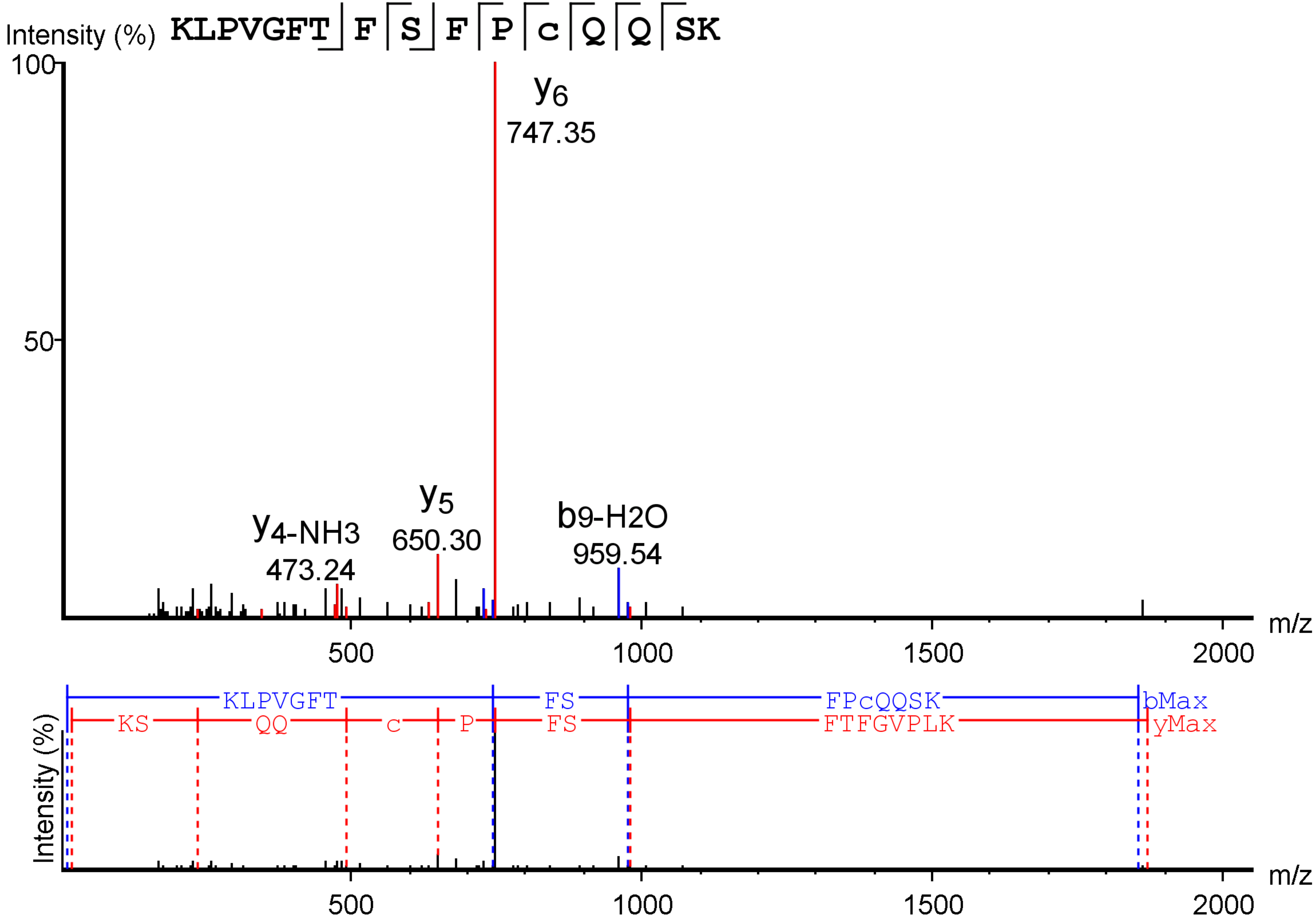

sp|Q96M86|DNHD1_HUMAN
K.LEDM(+15.99)R.G

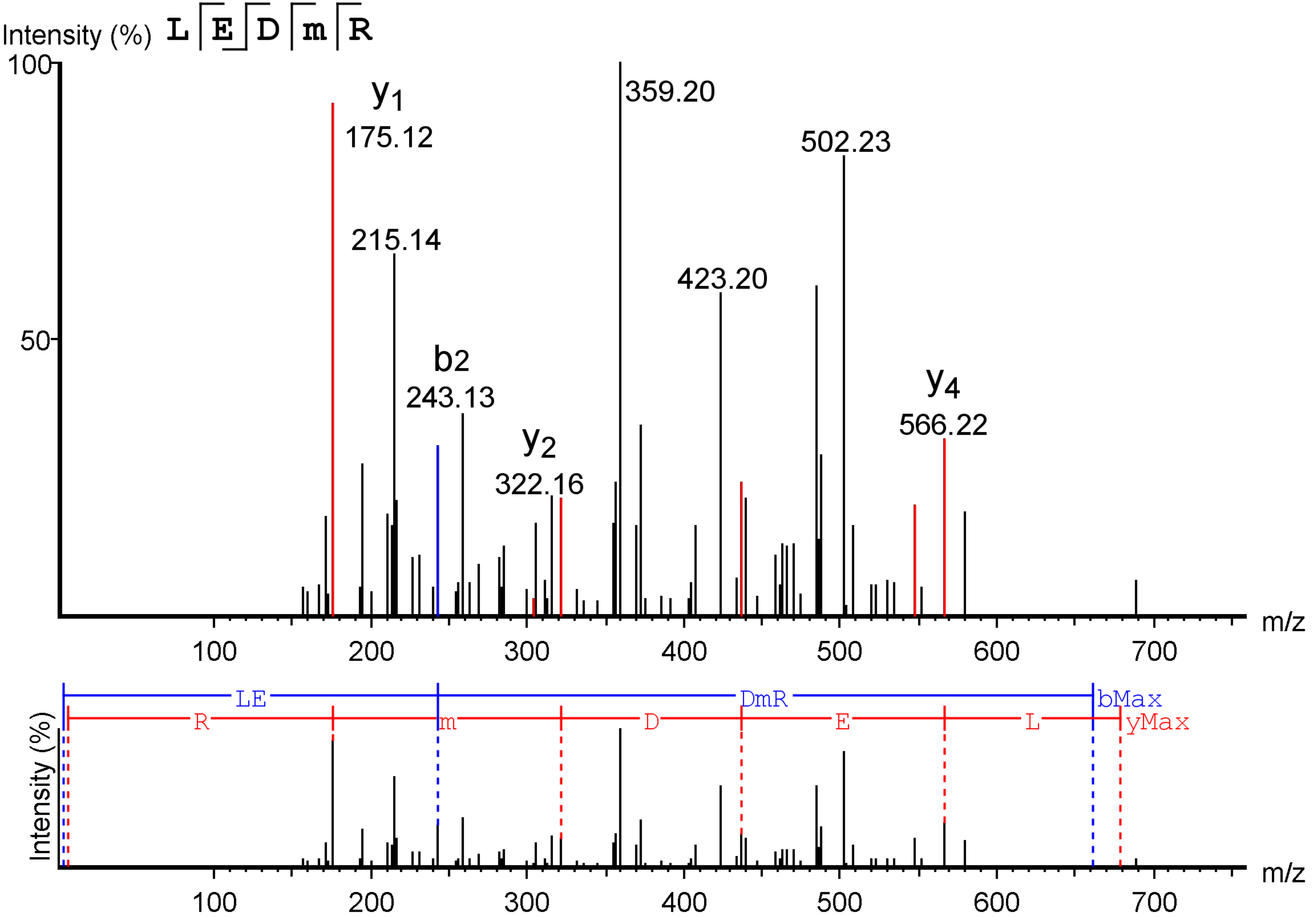

sp|O15240|VGF_HUMAN
K.AYQGVAAPFPK.A

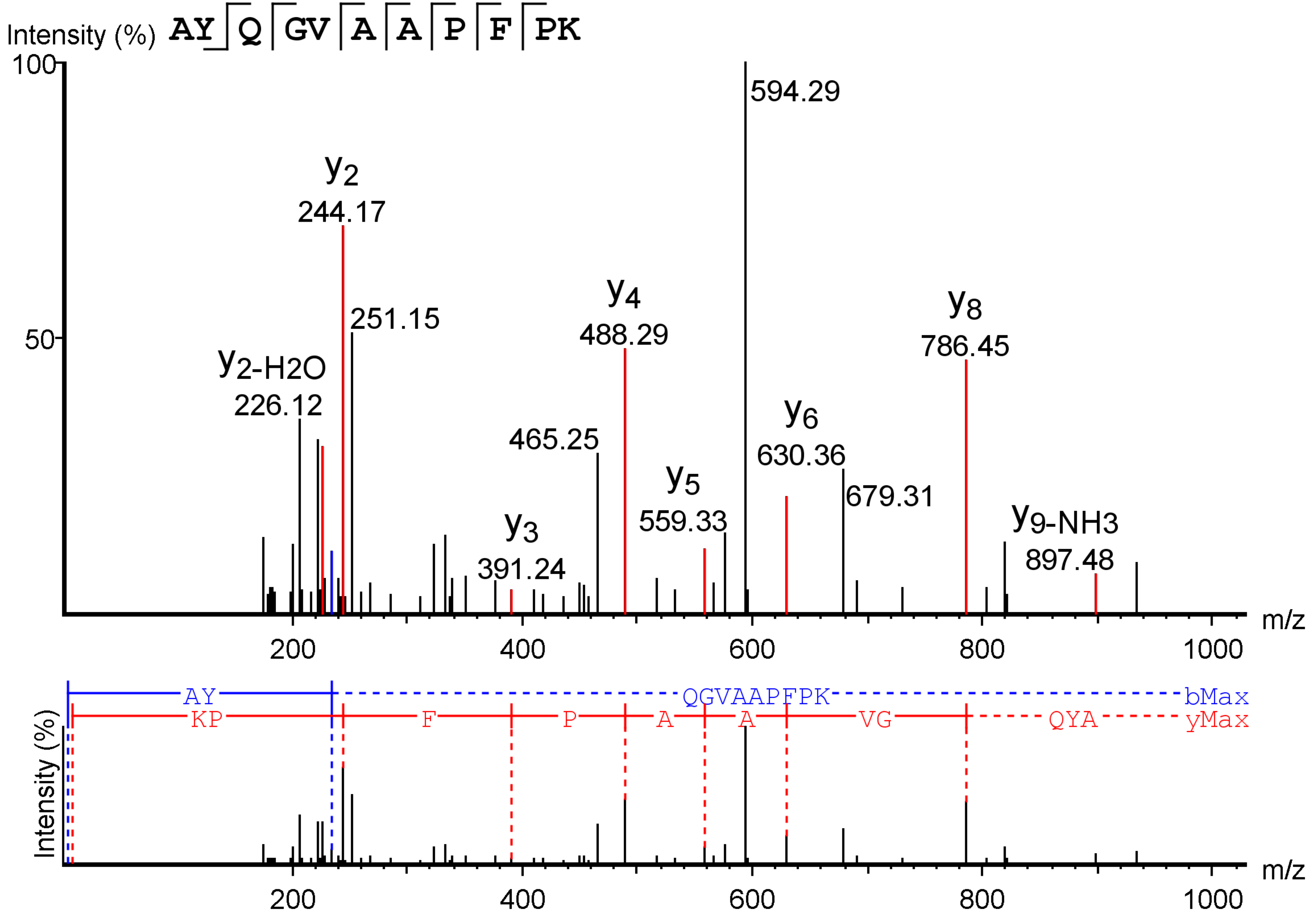

sp|Q7Z3B1|NEGR1_HUMAN
K.VVVNFAPTIQEIK.S

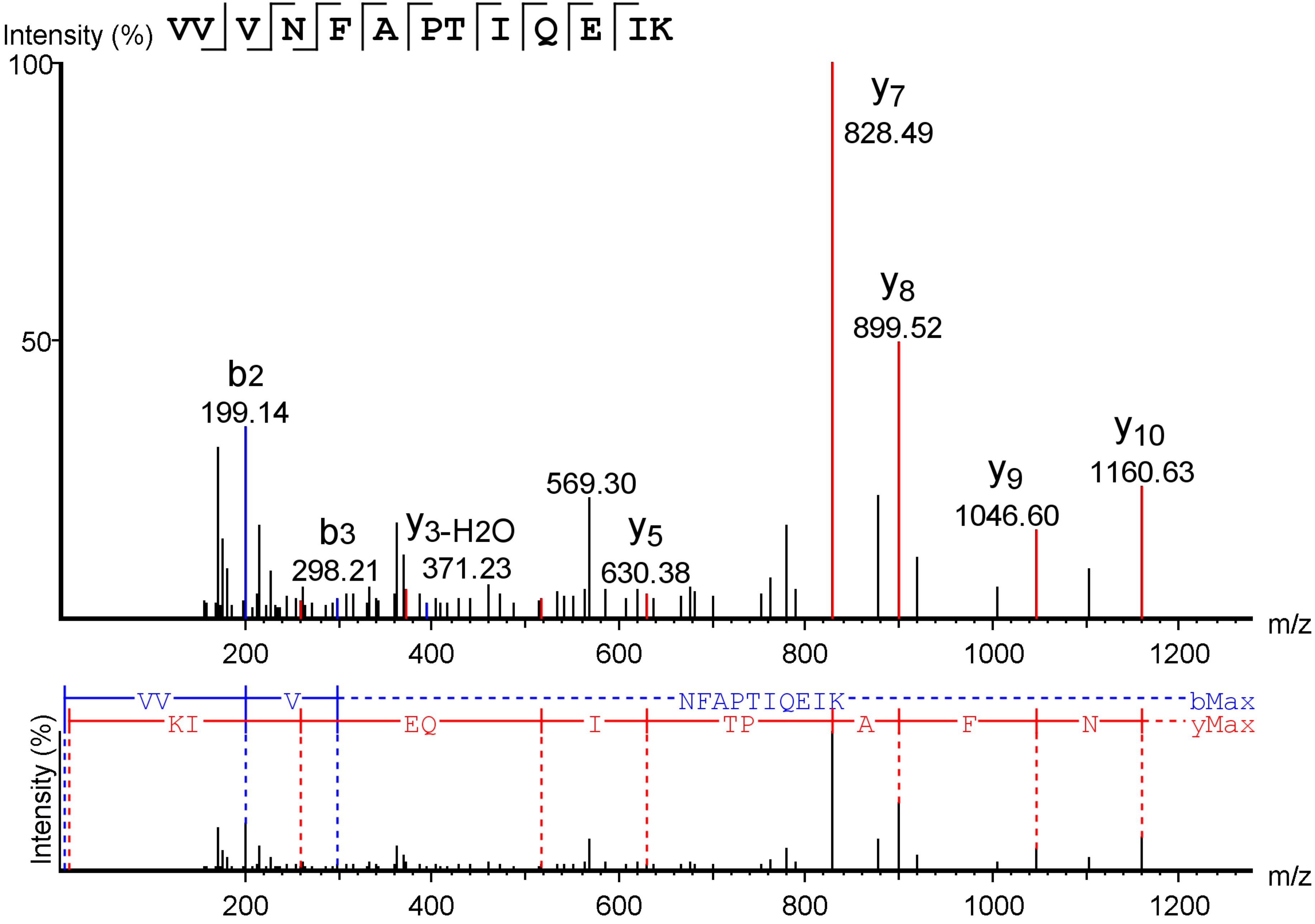

sp|Q96AQ6|PBIP1_HUMAN
K.DSHDPLPSWAELLRPK.Y

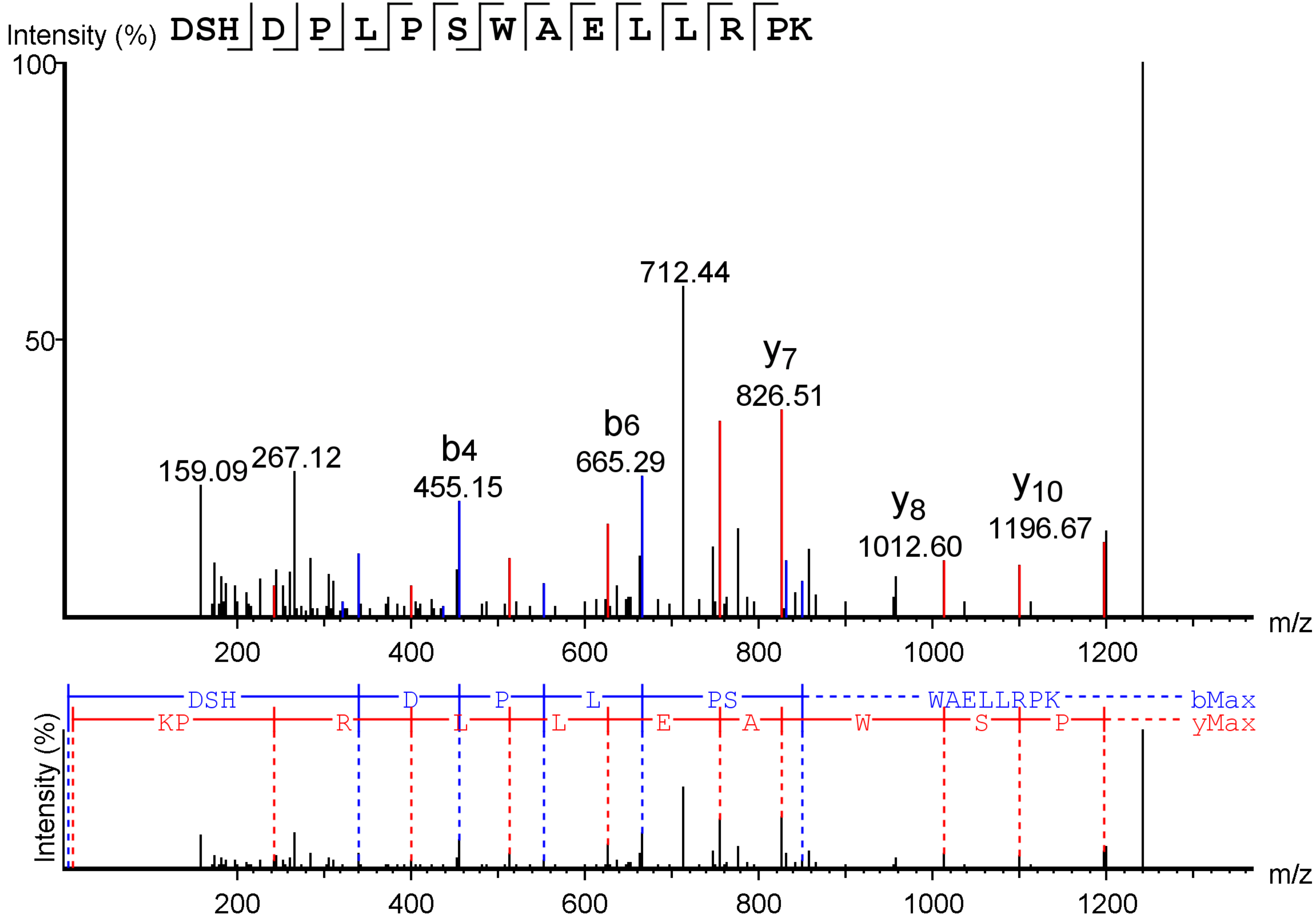

sp|A0A0C4DH38|HV551_HUMAN
K.KPGESLK.I

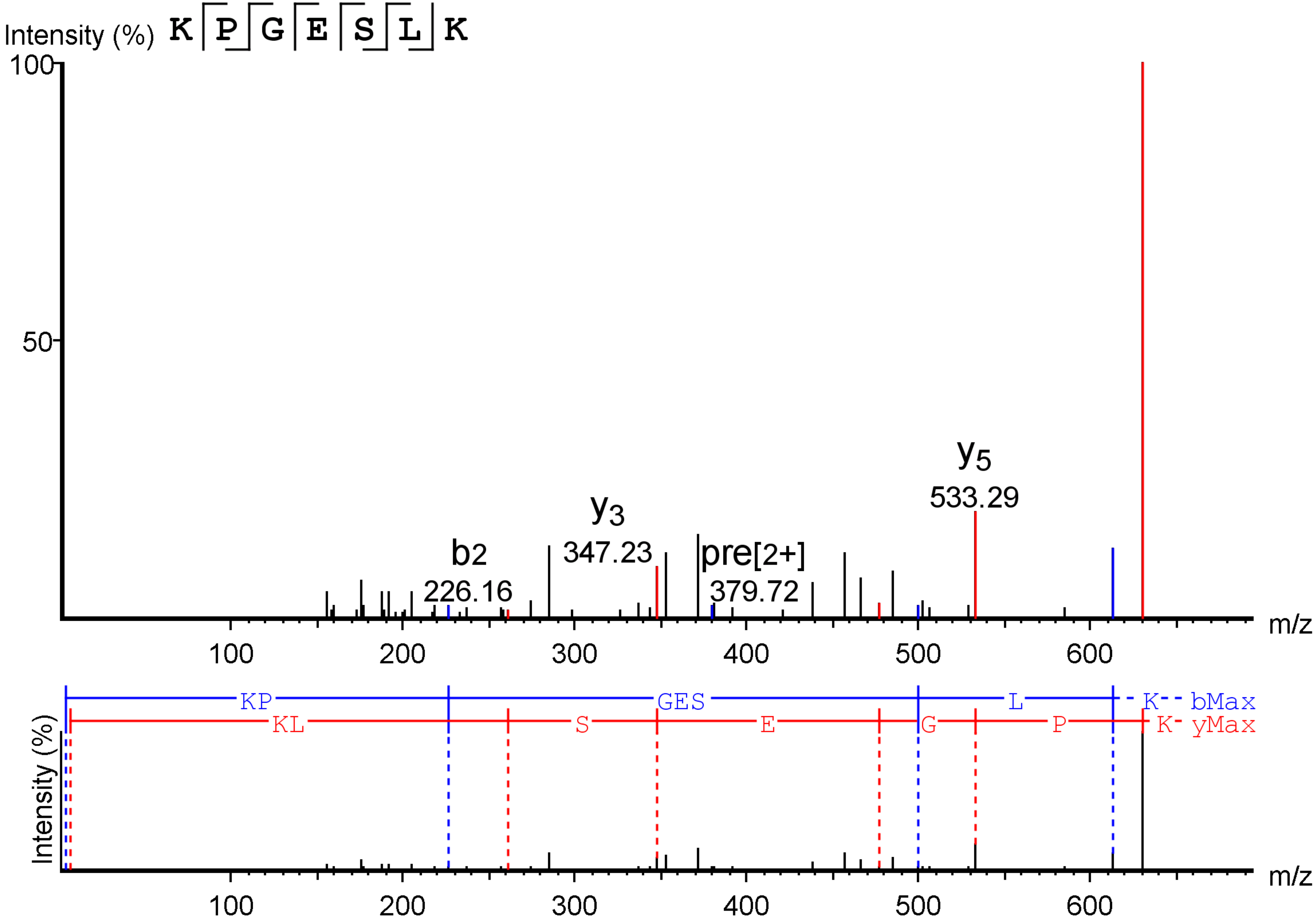

F8W8V9|F8W8V9_HUMAN

R.VS(+79.97)LGNDTRTFYQFEAAWDSSM(+15.99)HNSLLLNR.V

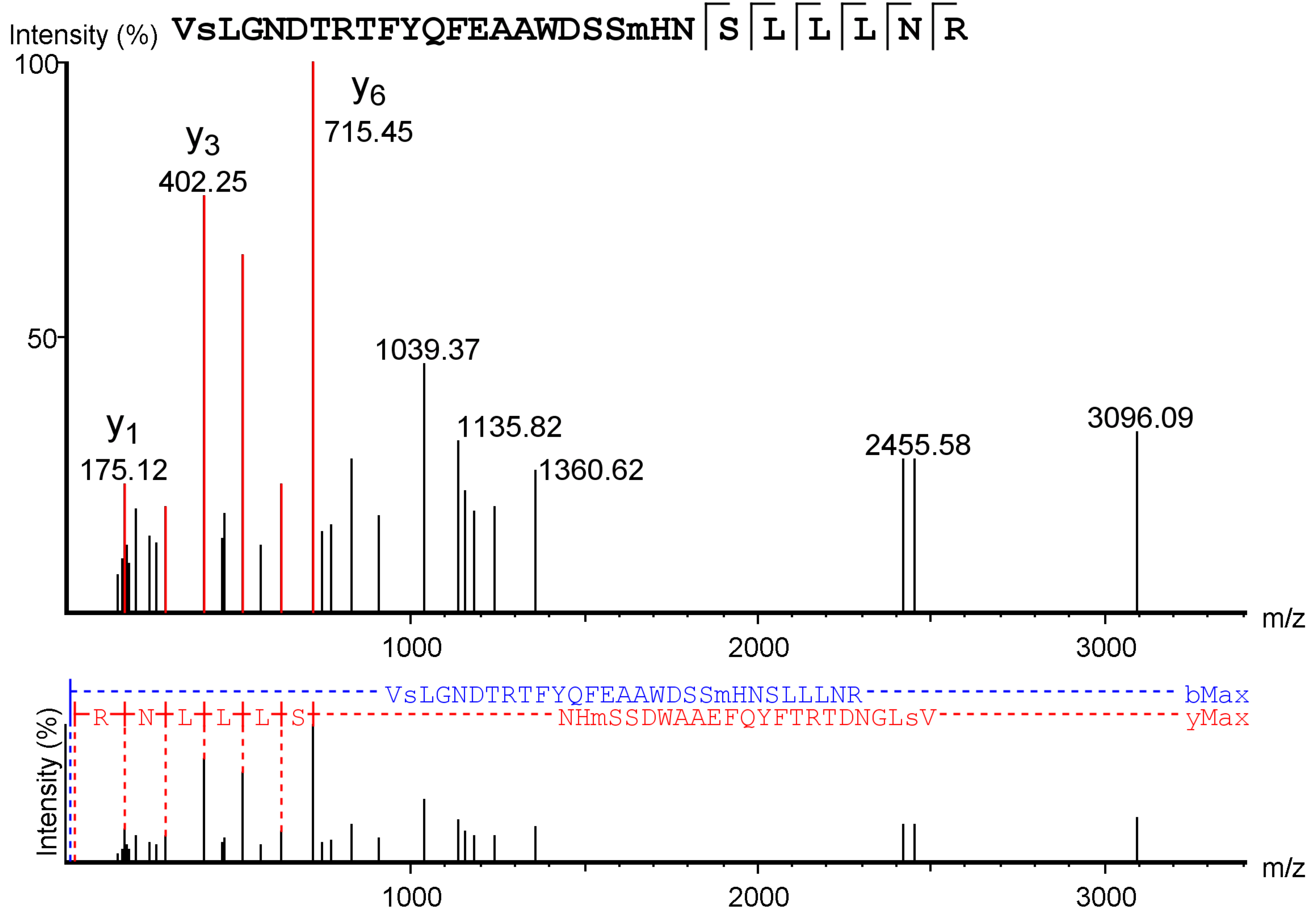

sp|Q13740|CD166_HUMAN
R.SSPSFSSLHYQDAGNYVC(+57.02)ETALQEVEGLKK.R

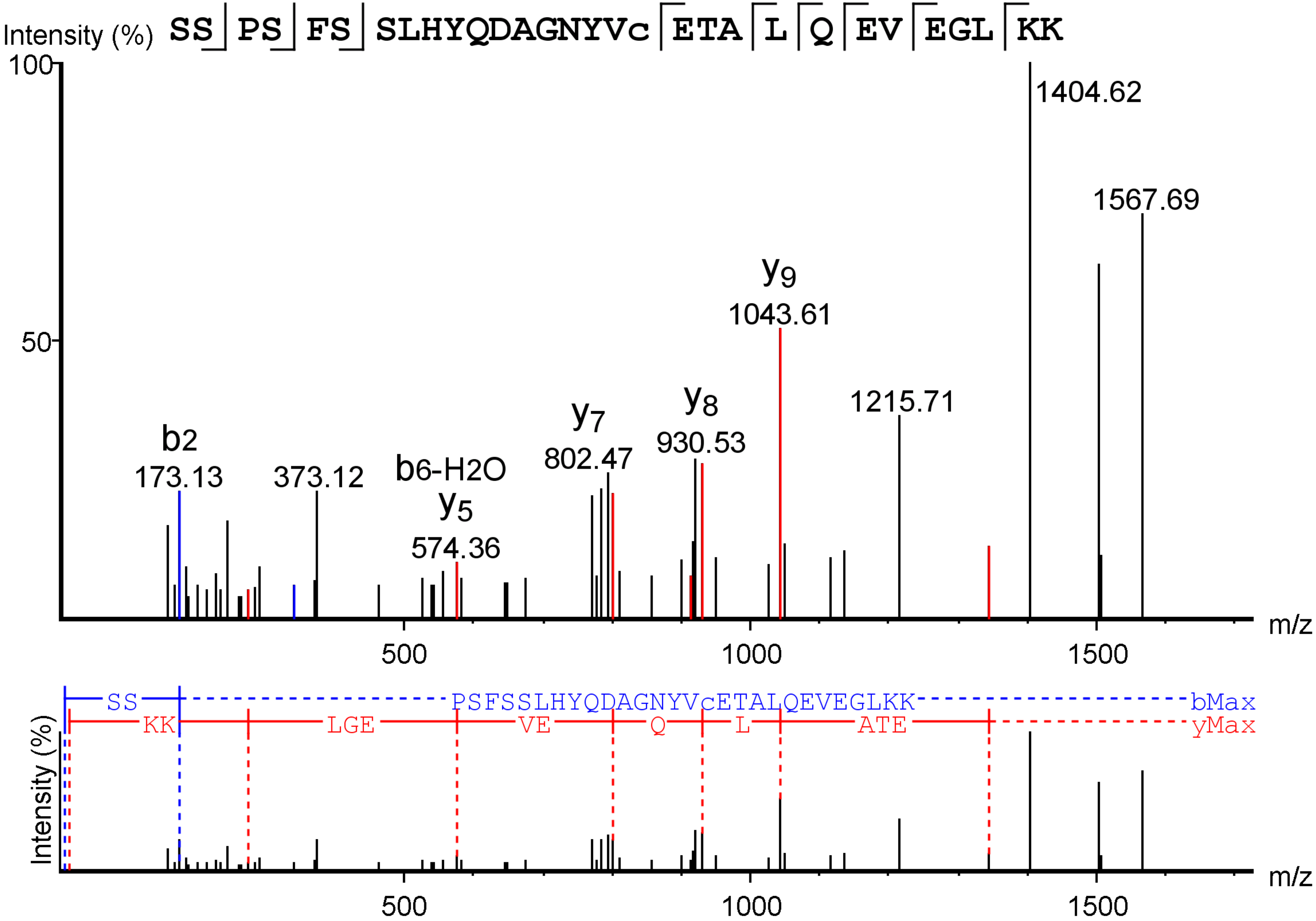

sp|P12273|PIP_HUMAN
R.ELGIC(+57.02)PDDAAVIPIK.N

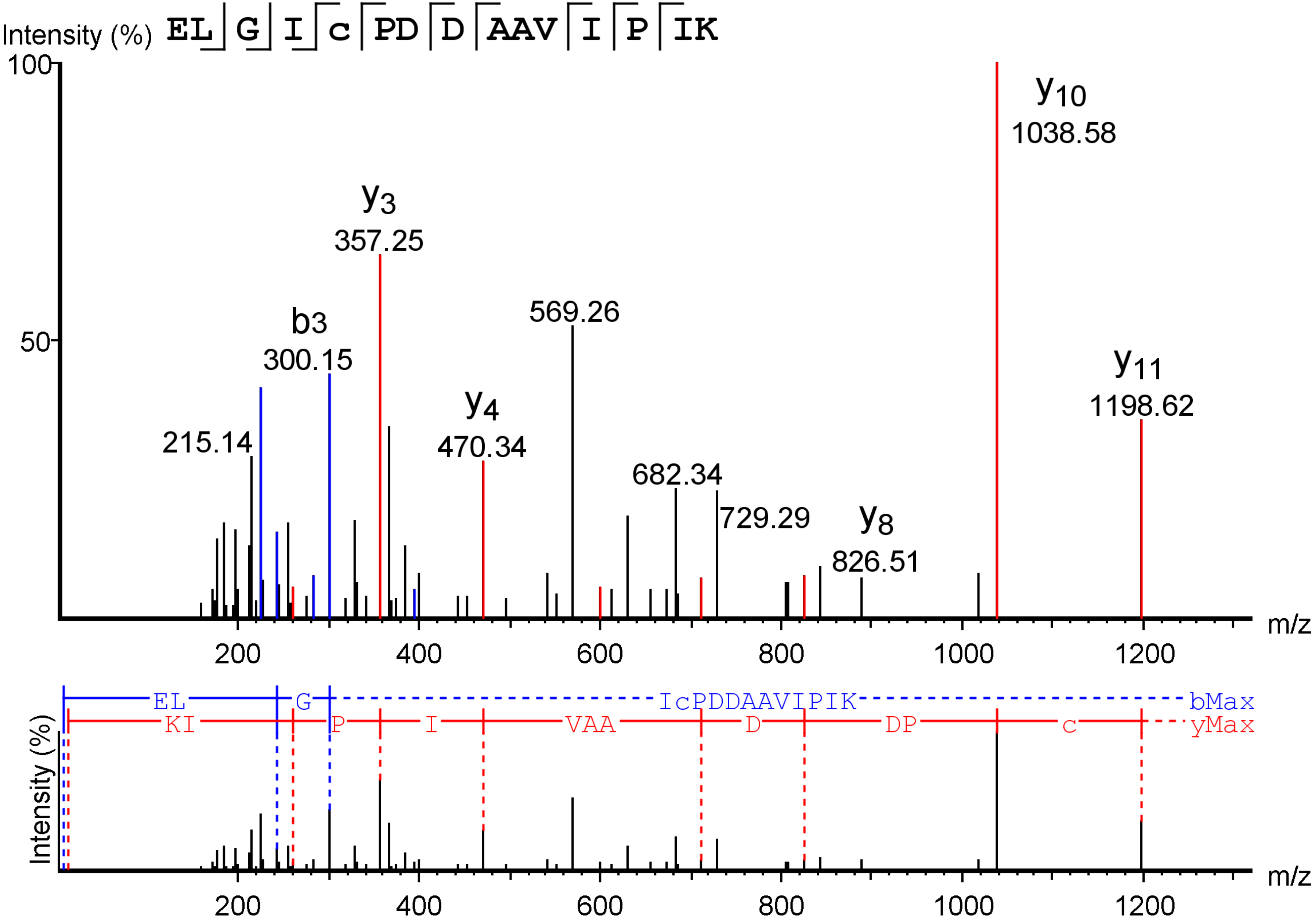

sp|Q9ULC3|RAB23_HUMAN
K.VVAEVGDIPTVLVQNK.I

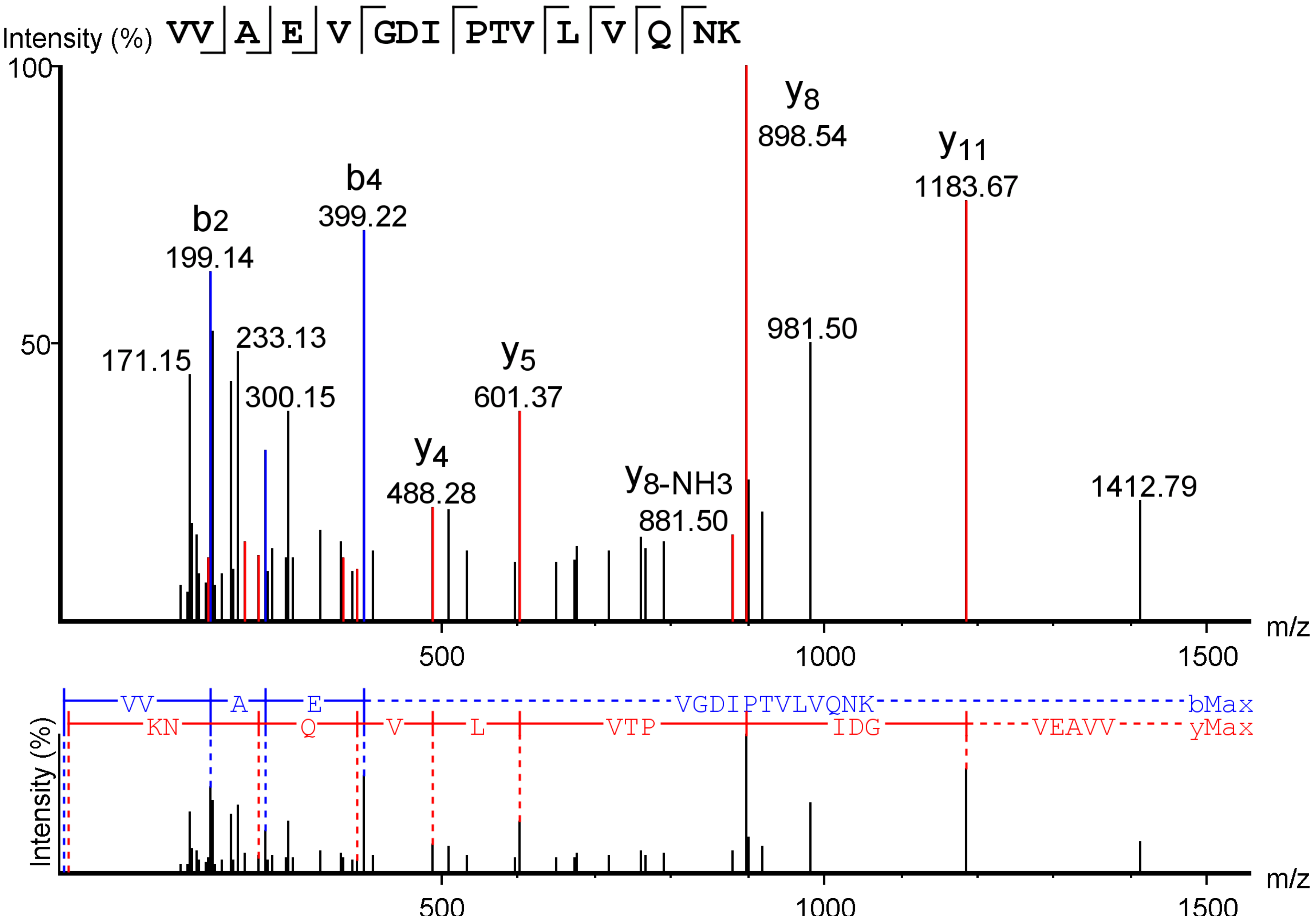

sp|P58546|MTPN_HUMAN
K.GPDGLTAFEATDNQAIK.A

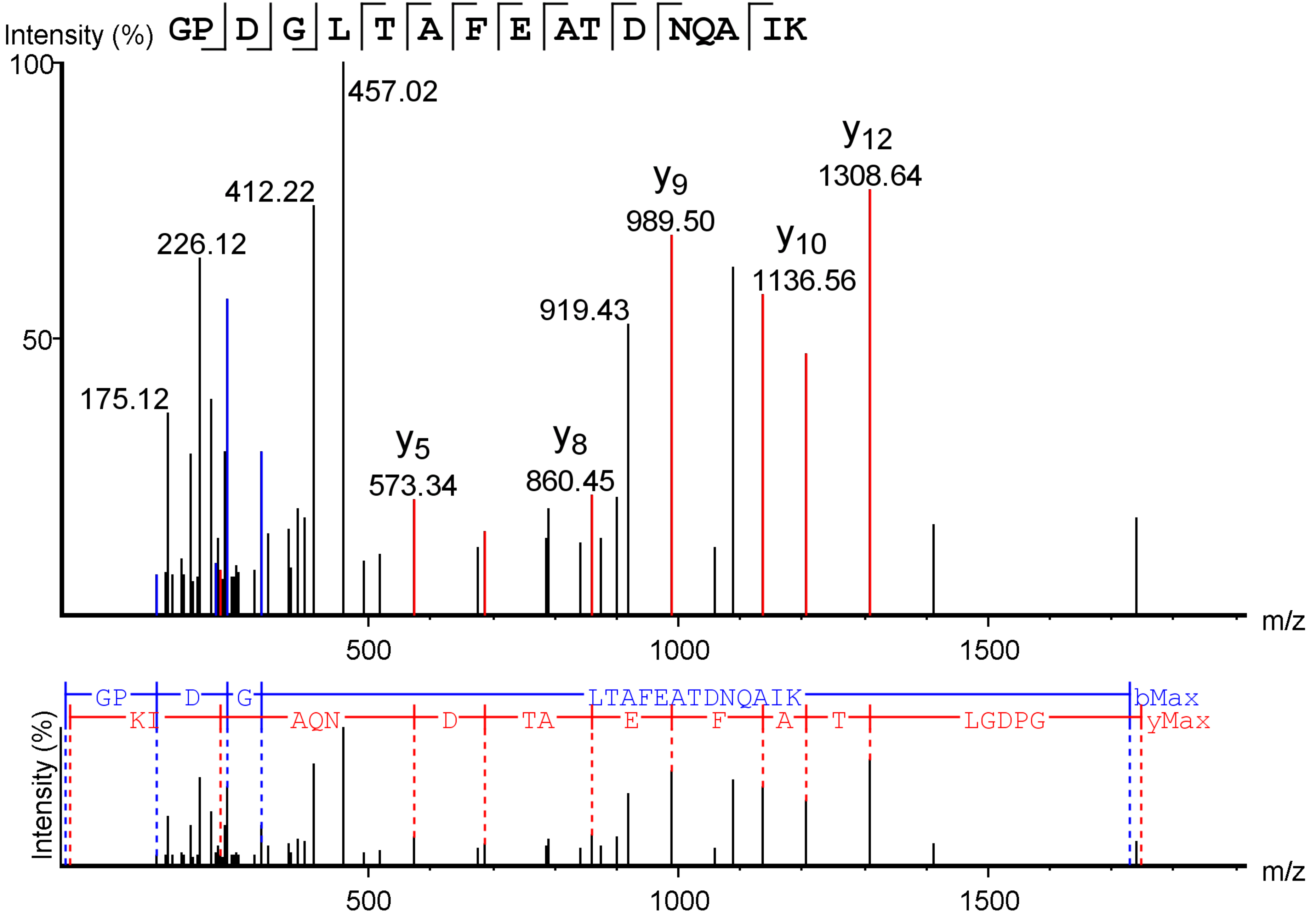

sp|P35237|SPB6_HUMAN
K.IAELLSPGSVDPLTR.L

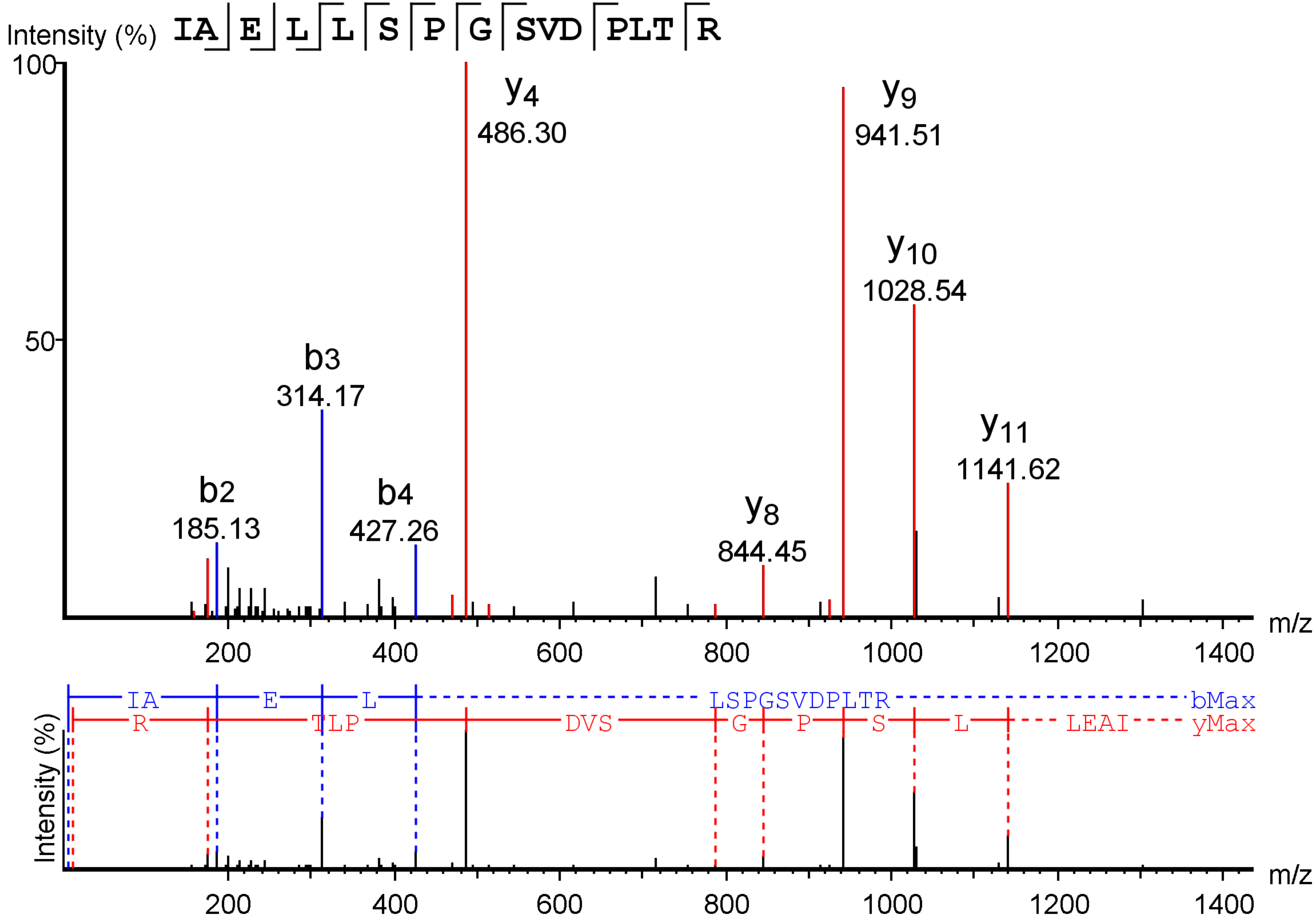

sp|Q9NR31|SAR1A_HUMAN
K.LVFLGLDNAGK.T

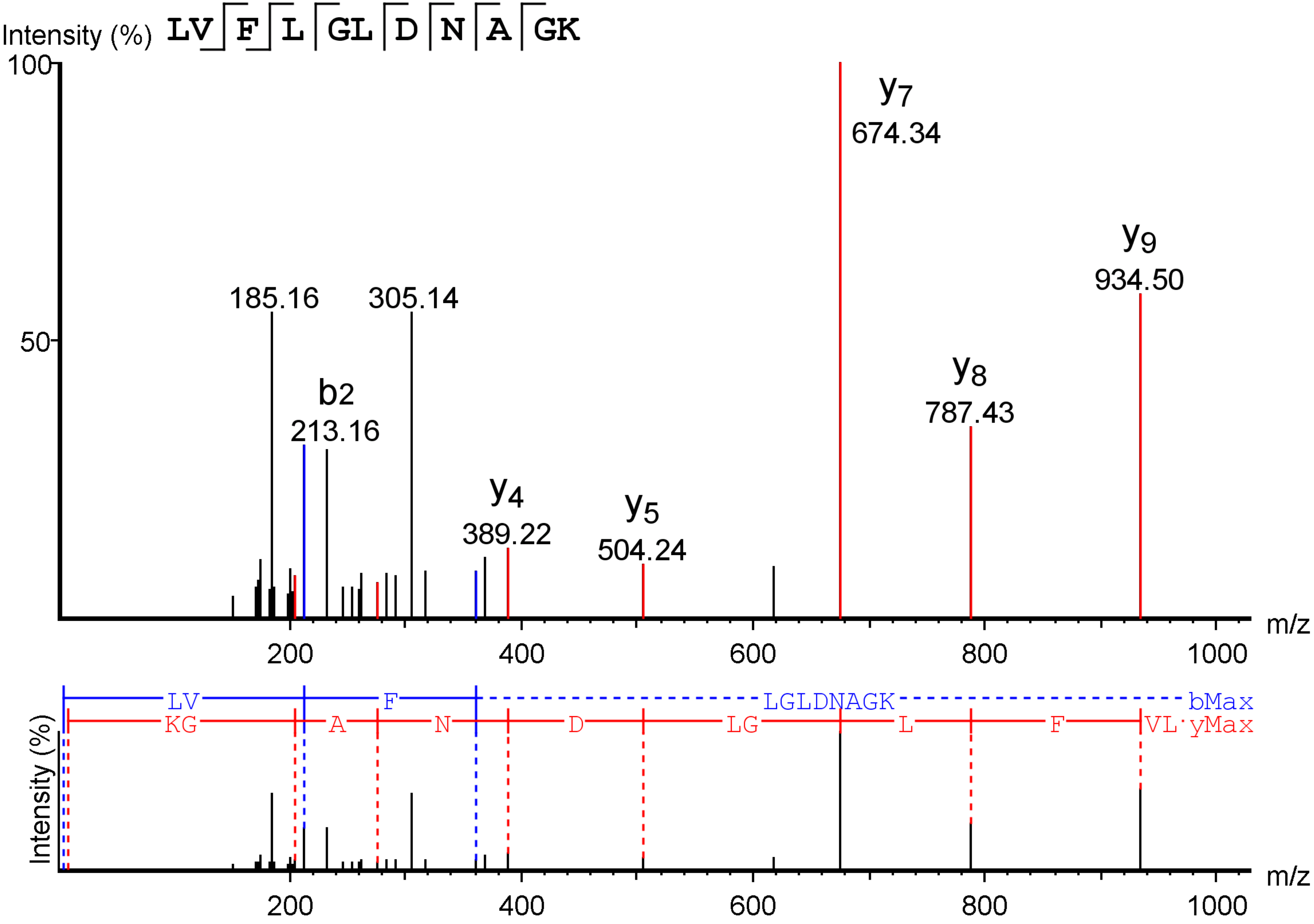

sp|P36578|RL4_HUMAN
R.IEEVPELPLVVEDK.V

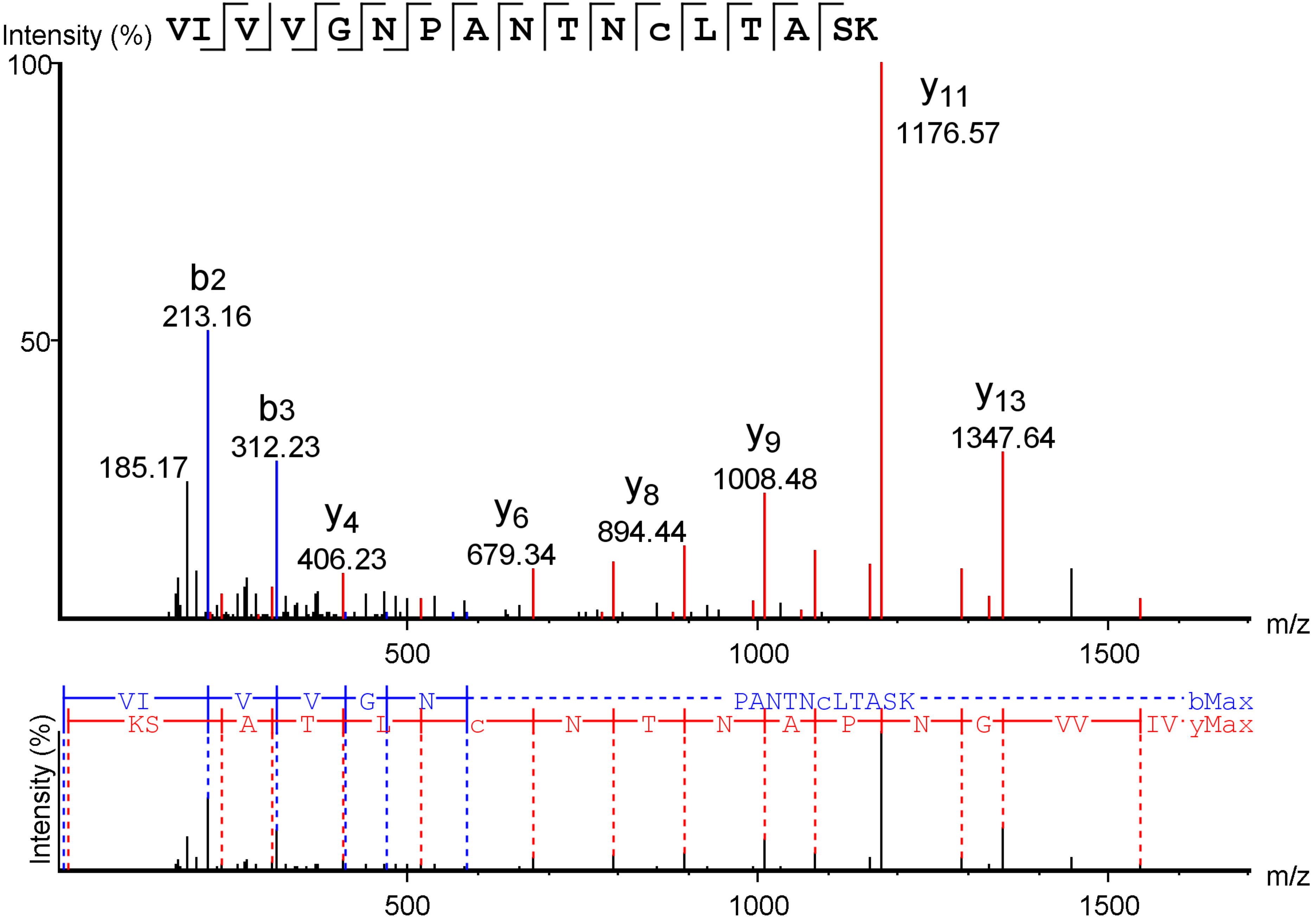

sp|O43237|DC1L2_HUMAN
K.TYGFHFTTPALVVEK.D

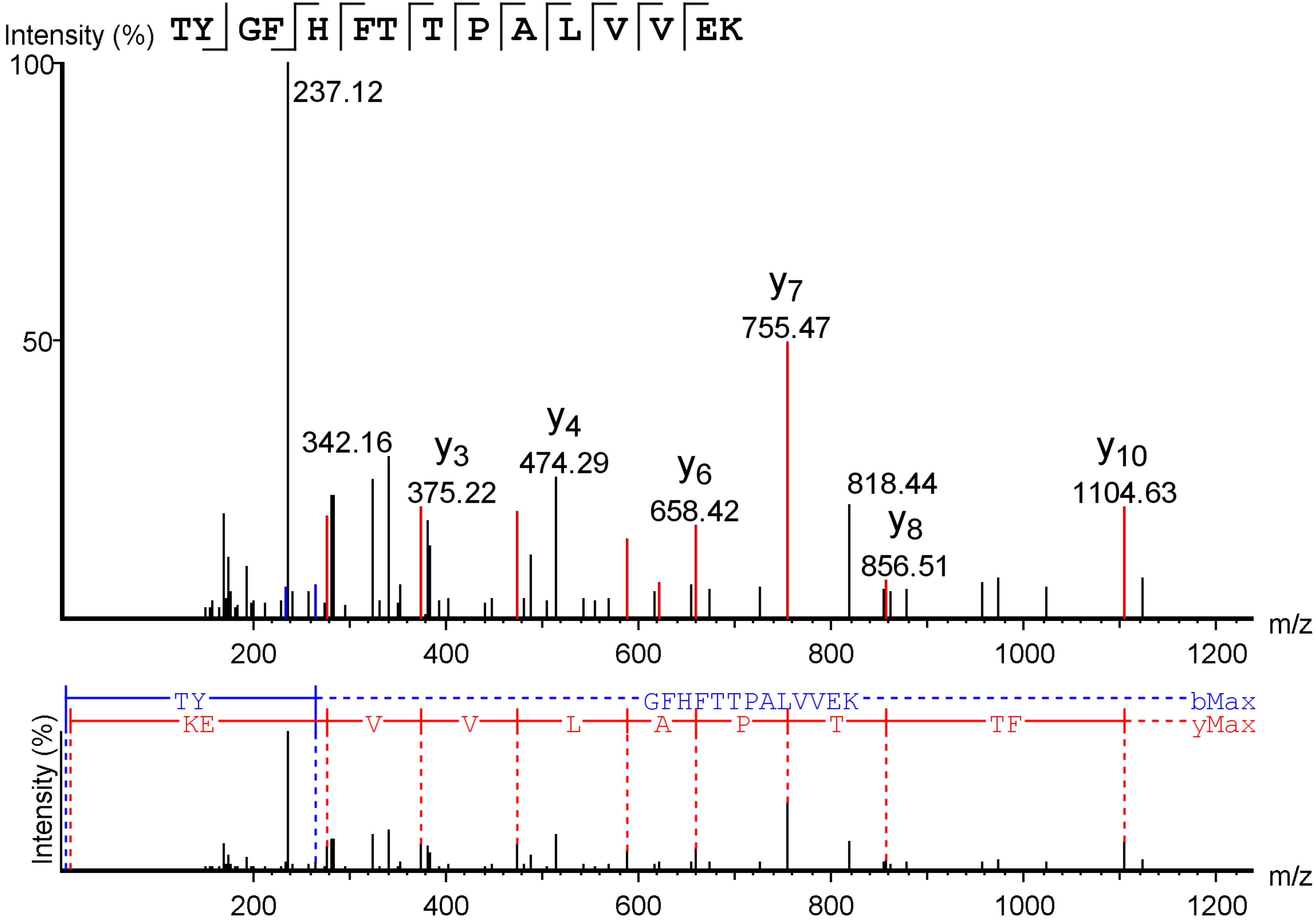

sp|P15586|GNS_HUMAN
K.IQEPNTFPAILR.S

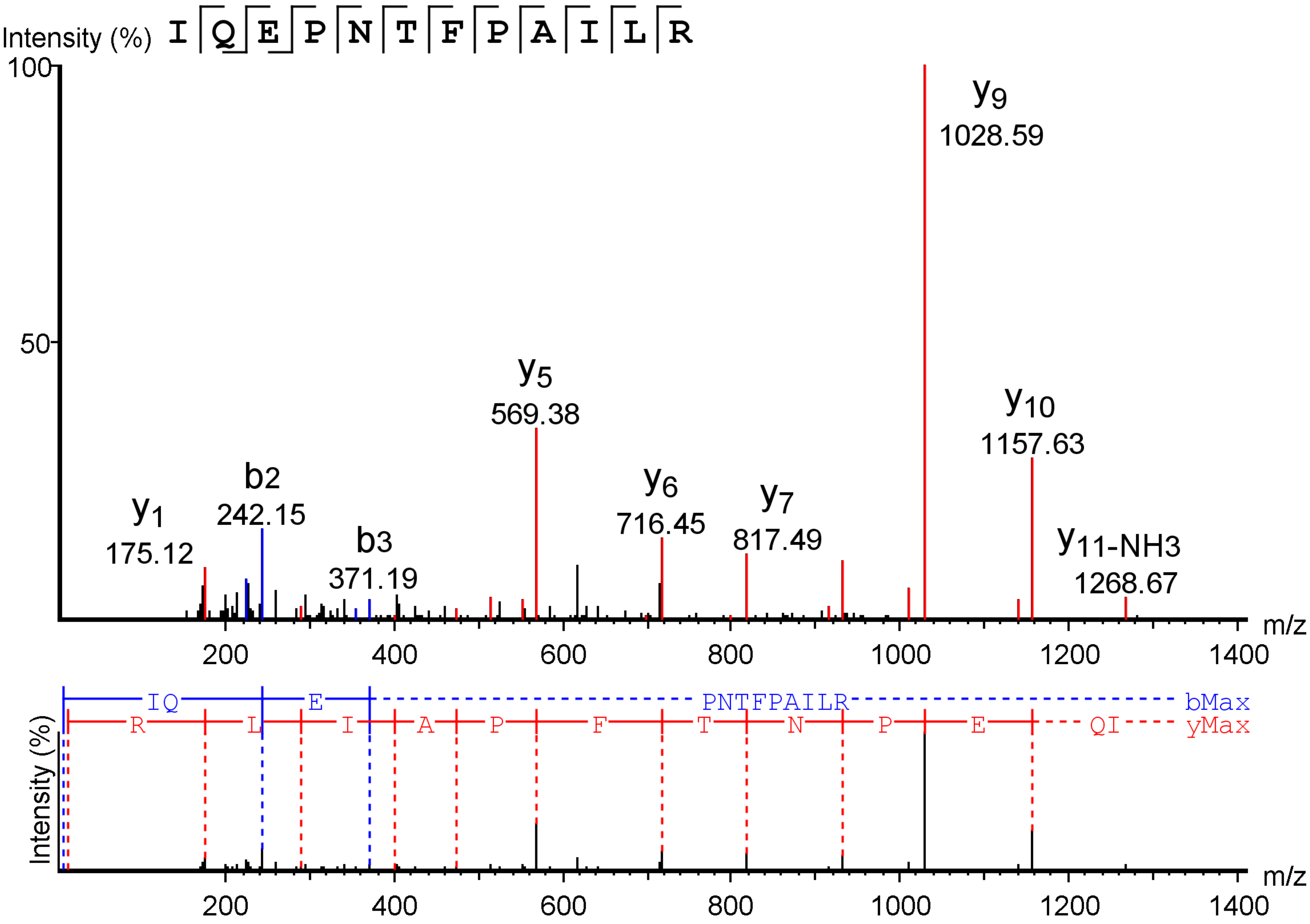

sp|P25705|ATPA_HUMAN
R.VVDALGNAIDGK.G

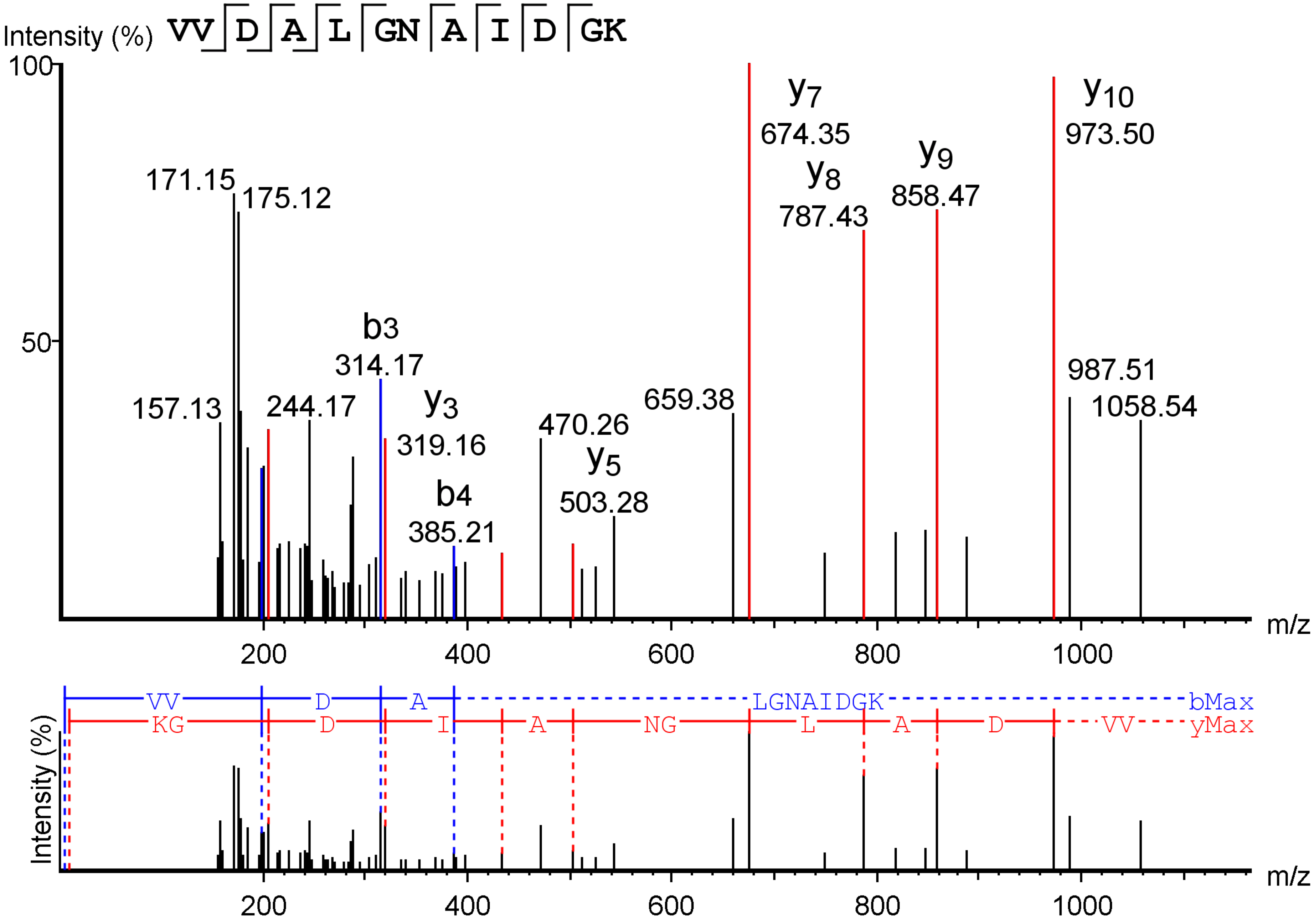

sp|P16298|PP2BB_HUMAN
K.LFEVGGSPANTR.Y

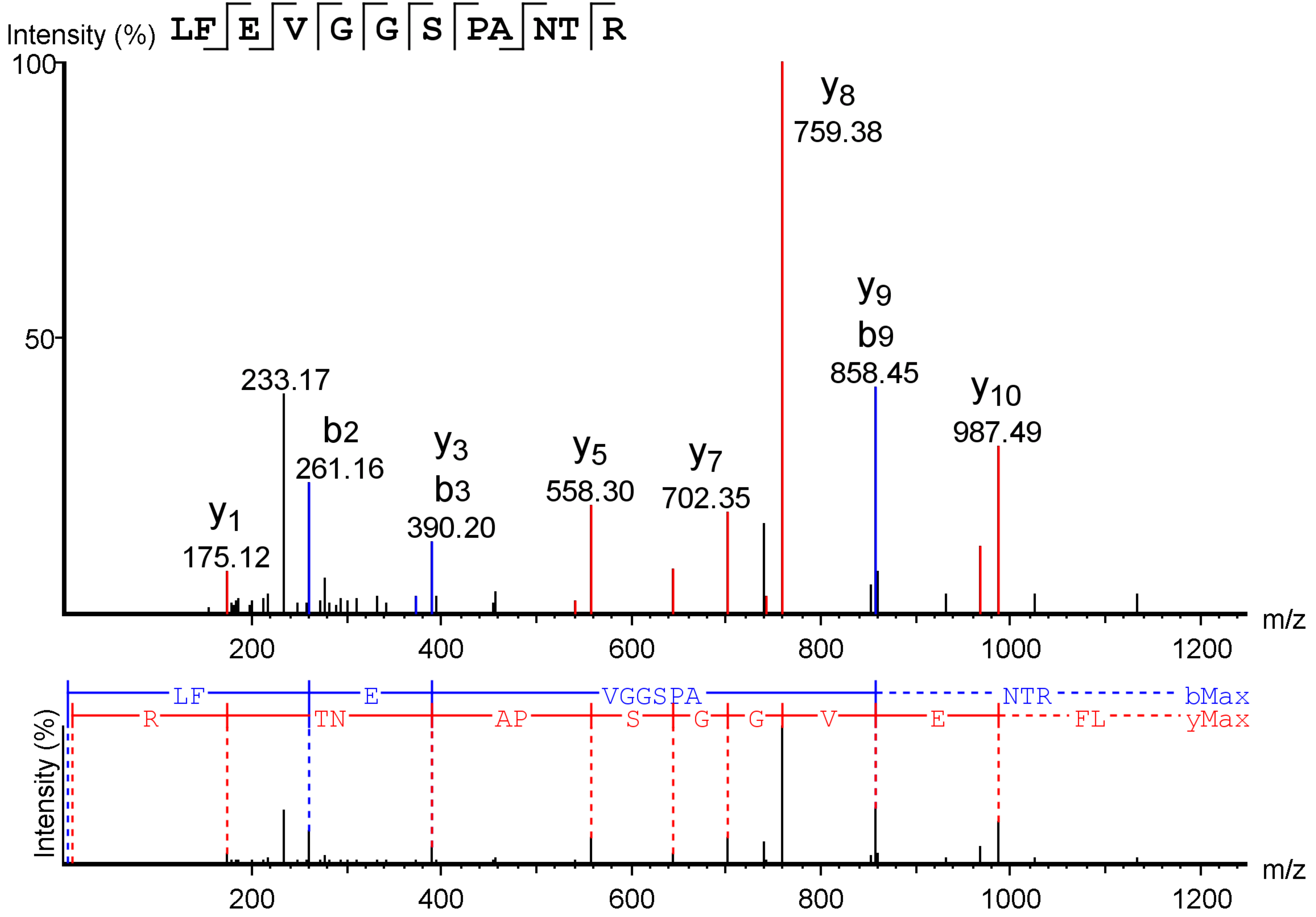

sp|P01782|HV309_HUMAN
R.AEDTALYYC(+57.02)AK.D

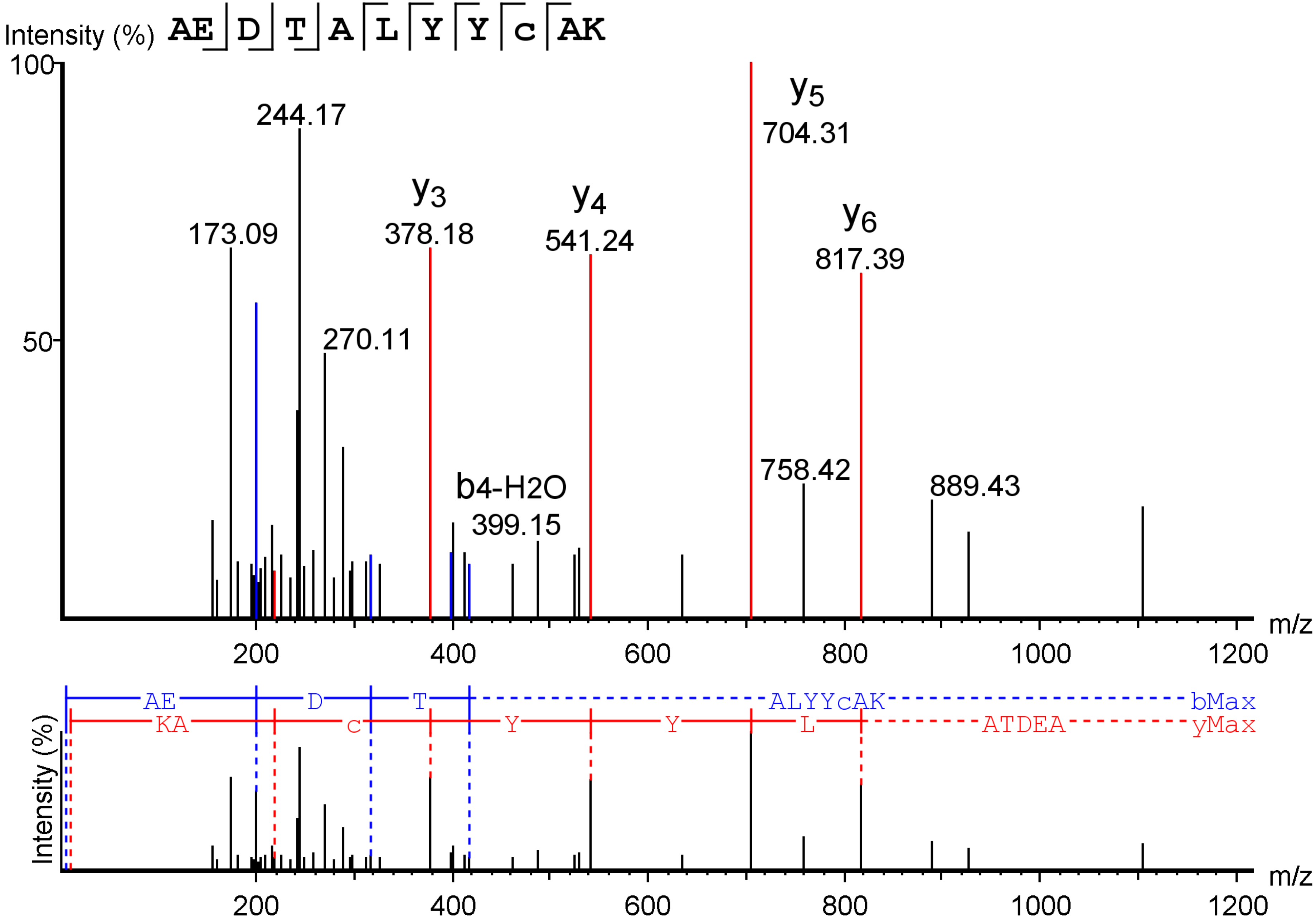

sp|Q14697|GANAB_HUMAN
R.YRVPDVLVADPPIAR.L

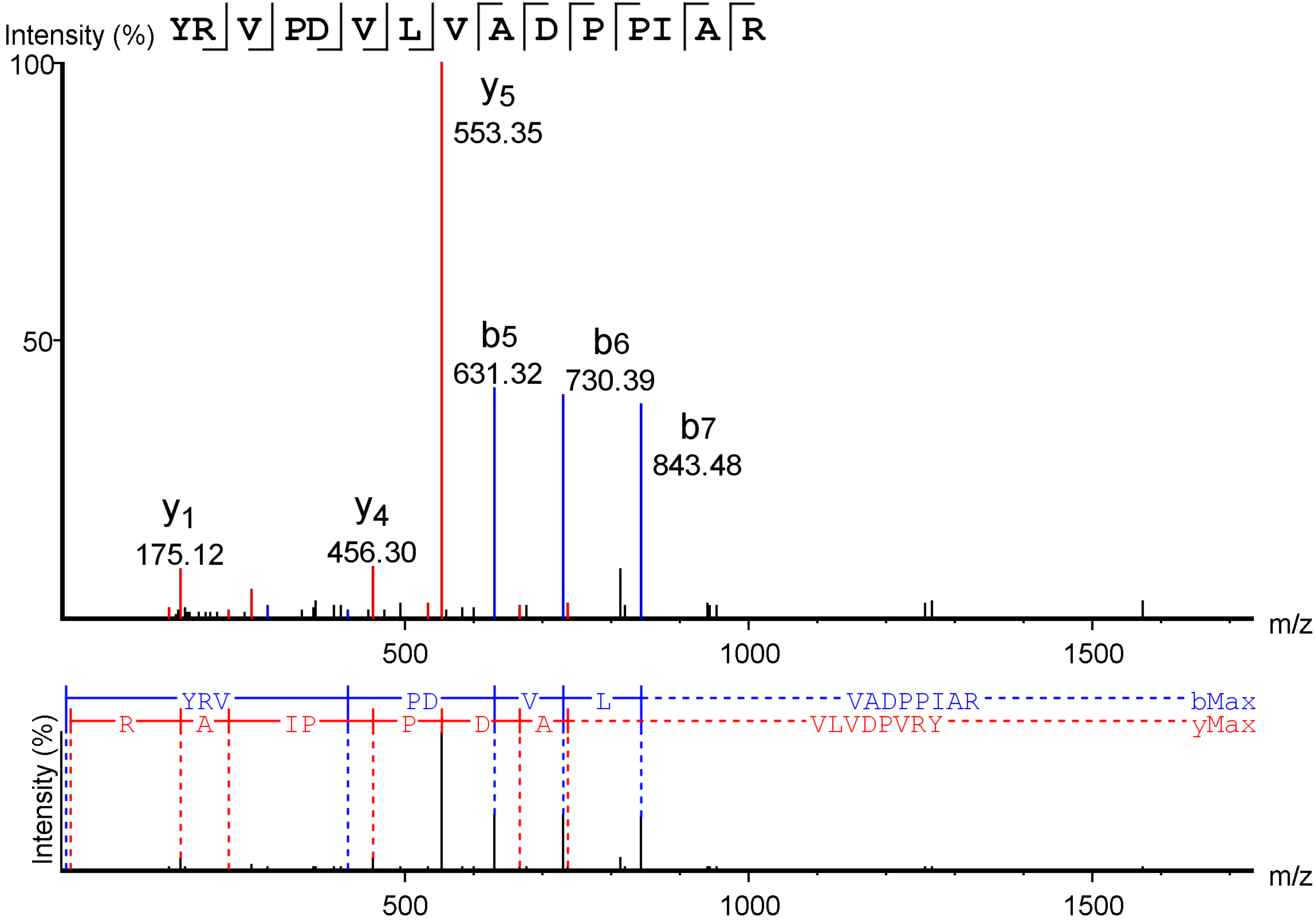

sp|P04424|ARLY_HUMAN
R.SRNDQVVTDLR.L

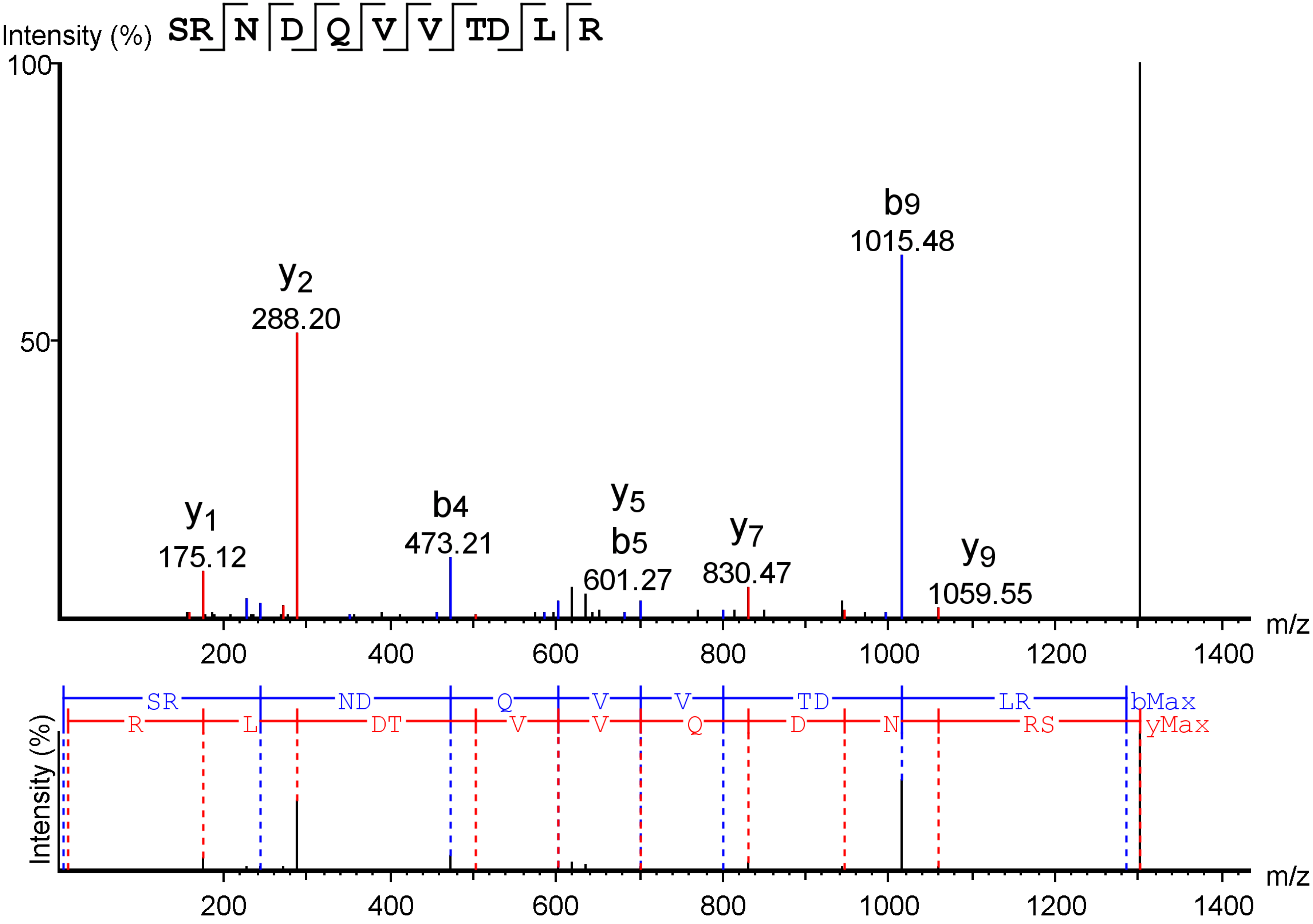

sp|Q9UM00|TMCO1_HUMAN
K.KETITESAGR.Q

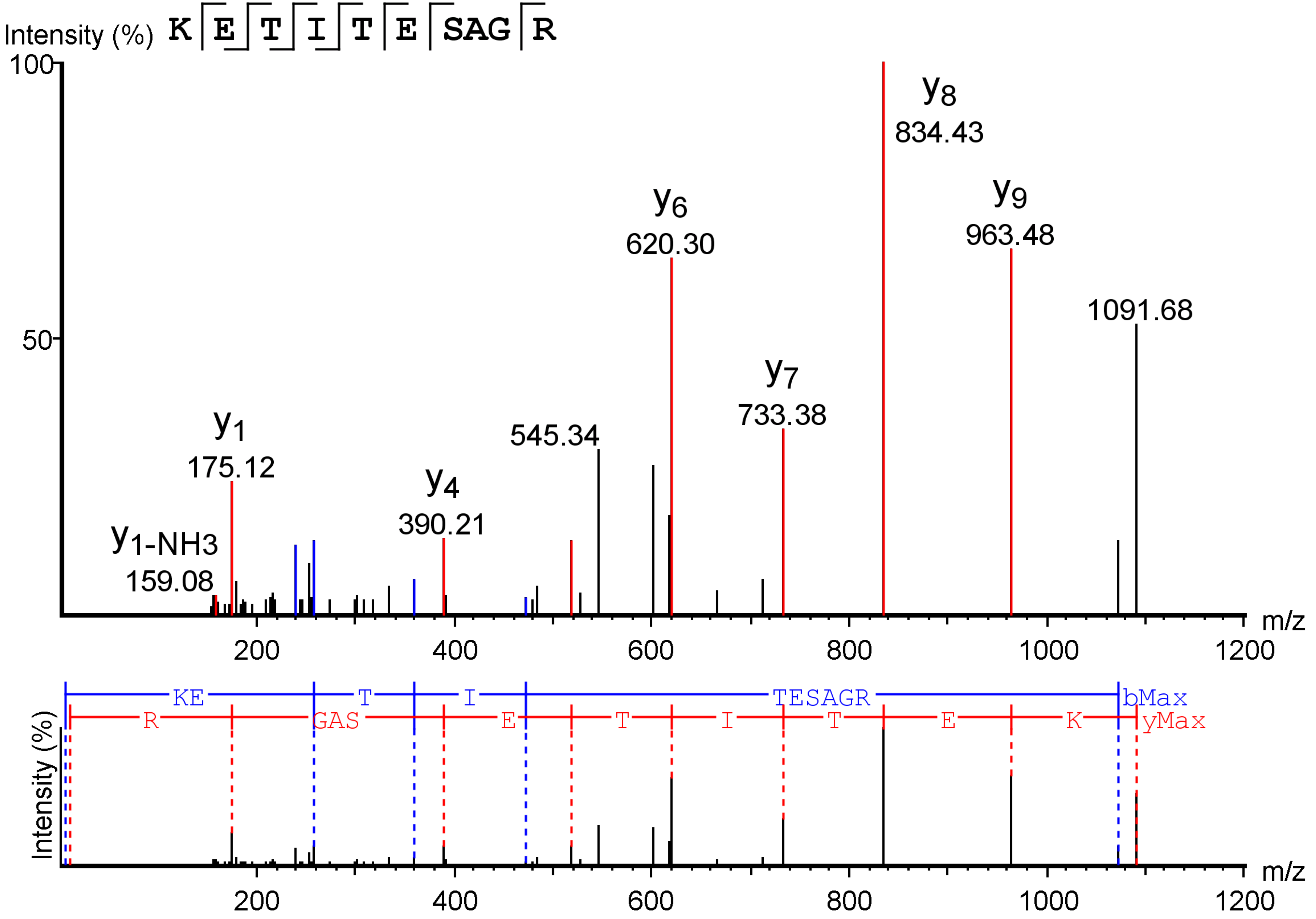

sp|Q92616|GCN1_HUMAN
R.TLGDLVR.K

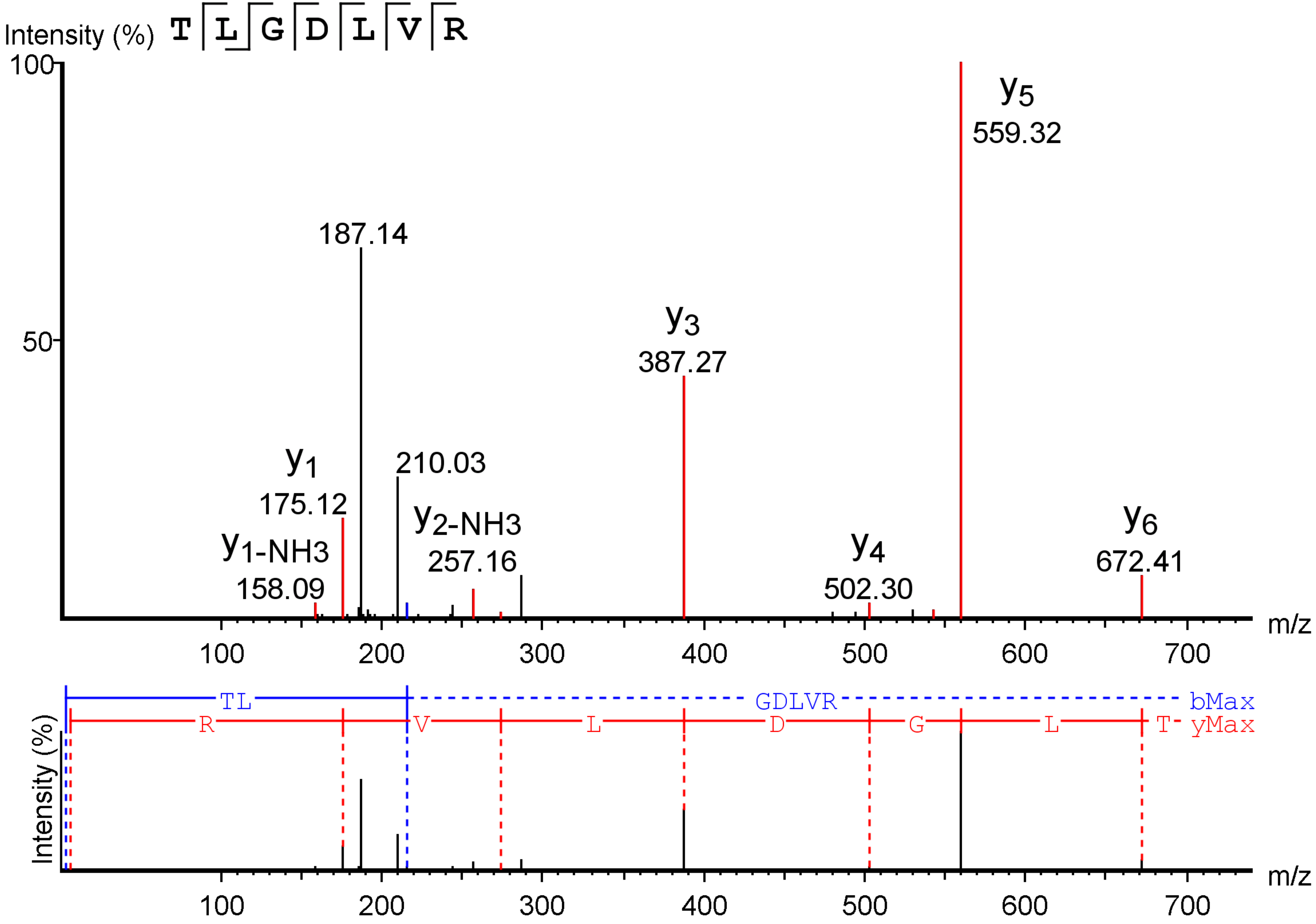

sp|O94985|CSTN1_HUMAN
K.VIDC(+57.02)LYTC(+57.02)K.E

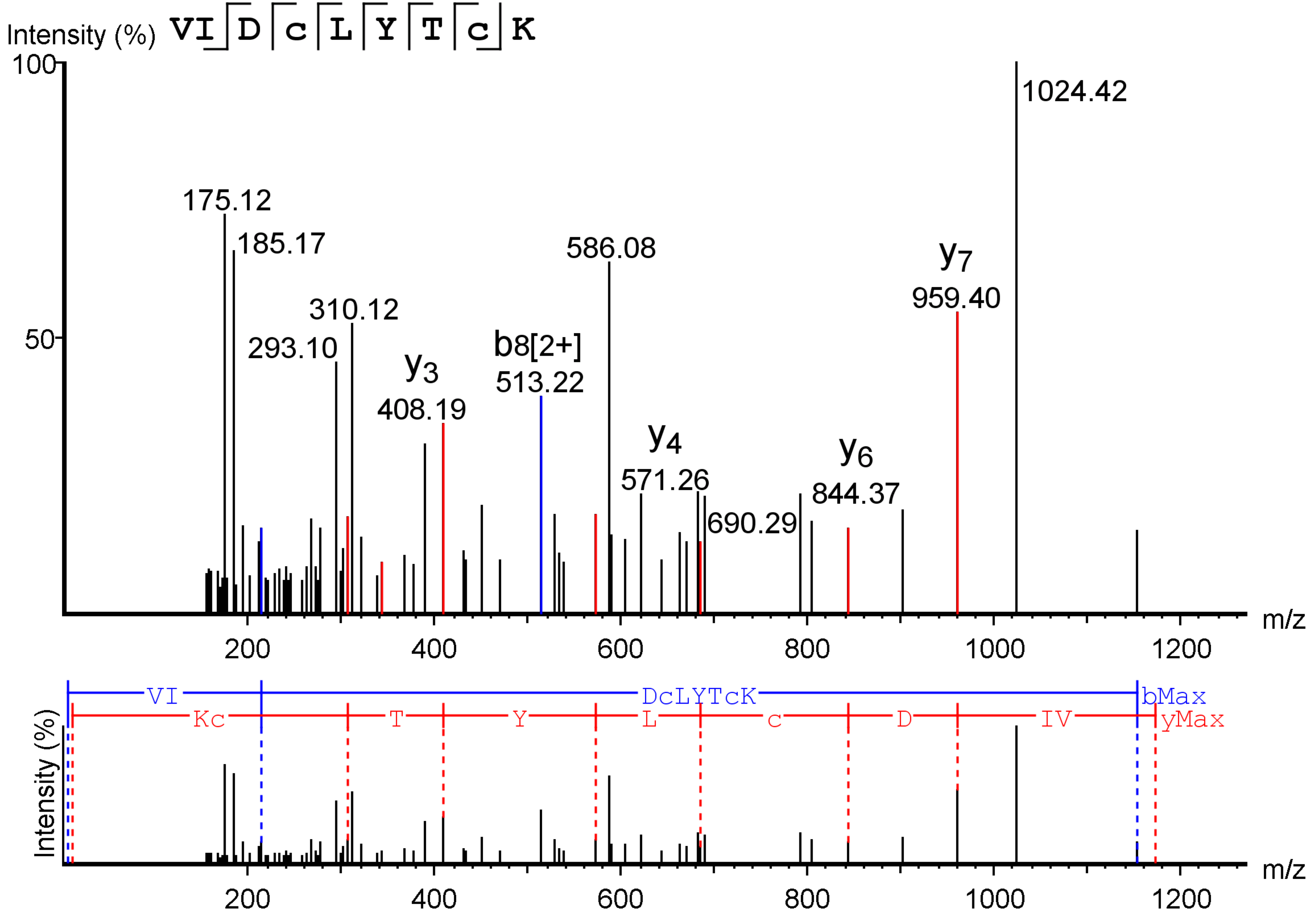

sp|P62304|RUXE_HUMAN
K.VM(+15.99)VQPINLIFR.Y

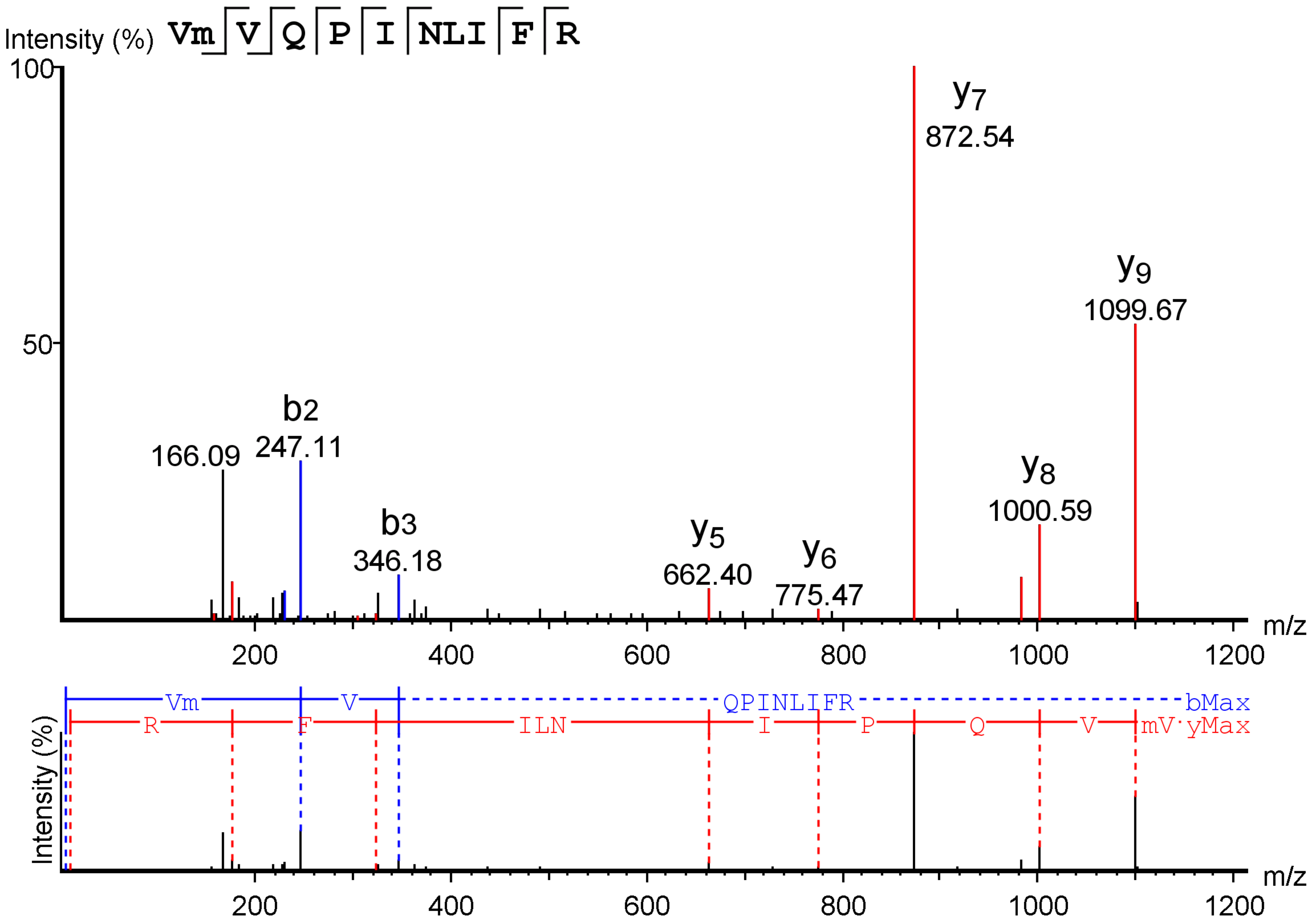

sp|P10155|RO60_HUMAN
K.LGLENAEALIR.L

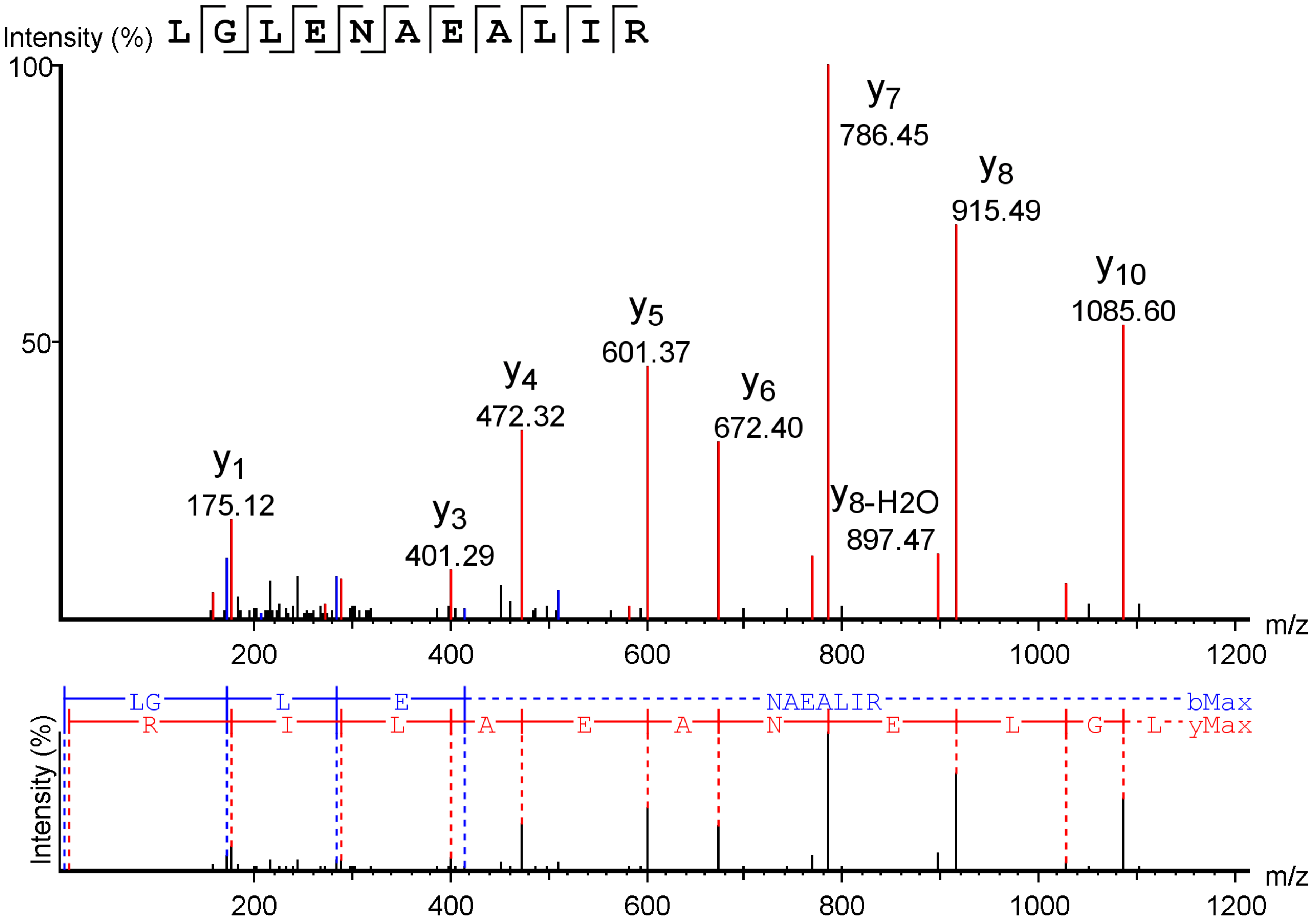

sp|P62314|SMD1_HUMAN
K.NREPVQLETLSIR.G

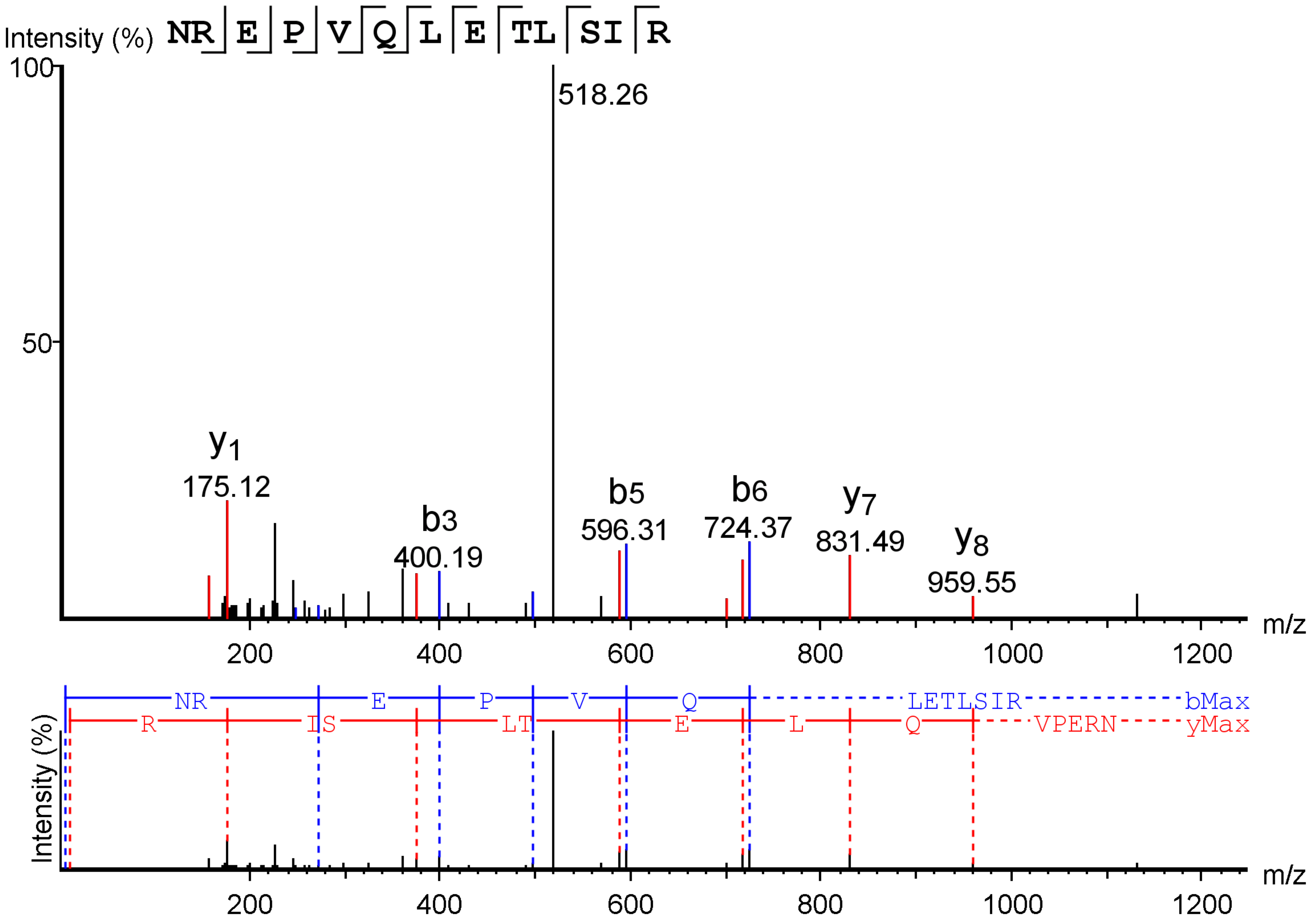

sp|O75347|TBCA_HUMAN
R.RLEAAYLDLQR.I

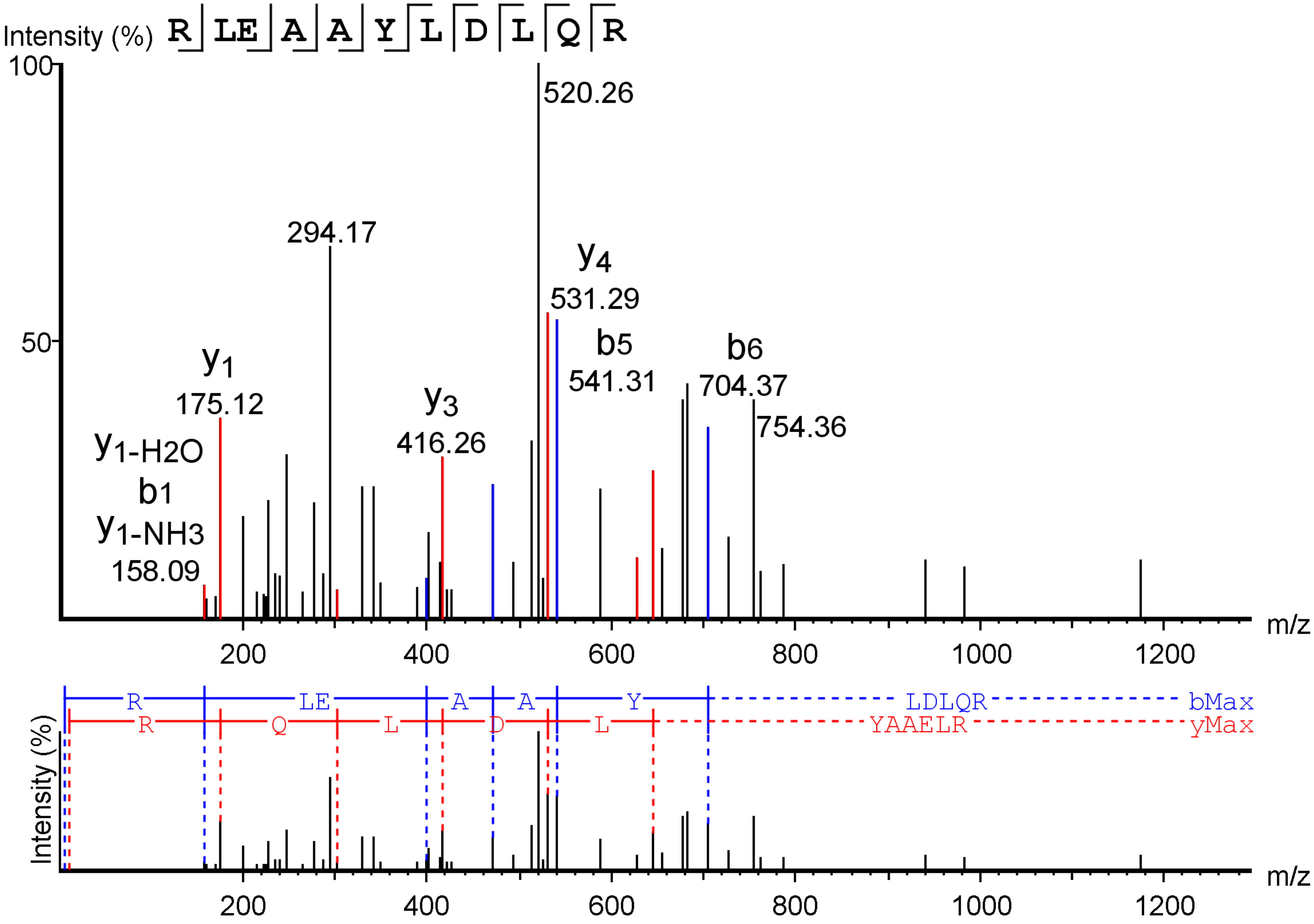

E9PJD9|E9PJD9_HUMAN
K.TGAAPIIDVVR.S

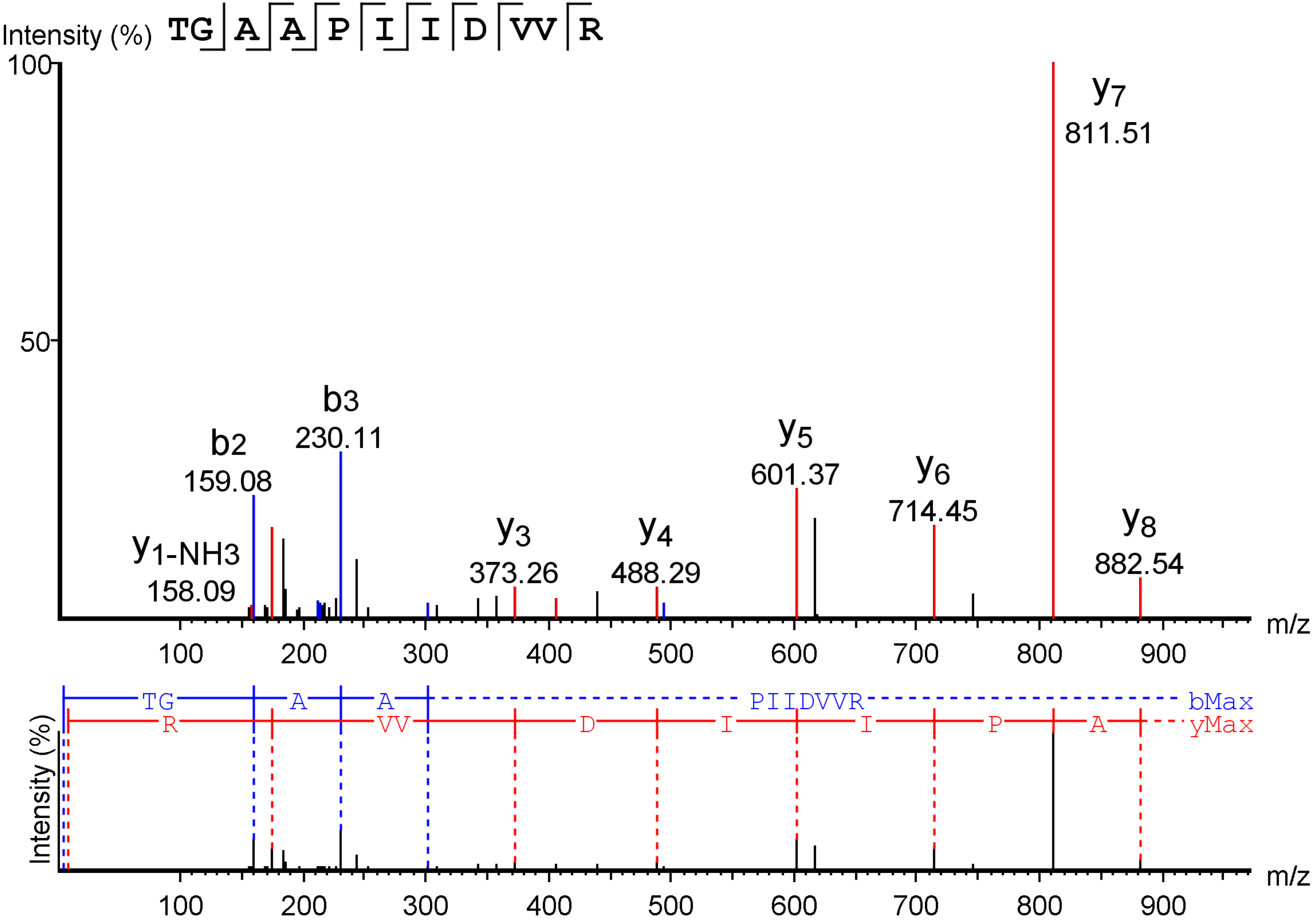

sp|Q8TD26|CHD6_HUMAN
R.EQADFYR.T

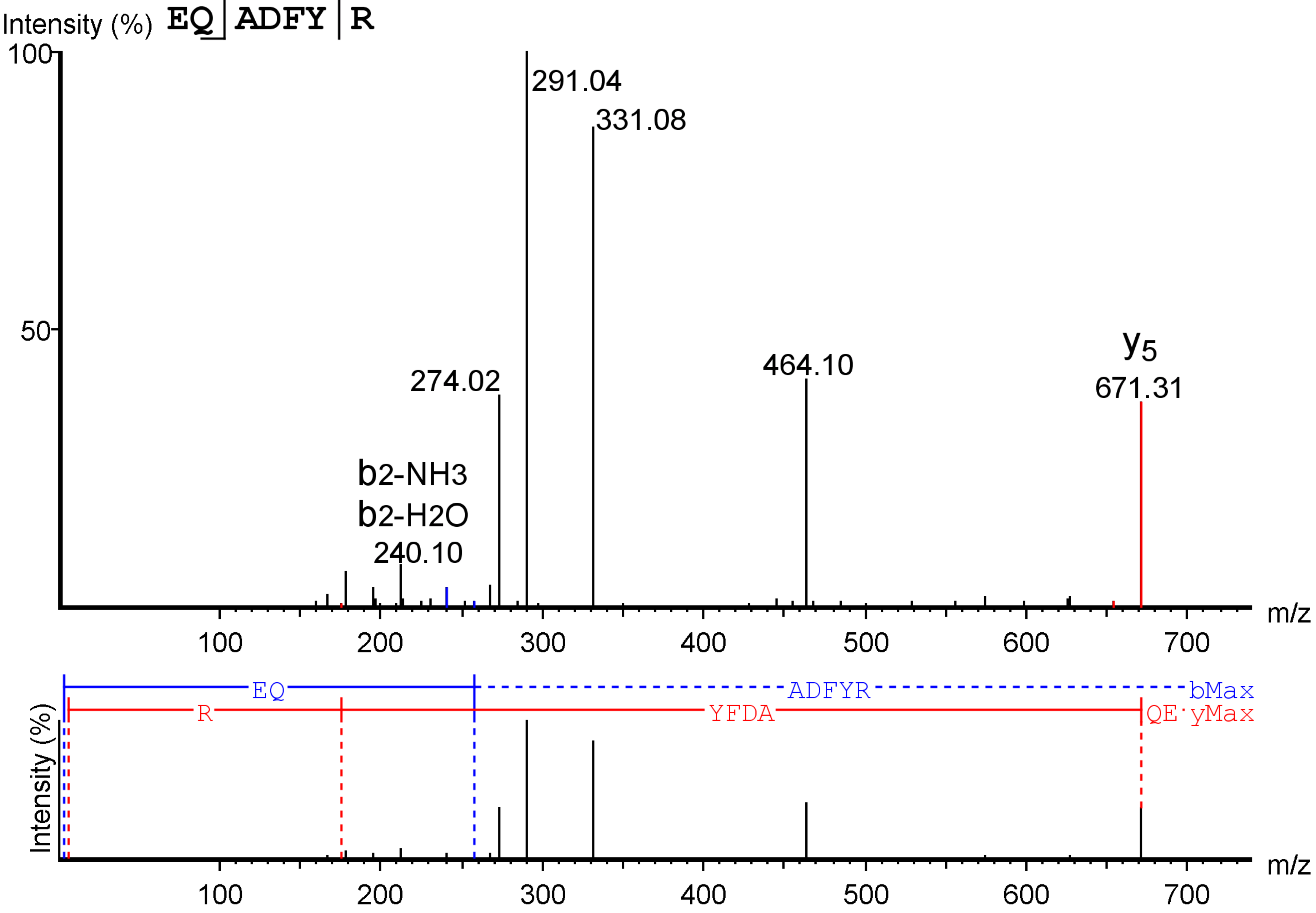

sp|P10114|RAP2A_HUMAN
K.VPVILVGNK.V

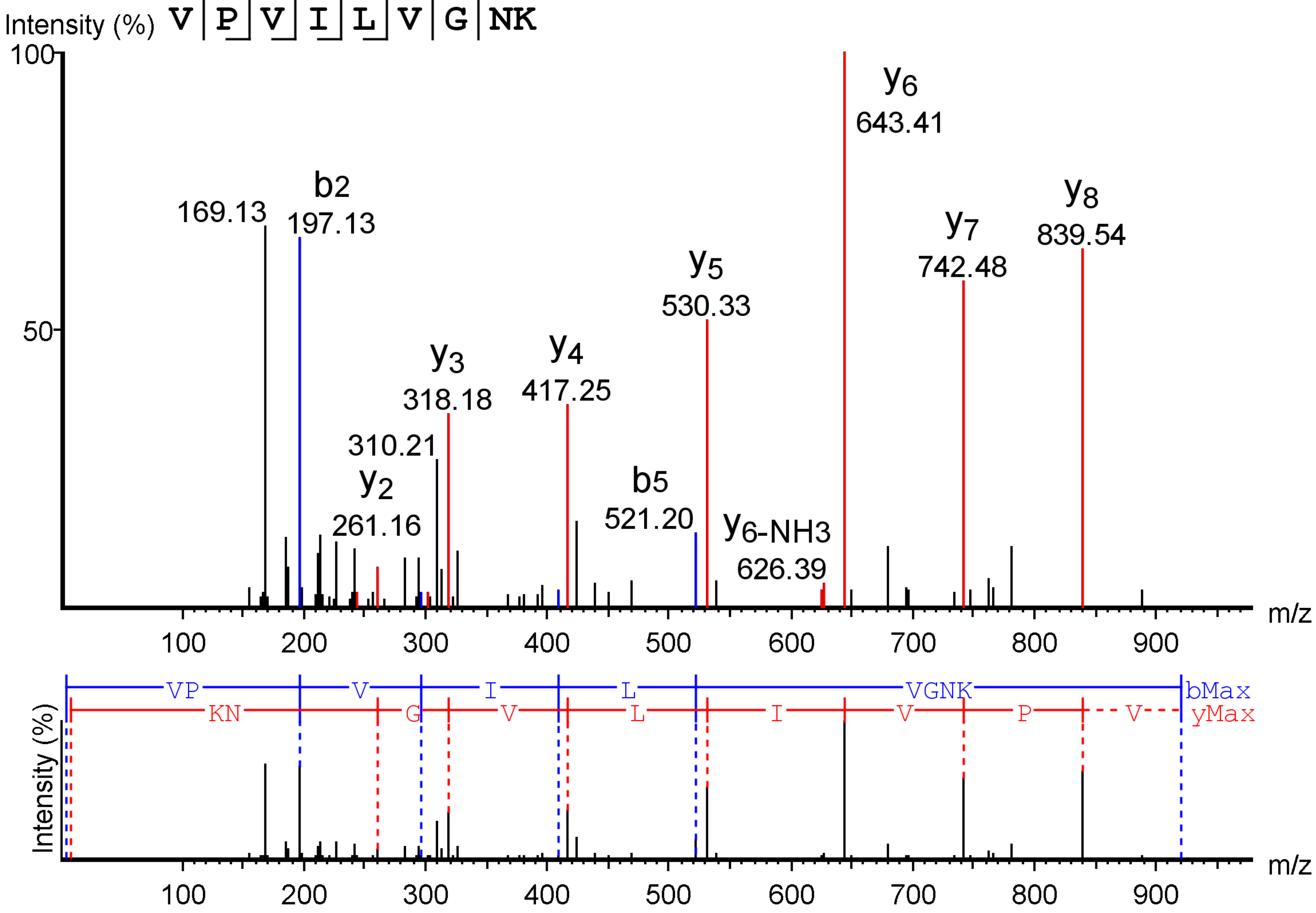

sp|Q13822|ENPP2_HUMAN
K.AGTFFWSVVIPHER.R

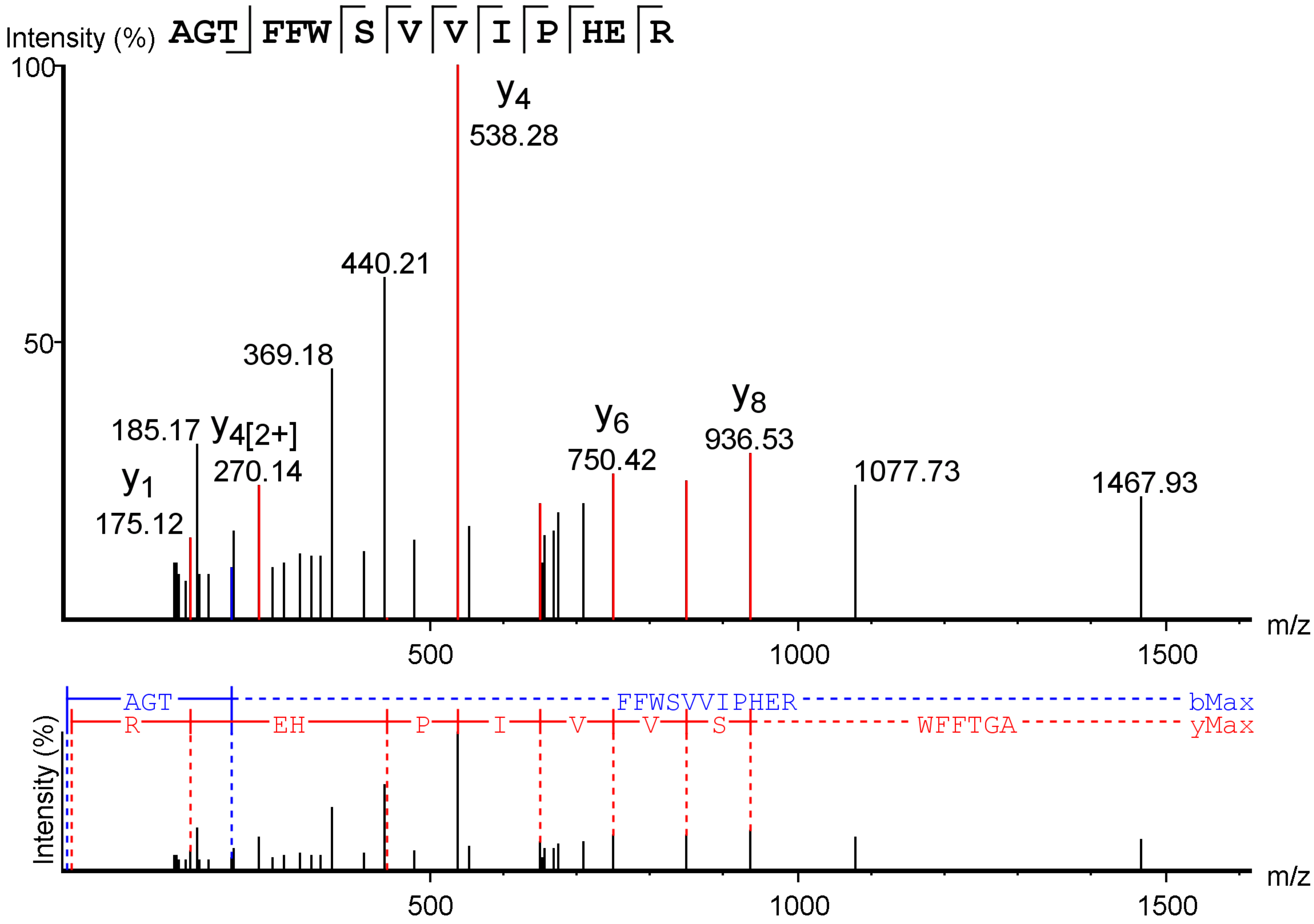

sp|Q9P2W7|B3GA1_HUMAN
R.ELVTLNDLEPK.A

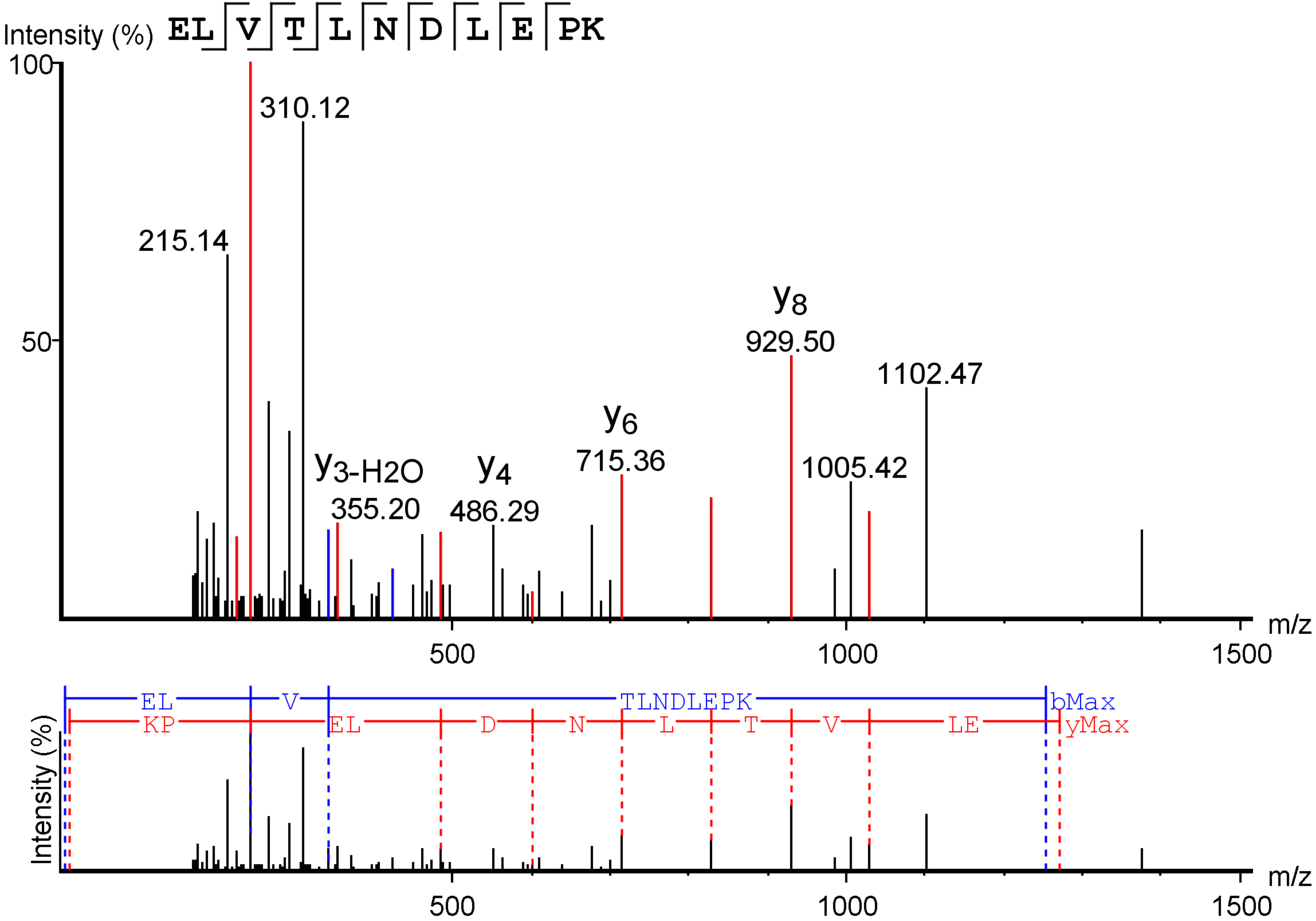

sp|Q9UHD8|SEPT9_HUMAN

K.STLINTLFK.S

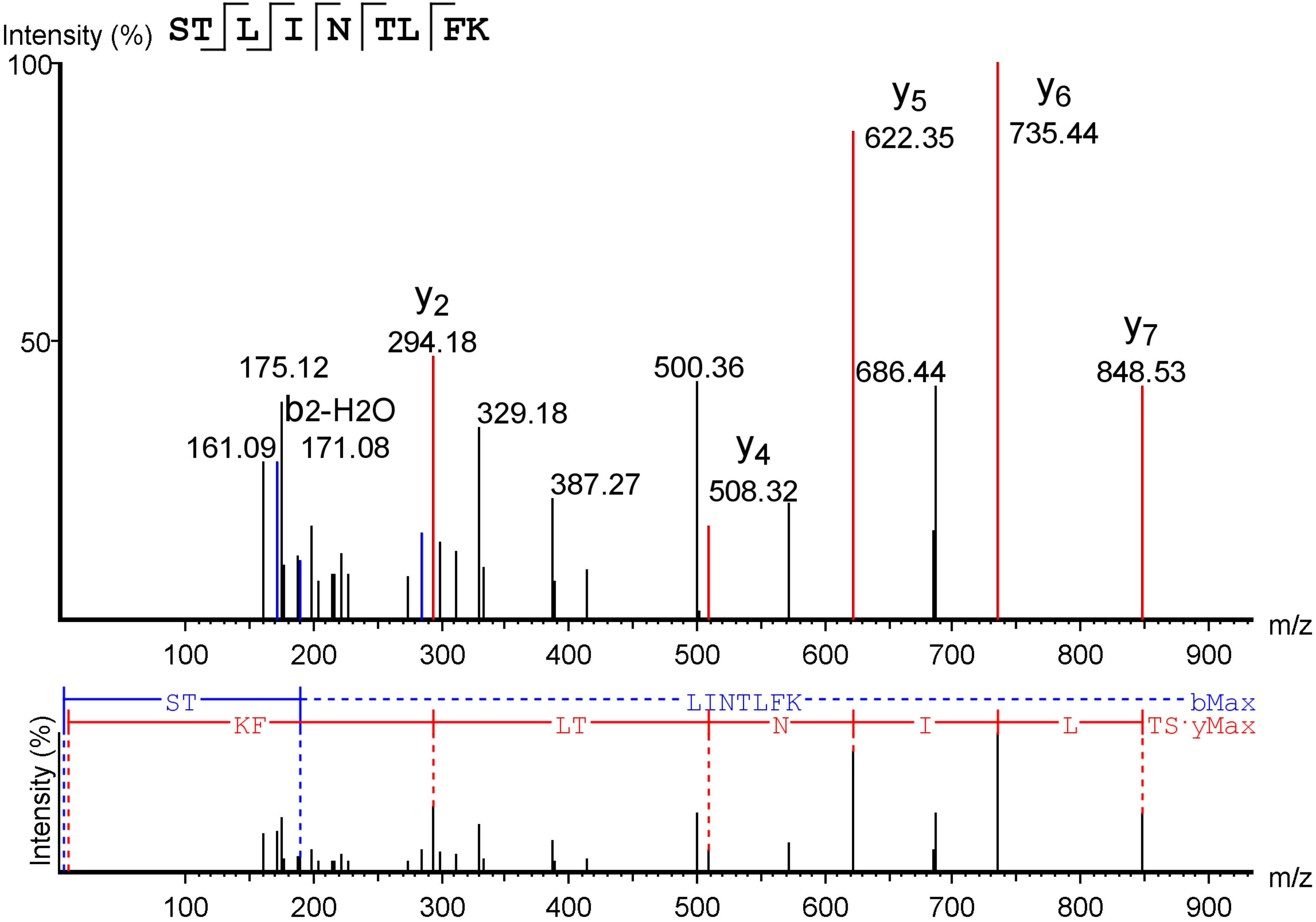

sp|Q9UHB9|SRP68_HUMAN
R.FETFC(+57.02)LDPSLVTK.Q

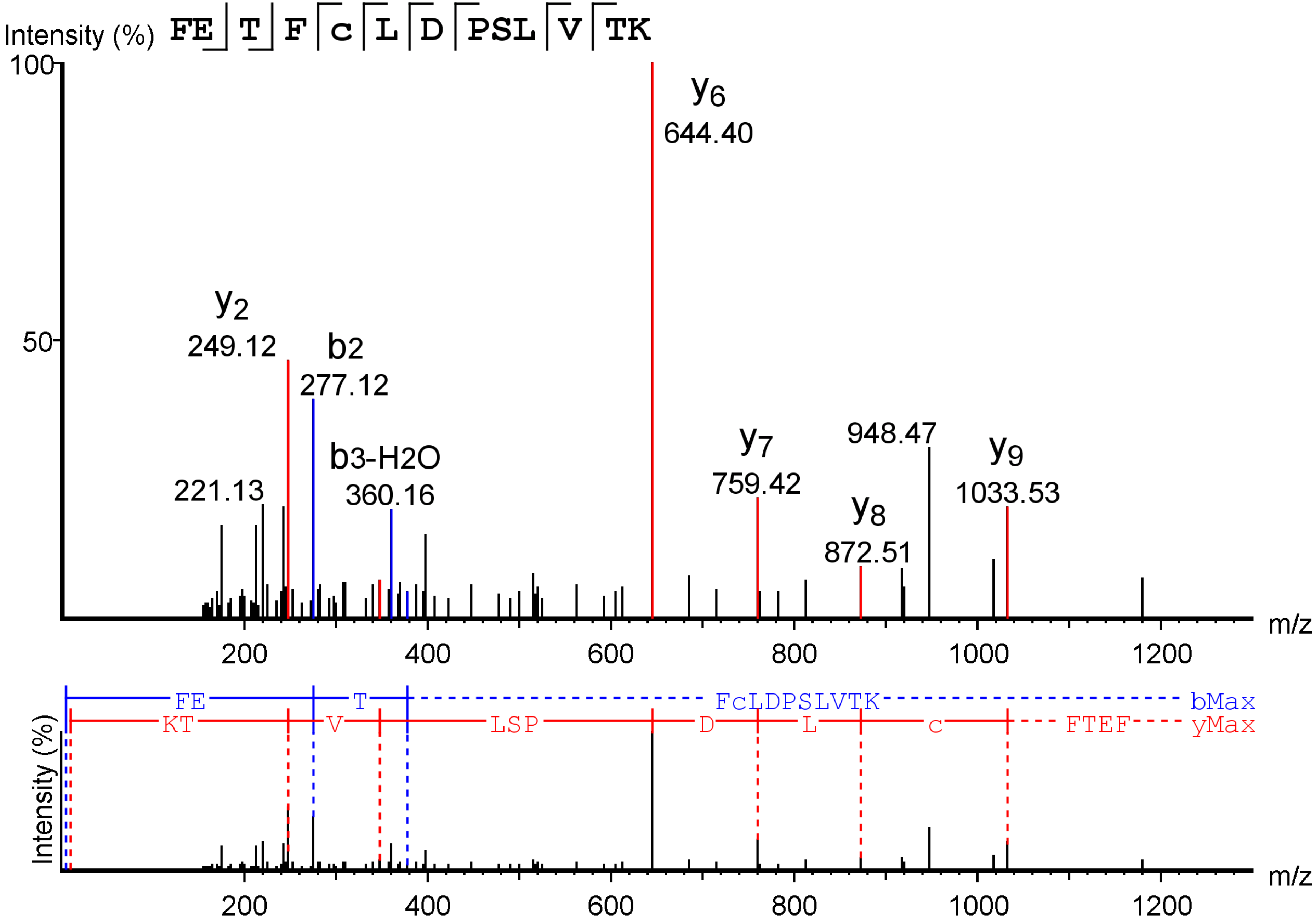

sp|P31949|S10AB_HUMAN
K.ISSPTETER.C

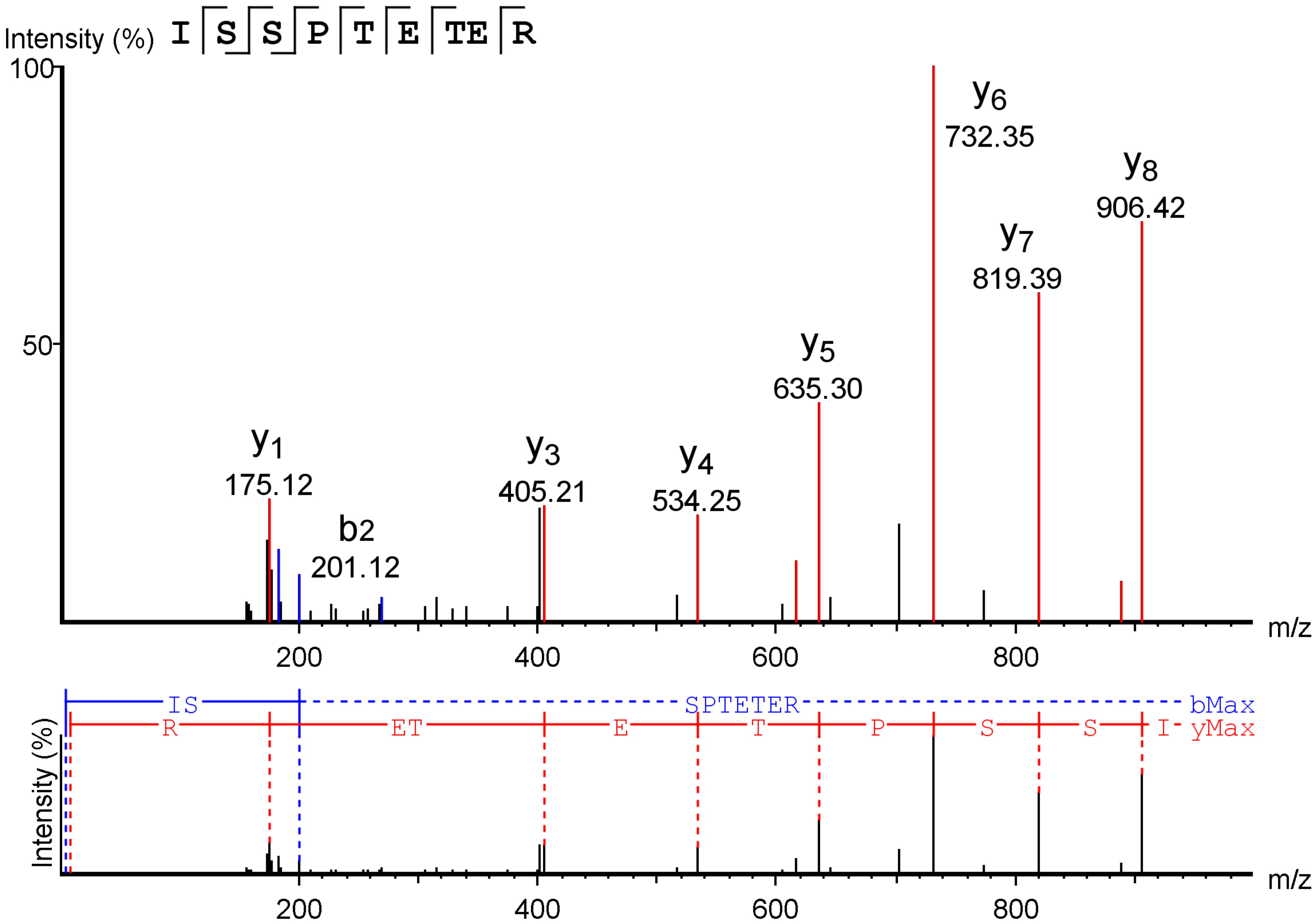

sp|O14910|LIN7A_HUMAN
K.ATVAAFAASEGHSHPR.V

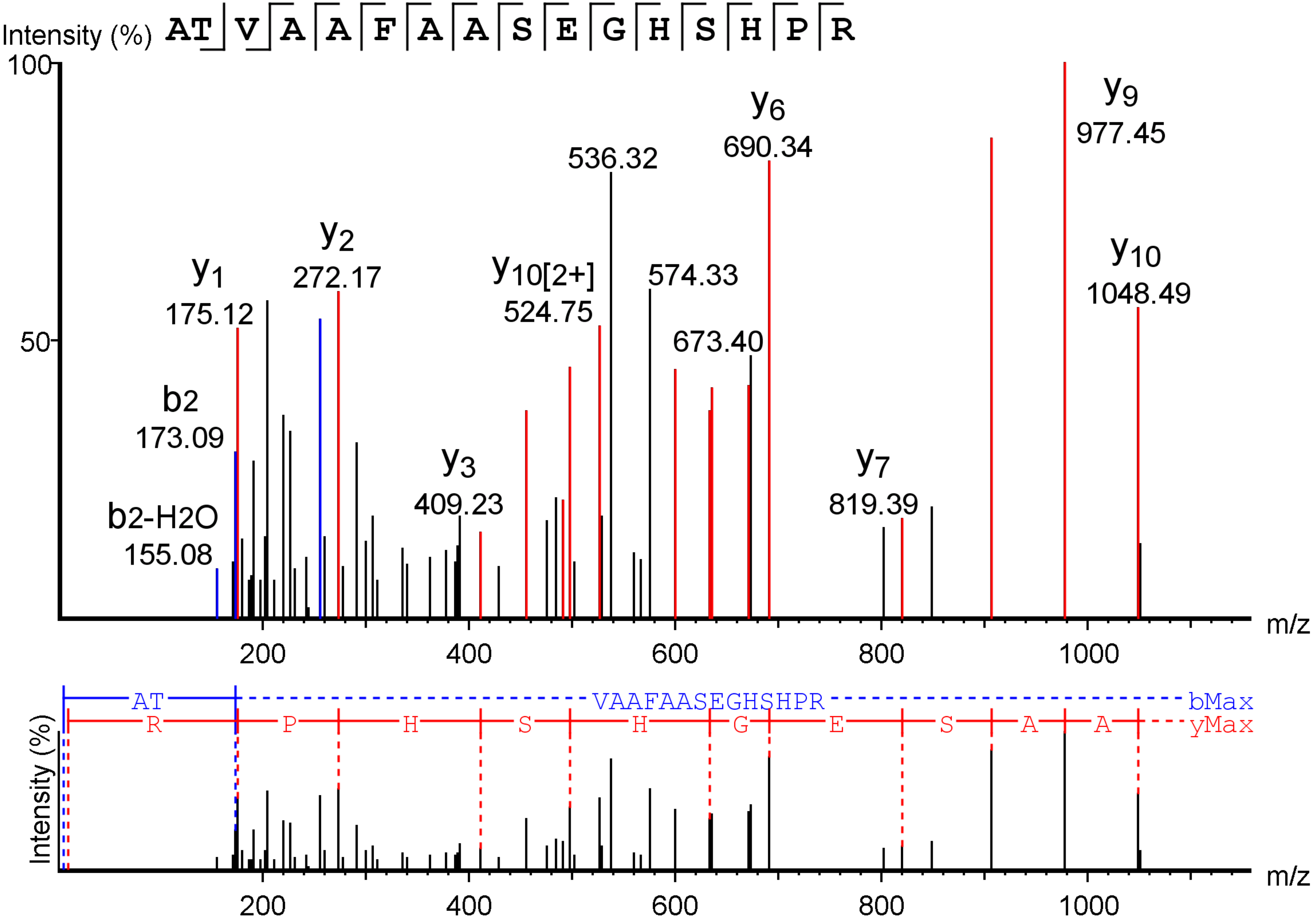

sp|Q99829|CPNE1_HUMAN
R.LYGPTNFAPIINHVAR.F

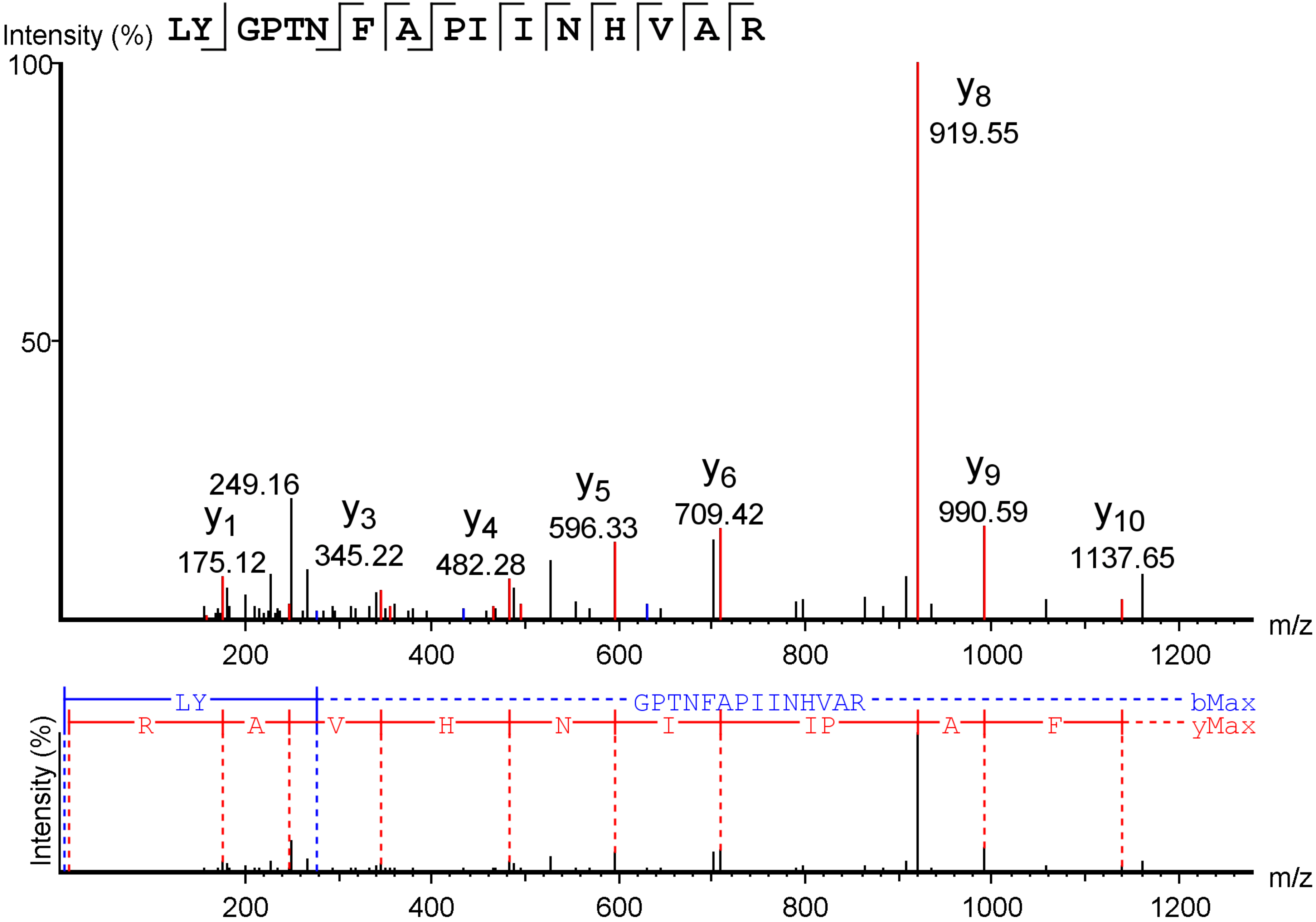

sp|P23471|PTPRZ_HUMAN
K.AIIDGVESVSR.F

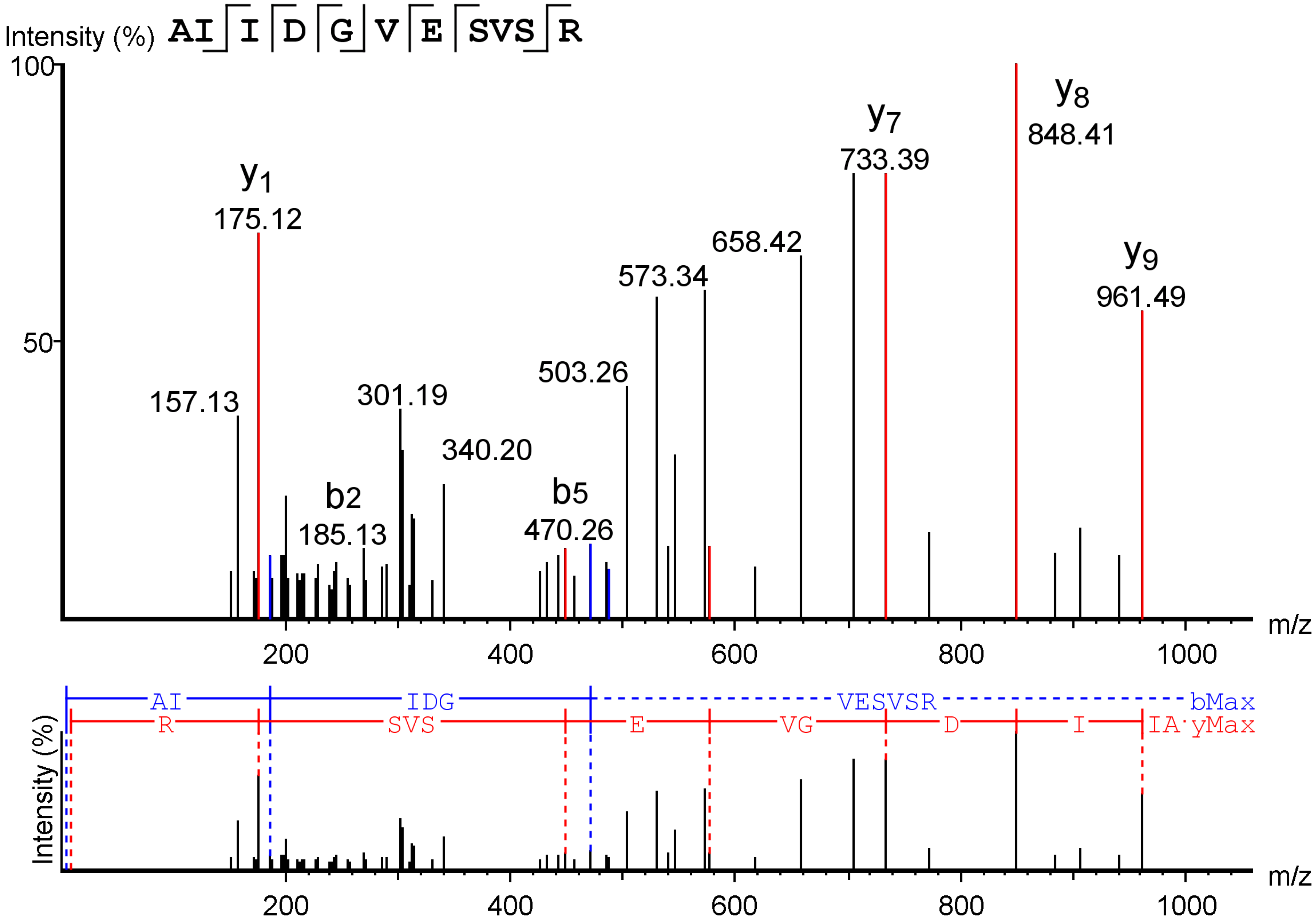

sp|P26022|PTX3_HUMAN
R.ALAAVLEELR.Q

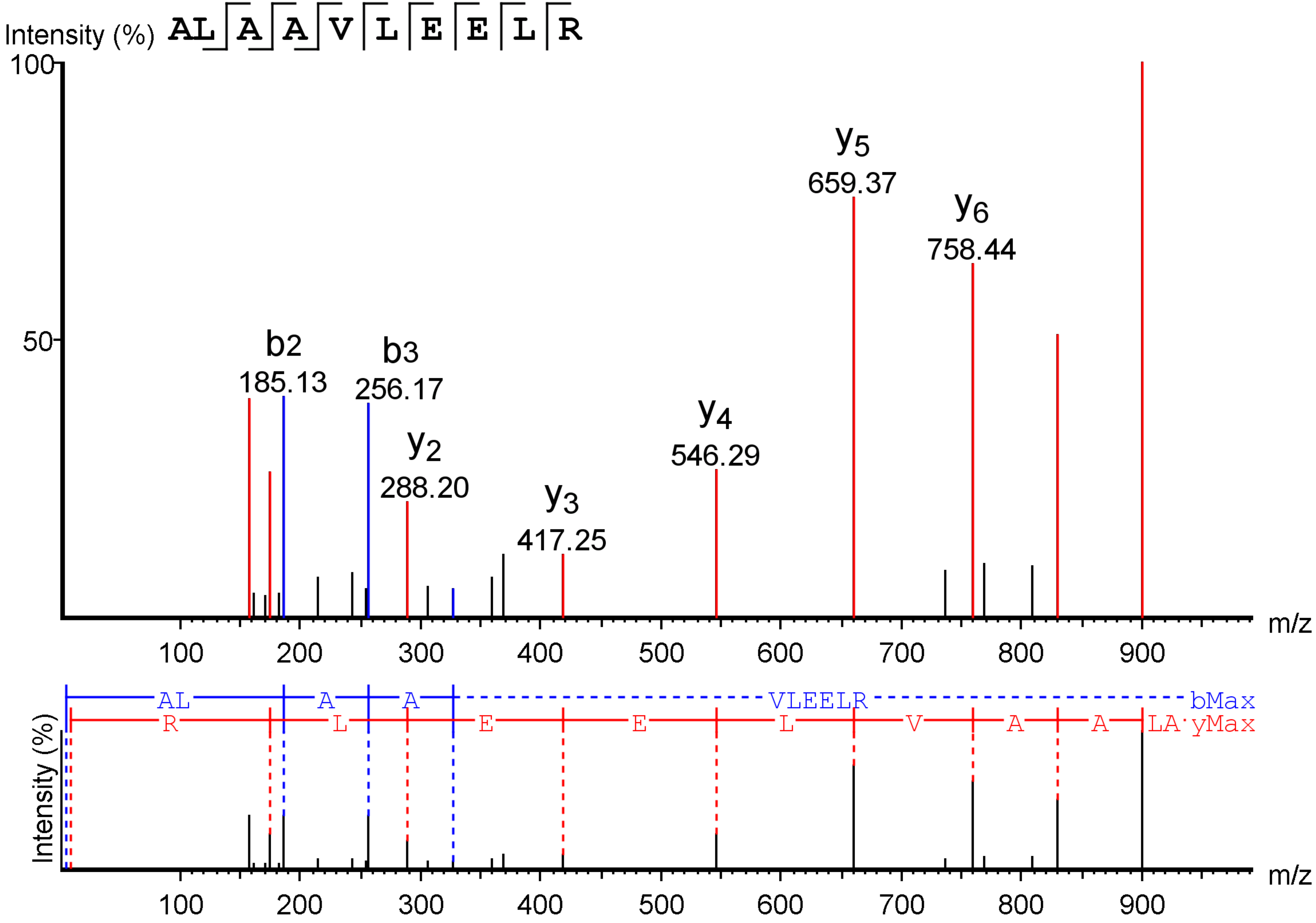

sp|Q92832|NELL1_HUM
AN K.AFLFQDIER.E

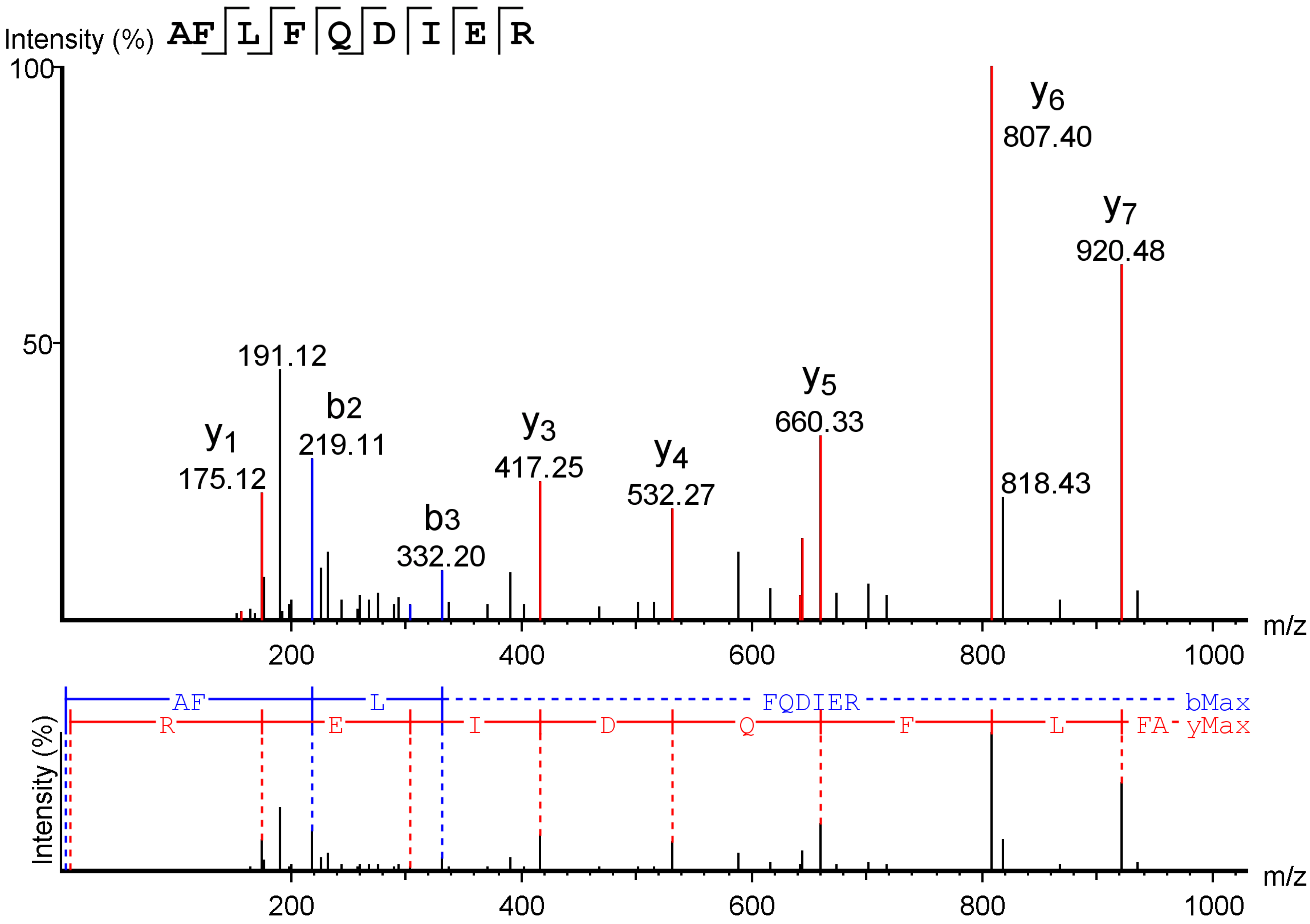

sp|Q7L576|CYFP1_HUMAN
R.LGTPQQIAIAR.E

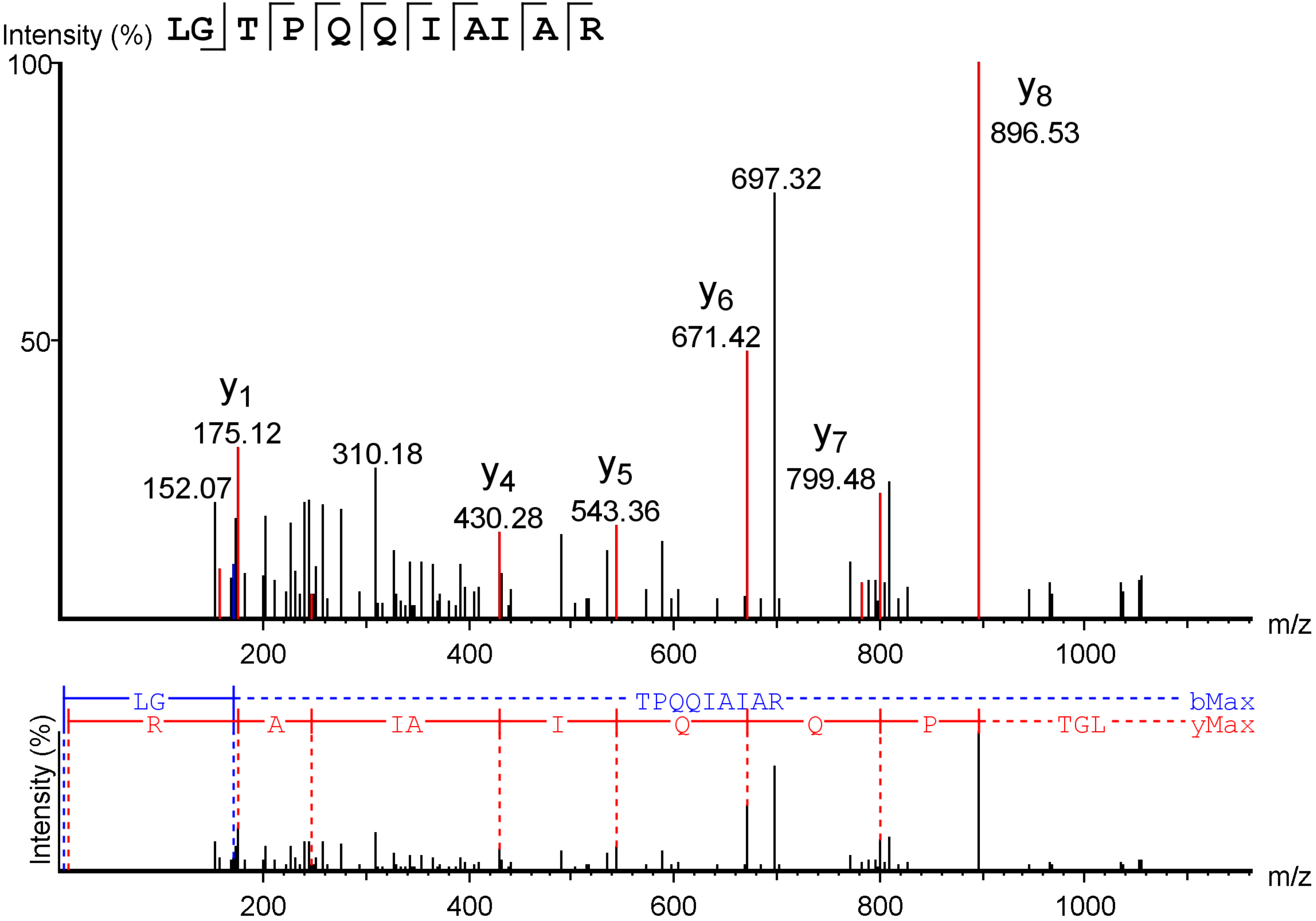

sp|O75781|PALM_HUMAN
R.LLEDSVSR.L

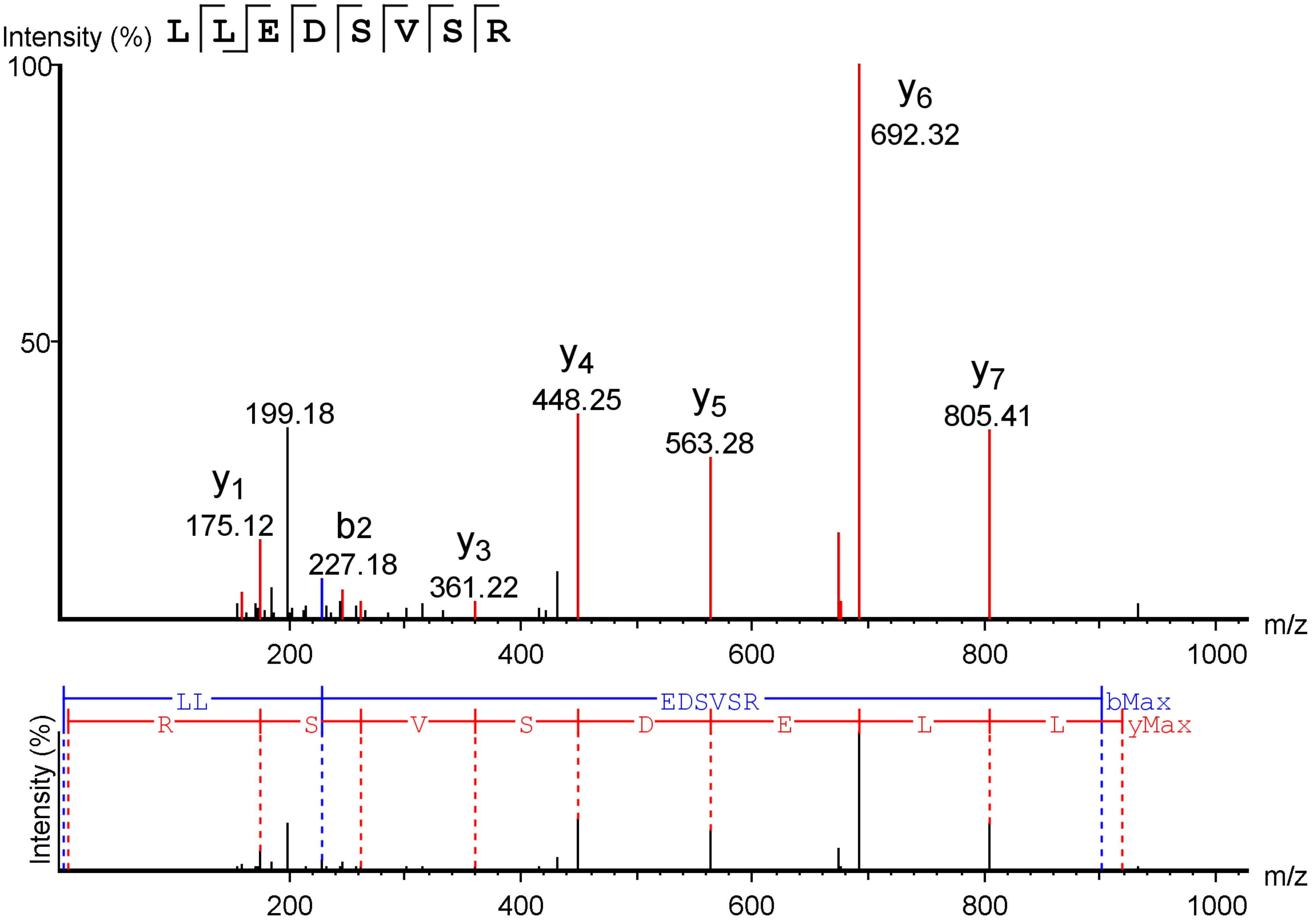

sp|Q15293|RCN1_HUMAN
K.RYIFDNVAK.V

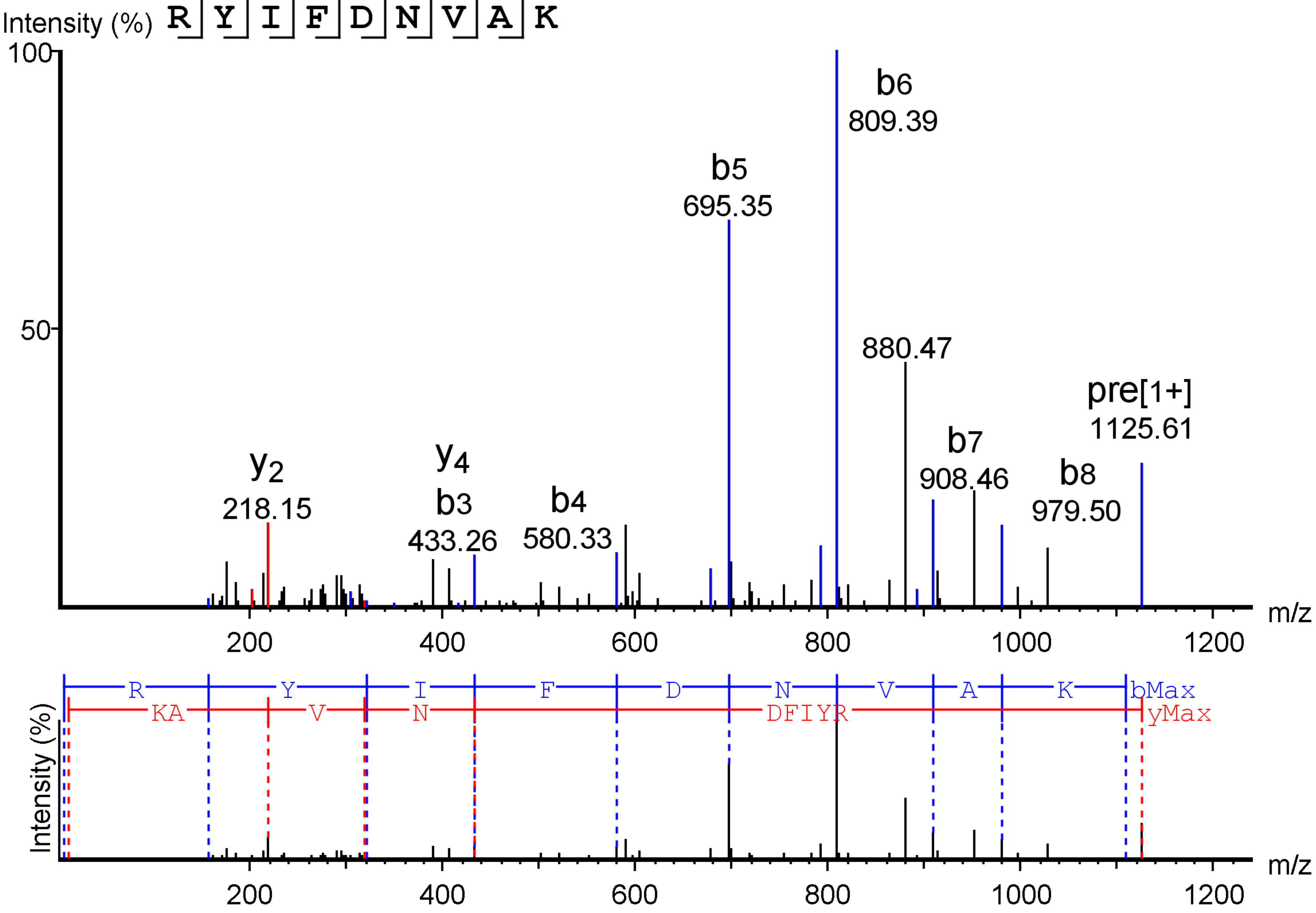

sp|A0A0J9YXX1|HV5X1_HUMAN
K.KPGESLR.I

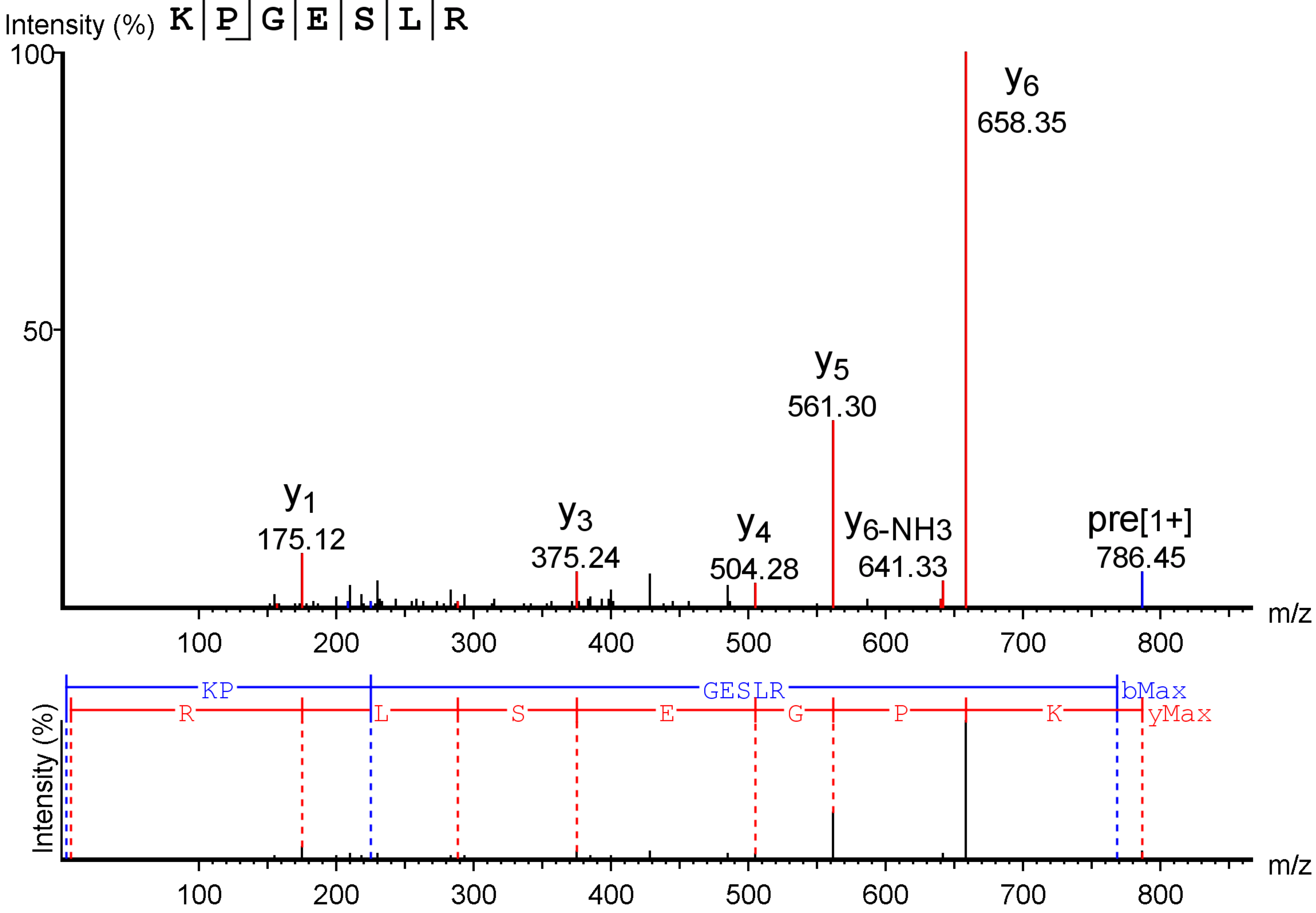

sp|P61970|NTF2_HUMAN
K.LSSLPFQK.I

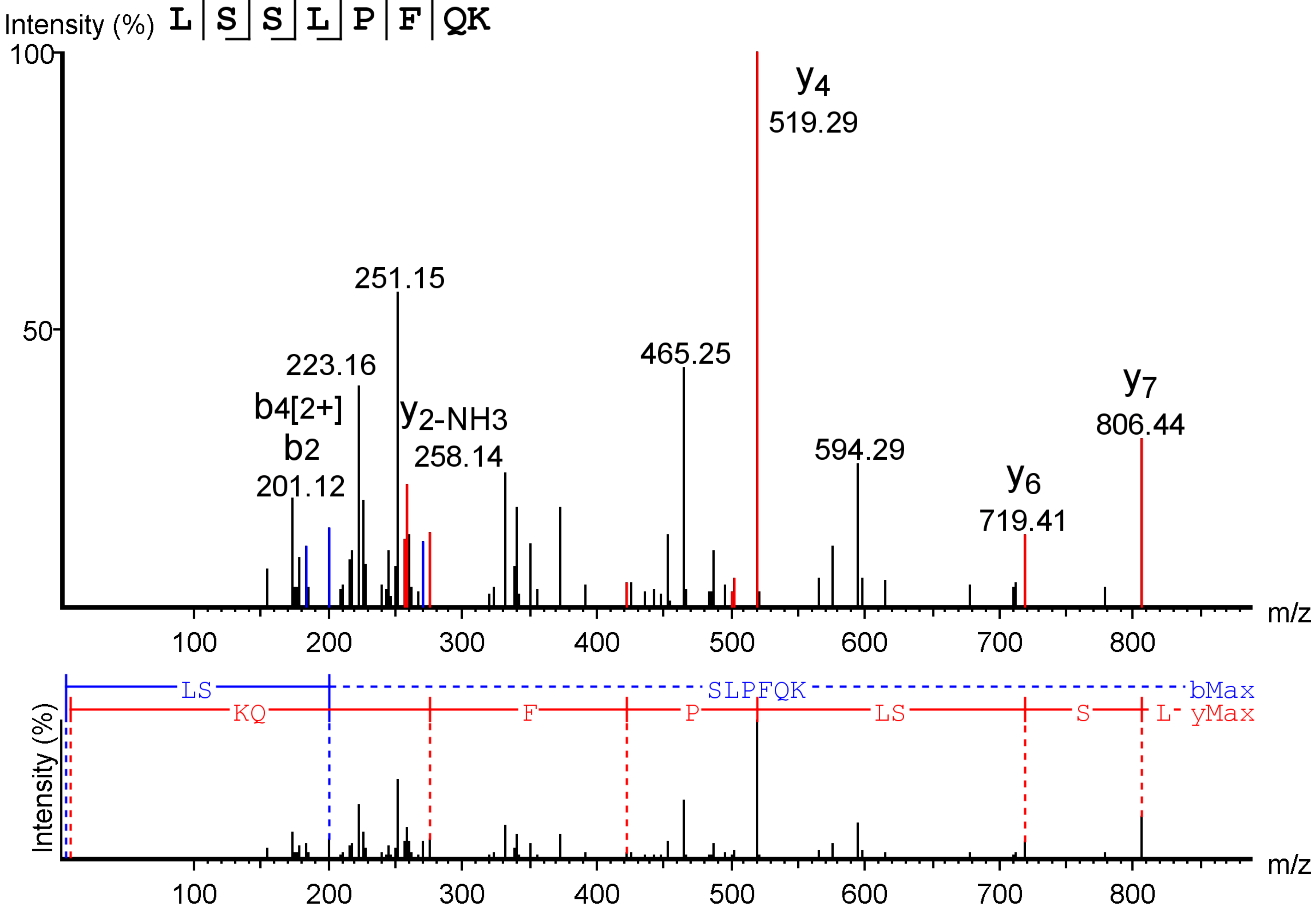

F1T0I1|F1T0I1_HUMAN
K.APGDLPAAGGPPSGAMPFYNPAQLAQAC(+57.02)AT(+79.97)S(+
79.97)GSSR.L

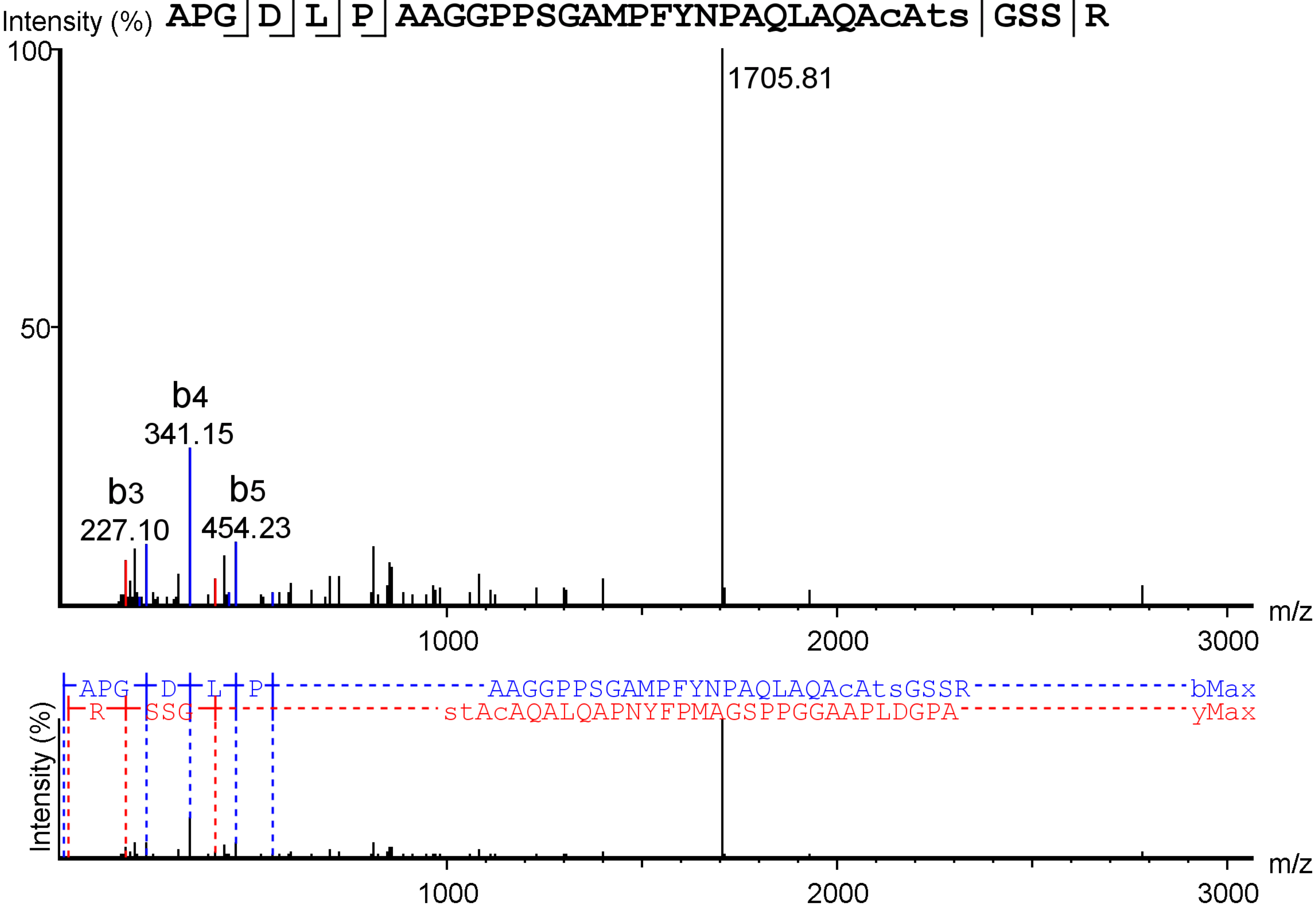

sp|Q92945|FUBP2_HUMAN
R.IGGGIDVPVPR.H

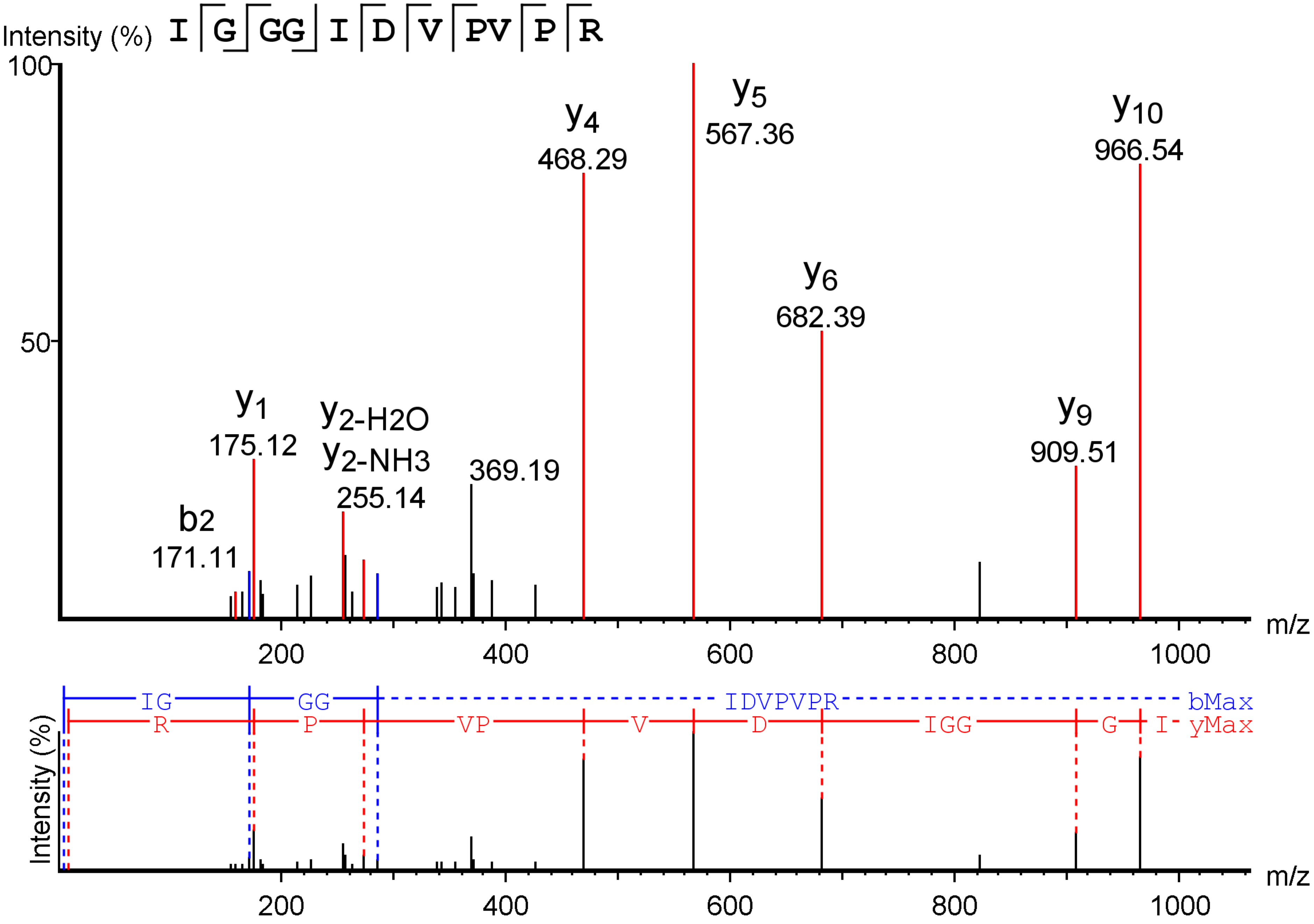

sp|O75973|C1QRF_HUMAN
R.TDGGDALSEQSGAPPPSTLVQGPQGKPGR.T

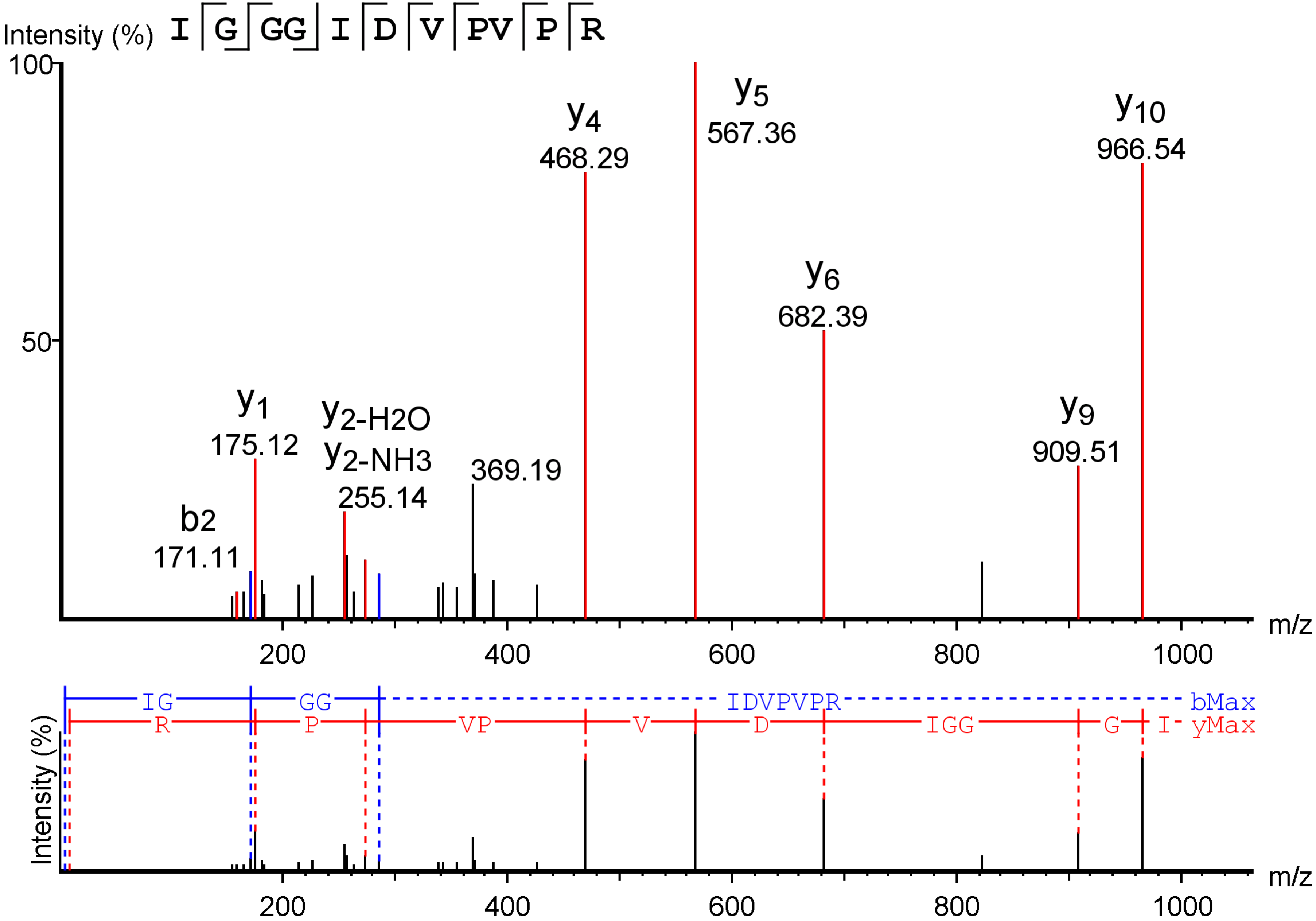

sp|Q6VVX0|CP2R1_HUMAN
R.YFGYGQK.S

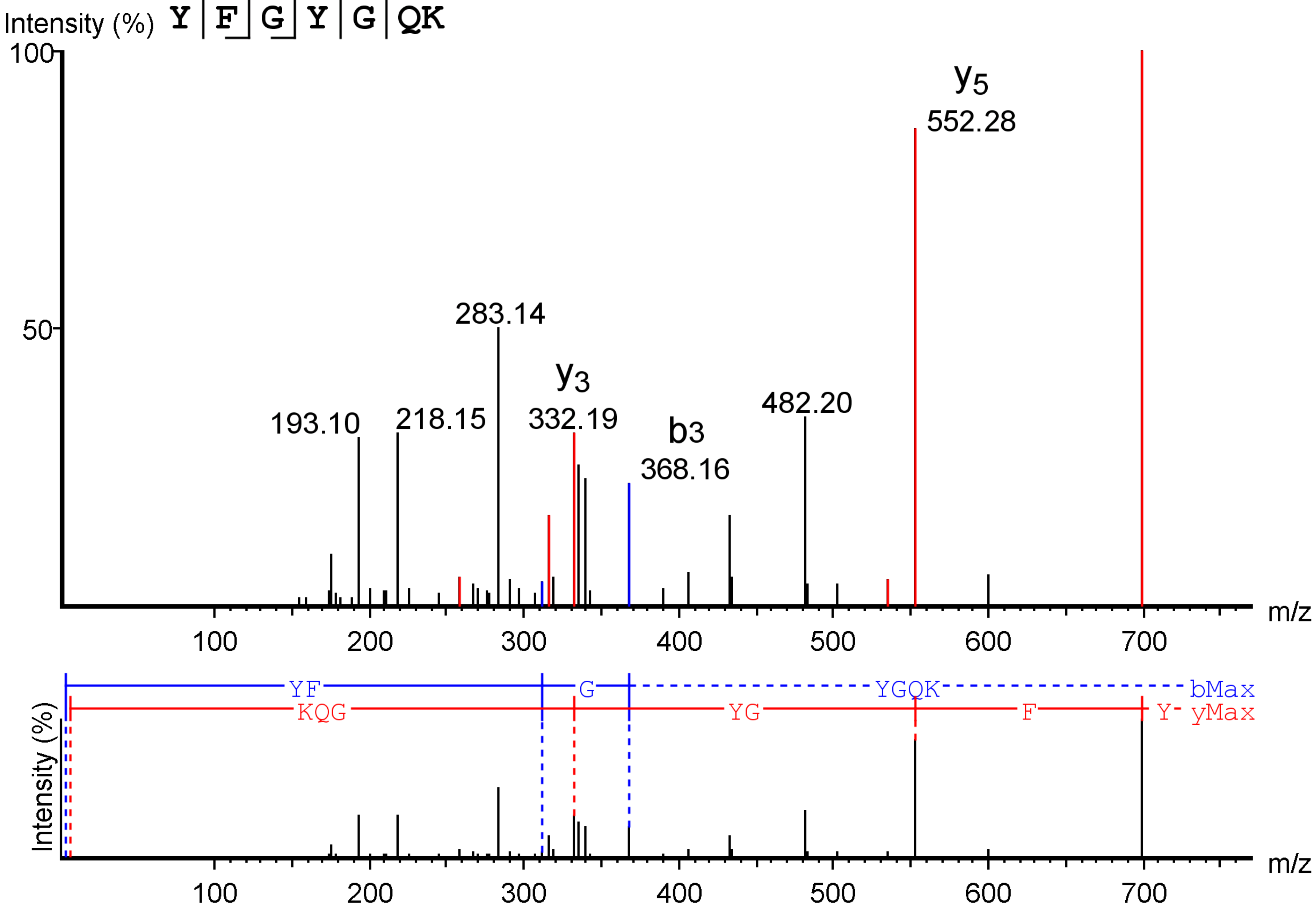

sp|Q8TE60|ATS18_HUMAN
R.TAEEKIQR.Y

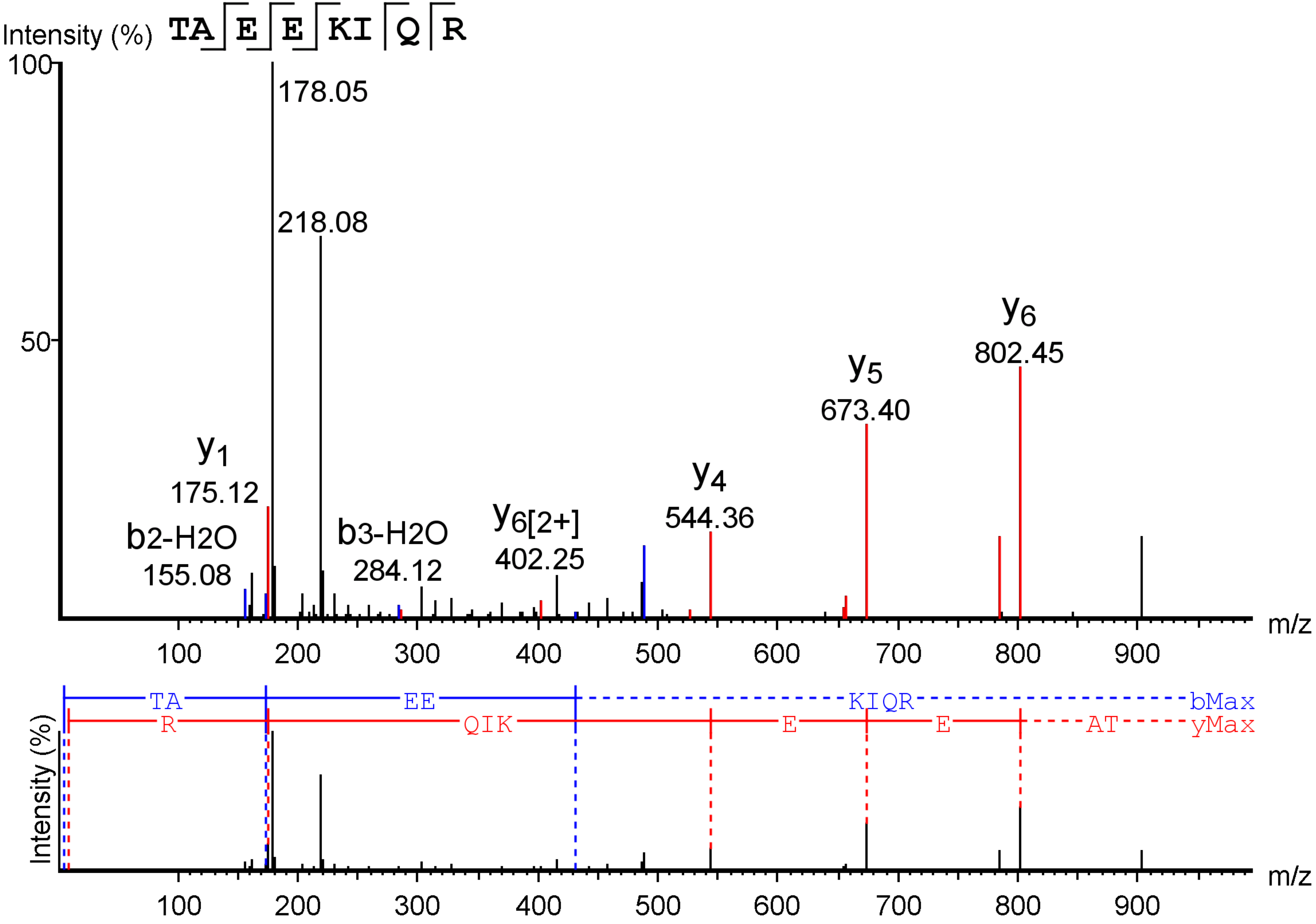

sp|P12259|FA5_HUMAN
K.LSEGASYLDHTFPAEK.M

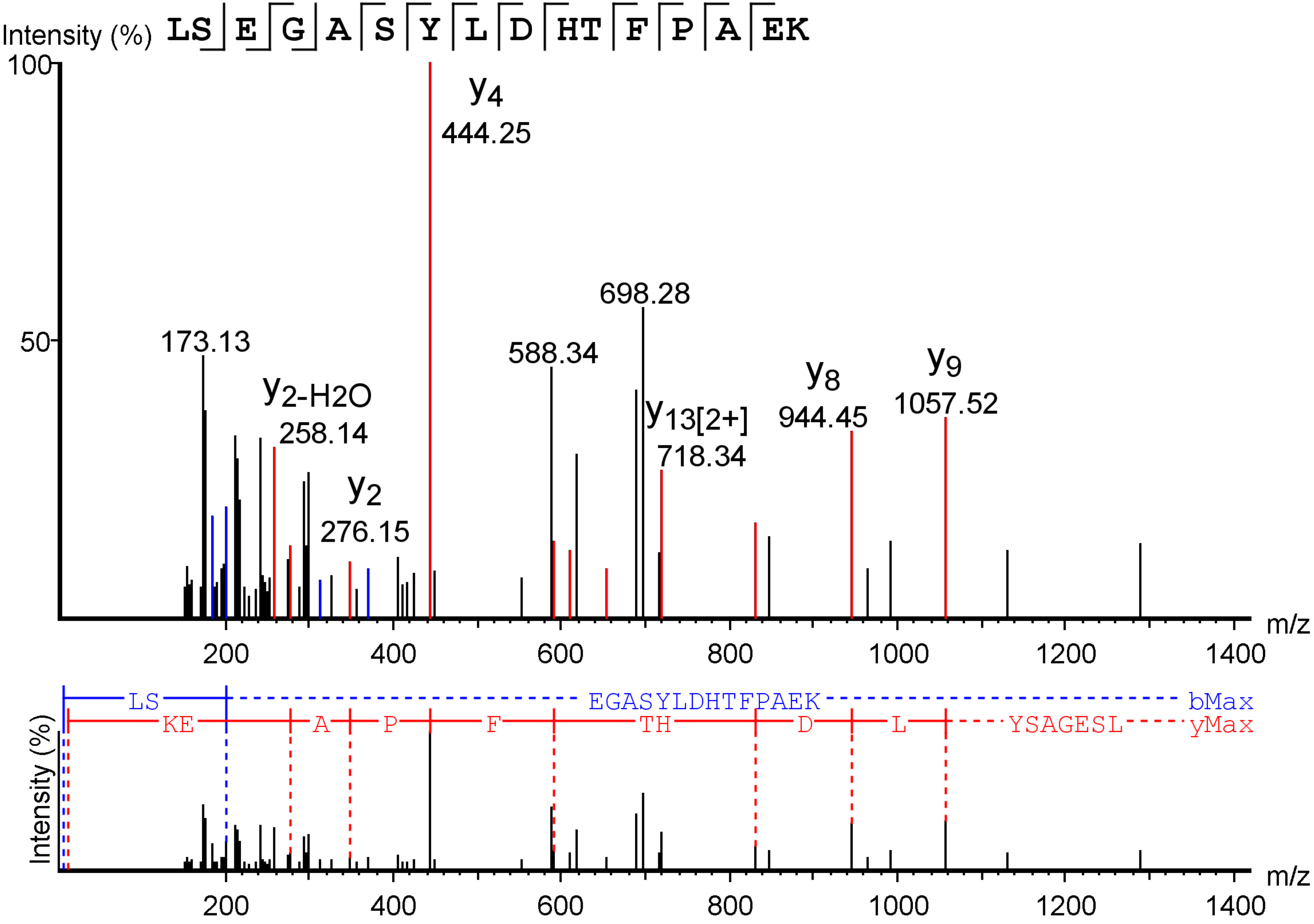

sp|Q9HCJ6|VAT1L_HUMAN
R.AVVLAGFGGLNK.L

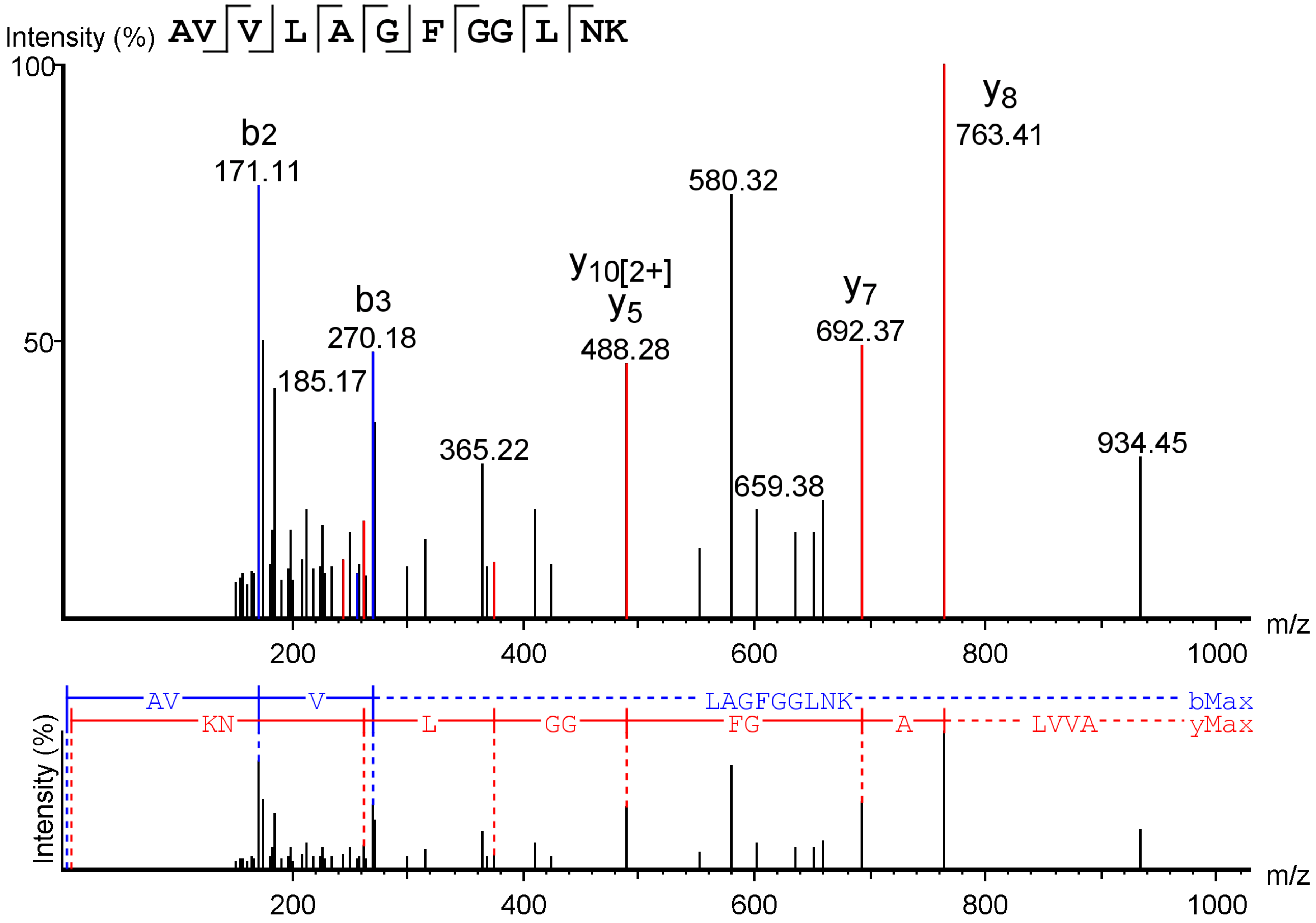

Supplement: Supplement 5. Single peptide ID Spectra [file 160003_0_supp_506365_q8dy2b.pdf]
